# Multi-omics analysis of multiple missions to space reveal a theme of lipid dysregulation in mouse liver

**DOI:** 10.1038/s41598-019-55869-2

**Published:** 2019-12-16

**Authors:** Afshin Beheshti, Kaushik Chakravarty, Homer Fogle, Hossein Fazelinia, Willian A. da Silveira, Valery Boyko, San-Huei Lai Polo, Amanda M. Saravia-Butler, Gary Hardiman, Deanne Taylor, Jonathan M. Galazka, Sylvain V. Costes

**Affiliations:** 1 Wyle Labs, Space Biosciences Division, NASA Ames Research Center, Moffett Field, CA USA; 2 twoXAR Inc, Mountain View, CA USA; 3 Department of Biomedical and Health Informatics, The Children’s Hospital of Philadelphia, Philadelphia, USA; Center for Mitochondrial and Epigenomic Medicine, The Children’s Hospital of Philadelphia, Philadelphia, USA; 4 Institute for Global Food Security, Queens University Belfast, Belfast, UK; 5 Logyx LLC, Space Biosciences Division, NASA Ames Research Center, Moffett Field, CA USA; 6 NASA Ames Research Center, Moffett Field, CA USA

**Keywords:** Molecular biology, Proteomics, Risk factors, Transcriptomics, Systems biology

## Abstract

Spaceflight has several detrimental effects on the physiology of astronauts, many of which are recapitulated in rodent models. Mouse studies performed on the Space Shuttle showed disruption of lipid metabolism in liver. However, given that these animals were not sacrificed on-orbit and instead returned live to earth, it is unclear if these disruptions were solely induced by space stressors (e.g. microgravity, space radiation) or in part explained by the stress of return to Earth. In this work we analyzed three liver datasets from two different strains of mice (C57BL/6 (Jackson) & BALB/c (Taconic)) flown aboard the International Space Station (ISS). Notably, these animals were sacrificed on-orbit and exposed to varying spaceflight durations (i.e. 21, 37, and 42 days vs 13 days for the Shuttle mice). Oil Red O (ORO) staining showed abnormal lipid accumulation in all space-flown mice compared to ground controls regardless of strain or exposure duration. Similarly, transcriptomic analysis by RNA-sequencing revealed several pathways that were affected in both strains related to increased lipid metabolism, fatty acid metabolism, lipid and fatty acid processing, lipid catabolic processing, and lipid localization. In addition, key upstream regulators were predicted to be commonly regulated across all conditions including Glucagon (GCG) and Insulin (INS). Moreover, quantitative proteomic analysis showed that a number of lipid related proteins were changed in the livers during spaceflight. Taken together, these data indicate that activation of lipotoxic pathways are the result of space stressors alone and this activation occurs in various genetic backgrounds during spaceflight exposures of weeks to months. If similar responses occur in humans, a prolonged change of these pathways may result in the development of liver disease and should be investigated further.

## Introduction

Spaceflight affects several physiological and biochemical processes in astronauts and rodent models. The most reported changes from the space environment are in muscle and bone loss^1,2^. Several studies also indicate that spaceflight affects components of the immune system, including the distribution and functionality of peripheral leukocytes, and activity of cytokines^3^. In addition, several decades of spaceflight investigations have eluded that there are subclinical diabetogenic changes that happen in microgravity^4^.

Research involving spaceflight and simulated ground-based microgravity studies (i.e. bedrest studies) result in manifestation of similar diseases such as diabetes, sedentary states, and aging^4^. Insulin resistance is believed to be a key risk factor for fatty liver disease, which is the hepatic manifestation of metabolic syndrome. To date, little work has been done on other tissues such as liver or pancreas because these tissues are not considered to be at risk for long-term spaceflight missions. However, with the increasing precision of omics technology, the approach to evaluate risk from long space missions may find a new paradigm. For example, liver, a critical organ for mammals, has been largely under-studied in the context of spaceflight environment. It performs array of functions that help support both carbohydrate and lipid metabolism among others functions, such as immunity, digestion, detoxification, amino acid catabolism, bile formation, and vitamin storage^5,6^. It is connected with nearly every system in the body, hence, it is prone to a variety of pathophysiologies.

Non-alcoholic fatty liver disease (NAFLD) is multifactorial in nature and comprises multiple pathways for disease progression. The initial insult is believed to be insulin resistance which could lead to more progressive steatosis with associated hepatitis, fibrosis, cirrhosis, and in some cases hepatocellular carcinoma (HCC)^7^. Two complicated individual stages of the disease are believed to exist, NAFLD which is comprised of non-alcoholic fatty liver (NAFL) and non-alcoholic steatohepatitis (NASH)^7^. NAFL is characterized by steatosis of the liver, involving more than 5% of parenchyma, with no evidence of hepatocyte injury^8^. NASH is a necroinflammatory involving hepatic stellate cells and other pro-inflammatory markers, such as IL-17, causing the liver cells to become injured in a background of steatosis^8^. The mechanism of progression for NAFL to NASH is currently unclear, but early evidence eludes to the sequential development of the disease^7^.

Interestingly, one study from Jonscher *et al*. has reported findings in mice flown for 13 days aboard the Space Transportation System (STS)−135 to have liver damage through accumulation of hepatic lipids droplets, elevated triglyceride levels, and loss of retinoids from the hepatic stellate cells (HSC) lipid droplets^9^. These results were correlated with the activation of peroxisome proliferator-activated receptor alpha (PPARα). PPARα is a transcriptional modulator of genes involved in peroxisomal and mitochondrial β-oxidation, fatty acid transport and hepatic glucose production, the latter being rodent-specific^10^. Pro-inflammatory and acute phase response signaling pathways are negatively regulating PPARα, as seen in rodent models of systemic inflammation, atherosclerosis and non-alcoholic steatohepatitis (NASH)^11,12^. Dysregulation of the PPARα signaling can lead to the generation of markers which are known to be precursor to early nonalcoholic fatty liver disease (NAFLD)-like symptoms^13,14^. Pecaut *et al*. demonstrated later from the same tissue that there were also increased fatty acid oxidation and decreased insulin levels^15^. In addition, Blaber *et al*. was able to uncover that a decrease with the defense mechanisms associated with hepatic oxidation (i.e. involving genes associated with autophagy and ubiquitin-proteasome) can induce mitochondrial dysfunction through senescence from a multi-omics analysis utilizing rodent livers from the STS-135 mission^16^. Lastly, several proteomic studies on the liver related to NAFLD have revealed key pathways being regulated in the liver which include cell cycle, glucose metabolism, autophagy, ketogenesis, and fatty acid transport that corresponds with what is being observed with the spaceflight samples^17,18^.

Astronauts’ data have shown similar physiological changes involving diabetogenic responses to microgravity such as reduced glucose tolerance, increased plasma glucose, relative decrease in circulating insulin and increased C-peptide excretion and insulin resistance^4^. Since astronauts and cosmonauts displayed subclinical glucose intolerance and issues with insulin secretion and metabolism^4^, deeper study of the effects of microgravity on liver function are essential to understand the implications of long-term space travel.

One important confounding factor for experiments conducted on the Space Shuttle is that animals were typically brought back live, with animals experiencing the stresses of re-entry and brief re-adaptation to Earth gravity. Sacrifice of animals were done 3.5 to 5.5 hours from de-orbit for mice from the STS-135 missions. Liver is known to be susceptible to stress, and live return may therefore have elicited molecular changes in the organs collected^9^. In other words, previous observations done on Space Shuttle samples may partially reflect changes in the liver that were only transient and were not induced by life in space.

In this study, we performed histology on archived liver tissues obtained from Ames Life Sciences Data Archive (ALSDA) from mice flown to the International Space Station (ISS). In these samples, mice were sacrificed in space and the experimental duration was much longer, removing the stress factor that could be observed when collecting tissue near the travel time. The same tissue had already been processed for both transcriptomics and proteomics by the GeneLab team and raw data was archived on NASA’s GeneLab database platform^19–21^. We therefore conducted pathway analysis on data from the same animals to see what molecular changes were revealed by omics. Our results demonstrate large changes in lipid localization and lipid and fatty acid metabolism and processing, regardless of strain and flight conditions. We confirmed that PPARα signaling is modified in the space environment. In addition, we also identified novel pathways and factors, which are correlated with early onset of liver diseases involving GCG and the circadian clock pathway. We believe this comprehensive study on the impact of spaceflight induced liver damage warrants more investigations at the human level.

## Results

### Histology of mouse liver flight samples indicates liver damage

**Figure 1:**
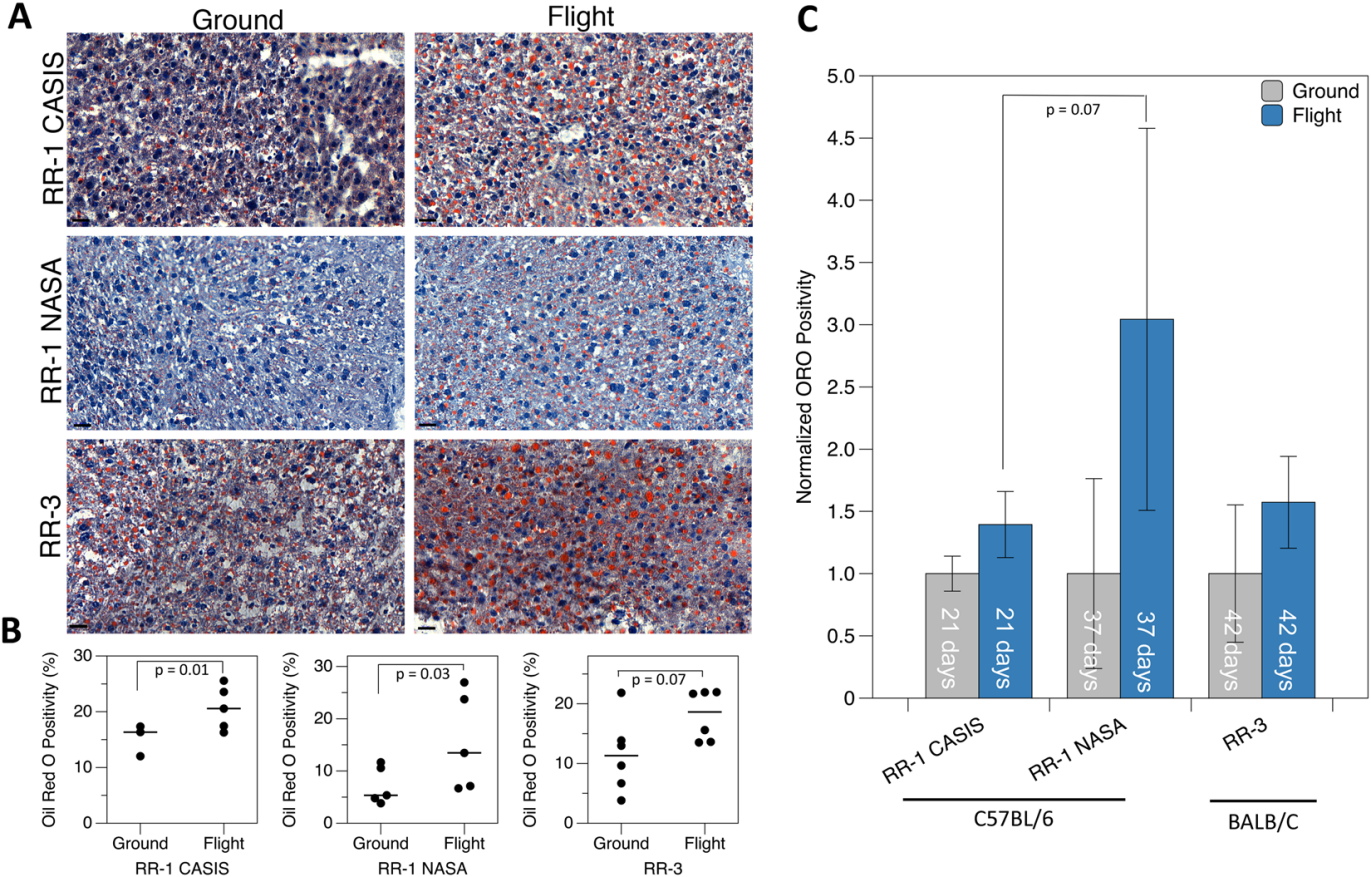
Oil Red O (ORO) staining in flight and ground mouse liver samples from RR-1 CASIS, RR-1 NASA and RR-3 missions. (**A**) Representative images of Oil Red O staining performed on OCT embedded frozen liver sections from flight and ground for all missions. Scale bar = 20 mm. (**B**) Flight samples from all missions show higher percent of tissue area stained with ORO indicative of liver damage across both strains of mice (unpaired Mann Whitney (Wilconox) test). (**C**) Temporal analysis of percent change of ORO lipid positivity in mice liver. Significant increase in lipid deposition is observed in livers of C57/BL6 exposed to microgravity for longer duration of time (unpaired Mann Whitney (Wilcoxon) test).

To assess the severity of steatosis and inflammation, liver samples were stained in hematoxylin and eosin (H&E) and ORO and analyzed by light microscopy and digital image analysis (DIA)^22^ (Fig. 1A). Jonscher *et al*. had originally shown in the livers of C57BL/6 mice that were flown on the STS-135 mission that there was an increase of ORO stain indicating increased levels of lipid accumulation in the liver^9^. One large confounding factor in this work was the fact that animals were returned live to Earth and thus suffered significant stress related to such return. Since their publication, multiple other rodent missions have been flown to the ISS which do not suffer from this confounding factor with animals sacrificed on the station. We have therefore utilized mice liver samples from both the Rodent Research 1 (RR-1) and Rodent Research 3 (RR-3) missions to further confirm whether increases in lipids in the liver is the result of spaceflight alone. As shown in Fig. 1B, flight samples from the RR-1 NASA and RR-1 CASIS missions showed significantly (p-value < 0.05) higher ORO staining, than the ground control samples (Fig. 1B), but there was no significant difference between RR-3 flight and ground samples. To determine if the duration of spaceflight exposure was correlated to increased ORO positivity, we compared the percent change in ORO positivity in RR-1 CASIS livers with RR-1 NASA livers, in which mice were exposed to 21 days and 37 days of spaceflight, respectively. However, the difference between these groups was not significant (p-value = 0.07) (Fig. 1C).

In addition, a two-way ANOVA was done on the normalized data, using the mission (i.e. RR-1 NASA, RR-1 CASIS, RR-3) and flight conditions (i.e. Flight vs Ground) as two different factors. A P-value of 0.0008 was found for the flight condition factor, clearly identifying space environment as the most significant factor influencing the level of ORO in the liver. However, ANOVA test also revealed a P-value of 0.0509 for the impact of mission, indicating each mission had significant impact on the difference of ORO level. When testing for interaction between the two factors, P-values dropped to 0.0004 for the flight condition and to 0.0323 for the mission factor with a significant interaction between mission and flight conditions. This suggests that the mission itself affects significantly the relationship between ORO level in the liver and space environment, with RR1-NASA samples showing the largest impact. One could interpret these results as duration having a significant impact on ORO level for the 37-day mission with C57BL/6 showing a significant increase of ORO in the liver compared to the 21-day mission. Similarly, the significant difference between RR-3 and RR-1 (i.e. 42-day and 37-day mission respectively) is probably reflecting a strain difference and ideally one would need an equivalent experiment for the duration of flight for the BALB/C to conclude definitively. We conclude that increasing exposure to microgravity-based chronic perturbation increases the lipid accumulation in a strain independent manner.

### Global transcriptomics differences occur between mouse liver flight samples and ground controls

To further investigate molecular changes that will occur in the liver during spaceflight that can cause the histological changes observed, we utilized existing omics datasets from GeneLab which included: GeneLab Datasets (GLDS)−25, GLDS-47, and GLDS-168. GLDS-25 contained transcriptomics data (i.e. microarrays) from livers flown on the STS-135 shuttle mission (female C57BL/6 mice flown for 13 days on STS-135) while GLDS-168 and GLDS-47 contain RNA-sequencing data for liver samples from mice flown to the ISS for both the Rodent Research 1 (RR1) (female C57BL/6 mice flown for 37 days on the ISS) and RR3 missions (female BALB/c mice flown for ~40 days on the ISS), respectively. GLDS-47 data were gathered by CASIS while GLDS-168 data was gathered by NASA. As mentioned in the previous section, Jonscher *et al*. had previously performed analysis on the liver for the STS-135 mission^9^. They performed transcriptomic and metabolomics to uncover genes and metabolites that changed during spaceflight associated with lipid accumulation in the liver that seemed to be driving PPAR response. For our analysis not only did we utilize the STS-135 dataset, but also used complementary transcriptomic and proteomic technology on datasets that did not suffer from live return. This allowed us to confirm that spaceflight alone is eliciting such significant changes in the liver.

**Figure 2:**
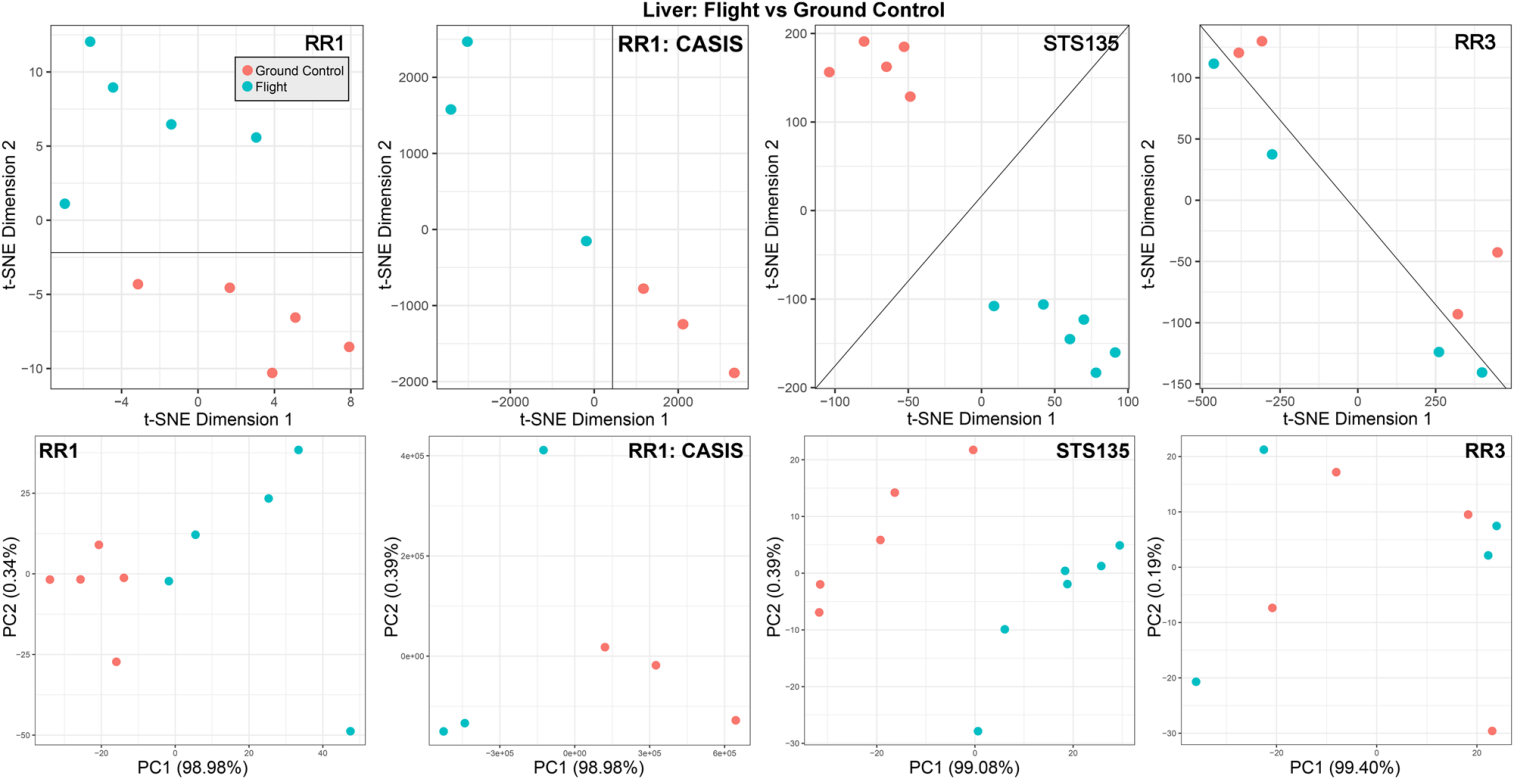
The global transcriptomics differences on livers from mice flown in space compared to ground controls. Clustering of individual biological liver samples from mice for each transcriptomics dataset (STS-135, RR1, and RR3) from GeneLab utilizing t-Distributed Stochastic Neighbor Embedding (t-SNE) (upper plots) and Principal Component Analysis (PCA) (lower plots) comparing Spaceflight samples to ground controls. Lines are drawn in the t-SNA plots to visually separate the flight samples from the ground controls. The axis for t-SNE plots are arbitrary. The variance for the PCA plots is indicated in the axis. The coordinates of each point in each plot represents the two most important gene pattern contributions (e.g. principal component for PCA) of individual biological replicates. Each replicate is obtained from individual mice exposed to the same experimental conditions. Two points in close proximity suggests a very similar gene expression pattern between the two corresponding samples.

Overall, t-Distributed Stochastic Neighbor Embedding (t-SNE) and Principal Component Analysis (PCA) plots show strong statistical separation between the liver flight samples and the ground controls (Fig. 2), independent of duration, flight condition, and strain. This indicates that large transcriptomics changes are occurring in the liver during spaceflight and warrants more detailed pathway analysis to determine the key functions driving the changes in the liver during spaceflight.

### Pathway analysis reveals upregulation of lipid functions and regulation in the liver of flight samples

We utilized pathway analysis tools to fully understand the global impact of spaceflight on the liver tissue. Through Gene Set Enrichment Analysis (GSEA)^23^ we determined the significant pathways (or gene sets) with a FDR ≤ 0.05 that are either activated or inhibited between livers from spaceflight versus the ground controls. We performed GSEA analyses on both Reactome pathways^24^ and the Gene Ontology (GO) biological process ontology^25,26^, clearly identifying the key pathways that were regulated in mice flown on the shuttle missions (STS-135). Since the Reactome pathways led to a less complex network, we will only report and discuss these pathways for the STS-135 data (Supplemental Fig. 2). We also provided the list of GO pathways as Supplemental Table 1. We observe that the majority of pathways being regulated in the liver are upregulated (i.e. red nodes in Supplemental Fig. 2), with a strong association to changes in lipids and lipoproteins metabolism^27^. This activation correlates with the lipid accumulation observed with the histology (Fig. 1). In addition, the activation of protein metabolism that is observed can be potentially linked to liver disease, since it has been previously reported that the dysregulation of this pathway is often associated with deamination and transamination of amino acids, disruption in amino acid kinetics, and possible cirrhosis^28^.

GSEA analysis was performed using GO pathways on the RNA-seq data for both female C57BL/6 and BALB/C mice that were flown on the ISS for ~40 days (RR1 and RR3 mission). We observed similarities and differences between all data sets (Supplemental Fig. 3). More specifically, nodes/pathways associated with lipid and fatty acid processing and metabolism were upregulated in both shorter missions on the shuttle (STS-135) and longer mission in the ISS (RR1, RR3), as denoted with yellow background in both Supplemental Figs. 2 and 3. In contrast, there are many more pathways that were oppositely regulated in livers between the two different strains (RR1 and RR3 missions). This observation is consistent with recent reports^29^, suggesting inherent differences in liver response between C57BL/6 and BALB/C, with differences in immune cell types, liver cytokine profiles, liver steatosis, degree of adiposity, and glucose levels. It has been reported under a normal diet that BALB/C mice compared to C57BL/6 mice have more pronounced liver steatosis and body weight gain. On the other hand, C57BL/6 mice exhibit higher weight gain, increased collagen deposition, and an increase in visceral adipose tissue on a high-fat diet^29^. Potentially, these differences related to body weight gain and collagen changes between the strains can account for the differences observed in the strains with the catabolic process associated pathways. Further studies should be done to investigate these differences, but for the focus of this manuscript we will be comparing the common pathways that are being regulated in the liver due to spaceflight.

**Figure 3:**
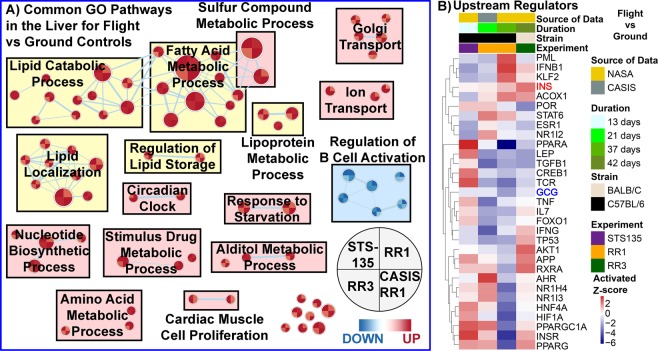
Common pathway analysis for livers from mice for all datasets (STS-135, RR1 and RR3 mission). (**A**) Only displaying the common Gene Set Enrichment Analysis (GSEA) with Gene Ontology (GO) pathways on liver’s from STS-135, RR1 and RR3 GeneLab datasets displayed as a network through a Cytoscape plugin, EnrichmentMap^73^. Red nodes indicate upregulation of the pathway and blue nodes represent downregulation. Lipid related pathways are shown with a yellow background. Each node represents one gene set and the size of the node indicates the number of genes involved with the predictions. Each node contains 4 wedges for each condition and the color of each wedge indicates if the gene set is downregulated (blue) or upregulated (red). The shade of the color indicates degree of regulation. The thickness of the edge (blue lines) represents the number of genes associated with the overlap of the gene sets (or nodes) that the edge connects. Clusters were named according to common function in each grouping. (**B**) Upstream regulator analysis utilizing Ingenuity Pathway Analysis (IPA) on the significantly regulated genes (FDR < 0.05). The results are shown only for the common upstream regulators across all conditions (STS-135, RR1, and RR3) as a heatmap of the activated z-scores.

When comparing only the common GO biological processes in the liver for spaceflight samples versus ground controls across all three datasets (i.e. RR1, RR3, and STS135 missions), we once again see lipid and fatty acid related processes being predominantly upregulated with a fairly uniform response across the three experimental groups (Fig. 3A). Other pathways found to be upregulated here are ion transport, alditol metabolic process, and vitamin/drug metabolic processing, all potentially associated with lipid functions. Interestingly, circadian clock related pathways were also commonly upregulated in the liver, which has been shown in the literature to greatly impact the lipid function and be involved with NAFLD^30^. When testing the same data with proprietary tools such as Ingenuity Pathway Analysis (IPA), several upstream regulators were predicted to be present in all conditions (Fig. 3B). Two upstream regulators were commonly regulated across all conditions, with Glucagon (GCG) being commonly downregulated and Insulin (INS) being commonly upregulated across all datasets and conditions. Although, GCG and INS are more commonly found to be involved with pancreatic functions to regulate blood sugar levels, it has also been shown that such signals in the liver can play a role with disease state. For example, an upregulation of INS can provide the liver with high blood glucose signals. In contrast GCG allows the liver to convert glycogen to glucose when the blood sugar levels are low^31^, and thus downregulation of GCG would interrupt such conversion eventually lowering blood glucose levels. Therefore, we propose that the upstream effectors that are predicted to regulate these changes are GCG and INS, which may be responding to physiological conditions that are observed during periods of high blood glucose levels. Similarly, these two upstream regulators are also known to be involved with lipid and fatty acid functions^32^. Overall, we believe that we have shown spaceflight induces in the liver of mice driving factors that might be responsible in driving NAFLD pathogenesis.

### Duration changes in livers from mice flown in space

Although the theme of this manuscript is determining functions in the liver that will be impacted by spaceflight independent of duration and strain, we thought it will be important to also briefly discuss the differences in biological functions that will occur as a function of duration. For this analysis we have utilized data from the same strain mice (i.e. C57BL/6) with two different durations in space on the ISS (21 and 37 days) to remove any additional confounding factors. This comparison will give us some insight on potential biological changes that will occur with longer exposure to the space environment on the liver. As in the previous section, for comparisons between different GeneLab datasets and omics platforms, we performed the analysis with the normalized enrichment scores (NES) determined through GSEA GO terms.

**Figure 4:**
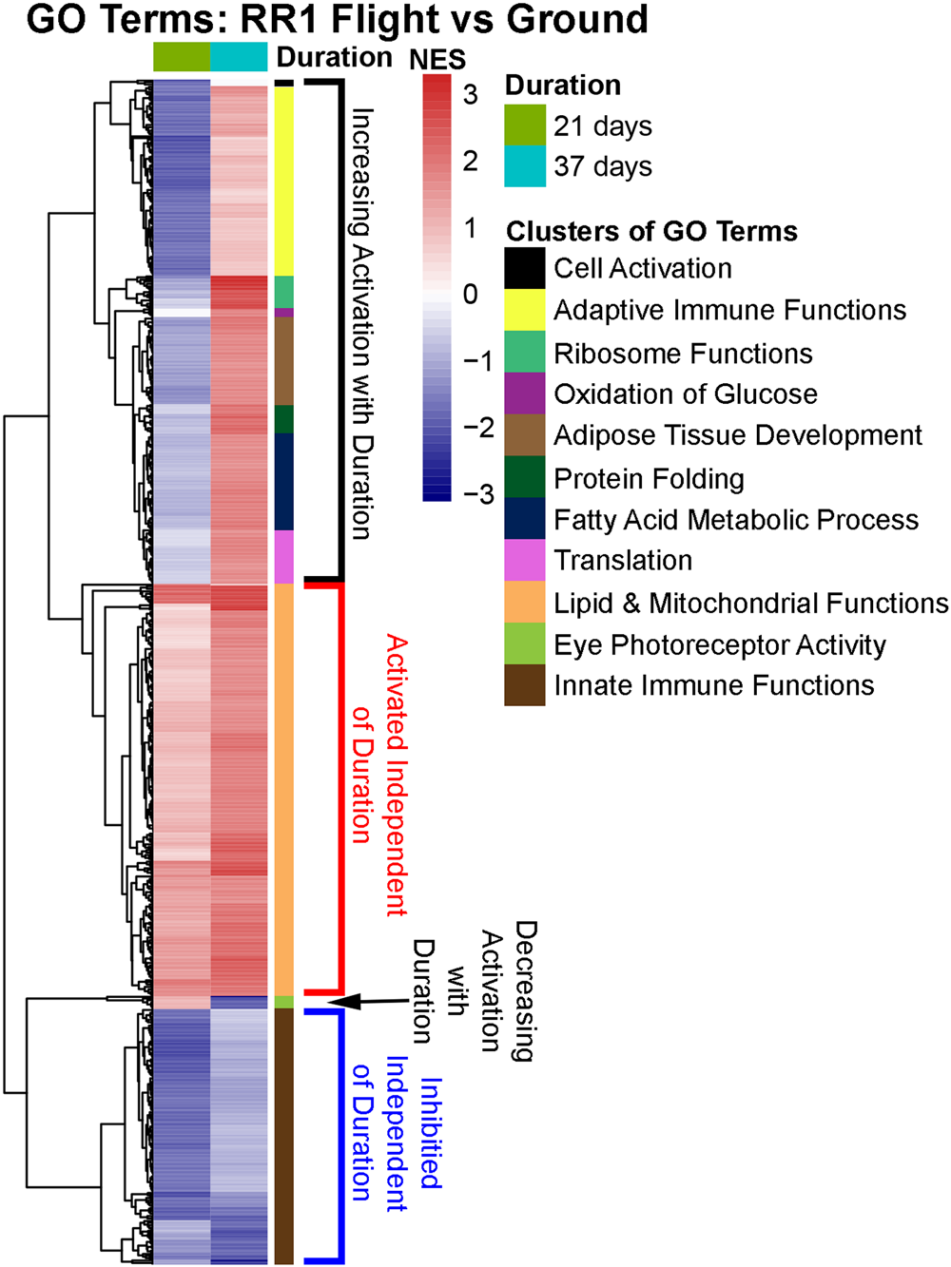
Pathway differences occurring in the liver as a function of duration in space. Comparison of the GO terms through the NES between the RR1 datasets with FDR ≤ 0.05. The right colored side bar on the heatmap indicates pathways grouped together with common functionality. In the heatmap the blue color represents inhibition determined by negative NES and the red color represents activation determined by positive NES.

To study the impact of duration in space on the liver, we directly compared the GO terms from the GSEA analysis containing FDR ≤ 0.05 between the two RR1 datasets (Fig. 4). We observed, in agreement with the previous results, that pathways related to lipid and mitochondrial functions were activated independent of duration in space. In addition, pathways related to innate immune functions were inhibited independent of duration in space which was also shown in the previous section for all datasets (Fig. 3A).

There were a set of pathways which were shown to be increased with longer duration in space in the liver (Fig. 4). The majority of these pathways are related to increases in the adaptive immune system. It has been previously shown that long exposure to the space environment does indeed activate persistent adaptive immune system pathways which will have potential to impact spaceflight associated health risks linked to reactivation of latent herpesviruses and increased incidence of infectious diseases^3,15,33^. Other pathways that were increased with longer space duration were oxidation of glucose, adipose tissue development, protein folding, fatty acid metabolic process, translation and ribosome functions, protein folding, and cell activation (Fig. 4). Oxidation of glucose and adipose tissue development are directly impacted by the adaptive immune system^34,35^ and both have been previously linked with adaptive immune system changes during spaceflight^3,15^. In addition, protein folding, translation, and ribosome pathways have been directly linked to activation of the adaptive immune system^36,37^. The increase in the fatty acid metabolic process pathways are in agreement with our results in the previous sections indicating an increase in the lipid accumulation as a function of duration in space (Fig. 1). These increases that occur during longer spaceflight are still in agreement with our observed results in the previous sections regarding accumulation of lipids in the liver, since increases in the adaptive immune system have also been associated with dysregulation with lipid and fatty acid functions^38^.

### Proteomics analysis on livers from mice flown in space exhibit lipid related proteins driving lipid metabolic processes

**Figure 5:**
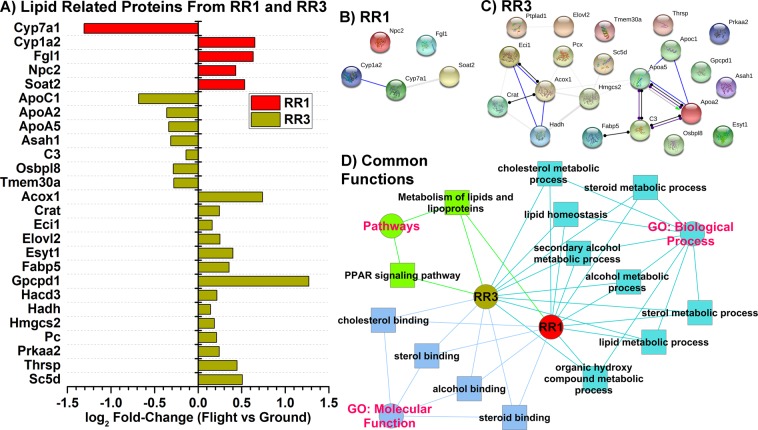
Lipid related proteins from proteomics analysis on livers from mice for RR1 and RR3 datasets. (**A**) The fold-change (log_2_) values for the lipid related proteins comparing livers from mice from both RR1 and RR3 GeneLab proteomics datasets. (**B**,**C**) RR1 and RR3 lipid related protein interaction networks predicted using STRING^41^, which considers both direct (e.g. co-IP) and indirect (e.g. co-expression, text mining) interactions. (**D**) The overall predicted common Gene Ontology (GO) molecular functions, GO biological processes, and pathways being regulated by all the lipid proteins for both RR1 and RR3 proteomics datasets determined by ToppCluster in the ToppGene Suite^42^. Cytoscape^43^ was utilized to displaying the connected network of functions. The colors for the node indicate the group the functions are associated with. The edges (connecting lines between the nodes) represent the connection between either the RR1 or RR3 proteins to the predicted pathways (green line), molecular functions (blue line), or biological process (aqua line).

Since transcriptomics data does not always translate directly to protein functions, we analyzed the proteomics data available on GeneLab for livers from the same mice as for the transcriptomics data. The only proteomics data available on GeneLab were for mice flown on the RR1 and RR3 missions. From the proteomics data, we determined the significantly regulated proteins (p-value ≤ 0.05) in the liver for spaceflight versus ground controls. From these proteins, we focused only on the proteins related to lipid functions and pathways chosen from the GO related annotations (Fig. 5A–C). From this analysis there are less significantly regulated lipid related proteins in RR1 mice than RR3 mice (Fig. 5A). This difference in the number of lipid related proteins can possibly be due to the strain differences between the two rodent missions (as discussed earlier). Although, these differences occur, we chose to focus on the common pathways that the proteins are regulating since it is known that different proteins can impact common pathways. The lipid related proteins for the livers from the RR3 mission were being expressed according to how the NAFLD pathogenesis will form. For example, the Apolipoproteins (ApoC1, ApoA2, and ApoA5) were being downregulated in the flight samples (Fig. 5A). For RR1 related lipid proteins we also observed that Fgl1 is upregulated. In addition, Cyp7a1 is known to be heavily involved with bile acid production and are known to impact both insulin production and NAFLD progression^13,39^. Cyp1A2 is increased with our proteomic data, and is an enzyme involved with detoxification. Cyp1A2 has been previously detected in livers of mice flown in space and with its levels returning to normal after the mice have reacclimated on Earth^40^. Interestingly, these proteins that are known to be associated with lipid functions are highly connected together in action and function (Fig. 5B,C) determined by inputting these proteins in STRING^41^ to construct a protein interaction network.

Lastly, we predicted the overall functional impact of the specific lipid proteins for livers from both RR1 and RR3 mice (Fig. 5D). The common predicted functions were determined by entering the lipid related proteins for RR1 and RR3 (Fig. 5A) in ToppCluster in the ToppGene Suite^42^ and displaying the connected network of functions for both RR1 and RR3 related lipid proteins with Cytoscape^43^. From this analysis it was revealed that the lipid proteins from the liver of the mice for both RR1 and RR3 missions shared common pathways potentially impacting lipid related metabolic processes and cholesterol binding pathways (Fig. 5D). Further research should be conducted to determine actual functional impact. In addition, it was revealed that these lipid proteins are directly linked to the PPAR signaling pathway. Jonscher *et al*. had previously demonstrated from work done on mice from the STS-135 mission, that PPARα was a highly upregulated pathway in the liver of these mice due to spaceflight and played an important role in lipid metabolism^9^. They believed that this regulation in the liver of PPARα due to spaceflight can be an indication of early NAFLD disease progression^13,14^.

## Discussion

The current investigation demonstrates fluctuations in the temporal patterns of biochemical signal transduction pathways caused by microgravity in three distinct spaceflight datasets (RR1-NASA, RR1-CASIS and RR3) involving RNA-seq and proteomics data analysis compared to microarray data from STS-135. Intriguingly, a subset of the metabolic pathways and molecular factors seem consistent with NAFLD (nonalcoholic fatty liver disease), a multi-factorial metabolic disease that upon progression could lead to NASH (nonalcoholic steatohepatitis) and finally irreversible liver fibrosis^13,14,27^. This subset of metabolic pathways include activation of lipid related pathways (in Fig. 3: lipid catabolic process, lipid metabolism, fatty acid metabolic process, lipid localization, lipoprotein metabolic process, and regulation of lipid storage), dysregulation of PARR pathways, Apolipoproteins, INS, and GCG (Figs. 3B and 5).

Lipid metabolism in spaceflight environments, specifically in the realm of lipid homeostasis, remains largely unclear to date. Mice flown in short mission STS135^9^, demonstrated major perturbations in key regulatory genes in lipid and fatty acid metabolism. Such perturbation is often thought to be the “first-hit” of NAFLD^44^ characterized by insulin resistance and perturbed fatty acid metabolism leading to liver lipid accumulation and steatosis^45^. Similarly in this work, histopathological analysis of hepatic lipid content as a biomarker demonstrated marked increase in ORO positivity in all space missions (Fig. 1). Upregulated pathways in these results have a direct impact in dysregulation of the lipids and contribution to increased risk of NAFLD. Our proteomic analysis showed that apolipoproteins were inhibited (Fig. 5). From the literature apolipoproteins have been shown to be involved in the formation of lipoproteins by binding to liver derived lipids and inhibition of the apolipoproteins can result in increased chances of NAFLD^46^. The upregulation of GCG is known to decrease fatty acid synthesis, synthesize additional glucose by gluconeogenesis, and increase lipolysis in the liver^32^. GCG and INS are also known to be involved in a feedback loop designed to maintain homeostasis for the blood glucose levels^32^. Our analysis indicates that INS is indeed upregulated while GCG is downregulated in the liver indicating that there can be an increase in lipid accumulation as we observed with the histology (Figs. 1 and 3). The upregulation of glucose metabolism that we observe has also been reported to contribute to liver disease through disruption of the normal glucose homeostasis that the liver maintains and also involved in insulin resistance^47^. The cell cycle pathways are observed to be upregulated and it has been reported that during excessive cell division that lipid accumulation can occur in the dividing cells contributing to dysregulation for lipid metabolism^48^. Lastly, we observe an increase in carbohydrate metabolism which has been reported to be associated with increased rate for the development of NAFLD^27^.

PPARα-mediated pathways were found to be activated in the proteomic data (Fig. 5), and mostly predicted to be downregulated in the transcriptomic data (Fig. 3B). Such downregulation has been associated to the “second-hit” phase of NAFLD known to lead to inflammation and fibrosis, an important pathophysiologic criteria for NASH and irreversible liver fibrosis, which also involve mitochondrial dysfunction and oxidative stress^44^. In addition, Fgl1 protein was elevated in the spaceflight livers (Fig. 4). This protein has been associated with late-stage NAFLD development and has been observed to have elevated expression in livers from obese patients with NASH^49^. Other suggestions of the “second hit” mechanism for NAFLD in our data are related to B-cell inhibition, with all datasets (STS-135, RR1, and RR3) (Fig. 3). B-cell regulation has been shown to play a small role with NAFLD progression^50^. Interestingly, the B-cell inhibition can also be involved with immunosuppression that is observed during spaceflight as discussed by Pecaut *et al*. for data that was obtained on mice from the STS-135 mission^15^.

In this work, the observed lipid uptake in liver occurred in both strains, with C57Bl/6J showing a stronger response both by histopathology (Fig. 1) and by a stronger separation of ground and flight gene expression by t-SNE plots (Fig. 2). Such observation is consistent with strain susceptibility of early onset of liver disease. For instance, looking at hyperplasia as an early onset of liver disease, it has been shown that C57Bl/6J have a higher spontaneous level of liver hyperplasia than BALB/cByJ, with 15.45% of young mice (16 weeks)^30^ and 3% of older mice (16–36 months old)^51^ positive for liver hyperplasia. In contrast, only one study^52^ reporting on liver hyperplasia in BALB/CByJ showed 0% of hyperplasia in either female or male, looking at bile ducts. In terms of response to treatment, the study^30^ on younger C57Bl/6J also showed that simply dysregulating the circadian rhythm with modified light cycles was sufficient to increase the level of hyperplasia from 15.45% to 75%. When remembering the difficulty for the animal to stay idle in microgravity^53^, it is not surprising to see the circadian rhythm being upregulated for all datasets and mouse strains (STS-135, RR1, and RR3) (Supplemental Figs. 2 and 3) and provides a source of stress that have been clearly correlated with liver hyperplasia in mice. Finally, C57BL/6 strain also displays severe phenotype of diet-induced NAFLD/NASH associated liver injury, obesity, glucose intolerance, and insulin response through genetic or epigenetic mechanisms compared to BALB/C^54,55^.

Even though everything is done to keep the light cycle as close to Earth as possible in these missions, microgravity most likely modifies sleeping pattern of these mice in space, primarily because of the difficulty for the animal to stay idle in microgravity^53^. Therefore, circadian rhythm seems to be an additional candidate that can contribute to NAFLD disease progression. We observe through our results that indeed circadian clock pathways are being upregulated for all datasets and mouse strains. (STS-135, RR-1, and RR-3) (Supplemental Figs. 2 and 3). In addition to microgravity, there are other factors in space that may also lead to liver disruption. One clear stressor that has been studied extensively in mice are ionizing radiation. However, most radiation studies have been focused on cancer as an endpoint. When searching the literature for the two strains used in our analysis, as observed for hyperplasia, reports were heavily biased towards C57BL/6J. One study^56^ showed how ionizing radiation elicited liver tumors, with 7.69% hepatocellular adenoma detected 92–94 weeks following 4Gy of total body irradiation (TBI) (Cs-137 source; 2.5Gy/min) at 12–14 weeks of age. Interestingly, in this work, mutating the gene Clock was sufficient to block tumor onset, implicating again circadian rhythm in radiation-induced liver carcinogenesis, and thus suggesting that the combination of space environment and disrupted sleep may work synergistically in enhancing liver disease, potentially including cancer. Another study^57^ reported 11.11% increase in hepatocellular carcinoma detected 14 months after radiation exposure to unspecified high doses of ionizing radiation. Nothing could be found in the literature regarding Balb/cByJ and radiation-induced liver tumors. One note of caution here in terms of interpretation is that the radiation doses used in the studies listed above are a thousand-fold higher than the two missions considered here. As one can find in GeneLab metadata for RR-1 and RR-3 missions, the total dose received to the animals was about the same in both cases: i.e. ~6.9 mGy delivered chronically over 4 weeks, with 60% of the dose from cosmic radiation instead of low-LET radiation used in the acute dose studies.

To conclude, we have identified in this work that NAFLD related pathways are activated in mice flown in space, independently of the strain or the duration of the mission. We suspect that activation of these pathways will persist during longer duration spaceflight missions, will be exacerbated by disrupted sleep resulting in gradual deposition of lipids in the liver and increased risk for nonalcoholic steatohepatitis (NASH) and irreversible liver fibrosis. More studies are needed to fully understand the mechanism of activation and persistence of this process to infer risks to astronauts.

## Materials and Method

### Animal and Sample Collection

The omics data for the mouse liver samples were obtained from NASA’s GeneLab public omics repository (https://genelab.nasa.gov/)^19–21^ from previous experiments performed by previous investigators from three spaceflight missions, Rodent Research-1 (RR-1) (with samples from both NASA and CASIS)^58,59^, and Rodent Research-3 (RR-3)^60^ and STS-135^61^. Since we did not perform these experiments and only obtained the data through GeneLab, no Institutional Animal Care and Use Committee (IACUC) was required. All previous animal experiments and methods were performed in accordance with the relevant guidelines at each institution and were approved by the institutions Institutional Animal Care and Use Committee (IACUC). We will provide details of the methodology for obtaining the tissues and processing which was done by previous investigators for the readers convenience, but all detailed information is also available on NASA’s GeneLab platform. The STS-135 mission was performed with the NASA AEM-X hardware^62^, while the RR-1 and RR-3 missions were performed with the new generation of the AEM-X referred to as Rodent Habitat^20^. The mice for each mission differed in the age and strain, as well as the mission duration. The metadata and experimental design pertaining to each mission was stored in NASA GeneLab database (https://genelab.nasa.gov/)^19–21^ as GLDS-168: RR-1 NASA^59^, GLDS-47: RR-1 CASIS^58^, GLDS-137: RR-3^60^ and GLDS-25 for STS-135^61,63^.

All animals were fed Nutrient Upgraded Rodent Food Bar (NuRFB)^63^. In all experiments, mice that were euthanized on orbit on the ISS were denoted as Flight samples, while mice of similar age, sex and strain housed for a similar number of days in identical hardware and matching ISS environmental conditions, were denoted as Ground control.

In the RR-1 mission, after 37 days of launch, five flight animals were euthanized using Euthasol injection followed by cervical dislocation and frozen as intact whole carcasses and stored in the Minus Eighty Degree Laboratory Freezer for ISS (MELFI) (−80 °C), except for two flight animals which were dissected on-board ISS before livers were isolated on-orbit and stored in the MELFI. Upon sample return on SpaceX-4, they were delivered to NASA Ames Research Center (ARC), RR-1 science team collected liver samples among other tissues from NASA frozen intact carcasses for flight and ground (n = 5). The carcasses were taken out from −80 °C freezer and thawed at room temperature for approximately 15 to 20 minutes, enough to allow the dissections to proceed. Liver was dissected out and was stored in liquid nitrogen after homogenization for all groups of animals except for two ground samples that were stored in mini cold bag and then in −80 °C freezer. For RR-1 CASIS mission, following 21 days of space travel, mice (n = 3 for each flight and ground control) were euthanized in a similar manner (like RR-1) either on orbit (flight samples) or on ground (ground controls) and the dissected livers were stored in mini cold bags in either MELFI (flight samples) or −80 °C freezer.

Mice from RR-3 mission (n = 6 for flight and ground), following 42 days of flight, were euthanized using Ketamine/xylazine injection followed by cervical dislocation. Liver was isolated from frozen carcasses and stored in liquid nitrogen following homogenization.

For STS-135, female C57BL/6J mice (n = 6) were placed into Animal Enclosure Modules (AEMs) and flown on the Space Shuttle Atlantis (STS-135) for 13 days. Ground AEM control mice (n = 6) were placed into the same hardware used in flight and environmental parameters such as temperature and CO_2_ levels were matched as closely as possible. Mice were anesthetized with 3–5% isoflurane and euthanized with 100% CO_2_ and exsanguination and dissected within 3–5 hours of landing.

### Histology and statistical analysis

Histology on samples from the three missions was performed by Triangulum Biopharma LLC (San Diego, CA) on archived tissues available from NASA Ames Life Sciences Data Archive (ALSDA). In brief, for each sample, one half was embedded in paraffin, while the second half was embedded in Optimal cutting temperature compound (OCT) for cryosectioning. Paraffin embedded samples were sectioned at 4 µm and stained with hematoxylin and eosin (H&E). OCT-embedded samples were cryosectioned at 5 µm and lipids stained with Oil Red O (ORO). Whole slide imaging was performed in bright field using a Panoramic Scan from 3D Histech and for each slide, marker positive (ORO staining) regions were identified using color deconvolution-based quantification. These identified regions were then quantified for precise positivity using the following metrics - ORO tissue positive area (in mm^2^) and percent of positively stained tissue area. In samples stained with Oil Red O lipid, Wilcoxon Rank Sum Test (Mann-Whitney U Test) was used to analyze differences in percent ORO positivity between flight and ground control samples. We observed high levels of ORO background staining for the RR-1 CASIS samples and we have adjusted accordingly for this during our quantification of the ORO stain.

### RNA isolation and purification

For all samples, homogenization buffer for RNA purification was made by adding 1:100 beta-mercaptoethanol to Buffer RLT, lysis buffer (Qiagen, Valenica, CA) and kept on ice. A piece of liver weighing between 20–30 mg was cut from each liver sample and immediately placed in a vial with 800 μL of the RLT solution. Each of these samples were then homogenized for approximately 20 seconds at 21,000 RPM using a Polytron PT1300D handheld homogenizer with a 5 mm standard dispersing aggregate (Kinematica, Bohemia, NY). Homogenates were centrifuged for 3 minutes at room temperature to remove tissue debris. The supernatant from each sample was then used to purify DNA and RNA via Qiagen AllPrep DNA/RNA Mini Kit (Qiagen, Valencia, CA). DNA was eluted in 50 μL of DNase- and RNase- free water per sample. RNA was eluted in 30 μL of DNase- and RNase- free water per sample. Concentration and absorbance ratios for all DNA and RNA samples were measured using the NanoDrop 2000 UV-Vis Spectrophotometer (Thermo Fisher Scientific, Waltham, MA). RNA quality was assessed using the Agilent 2100 Bioanalyzer (Agilent Technologies, Santa Clara, CA). A minimum of 1 µg of RNA having RIN values of 7 or above was sent out for RNA sequencing.

### Transcriptomics data analysis

Microarray transcription profiling data from STS-135 mouse liver samples, obtained from the GeneLab Dataset-25 (GLDS-25), was preprocessed in R using the Affy package. Robust multi-array average (RMA) quantile normalization was performed on the intensity values. Since the dataset contained three different biological groups (Flight samples, Habitat ground controls or AEM, and Vivarium ground controls), we imported the data into MultiExperiment Viewer^64^ and statistically significant genes was determined by ANOVA analysis between all conditions for GLDS-25 with a False Discovery Rate (FDR) ≤ 0.05. This resulted in 519 significant number genes (with FDR ≤ 0.05) for that were utilized for the remainder of the analysis.

RNA-Seq analysis was performed on NASA GLDS-168 and GLDS-47 datasets, which includes both RR1 and RR3 liver data. Specific details on RNA sequencing methods can be found on GeneLab’s platform which was described by the original investigator. Data validation and quality control was performed with FASTQC and Trim Galore! read alignment to the mouse genome using STAR RNA-seq aligner^65^ generation of gene-level expected count data with RSEM^65^. The list of software and its versions is as follow: FASTQC version 0.11.8, Trim Galore! Version 0.50, STAR version STAR_2.6.1a_08–27, mouse genome version mm10-GRCm38, RSEM version 1.3.1. The quality of the sequencing step was evaluated with FASTQC, Trim Galore was used to pre-process the expression data by trimming Illumina standard adapter sequences and nucleotides with a quality Phred score below 33.

For Differential Expression (DE) analysis, R Version 3.5.1 and DESeq. 2 Version 1.22.2 were used. Expected counts from the RSEM step were extracted and rounded up to the next integer and used as input for DE analysis. Significantly regulated genes were determined with a FDR ≤ 0.05 for GLDS-168 data using Benjamini-Hochberg multiple testing adjustment procedure and p-value ≤ 0.05 for GLDS-47. For the RR1 and RR3 data from the GLDS-168 there were 5874 genes and 588 significantly regulated genes respectively. For the GLDS-47 RR1: CASIS data there was 1125 significantly regulated genes.

For t-Distributed Stochastic Neighbor Embedding (t-SNE) plots^66^ we utilized R and performed t-SNE on all the genes and compared the separation between flight and habitat ground samples. Gene set enrichment analysis (GSEA) was done using the Reactome and C5: Gene Ontology (GO) gene sets with a FDR ≤ 0.05 from the entire list of genes and additional leading edge analysis was performed as described by Subramanian *et al*.^23^. All the datasets were compared with “Flight vs Ground Control” samples, the ranked list of genes were defined by the signal-to-noise metric, and the statistical significance were determined by 1000 permutations of the gene sets.

Pathway analysis and subsequent predictions in each tissue were done using the statistically significant genes with a fold-change ≥ 1.2 (or ≤ −1.2) comparing flight conditions versus habitat ground controls (with either p-value < 0.05 or FDR < 0.05 as stated above). Low fold-change has become quite standard when trying to identify genes that are differentially expressed^67–69^, as low cutoffs are less affected by different data normalization schemes and they are less likely to eliminate key genes operating under very tight level regulation. Ingenuity Pathway Analysis (IPA) software (Ingenuity Systems) was used to predict statistically significant activation or inhibition of upstream regulators using activation *Z*-score statistics ( ≥ 2, activated or ≤ −2, inhibited)^70^. Heat maps were generated using packages available through R (pheatmap for heat maps).

### Data processing and statistical analysis for proteomics data

The details for the proteomic data acquisition methods for the proteomic data is available on GeneLab which was utilized for this manuscript. Perseus (1.6.2.2) and in-house R scripts were used for proteomics data processing and statistical analysis^71^. The corrected reporter intensity values generated by MaxQuant were used to analyze the TMT-based proteomics data. Protein groups containing matches to decoy database or contaminants were discarded. Total ion intensity for each reporter ion channel were calculated and matched to correct for the sample loads in each TMT experiment. Only proteins that were quantified in the pooled samples were used for the analysis. Subsequently, internal reference scaling (IRS) method was employed to normalize protein intensities between different TMT runs using common proteins in pooled internal standards^72^. The data was log_2_ transformed and scaled by subtracting the median for each sample. Limma was employed to determine differentially protein abundance between groups and volcano plots were generated using EnhancedVolcano package (Bioconductor) to visualize the affected proteins. The affected proteins with p-value of less than 0.05 and log fold change of greater than 1.0 or less than −1.0 were considered significant. For RR3 data from the 2928 proteins 233 proteins were significantly expressed with a p-value ≤ 0.05. For RR1 data from the 2928 proteins 146 proteins were significantly expressed with a p-value ≤ 0.05. The categories with p-value ≤ 0.05 were considered as significant.

## Supplementary information

Supplemental Material
