# Author Correction: Multi-omics analysis of multiple missions to space reveal a theme of lipid dysregulation in mouse liver

**DOI:** 10.1038/s41598-020-58490-w

**Published:** 2020-01-27

**Authors:** Afshin Beheshti, Kaushik Chakravarty, Homer Fogle, Hossein Fazelinia, Willian A. da Silveira, Valery Boyko, San-Huei Lai Polo, Amanda M. Saravia-Butler, Gary Hardiman, Deanne Taylor, Jonathan M. Galazka, Sylvain V. Costes

**Affiliations:** 1 Wyle Labs, Space Biosciences Division, NASA Ames Research Center, Moffett Field, CA USA; 2 twoXAR Inc, Mountain View, CA USA; 3 Protein and Proteomics Core Facility, and the Department of Biomedical and Health Informatics, The Children’s Hospital of Philadelphia, Philadelphia, PA 19104 USA; 4 Institute for Global Food Security, Queens University Belfast, Belfast, UK; 5 Logyx LLC, Space Biosciences Division, NASA Ames Research Center, Moffett Field, CA USA; 6 Department of Biomedical and Health Informatics, The Children’s Hospital of Philadelphia, and the Department of Pediatrics, The University of Pennsylvania Perelman School of Medicine, Philadelphia, PA 19104 USA; 7 NASA Ames Research Center, Moffett Field, CA USA

Correction to: *Scientific Reports* 10.1038/s41598-019-55869-2, published online 16 December 2019

In the original version of this Article, Hossein Fazelinia was incorrectly affiliated with ‘Department of Biomedical and Health Informatics, The Children’s Hospital of Philadelphia, Philadelphia, USA; Center for Mitochondrial and Epigenomic Medicine, The Children’s Hospital of Philadelphia, Philadelphia, USA’. In addition, Deanne Taylor was incorrectly affiliated with ‘twoXAR Inc, Mountain View, CA, USA’. The correct affiliations are listed below.

Hossein Fazelinia:

Protein and Proteomics Core Facility, and the Department of Biomedical and Health Informatics, The Children’s Hospital of Philadelphia, Philadelphia, PA 19104, USA

Deanne Taylor:

Department of Biomedical and Health Informatics, The Children’s Hospital of Philadelphia, and the Department of Pediatrics, The University of Pennsylvania Perelman School of Medicine, Philadelphia, PA 19104, USA

These errors have now been corrected in the PDF and HTML versions of the Article.

