# Supplementary material for: Multi-omics analysis of multiple missions to space reveal a theme of lipid dysregulation in mouse liver
Source: Sci Rep. 2019 Dec 16;9:19195. doi: 10.1038/s41598-019-55869-2 (PMC6915713; doi:10.1038/s41598-019-55869-2)
Supplement: Supplementary file 1 — Supplemental Material [file 41598_2019_55869_MOESM1_ESM.pdf]

Supplemental Material

Multi-omics analysis of multiple missions to space reveal a theme of lipid dysregulation in mouse liver

Afshin Beheshti[1*], Kaushik Chakravarty[2*], Homer Fogle[1], Hossein Fazelinia[3], Willian A. da Silveira[4], Valery Boyko[1], San-Huei Lai Polo[1], Amanda M. Saravia-Butler[5], Gary Hardiman[4], Deanne Taylor[2], Jonathan M. Galazka[6], and Sylvain V. Costes[6]

*These authors are co-first authors

[1]Wyle Labs, Space Biosciences Division, NASA Ames Research Center, Moffett Field, CA
[2]twoXAR Inc. Mountain View, CA[3]Department of Biomedical and Health Informatics, The Children's Hospital of Philadelphia, Philadelphia, USA; Center for Mitochondrial and Epigenomic Medicine, The Children's Hospital of Philadelphia, USA.
[4]Institute for Global Food Security, Queens University Belfast, UK
[5]Logyx LLC, Space Biosciences Division, NASA Ames Research Center, Moffett Field, CA
[6]NASA Ames Research Center, Moffett Field, CA

Corresponding Authors:
Afshin Beheshti, and Sylvain Costes
afshin.beheshti@nasa.gov, and sylvain.v.costes@nasa.gov

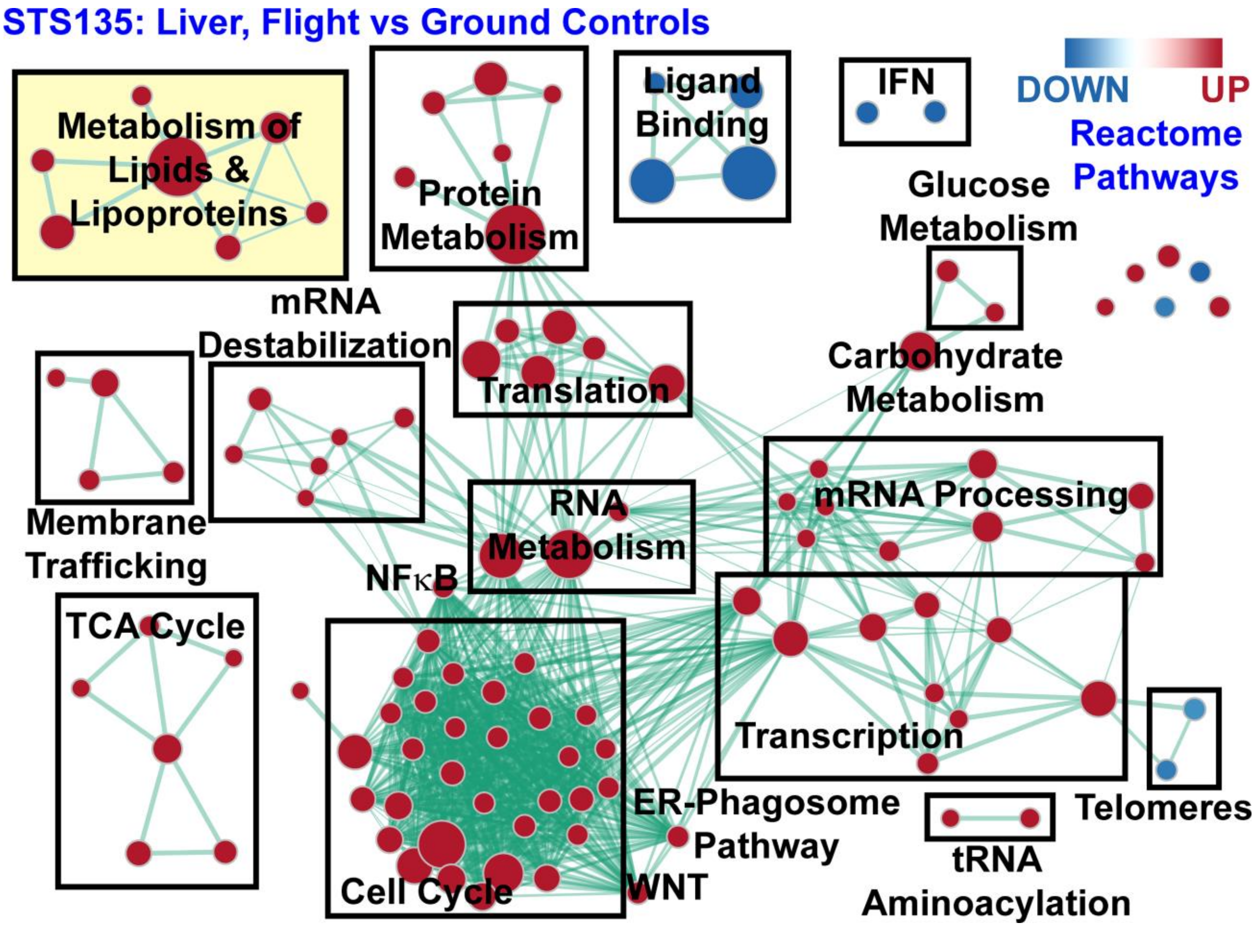

Supplemental Figure 1. Pathway analysis for livers from mice on the STS-135 mission. Gene Set Enrichment Analysis (GSEA) with Reactome pathways on liver's from STS-135 GeneLab dataset displayed as a network through a Cytoscape plugin, EnrichmentMap. Lipid related pathways are shown with a yellow background. Each node represents one gene set and the size of the node indicates the number of genes involved with the predictions. The color of the node indicates if the gene set is downregulated (blue) or upregulated (red). The shade of the color indicates degree of regulation. The thickness of the edge (green lines) represents the number of genes associated with the overlap of the gene sets (or nodes) that the edge connects. Clusters were named according to common function in each grouping.

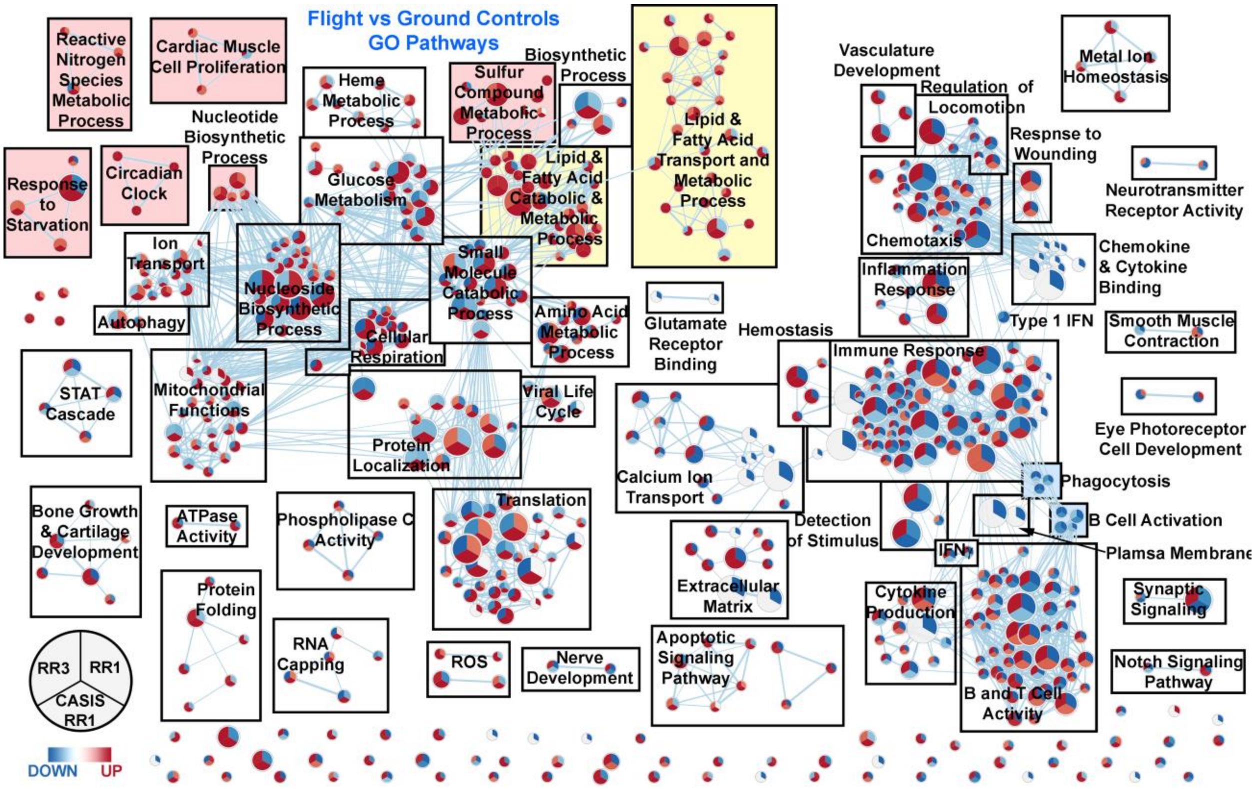

Supplemental Figure 2. Pathway analysis for livers from mice on the RR1 and RR3 mission. Gene Set Enrichment Analysis (GSEA) with Gene Ontology (GO) pathways on liver's from RR1 and RR3 GeneLab dataset displayed as a network through a Cytoscape plugin, EnrichmentMap. Red nodes indicate upregulation of the pathway and blue nodes represent downregulation. Lipid related pathways are shown with a yellow background. Light red background for nodes in network represents common pathways being upregulated between RR1 and RR3, while light blue background represents common pathways being downregulated between RR1 and RR3. Each node represents one gene set and the size of the node indicates the number of genes involved with the predictions. Each node contains 3 wedges for each condition and the color of each wedge indicates if the gene set is downregulated (blue) or upregulated (red). The shade of the color indicates degree of regulation. The thickness of the edge (blue lines) represents the number of genes associated with the overlap of the gene sets (or nodes) that the edge connects. Clusters were named according to common function in each grouping.

Supplemental Table 1. Gene Set Enrichment Analysis on the Gene Ontology (GO) terms for the STS-135 transcriptomic data set for Flight samples vs Habitat Ground Controls. Size = the number genes for the predicted GO term, and ES = Enrichment Score, NES = Normalized Enrichment Score.

NAME	SIZE	ES	NES	NOM p-val	FDR q-val
RIBOSOME BIOGENESIS	248	0.67	3.06	0.0000	0.0000
RIBONUCLEOPROTEIN COMPLEX BIOGENESIS	352	0.63	2.94	0.0000	0.0000
NCRNA PROCESSING	313	0.63	2.93	0.0000	0.0000
RRNA METABOLIC PROCESS	204	0.66	2.90	0.0000	0.0000
MITOCHONDRIAL MATRIX	352	0.60	2.80	0.0000	0.0000
NCRNA METABOLIC PROCESS	445	0.59	2.79	0.0000	0.0000
TRNA METABOLIC PROCESS	152	0.64	2.72	0.0000	0.0000
PRERIBOSOME	46	0.73	2.60	0.0000	0.0000
TRNA PROCESSING	96	0.65	2.58	0.0000	0.0000
COFACTOR BIOSYNTHETIC PROCESS	145	0.60	2.55	0.0000	0.0000
NUCLEOLAR PART	58	0.70	2.54	0.0000	0.0000
RNA MODIFICATION	94	0.63	2.53	0.0000	0.0000
TRANSLATIONAL TERMINATION	84	0.65	2.51	0.0000	0.0000
MITOCHONDRIAL TRANSLATION	96	0.64	2.48	0.0000	0.0000
ORGANELLAR RIBOSOME	65	0.66	2.47	0.0000	0.0000
COFACTOR METABOLIC PROCESS	289	0.54	2.46	0.0000	0.0000
ORGANELLE INNER MEMBRANE	451	0.52	2.45	0.0000	0.0000
AMIDE BIOSYNTHETIC PROCESS	431	0.52	2.45	0.0000	0.0000
MICROBODY	123	0.58	2.41	0.0000	0.0000
MITOCHONDRION ORGANIZATION	489	0.50	2.40	0.0000	0.0000
RIBONUCLEOPROTEIN COMPLEX SUBUNIT ORGANIZATION	162	0.56	2.38	0.0000	0.0000
RIBONUCLEOPROTEIN COMPLEX BINDING	88	0.61	2.38	0.0000	0.0000
RNA METHYLATION	37	0.72	2.37	0.0000	0.0000
MICROBODY PART	85	0.61	2.36	0.0000	0.0000
CELLULAR PROTEIN COMPLEX DISASSEMBLY	111	0.57	2.36	0.0000	0.0000
TRANSLATIONAL ELONGATION	100	0.59	2.36	0.0000	0.0000
LIPID OXIDATION	61	0.63	2.35	0.0000	0.0000
TRANSLATION INITIATION FACTOR ACTIVITY	46	0.67	2.35	0.0000	0.0000
ORGANELLAR LARGE RIBOSOMAL SUBUNIT	28	0.73	2.35	0.0000	0.0000
PEPTIDE METABOLIC PROCESS	483	0.49	2.34	0.0000	0.0000
MITOCHONDRIAL MEMBRANE PART	142	0.55	2.34	0.0000	0.0000

**RIBOSOMAL SMALL SUBUNIT BIOGENESIS**	41	0.68	2.34	0.0000	0.0000
**TRANSLATION FACTOR ACTIVITY RNA BINDING**	80	0.60	2.33	0.0000	0.0000
**RNA METHYLTRANSFERASE ACTIVITY**	30	0.74	2.33	0.0000	0.0000
**RNA SPLICING VIA TRANSESTERIFICATION REACTIONS**	216	0.53	2.33	0.0000	0.0000
**TRNA MODIFICATION**	49	0.66	2.33	0.0000	0.0000
**FATTY ACID CATABOLIC PROCESS**	60	0.63	2.32	0.0000	0.0000
**INTRINSIC COMPONENT OF MITOCHONDRIAL MEMBRANE**	41	0.67	2.32	0.0000	0.0000
**DNA TEMPLATED TRANSCRIPTION ELONGATION**	84	0.60	2.32	0.0000	0.0000
**MITOCHONDRIAL PROTEIN COMPLEX**	111	0.57	2.32	0.0000	0.0000
**COENZYME BIOSYNTHETIC PROCESS**	111	0.57	2.31	0.0000	0.0000
**COENZYME METABOLIC PROCESS**	235	0.52	2.31	0.0000	0.0000
**CATALYTIC STEP 2 SPLICEOSOME**	73	0.60	2.31	0.0000	0.0000
**MATURATION OF 5 8S RRNA**	25	0.74	2.29	0.0000	0.0000
**MITOCHONDRIAL RESPIRATORY CHAIN COMPLEX ASSEMBLY**	60	0.62	2.29	0.0000	0.0000
**VESICLE COATING**	65	0.60	2.28	0.0000	0.0000
**MEMBRANE BUDDING**	101	0.57	2.28	0.0000	0.0000
**RESPONSE TO TYPE I INTERFERON**	48	-0.67	-2.61	0.0000	0.0000
**LYMPHOCYTE MIGRATION**	35	-0.68	-2.41	0.0000	0.0000
**RIBOSOMAL LARGE SUBUNIT BIOGENESIS**	38	0.67	2.27	0.0000	0.0000
**PROTEIN LOCALIZATION TO MITOCHONDRION**	58	0.60	2.24	0.0000	0.0000
**FATTY ACID BETA OXIDATION**	43	0.65	2.24	0.0000	0.0000
**MATURATION OF SSU RRNA**	31	0.70	2.25	0.0000	0.0000
**REGULATION OF RECEPTOR BIOSYNTHETIC PROCESS**	20	0.78	2.25	0.0000	0.0000
**RNA SPLICING**	285	0.50	2.25	0.0000	0.0000
**SMALL SUBUNIT PROCESSOME**	26	0.74	2.26	0.0000	0.0000
**STRUCTURAL CONSTITUENT OF RIBOSOME**	178	0.52	2.26	0.0000	0.0000
**GOLGI ORGANIZATION**	78	0.59	2.27	0.0000	0.0000
**MRNA METABOLIC PROCESS**	494	0.47	2.27	0.0000	0.0000
**INNER MITOCHONDRIAL MEMBRANE PROTEIN COMPLEX**	84	0.57	2.23	0.0000	0.0001
**RIBOSOME**	184	0.51	2.23	0.0000	0.0001
**ASPARTATE FAMILY AMINO ACID METABOLIC PROCESS**	52	0.61	2.23	0.0000	0.0001

SNORNA BINDING	23	0.73	2.23	0.0000	0.0001
RIBOSOMAL SUBUNIT	131	0.53	2.22	0.0000	0.0001
MRNA PROCESSING	343	0.48	2.22	0.0000	0.0001
CELLULAR RESPIRATION	126	0.53	2.22	0.0000	0.0001
ER TO GOLGI VESICLE MEDIATED TRANSPORT	144	0.52	2.22	0.0000	0.0001
LARGE RIBOSOMAL SUBUNIT	77	0.58	2.20	0.0000	0.0001
SINGLE ORGANISM MEMBRANE BUDDING	64	0.59	2.21	0.0000	0.0001
RNA 3 END PROCESSING	76	0.57	2.21	0.0000	0.0001
MULTIVESICULAR BODY ORGANIZATION	27	0.70	2.21	0.0000	0.0001
RIBONUCLEOPROTEIN COMPLEX LOCALIZATION	92	0.56	2.19	0.0000	0.0001
AMINO ACID ACTIVATION	46	0.62	2.19	0.0000	0.0001
EXOSOME RNASE COMPLEX	18	0.79	2.20	0.0000	0.0001
RNA LOCALIZATION	144	0.52	2.20	0.0000	0.0001
TRANSCRIPTION ELONGATION FROM RNA POLYMERASE II PROMOTER	70	0.59	2.20	0.0000	0.0001
MACROMOLECULAR COMPLEX DISASSEMBLY	166	0.51	2.20	0.0000	0.0001
90S PRERIBOSOME	18	0.78	2.18	0.0000	0.0002
TRNA AMINOACYLATION	46	0.62	2.18	0.0000	0.0002
INTRA GOLGI VESICLE MEDIATED TRANSPORT	33	0.67	2.19	0.0000	0.0002
UBIQUITIN LIGASE COMPLEX	235	0.48	2.17	0.0000	0.0002
SPLICEOSOMAL COMPLEX	137	0.53	2.17	0.0000	0.0002
TRANSLATIONAL INITIATION	122	0.52	2.15	0.0000	0.0002
TRNA BINDING	38	0.63	2.15	0.0000	0.0002
PEROXISOME ORGANIZATION	28	0.67	2.15	0.0000	0.0002
THIOESTER METABOLIC PROCESS	76	0.55	2.15	0.0000	0.0002
VERY LONG CHAIN FATTY ACID METABOLIC PROCESS	24	0.71	2.17	0.0000	0.0002
S ADENOSYLMETHIONINE DEPENDENT METHYLTRANSFERASE ACTIVITY	105	0.53	2.15	0.0000	0.0002
AEROBIC RESPIRATION	47	0.61	2.15	0.0000	0.0002
CARBOXYLIC ACID CATABOLIC PROCESS	178	0.50	2.15	0.0000	0.0002
RIBOSOME ASSEMBLY	46	0.62	2.17	0.0000	0.0002
ORGANIC ACID CATABOLIC PROCESS	178	0.50	2.15	0.0000	0.0002
MICROBODY MEMBRANE	54	0.60	2.16	0.0000	0.0002
RNA PHOSPHODIESTER BOND HYDROLYSIS	97	0.54	2.16	0.0000	0.0002
LIPID PARTICLE	53	0.60	2.14	0.0000	0.0002

**INTRINSIC COMPONENT OF MITOCHONDRIAL OUTER MEMBRANE**	19	0.75	2.14	0.0000	0.0002
**MITOCHONDRIAL RESPIRATORY CHAIN COMPLEX I ASSEMBLY**	46	0.60	2.13	0.0000	0.0003
**RNA HELICASE ACTIVITY**	59	0.58	2.13	0.0000	0.0003
**MATURATION OF SSU RRNA FROM TRICISTRONIC RRNA TRANSCRIPT SSU RRNA 5 8S RRNA LSU RRNA**	24	0.70	2.13	0.0000	0.0003
**ENERGY DERIVATION BY OXIDATION OF ORGANIC COMPOUNDS**	187	0.49	2.12	0.0000	0.0004
**PRENYLTRANSFERASE ACTIVITY**	15	0.79	2.12	0.0000	0.0004
**VESICLE TARGETING**	68	0.57	2.12	0.0000	0.0004
**PROTEIN TRANSPORTER ACTIVITY**	95	0.53	2.13	0.0000	0.0004
**TRANSFERASE ACTIVITY TRANSFERRING ONE CARBON GROUPS**	164	0.49	2.12	0.0000	0.0004
**GOLGI VESICLE TRANSPORT**	269	0.47	2.12	0.0000	0.0004
**COA HYDROLASE ACTIVITY**	19	0.74	2.12	0.0000	0.0004
**PROTEIN TARGETING TO MITOCHONDRION**	42	0.62	2.12	0.0000	0.0004
**ACYL COA METABOLIC PROCESS**	76	0.55	2.12	0.0000	0.0004
**TRNA METHYLTRANSFERASE ACTIVITY**	15	0.77	2.11	0.0000	0.0004
**NADH DEHYDROGENASE COMPLEX ASSEMBLY**	46	0.60	2.11	0.0000	0.0004
**CYTOPLASMIC TRANSLATION**	34	0.63	2.11	0.0000	0.0004
**TRNA METHYLATION**	18	0.74	2.10	0.0000	0.0006
**PROTEIN POLYUBIQUITINATION**	219	0.47	2.09	0.0000	0.0006
**PRERIBOSOME LARGE SUBUNIT PRECURSOR**	16	0.77	2.09	0.0000	0.0007
**TRANSFERASE ACTIVITY TRANSFERRING ACYL GROUPS OTHER THAN AMINO ACYL GROUPS**	172	0.48	2.09	0.0000	0.0007
**CULLIN RING UBIQUITIN LIGASE COMPLEX**	132	0.50	2.09	0.0000	0.0007
**MONOCARBOXYLIC ACID CATABOLIC PROCESS**	79	0.54	2.09	0.0000	0.0007
**AUTOPHAGOSOME ORGANIZATION**	29	0.65	2.09	0.0000	0.0007
**NUCLEAR EXPORT**	117	0.51	2.08	0.0000	0.0008
**TRNA TRANSPORT**	29	0.66	2.08	0.0000	0.0008
**MICROBODY LUMEN**	41	0.61	2.08	0.0000	0.0008
**ESCRT COMPLEX**	23	0.69	2.08	0.0000	0.0008
**REGULATION OF TRANSLATIONAL ELONGATION**	20	0.70	2.08	0.0000	0.0009

**DNA TEMPLATED TRANSCRIPTION TERMINATION**	81	0.54	2.08	0.0000	0.0009
**CIS GOLGI NETWORK**	39	0.62	2.08	0.0000	0.0009
**ELECTRON TRANSPORT CHAIN**	82	0.53	2.07	0.0000	0.0011
**ANAPHASE PROMOTING COMPLEX DEPENDENT CATABOLIC PROCESS**	69	0.54	2.06	0.0000	0.0011
**MULTI ORGANISM LOCALIZATION**	54	0.56	2.06	0.0000	0.0012
**PROTEIN TRANSMEMBRANE TRANSPORT**	44	0.60	2.06	0.0000	0.0012
**REGULATION OF PROTEIN UBIQUITINATION INVOLVED IN UBIQUITIN DEPENDENT PROTEIN CATABOLIC PROCESS**	91	0.51	2.06	0.0000	0.0012
**MITOCHONDRIAL TRANSPORT**	142	0.49	2.06	0.0000	0.0012
**MULTI ORGANISM TRANSPORT**	54	0.56	2.06	0.0000	0.0012
**MITOCHONDRIAL RESPIRATORY CHAIN COMPLEX I BIOGENESIS**	46	0.60	2.06	0.0000	0.0012
**PORPHYRIN CONTAINING COMPOUND METABOLIC PROCESS**	31	0.63	2.05	0.0000	0.0012
**RRNA BINDING**	50	0.58	2.06	0.0000	0.0012
**TETRAPYRROLE BIOSYNTHETIC PROCESS**	25	0.67	2.05	0.0017	0.0013
**PROTEASOMAL PROTEIN CATABOLIC PROCESS**	240	0.46	2.05	0.0000	0.0013
**MRNA 3 END PROCESSING**	56	0.55	2.05	0.0000	0.0013
**REGULATION OF MITOPHAGY**	36	0.62	2.04	0.0000	0.0015
**GLYOXYLATE METABOLIC PROCESS**	26	0.65	2.04	0.0000	0.0015
**TRANSFERASE ACTIVITY TRANSFERRING ACYL GROUPS**	201	0.46	2.04	0.0000	0.0016
**EXORIBONUCLEASE ACTIVITY**	31	0.65	2.03	0.0000	0.0016
**MATURATION OF 5 8S RRNA FROM TRICISTRONIC RRNA TRANSCRIPT SSU RRNA 5 8S RRNA LSU RRNA**	17	0.73	2.03	0.0000	0.0017
**PURINE NTP DEPENDENT HELICASE ACTIVITY**	84	0.52	2.03	0.0000	0.0017
**ESTABLISHMENT OF PROTEIN LOCALIZATION TO ORGANELLE**	297	0.44	2.02	0.0000	0.0019
**OXIDATIVE PHOSPHORYLATION**	71	0.53	2.02	0.0000	0.0019
**VACUOLAR MEMBRANE**	487	0.42	2.02	0.0000	0.0021
**QUINONE METABOLIC PROCESS**	19	0.71	2.02	0.0000	0.0022
**LIGASE ACTIVITY FORMING CARBON OXYGEN BONDS**	39	0.60	2.01	0.0000	0.0023
**VACUOLAR TRANSPORT**	205	0.46	2.01	0.0000	0.0023
**RNA CATABOLIC PROCESS**	195	0.45	2.01	0.0000	0.0023

**ENDORIBONUCLEASE ACTIVITY PRODUCING 5 PHOSPHOMONOESTERS**	22	0.67	2.01	0.0000	0.0024
**CELLULAR IRON ION HOMEOSTASIS**	38	0.60	2.01	0.0000	0.0024
**MRNA CLEAVAGE FACTOR COMPLEX**	16	0.72	2.00	0.0017	0.0024
**TRANS GOLGI NETWORK MEMBRANE**	58	0.54	2.01	0.0000	0.0024
**PROTEIN LOCALIZATION TO ORGANELLE**	473	0.42	2.01	0.0000	0.0024
**TRANSCRIPTION FROM RNA POLYMERASE I PROMOTER**	33	0.61	2.01	0.0000	0.0024
**EXONUCLEASE ACTIVITY ACTIVE WITH EITHER RIBO OR DEOXYRIBONUCLEIC ACIDS AND PRODUCING 5 PHOSPHOMONOESTERS**	39	0.59	2.01	0.0017	0.0024
**PROTEIN CATABOLIC PROCESS**	494	0.42	2.01	0.0000	0.0024
**THIOLESTER HYDROLASE ACTIVITY**	31	0.62	2.00	0.0000	0.0026
**LATE ENDOSOME MEMBRANE**	80	0.51	2.00	0.0000	0.0026
**OUTER MEMBRANE**	169	0.46	2.00	0.0000	0.0027
**SMALL MOLECULE CATABOLIC PROCESS**	285	0.44	2.00	0.0000	0.0027
**COATED MEMBRANE**	78	0.52	1.99	0.0000	0.0027
**PROTEASOME ACCESSORY COMPLEX**	24	0.66	2.00	0.0017	0.0027
**NUCLEAR TRANSCRIBED MRNA CATABOLIC PROCESS DEADENYLATION DEPENDENT DECAY**	53	0.57	1.99	0.0000	0.0029
**BRANCHED CHAIN AMINO ACID METABOLIC PROCESS**	23	0.67	1.99	0.0000	0.0030
**LIGASE ACTIVITY**	357	0.43	1.98	0.0000	0.0030
**COPI COATED VESICLE MEMBRANE**	16	0.71	1.98	0.0000	0.0031
**VESICLE COAT**	42	0.59	1.98	0.0000	0.0031
**REGULATION OF MITOCHONDRIAL FISSION**	18	0.71	1.98	0.0017	0.0031
**CARBOXYLIC ESTER HYDROLASE ACTIVITY**	111	0.48	1.98	0.0000	0.0031
**THIOESTER BIOSYNTHETIC PROCESS**	48	0.56	1.98	0.0000	0.0032
**NUCLEOBASE CONTAINING COMPOUND TRANSPORT**	157	0.46	1.97	0.0000	0.0036
**INTRINSIC COMPONENT OF ORGANELLE MEMBRANE**	227	0.44	1.96	0.0000	0.0038
**NUCLEAR TRANSCRIBED MRNA CATABOLIC PROCESS NONSENSE MEDIATED DECAY**	98	0.48	1.97	0.0000	0.0038
**AMINO ACID BETAINE METABOLIC PROCESS**	16	0.72	1.96	0.0018	0.0039
**PROTEIN K11 LINKED UBIQUITINATION**	23	0.65	1.96	0.0000	0.0039
**NEGATIVE REGULATION OF HEART CONTRACTION**	20	0.67	1.96	0.0018	0.0041

**CELLULAR CARBOHYDRATE CATABOLIC PROCESS**	26	0.63	1.96	0.0000	0.0043
**GLYCOSYL COMPOUND METABOLIC PROCESS**	306	0.42	1.96	0.0000	0.0044
**ACYL COA BIOSYNTHETIC PROCESS**	48	0.56	1.96	0.0000	0.0044
**RRNA MODIFICATION**	18	0.70	1.96	0.0000	0.0044
**CLEAVAGE INVOLVED IN RRNA PROCESSING**	16	0.72	1.95	0.0017	0.0044
**MULTI ORGANISM METABOLIC PROCESS**	113	0.48	1.95	0.0000	0.0044
**GLYCOSYL COMPOUND BIOSYNTHETIC PROCESS**	98	0.49	1.95	0.0000	0.0045
**GPI ANCHOR METABOLIC PROCESS**	28	0.63	1.95	0.0016	0.0045
**HELICASE ACTIVITY**	135	0.46	1.95	0.0000	0.0046
**RNA POLYMERASE COMPLEX**	105	0.49	1.95	0.0000	0.0046
**RNA PHOSPHODIESTER BOND HYDROLYSIS EXONUCLEOLYTIC**	32	0.61	1.95	0.0000	0.0046
**TRANS GOLGI NETWORK**	156	0.46	1.95	0.0000	0.0046
**GENERATION OF PRECURSOR METABOLITES AND ENERGY**	255	0.43	1.95	0.0000	0.0046
**POST GOLGI VESICLE MEDIATED TRANSPORT**	71	0.51	1.95	0.0000	0.0047
**RNA PHOSPHODIESTER BOND HYDROLYSIS ENDONUCLEOLYTIC**	46	0.55	1.95	0.0000	0.0047
**CELLULAR AMINO ACID METABOLIC PROCESS**	298	0.42	1.94	0.0000	0.0048
**MITOCHONDRIAL FUSION**	17	0.68	1.94	0.0000	0.0049
**POSTTRANSCRIPTIONAL REGULATION OF GENE EXPRESSION**	404	0.41	1.94	0.0000	0.0049
**RNA POLYMERASE II REPRESSING TRANSCRIPTION FACTOR BINDING**	25	0.63	1.94	0.0000	0.0049
**NUCLEAR PORE**	66	0.51	1.94	0.0000	0.0052
**FORMATION OF TRANSLATION PREINITIATION COMPLEX**	17	0.70	1.94	0.0000	0.0052
**UBIQUITIN LIKE PROTEIN TRANSFERASE ACTIVITY**	367	0.41	1.93	0.0000	0.0053
**NUCLEAR TRANSCRIBED MRNA CATABOLIC PROCESS EXONUCLEOLYTIC**	29	0.62	1.93	0.0000	0.0054
**SMALL NUCLEOLAR RIBONUCLEOPROTEIN COMPLEX**	17	0.70	1.93	0.0000	0.0054
**RIBOSOMAL LARGE SUBUNIT ASSEMBLY**	22	0.65	1.93	0.0000	0.0055
**CELLULAR COMPONENT DISASSEMBLY**	449	0.41	1.93	0.0000	0.0055
**AUTOPHAGY**	312	0.42	1.93	0.0000	0.0055

**NUCLEIC ACID PHOSPHODIESTER BOND HYDROLYSIS**	207	0.43	1.93	0.0000	0.0056
**RESPIRATORY CHAIN**	69	0.51	1.92	0.0000	0.0061
**HEPARAN SULFATE PROTEOGLYCAN METABOLIC PROCESS**	28	0.62	1.92	0.0000	0.0061
**ENDOSOMAL PART**	350	0.41	1.92	0.0000	0.0061
**SULFUR COMPOUND METABOLIC PROCESS**	315	0.41	1.92	0.0000	0.0062
**FATTY ACID METABOLIC PROCESS**	237	0.43	1.92	0.0000	0.0062
**CAJAL BODY**	44	0.55	1.92	0.0016	0.0062
**PURINE NUCLEOSIDE BIOSYNTHETIC PROCESS**	72	0.50	1.92	0.0000	0.0062
**UBIQUITIN LIKE PROTEIN LIGASE BINDING**	232	0.43	1.92	0.0000	0.0062
**MEMBRANE DISASSEMBLY**	40	0.56	1.92	0.0000	0.0062
**PURINE RIBONUCLEOSIDE BIOSYNTHETIC PROCESS**	72	0.50	1.92	0.0000	0.0062
**INTRINSIC COMPONENT OF MITOCHONDRIAL INNER MEMBRANE**	16	0.71	1.92	0.0035	0.0063
**PHOSPHATIDIC ACID METABOLIC PROCESS**	31	0.59	1.92	0.0000	0.0063
**RIBONUCLEASE ACTIVITY**	79	0.50	1.92	0.0000	0.0063
**ORGANELLE SUBCOMPARTMENT**	254	0.42	1.91	0.0000	0.0064
**CUL4 RING E3 UBIQUITIN LIGASE COMPLEX**	21	0.66	1.92	0.0000	0.0064
**EUKARYOTIC TRANSLATION INITIATION FACTOR 3 COMPLEX**	15	0.71	1.91	0.0000	0.0064
**PROTEIN K63 LINKED UBIQUITINATION**	30	0.59	1.91	0.0017	0.0065
**ASPARTATE FAMILY AMINO ACID CATABOLIC PROCESS**	17	0.69	1.91	0.0053	0.0065
**HEME METABOLIC PROCESS**	25	0.61	1.91	0.0000	0.0067
**REGULATION OF LIGASE ACTIVITY**	113	0.47	1.91	0.0000	0.0067
**LIPID CATABOLIC PROCESS**	211	0.43	1.91	0.0000	0.0068
**LIPOPROTEIN BIOSYNTHETIC PROCESS**	76	0.49	1.90	0.0000	0.0071
**SNRNA METABOLIC PROCESS**	74	0.50	1.90	0.0000	0.0071
**VACUOLE ORGANIZATION**	132	0.46	1.90	0.0000	0.0071
**OXIDOREDUCTASE ACTIVITY ACTING ON THE CH CH GROUP OF DONORS**	50	0.53	1.90	0.0000	0.0072
**NUCLEOTIDE EXCISION REPAIR PREINCISION COMPLEX ASSEMBLY**	24	0.62	1.90	0.0017	0.0072
**NUCLEAR ENVELOPE DISASSEMBLY**	40	0.56	1.90	0.0000	0.0073
**SUGAR TRANSMEMBRANE TRANSPORTER ACTIVITY**	26	0.61	1.90	0.0050	0.0073

**CHEMOKINE ACTIVITY**	30	-0.65	-2.20	0.0000	0.0073
**MULTI ORGANISM MEMBRANE BUDDING**	21	0.64	1.90	0.0000	0.0074
**SNARE COMPLEX**	48	0.55	1.90	0.0000	0.0074
**COPI COATED VESICLE**	22	0.64	1.90	0.0017	0.0074
**ENDOPLASMIC RETICULUM ORGANIZATION**	32	0.58	1.90	0.0017	0.0075
**GLYCOSYL COMPOUND CATABOLIC PROCESS**	32	0.59	1.90	0.0000	0.0075
**POSITIVE REGULATION OF MITOCHONDRION ORGANIZATION**	143	0.45	1.90	0.0000	0.0075
**LYMPHOCYTE CHEMOTAXIS**	24	-0.67	-2.21	0.0000	0.0076
**ASPARTATE FAMILY AMINO ACID BIOSYNTHETIC PROCESS**	23	0.63	1.90	0.0000	0.0076
**PHOSPHATASE COMPLEX**	41	0.55	1.89	0.0000	0.0077
**OLIGOSACCHARIDE LIPID INTERMEDIATE BIOSYNTHETIC PROCESS**	18	0.67	1.89	0.0018	0.0078
**METHYLATION**	217	0.43	1.89	0.0000	0.0078
**REGULATION OF LIPID CATABOLIC PROCESS**	47	0.54	1.89	0.0000	0.0080
**NCRNA CATABOLIC PROCESS**	18	0.68	1.89	0.0000	0.0081
**DOLICHOL LINKED OLIGOSACCHARIDE BIOSYNTHETIC PROCESS**	18	0.67	1.89	0.0000	0.0081
**REGULATION OF RESPONSE TO NUTRIENT LEVELS**	156	0.44	1.89	0.0000	0.0082
**RNA SECONDARY STRUCTURE UNWINDING**	39	0.55	1.89	0.0000	0.0082
**TRANSFERASE COMPLEX TRANSFERRING PHOSPHORUS CONTAINING GROUPS**	209	0.43	1.89	0.0000	0.0082
**PROTEIN SERINE THREONINE PHOSPHATASE ACTIVITY**	57	0.53	1.89	0.0000	0.0083
**FATTY ACYL COA METABOLIC PROCESS**	45	0.54	1.88	0.0017	0.0083
**IRON ION HOMEOSTASIS**	60	0.52	1.88	0.0000	0.0089
**COATED VESICLE**	195	0.43	1.88	0.0000	0.0089
**ENDONUCLEASE ACTIVITY ACTIVE WITH EITHER RIBO OR DEOXYRIBONUCLEIC ACIDS AND PRODUCING 5 PHOSPHOMONOESTERS**	25	0.61	1.88	0.0017	0.0089
**NUCLEAR ENVELOPE ORGANIZATION**	69	0.50	1.88	0.0000	0.0089
**MITOCHONDRIAL ELECTRON TRANSPORT NADH TO UBIQUINONE**	37	0.55	1.88	0.0016	0.0089
**PROTEIN N TERMINUS BINDING**	90	0.48	1.88	0.0000	0.0089

**OXIDOREDUCTION COENZYME METABOLIC PROCESS**	94	0.47	1.88	0.0000	0.0089
**REGULATION OF RESPONSE TO EXTRACELLULAR STIMULUS**	156	0.44	1.88	0.0000	0.0089
**PEROXISOMAL TRANSPORT**	16	0.68	1.87	0.0000	0.0094
**BETA TUBULIN BINDING**	33	0.59	1.87	0.0000	0.0095
**ORGANELLAR SMALL RIBOSOMAL SUBUNIT**	24	0.63	1.87	0.0017	0.0096
**REGULATION OF MITOCHONDRION ORGANIZATION**	186	0.43	1.87	0.0000	0.0097
**NUCLEOTIDE EXCISION REPAIR DNA DAMAGE RECOGNITION**	19	0.65	1.87	0.0000	0.0098
**GOLGI ASSOCIATED VESICLE**	71	0.49	1.87	0.0000	0.0099
**VIRAL BUDDING**	21	0.64	1.86	0.0000	0.0100
**SIGNAL SEQUENCE BINDING**	34	0.58	1.86	0.0000	0.0104
**PROTEIN LOCALIZATION TO VACUOLE**	37	0.55	1.86	0.0000	0.0105
**COATED VESICLE MEMBRANE**	109	0.46	1.86	0.0000	0.0106
**MITOCHONDRIAL TRANSMEMBRANE TRANSPORT**	47	0.53	1.86	0.0000	0.0106
**MITOCHONDRIAL RNA METABOLIC PROCESS**	21	0.63	1.86	0.0018	0.0106
**PROTEIN IMPORT**	137	0.44	1.86	0.0000	0.0106
**OXIDOREDUCTASE ACTIVITY ACTING ON NAD P H QUINONE OR SIMILAR COMPOUND AS ACCEPTOR**	42	0.54	1.86	0.0016	0.0108
**REGULATION OF LIPID STORAGE**	38	0.55	1.86	0.0016	0.0108
**BROWN FAT CELL DIFFERENTIATION**	28	0.60	1.85	0.0052	0.0109
**COENZYME BINDING**	165	0.43	1.85	0.0000	0.0111
**MONOSACCHARIDE TRANSMEMBRANE TRANSPORTER ACTIVITY**	16	0.68	1.85	0.0000	0.0111
**NF KAPPAB BINDING**	26	0.61	1.85	0.0033	0.0111
**EXONUCLEASE ACTIVITY**	66	0.50	1.85	0.0015	0.0111
**OXIDOREDUCTASE ACTIVITY ACTING ON NAD P H**	78	0.48	1.85	0.0000	0.0113
**NCRNA TRANSCRIPTION**	79	0.47	1.85	0.0000	0.0113
**REGULATION OF TOR SIGNALING**	61	0.50	1.85	0.0000	0.0114
**RETROGRADE VESICLE MEDIATED TRANSPORT GOLGI TO ER**	68	0.50	1.85	0.0000	0.0114
**REGULATION OF PLASMA LIPOPROTEIN PARTICLE LEVELS**	41	0.55	1.85	0.0017	0.0116
**NUCLEOID**	36	0.57	1.85	0.0017	0.0116
**ORGANELLE DISASSEMBLY**	153	0.43	1.84	0.0000	0.0116

**CELLULAR CARBOHYDRATE METABOLIC PROCESS**	117	0.44	1.85	0.0000	0.0116
**KETONE BIOSYNTHETIC PROCESS**	21	0.63	1.85	0.0034	0.0116
**MONOSACCHARIDE BIOSYNTHETIC PROCESS**	46	0.53	1.84	0.0017	0.0116
**NUCLEOSIDE BISPHOSPHATE METABOLIC PROCESS**	32	0.57	1.85	0.0032	0.0116
**CARBOHYDRATE BIOSYNTHETIC PROCESS**	101	0.46	1.84	0.0015	0.0118
**MULTI ORGANISM ORGANELLE ORGANIZATION**	21	0.64	1.84	0.0017	0.0118
**UBIQUITIN LIKE PROTEIN CONJUGATING ENZYME BINDING**	33	0.57	1.84	0.0016	0.0118
**ACETYLTRANSFERASE ACTIVITY**	82	0.47	1.84	0.0000	0.0119
**MEMBRANE BIOGENESIS**	28	0.58	1.84	0.0017	0.0119
**RIBOSOME BINDING**	42	0.54	1.84	0.0000	0.0119
**HEME BIOSYNTHETIC PROCESS**	19	0.63	1.84	0.0000	0.0119
**ORGANOPHOSPHATE BIOSYNTHETIC PROCESS**	398	0.39	1.84	0.0000	0.0121
**METAL CLUSTER BINDING**	57	0.51	1.84	0.0000	0.0122
**ALDITOL PHOSPHATE METABOLIC PROCESS**	33	0.56	1.84	0.0016	0.0122
**NIK NF KAPPAB SIGNALING**	74	0.49	1.84	0.0000	0.0122
**REGULATION OF TRIGLYCERIDE METABOLIC PROCESS**	31	0.57	1.83	0.0017	0.0124
**RIBONUCLEOSIDE BISPHOSPHATE METABOLIC PROCESS**	32	0.57	1.83	0.0017	0.0124
**POSITIVE REGULATION OF DENDRITE MORPHOGENESIS**	32	0.57	1.83	0.0017	0.0125
**PROTEIN MODIFICATION BY SMALL PROTEIN REMOVAL**	101	0.45	1.83	0.0000	0.0126
**PURINE NUCLEOSIDE BISPHOSPHATE METABOLIC PROCESS**	32	0.57	1.83	0.0000	0.0128
**TRANSLATION PREINITIATION COMPLEX**	15	0.69	1.83	0.0000	0.0128
**NUCLEAR SPECK**	163	0.42	1.83	0.0000	0.0128
**CELLULAR PROTEIN COMPLEX ASSEMBLY**	283	0.40	1.83	0.0000	0.0128
**DNA DIRECTED RNA POLYMERASE II HOLOENZYME**	80	0.47	1.83	0.0000	0.0128
**RELAXATION OF MUSCLE**	20	0.64	1.83	0.0000	0.0130
**RIBONUCLEOSIDE TRIPHOSPHATE BIOSYNTHETIC PROCESS**	40	0.53	1.83	0.0034	0.0131
**POSITIVE REGULATION OF LIGASE ACTIVITY**	93	0.45	1.83	0.0000	0.0133

**NEGATIVE REGULATION OF PROTEIN MODIFICATION BY SMALL PROTEIN CONJUGATION OR REMOVAL**	120	0.44	1.83	0.0000	0.0133
**NCRNA 3 END PROCESSING**	16	0.67	1.82	0.0035	0.0134
**POSITIVE REGULATION OF LIPID STORAGE**	18	0.65	1.82	0.0017	0.0134
**NUCLEAR ENVELOPE REASSEMBLY**	17	0.67	1.82	0.0035	0.0134
**PROTEIN TARGETING**	340	0.39	1.82	0.0000	0.0134
**ISOMERASE ACTIVITY**	132	0.44	1.82	0.0000	0.0134
**GDP BINDING**	48	0.52	1.82	0.0000	0.0134
**RESPONSE TO X RAY**	24	0.60	1.82	0.0035	0.0134
**CELLULAR LIPID CATABOLIC PROCESS**	132	0.44	1.82	0.0000	0.0138
**PURINE RIBONUCLEOSIDE BISPHOSPHATE METABOLIC PROCESS**	15	0.67	1.82	0.0034	0.0139
**VIRION ASSEMBLY**	30	0.58	1.82	0.0017	0.0139
**TRANSFERASE ACTIVITY TRANSFERRING ALKYL OR ARYL OTHER THAN METHYL GROUPS**	55	0.50	1.82	0.0015	0.0140
**NUCLEAR TRANSCRIBED MRNA POLY A TAIL SHORTENING**	23	0.61	1.82	0.0000	0.0140
**NUCLEOBASE CONTAINING SMALL MOLECULE METABOLIC PROCESS**	457	0.38	1.82	0.0000	0.0140
**BLASTOCYST DEVELOPMENT**	56	0.50	1.81	0.0000	0.0143
**BASAL TRANSCRIPTION MACHINERY BINDING**	21	0.63	1.82	0.0017	0.0143
**PEPTIDYL LYSINE MODIFICATION**	262	0.40	1.82	0.0000	0.0143
**FC EPSILON RECEPTOR SIGNALING PATHWAY**	116	0.44	1.81	0.0000	0.0144
**LIGASE ACTIVITY FORMING CARBON NITROGEN BONDS**	49	0.52	1.81	0.0000	0.0144
**REGULATION OF CELLULAR PROTEIN CATABOLIC PROCESS**	246	0.40	1.81	0.0000	0.0145
**OXIDOREDUCTASE ACTIVITY ACTING ON A SULFUR GROUP OF DONORS**	46	0.52	1.81	0.0000	0.0145
**POSITIVE REGULATION OF DNA TEMPLATED TRANSCRIPTION ELONGATION**	19	0.62	1.81	0.0069	0.0146
**NUCLEOTIDE EXCISION REPAIR DNA INCISION 3 TO LESION**	17	0.64	1.81	0.0153	0.0151
**MULTI ORGANISM MEMBRANE ORGANIZATION**	27	0.58	1.81	0.0051	0.0151
**PROTON TRANSPORTING TWO SECTOR ATPASE COMPLEX**	39	0.54	1.81	0.0000	0.0152

**RETROGRADE TRANSPORT ENDOSOME TO GOLGI**	57	0.50	1.81	0.0016	0.0154
**UBIQUITIN LIKE PROTEIN SPECIFIC PROTEASE ACTIVITY**	89	0.46	1.80	0.0015	0.0158
**TETRAPYRROLE METABOLIC PROCESS**	48	0.51	1.80	0.0000	0.0159
**SNAP RECEPTOR ACTIVITY**	33	0.55	1.80	0.0016	0.0161
**POLYSOME**	37	0.54	1.80	0.0000	0.0166
**COFACTOR BINDING**	242	0.40	1.80	0.0000	0.0166
**LYTIC VACUOLE MEMBRANE**	223	0.40	1.80	0.0000	0.0166
**NUCLEAR BODY**	290	0.39	1.80	0.0000	0.0166
**TRANSCRIPTION COUPLED NUCLEOTIDE EXCISION REPAIR**	63	0.48	1.80	0.0000	0.0166
**ACETYLTRANSFERASE COMPLEX**	77	0.47	1.80	0.0000	0.0166
**METHYLTRANSFERASE COMPLEX**	74	0.47	1.80	0.0000	0.0167
**NUCLEOTIDE EXCISION REPAIR PREINCISION COMPLEX STABILIZATION**	17	0.64	1.80	0.0017	0.0169
**ENDOMEMBRANE SYSTEM ORGANIZATION**	412	0.38	1.79	0.0000	0.0169
**HEPARAN SULFATE PROTEOGLYCAN BIOSYNTHETIC PROCESS**	23	0.60	1.79	0.0086	0.0170
**GOLGI TO PLASMA MEMBRANE TRANSPORT**	35	0.55	1.79	0.0017	0.0170
**PROTEIN ACETYLATION**	101	0.44	1.79	0.0000	0.0174
**RNA CAPPING**	33	0.55	1.79	0.0049	0.0174
**NEGATIVE REGULATION OF MRNA METABOLIC PROCESS**	28	0.57	1.79	0.0053	0.0174
**CHEMOKINE MEDIATED SIGNALING PATHWAY**	54	-0.55	-2.13	0.0000	0.0174
**TRANSITION METAL ION HOMEOSTASIS**	92	0.46	1.79	0.0015	0.0174
**FATTY ACYL COA BINDING**	28	0.57	1.79	0.0050	0.0175
**PEPTIDE N ACETYLTRANSFERASE ACTIVITY**	47	0.51	1.79	0.0000	0.0175
**VESICLE ORGANIZATION**	251	0.40	1.79	0.0000	0.0176
**AUTOPHAGOSOME MEMBRANE**	21	0.61	1.79	0.0035	0.0179
**NEGATIVE REGULATION OF TRANSCRIPTION FACTOR IMPORT INTO NUCLEUS**	34	0.54	1.79	0.0000	0.0180
**VITAMIN TRANSPORTER ACTIVITY**	21	0.60	1.78	0.0052	0.0183
**REGULATION OF CELLULAR AMIDE METABOLIC PROCESS**	317	0.38	1.78	0.0000	0.0185
**N ACYLTRANSFERASE ACTIVITY**	75	0.46	1.78	0.0000	0.0185
**NEGATIVE REGULATION OF TOR SIGNALING**	28	0.56	1.78	0.0052	0.0185

**OXIDOREDUCTASE COMPLEX**	84	0.46	1.78	0.0000	0.0185
**CELLULAR AMINO ACID CATABOLIC PROCESS**	101	0.44	1.78	0.0000	0.0185
**MACROAUTOPHAGY**	215	0.40	1.78	0.0000	0.0187
**PHOSPHOLIPID METABOLIC PROCESS**	328	0.38	1.78	0.0000	0.0188
**NUCLEOSIDE TRIPHOSPHATE BIOSYNTHETIC PROCESS**	48	0.50	1.78	0.0015	0.0188
**SMALL RIBOSOMAL SUBUNIT**	54	0.49	1.78	0.0016	0.0188
**PROTEIN SUMOYLATION**	103	0.44	1.78	0.0000	0.0189
**OXIDOREDUCTASE ACTIVITY ACTING ON CH OH GROUP OF DONORS**	111	0.43	1.78	0.0000	0.0189
**POSITIVE REGULATION OF CELLULAR PROTEIN CATABOLIC PROCESS**	176	0.41	1.78	0.0000	0.0189
**NEUTRAL LIPID METABOLIC PROCESS**	77	0.46	1.78	0.0015	0.0190
**PIGMENT METABOLIC PROCESS**	48	0.49	1.78	0.0033	0.0192
**FATTY ACID BETA OXIDATION USING ACYL COA DEHYDROGENASE**	16	0.66	1.78	0.0102	0.0193
**SULFUR COMPOUND BIOSYNTHETIC PROCESS**	182	0.41	1.77	0.0000	0.0193
**FATTY ACID TRANSPORT**	52	0.50	1.77	0.0029	0.0193
**DNA TEMPLATED TRANSCRIPTION INITIATION**	178	0.41	1.77	0.0000	0.0194
**MEDIATOR COMPLEX**	32	0.55	1.77	0.0046	0.0195
**ENDOSOME TO LYSOSOME TRANSPORT**	33	0.54	1.77	0.0065	0.0196
**NUCLEOSIDE TRIPHOSPHATE METABOLIC PROCESS**	191	0.40	1.77	0.0000	0.0196
**REGULATION OF FATTY ACID OXIDATION**	26	0.57	1.77	0.0050	0.0197
**ANTIGEN PROCESSING AND PRESENTATION OF EXOGENOUS PEPTIDE ANTIGEN VIA MHC CLASS I**	56	0.48	1.77	0.0000	0.0198
**REGULATION OF PROTEASOMAL PROTEIN CATABOLIC PROCESS**	163	0.41	1.77	0.0000	0.0199
**PROTEIN UBIQUITINATION INVOLVED IN UBIQUITIN DEPENDENT PROTEIN CATABOLIC PROCESS**	114	0.43	1.77	0.0000	0.0199
**INTRINSIC COMPONENT OF GOLGI MEMBRANE**	51	0.49	1.77	0.0017	0.0199
**PSEUDOURIDINE SYNTHESIS**	17	0.64	1.77	0.0069	0.0201
**7 METHYLGUANOSINE RNA CAPPING**	33	0.55	1.77	0.0000	0.0201
**UBIQUITIN LIKE PROTEIN LIGASE ACTIVITY**	176	0.41	1.77	0.0000	0.0201
**FLAVIN ADENINE DINUCLEOTIDE BINDING**	68	0.47	1.77	0.0016	0.0203

**TRANSFORMING GROWTH FACTOR BETA BINDING**	15	0.66	1.76	0.0086	0.0206
**NEGATIVE REGULATION OF RNA SPLICING**	18	0.63	1.76	0.0035	0.0210
**LIGAND DEPENDENT NUCLEAR RECEPTOR TRANSCRIPTION COACTIVATOR ACTIVITY**	44	0.51	1.76	0.0016	0.0210
**GLYCEROLIPID METABOLIC PROCESS**	326	0.38	1.76	0.0000	0.0210
**PROTEIN MONOUBIQUITINATION**	44	0.50	1.76	0.0016	0.0212
**NUCLEOTIDE EXCISION REPAIR**	93	0.44	1.76	0.0000	0.0221
**REGULATION OF RNA STABILITY**	127	0.42	1.76	0.0000	0.0221
**N ACETYLTRANSFERASE ACTIVITY**	66	0.47	1.76	0.0015	0.0221
**MONOCARBOXYLIC ACID METABOLIC PROCESS**	408	0.37	1.76	0.0000	0.0222
**POSITIVE REGULATION OF LEUKOCYTE CHEMOTAXIS**	70	-0.50	-2.09	0.0000	0.0225
**LYSOSOMAL TRANSPORT**	57	0.47	1.75	0.0017	0.0226
**O ACYLTRANSFERASE ACTIVITY**	44	0.51	1.75	0.0033	0.0226
**CYCLIN BINDING**	18	0.62	1.75	0.0017	0.0227
**PROTON TRANSPORTING ATP SYNTHASE COMPLEX**	16	0.64	1.75	0.0071	0.0227
**ANTIGEN PROCESSING AND PRESENTATION OF PEPTIDE ANTIGEN VIA MHC CLASS I**	79	0.46	1.75	0.0015	0.0227
**GLYCEROLIPID CATABOLIC PROCESS**	35	0.53	1.75	0.0017	0.0227
**MYD88 DEPENDENT TOLL LIKE RECEPTOR SIGNALING PATHWAY**	27	0.56	1.75	0.0050	0.0227
**LONG CHAIN FATTY ACID TRANSPORT**	38	0.52	1.75	0.0017	0.0229
**NUCLEOBASE CONTAINING SMALL MOLECULE INTERCONVERSION**	20	0.61	1.75	0.0017	0.0231
**NUCLEAR TRANSPORT**	295	0.38	1.75	0.0000	0.0234
**METHIONINE METABOLIC PROCESS**	18	0.61	1.75	0.0085	0.0234
**MITOCHONDRION LOCALIZATION**	28	0.56	1.75	0.0068	0.0234
**RESPONSE TO ENDOPLASMIC RETICULUM STRESS**	208	0.39	1.75	0.0000	0.0235
**2 OXOGLUTARATE METABOLIC PROCESS**	20	0.60	1.75	0.0102	0.0235
**SPLICEOSOMAL SNRNP ASSEMBLY**	30	0.55	1.74	0.0048	0.0237
**ENDOPLASMIC RETICULUM GOLGI INTERMEDIATE COMPARTMENT**	87	0.45	1.75	0.0000	0.0238
**PROTEASE BINDING**	86	0.45	1.74	0.0015	0.0238
**CYTOCHROME COMPLEX ASSEMBLY**	15	0.64	1.74	0.0037	0.0238
**CELLULAR TRANSITION METAL ION HOMEOSTASIS**	65	0.47	1.74	0.0015	0.0239

**CELLULAR CARBOHYDRATE BIOSYNTHETIC PROCESS**	38	0.52	1.74	0.0016	0.0240
**HYDRO LYASE ACTIVITY**	33	0.53	1.74	0.0049	0.0240
**DNA PACKAGING COMPLEX**	56	-0.52	-2.06	0.0000	0.0240
**REGULATION OF ALPHA BETA T CELL ACTIVATION**	56	-0.53	-2.10	0.0000	0.0243
**POSITIVE REGULATION OF TRIGLYCERIDE METABOLIC PROCESS**	20	0.61	1.74	0.0068	0.0245
**CELLULAR RESPONSE TO AMINO ACID STARVATION**	25	0.58	1.74	0.0036	0.0245
**DNA GEOMETRIC CHANGE**	67	0.46	1.74	0.0015	0.0246
**REGULATION OF TRANSLATIONAL INITIATION**	76	0.45	1.74	0.0000	0.0246
**MANGANESE ION BINDING**	47	0.50	1.74	0.0048	0.0247
**DEPHOSPHORYLATION**	249	0.38	1.74	0.0000	0.0247
**PURINE RIBONUCLEOSIDE MONOPHOSPHATE BIOSYNTHETIC PROCESS**	50	0.49	1.74	0.0031	0.0250
**NUCLEOTIDE EXCISION REPAIR DNA DUPLEX UNWINDING**	17	0.63	1.74	0.0052	0.0251
**LYSOPHOSPHOLIPID ACYLTRANSFERASE ACTIVITY**	18	0.62	1.74	0.0158	0.0251
**NADH DEHYDROGENASE COMPLEX**	38	0.52	1.74	0.0049	0.0252
**NUCLEUS ORGANIZATION**	115	0.43	1.74	0.0000	0.0252
**NADH DEHYDROGENASE ACTIVITY**	33	0.53	1.73	0.0080	0.0253
**PROTEIN ACYLATION**	130	0.42	1.73	0.0000	0.0253
**POSITIVE REGULATION OF TRANSCRIPTION FROM RNA POLYMERASE II PROMOTER INVOLVED IN CELLULAR RESPONSE TO CHEMICAL STIMULUS**	25	0.57	1.73	0.0052	0.0255
**RIBONUCLEOSIDE CATABOLIC PROCESS**	17	0.62	1.73	0.0054	0.0256
**POSITIVE REGULATION OF RESPONSE TO NUTRIENT LEVELS**	44	0.50	1.73	0.0032	0.0256
**POLY PURINE TRACT BINDING**	18	0.61	1.73	0.0052	0.0256
**CHEMOKINE RECEPTOR BINDING**	38	-0.56	-2.07	0.0000	0.0257
**THIOL DEPENDENT UBIQUITIN SPECIFIC PROTEASE ACTIVITY**	65	0.47	1.73	0.0015	0.0257
**MRNA 3 UTR BINDING**	45	0.50	1.73	0.0096	0.0260
**POSITIVE REGULATION OF RESPONSE TO EXTRACELLULAR STIMULUS**	44	0.50	1.73	0.0064	0.0261
**REGULATION OF SPROUTING ANGIOGENESIS**	26	-0.61	-2.07	0.0000	0.0261

**HISTONE H3 ACETYLATION**	37	0.52	1.73	0.0000	0.0261
**ORGANIC CYCLIC COMPOUND CATABOLIC PROCESS**	361	0.37	1.73	0.0000	0.0266
**PHOSPHOLIPID CATABOLIC PROCESS**	29	0.55	1.73	0.0113	0.0271
**CIS TRANS ISOMERASE ACTIVITY**	33	0.53	1.73	0.0032	0.0271
**POSITIVE REGULATION OF VIRAL PROCESS**	80	0.44	1.72	0.0015	0.0272
**REGULATION OF THE FORCE OF HEART CONTRACTION**	28	0.55	1.72	0.0070	0.0279
**N TERMINAL PROTEIN AMINO ACID MODIFICATION**	21	0.58	1.72	0.0117	0.0281
**SCF DEPENDENT PROTEASOMAL UBIQUITIN DEPENDENT PROTEIN CATABOLIC PROCESS**	20	0.60	1.72	0.0052	0.0284
**QUINONE BINDING**	16	0.63	1.72	0.0073	0.0287
**GOLGI TO VACUOLE TRANSPORT**	24	0.56	1.72	0.0053	0.0289
**STEROL TRANSPORT**	47	0.49	1.72	0.0015	0.0289
**GOLGI STACK**	104	0.42	1.72	0.0029	0.0292
**REVERSE CHOLESTEROL TRANSPORT**	16	0.61	1.72	0.0087	0.0292
**REPRESSING TRANSCRIPTION FACTOR BINDING**	50	0.48	1.72	0.0032	0.0294
**REGULATION OF LYMPHOCYTE MIGRATION**	32	-0.57	-2.02	0.0000	0.0294
**TERMINATION OF RNA POLYMERASE II TRANSCRIPTION**	46	0.49	1.71	0.0031	0.0295
**4 IRON 4 SULFUR CLUSTER BINDING**	38	0.51	1.71	0.0097	0.0295
**PURINE NUCLEOSIDE MONOPHOSPHATE BIOSYNTHETIC PROCESS**	50	0.49	1.71	0.0064	0.0295
**PHOSPHOLIPID BIOSYNTHETIC PROCESS**	215	0.39	1.71	0.0000	0.0295
**POSITIVE REGULATION OF DENDRITE DEVELOPMENT**	63	0.46	1.71	0.0015	0.0296
**PROTEIN PEPTIDYL PROLYL ISOMERIZATION**	32	0.53	1.71	0.0085	0.0298
**NEGATIVE REGULATION OF NF KAPPAB IMPORT INTO NUCLEUS**	16	0.61	1.71	0.0089	0.0299
**POSITIVE REGULATION OF DNA TEMPLATED TRANSCRIPTION INITIATION**	23	0.57	1.71	0.0051	0.0299
**ACYL COA DEHYDROGENASE ACTIVITY**	15	0.64	1.71	0.0091	0.0299
**POSITIVE REGULATION OF CHEMOTAXIS**	106	-0.46	-2.03	0.0000	0.0300
**POSITIVE REGULATION OF LEUKOCYTE MIGRATION**	96	-0.46	-2.03	0.0000	0.0303

**POSITIVE REGULATION OF NEUTROPHIL MIGRATION**	24	-0.62	-2.01	0.0000	0.0308
**NEUTRAL LIPID BIOSYNTHETIC PROCESS**	24	0.57	1.71	0.0117	0.0310
**POSITIVE REGULATION OF PROTEIN MODIFICATION BY SMALL PROTEIN CONJUGATION OR REMOVAL**	171	0.40	1.71	0.0000	0.0314
**RNA POLYMERASE ACTIVITY**	38	0.51	1.71	0.0078	0.0314
**ATP BIOSYNTHETIC PROCESS**	27	0.54	1.70	0.0052	0.0316
**TBP CLASS PROTEIN BINDING**	18	0.60	1.70	0.0141	0.0316
**ACYLGLYCEROL BIOSYNTHETIC PROCESS**	24	0.57	1.70	0.0118	0.0320
**PROTEIN K48 LINKED UBIQUITINATION**	40	0.50	1.70	0.0078	0.0321
**REGULATION OF CD4 POSITIVE ALPHA BETA T CELL ACTIVATION**	33	-0.58	-2.03	0.0000	0.0321
**EXON EXON JUNCTION COMPLEX**	21	0.59	1.70	0.0071	0.0324
**IRON ION TRANSPORT**	48	0.49	1.70	0.0016	0.0324
**PHOSPHATIDYLSERINE METABOLIC PROCESS**	27	0.53	1.70	0.0068	0.0327
**WATER SOLUBLE VITAMIN METABOLIC PROCESS**	73	0.44	1.70	0.0015	0.0336
**N METHYLTRANSFERASE ACTIVITY**	68	0.45	1.70	0.0030	0.0336
**LIPOPROTEIN METABOLIC PROCESS**	106	0.42	1.69	0.0015	0.0340
**GLYCEROPHOSPHOLIPID METABOLIC PROCESS**	272	0.37	1.69	0.0000	0.0341
**ORGANELLE ENVELOPE LUMEN**	71	0.45	1.70	0.0000	0.0341
**REGULATION OF FATTY ACID BETA OXIDATION**	15	0.62	1.69	0.0163	0.0342
**REGULATION OF DNA TEMPLATED TRANSCRIPTION INITIATION**	27	0.54	1.69	0.0066	0.0343
**LIPID MODIFICATION**	186	0.39	1.69	0.0000	0.0344
**NUCLEASE ACTIVITY**	163	0.40	1.69	0.0000	0.0344
**MICROTUBULE ORGANIZING CENTER ORGANIZATION**	76	0.44	1.69	0.0000	0.0347
**RRNA TRANSCRIPTION**	15	0.64	1.69	0.0124	0.0347
**REGULATION OF SYNCYTIUM FORMATION BY PLASMA MEMBRANE FUSION**	22	-0.63	-1.97	0.0000	0.0347
**SNRNA PROCESSING**	19	0.59	1.69	0.0156	0.0348
**FOLIC ACID CONTAINING COMPOUND METABOLIC PROCESS**	24	0.55	1.69	0.0048	0.0348
**LATE ENDOSOME**	169	0.39	1.69	0.0000	0.0352
**MACROMOLECULE METHYLATION**	167	0.39	1.69	0.0000	0.0354

**REGULATION OF PROTEIN MODIFICATION BY SMALL PROTEIN CONJUGATION OR REMOVAL**	245	0.38	1.69	0.0000	0.0356
**NUCLEOSIDE MONOPHOSPHATE METABOLIC PROCESS**	206	0.38	1.69	0.0000	0.0356
**REGULATION OF GRANULOCYTE CHEMOTAXIS**	34	-0.55	-1.96	0.0000	0.0356
**NUCLEOSIDE PHOSPHATE BIOSYNTHETIC PROCESS**	159	0.39	1.69	0.0000	0.0357
**GOLGI ASSOCIATED VESICLE MEMBRANE**	42	0.49	1.69	0.0132	0.0357
**REGULATION OF TYPE 2 IMMUNE RESPONSE**	24	-0.61	-1.99	0.0000	0.0357
**ATPASE ACTIVITY COUPLED**	282	0.37	1.69	0.0000	0.0360
**UBIQUITIN DEPENDENT PROTEIN CATABOLIC PROCESS VIA THE MULTIVESICULAR BODY SORTING PATHWAY**	16	0.62	1.69	0.0123	0.0360
**NUCLEOTIDYLTRANSFERASE ACTIVITY**	110	0.41	1.69	0.0015	0.0360
**LONG CHAIN FATTY ACID METABOLIC PROCESS**	64	0.46	1.68	0.0000	0.0361
**MITOTIC NUCLEAR DIVISION**	310	0.36	1.68	0.0000	0.0363
**ER TO GOLGI TRANSPORT VESICLE MEMBRANE**	33	0.50	1.68	0.0144	0.0363
**REGULATION OF MYOBLAST FUSION**	17	-0.67	-1.99	0.0000	0.0363
**ORGANIC ANION TRANSPORT**	332	0.36	1.68	0.0000	0.0364
**VOLTAGE GATED CATION CHANNEL ACTIVITY**	130	-0.41	-1.97	0.0000	0.0364
**RNA POLYADENYLATION**	28	0.55	1.68	0.0102	0.0368
**PROTEASOME CORE COMPLEX**	19	0.58	1.68	0.0067	0.0369
**PEPTIDE RECEPTOR ACTIVITY**	123	-0.42	-1.97	0.0000	0.0372
**GLYCEROLIPID BIOSYNTHETIC PROCESS**	190	0.38	1.68	0.0000	0.0377
**ACID THIOL LIGASE ACTIVITY**	16	0.62	1.68	0.0223	0.0380
**RESPONSE TO STARVATION**	136	0.40	1.68	0.0000	0.0380
**FOLIC ACID METABOLIC PROCESS**	15	0.63	1.68	0.0162	0.0380
**METAPHASE PLATE CONGRESSION**	33	0.52	1.68	0.0135	0.0380
**ORGANIC ACID TRANSPORT**	235	0.37	1.67	0.0000	0.0387
**CELLULAR RESPONSE TO VASCULAR ENDOTHELIAL GROWTH FACTOR STIMULUS**	29	-0.58	-1.97	0.0000	0.0391
**POSITIVE REGULATION OF CATABOLIC PROCESS**	351	0.36	1.67	0.0000	0.0393
**ENDOSOME ORGANIZATION**	52	0.47	1.67	0.0077	0.0398
**SULFUR AMINO ACID BIOSYNTHETIC PROCESS**	19	0.58	1.67	0.0136	0.0398

NUCLEOSIDE MONOPHOSPHATE BIOSYNTHETIC PROCESS	70	0.44	1.67	0.0015	0.0400
MRNA BINDING	135	0.40	1.67	0.0014	0.0402
REGULATION OF RYANODINE SENSITIVE CALCIUM RELEASE CHANNEL ACTIVITY	25	0.55	1.67	0.0174	0.0406
ALDITOL METABOLIC PROCESS	16	0.61	1.67	0.0088	0.0406
REGULATION OF CELL CYCLE PHASE TRANSITION	281	0.37	1.67	0.0000	0.0417
CELL CHEMOTAXIS	135	-0.42	-1.95	0.0000	0.0417
BLASTOCYST FORMATION	27	0.53	1.66	0.0101	0.0421
NUCLEAR LOCALIZATION SEQUENCE BINDING	19	0.59	1.66	0.0124	0.0422
OXIDOREDUCTASE ACTIVITY ACTING ON THE CH OH GROUP OF DONORS NAD OR NADP AS ACCEPTOR	92	0.42	1.66	0.0030	0.0431
NITROGEN COMPOUND TRANSPORT	435	0.35	1.66	0.0000	0.0437
PHOSPHATASE ACTIVITY	241	0.37	1.66	0.0000	0.0440
GLOBAL GENOME NUCLEOTIDE EXCISION REPAIR	26	0.54	1.66	0.0179	0.0441
2 IRON 2 SULFUR CLUSTER BINDING	18	0.59	1.66	0.0153	0.0444
NEGATIVE REGULATION OF REACTIVE OXYGEN SPECIES METABOLIC PROCESS	37	0.49	1.66	0.0082	0.0444
HYDROGEN EXPORTING ATPASE ACTIVITY	24	0.55	1.66	0.0212	0.0447
VITAMIN D RECEPTOR BINDING	17	0.59	1.65	0.0189	0.0452
CYSTEINE TYPE PEPTIDASE ACTIVITY	144	0.39	1.65	0.0014	0.0453
ACYLGLYCEROL O ACYLTRANSFERASE ACTIVITY	26	0.54	1.65	0.0067	0.0459
POSITIVE REGULATION OF LIPID CATABOLIC PROCESS	25	0.53	1.65	0.0177	0.0459
ALPHA AMINO ACID METABOLIC PROCESS	206	0.37	1.65	0.0013	0.0460
TRANSCRIPTION ELONGATION FACTOR COMPLEX	40	0.49	1.65	0.0109	0.0461
TRICARBOXYLIC ACID METABOLIC PROCESS	34	0.51	1.65	0.0087	0.0461
NUCLEOTIDE SUGAR METABOLIC PROCESS	31	0.51	1.65	0.0099	0.0462
PTERIDINE CONTAINING COMPOUND METABOLIC PROCESS	31	0.52	1.65	0.0105	0.0462
ERAD PATHWAY	67	0.44	1.65	0.0031	0.0466
LIGASE ACTIVITY FORMING CARBON SULFUR BONDS	33	0.51	1.65	0.0101	0.0471
RESPONSE TO UV	100	0.41	1.65	0.0014	0.0472

**RESPONSE TO MONOAMINE**	32	0.51	1.65	0.0136	0.0472
**REGULATION OF PROTEIN TARGETING TO MITOCHONDRION**	86	0.42	1.65	0.0015	0.0472
**MITOCHONDRIAL MEMBRANE ORGANIZATION**	73	0.43	1.65	0.0076	0.0473
**NAD BINDING**	52	0.47	1.64	0.0015	0.0479
**ESTABLISHMENT OF PROTEIN LOCALIZATION TO MEMBRANE**	227	0.37	1.64	0.0000	0.0484
**GOLGI TO PLASMA MEMBRANE PROTEIN TRANSPORT**	24	0.54	1.64	0.0126	0.0485
**MEMBRANE FUSION**	139	0.39	1.64	0.0014	0.0495
**ESTABLISHMENT OF PROTEIN LOCALIZATION TO VACUOLE**	24	0.54	1.64	0.0203	0.0498
**REGULATION OF SPINDLE ORGANIZATION**	17	0.59	1.64	0.0199	0.0499
**MEMBRANE LIPID METABOLIC PROCESS**	154	0.39	1.64	0.0000	0.0505
**POSITIVE REGULATION OF PROTEIN CATABOLIC PROCESS**	232	0.36	1.64	0.0000	0.0506
**TRNA SPECIFIC RIBONUCLEASE ACTIVITY**	15	0.61	1.64	0.0190	0.0509
**ACTIVATION OF MAPKK ACTIVITY**	45	0.47	1.64	0.0063	0.0512
**COFACTOR TRANSPORT**	22	0.56	1.63	0.0269	0.0512
**TRANSCRIPTION INITIATION FROM RNA POLYMERASE II PROMOTER**	141	0.39	1.63	0.0028	0.0513
**CYTOSOLIC PART**	175	0.38	1.63	0.0000	0.0515
**REGULATION OF LYMPHOCYTE CHEMOTAXIS**	17	-0.66	-1.92	0.0000	0.0521
**INNATE IMMUNE RESPONSE ACTIVATING CELL SURFACE RECEPTOR SIGNALING PATHWAY**	91	0.42	1.63	0.0117	0.0527
**REGULATION OF PROTEIN STABILITY**	196	0.37	1.63	0.0000	0.0530
**REGULATION OF NFAT PROTEIN IMPORT INTO NUCLEUS**	15	0.60	1.63	0.0295	0.0530
**REGULATION OF PROTEIN HOMODIMERIZATION ACTIVITY**	20	0.57	1.63	0.0199	0.0531
**ALPHA AMINO ACID CATABOLIC PROCESS**	85	0.42	1.63	0.0092	0.0533
**U4 U6 X U5 TRI SNRNP COMPLEX**	18	0.58	1.63	0.0162	0.0533
**ORGANELLE ASSEMBLY**	407	0.35	1.63	0.0000	0.0534
**POSITIVE REGULATION OF VIRAL TRANSCRIPTION**	35	0.50	1.63	0.0119	0.0534
**PHOSPHATIDYLINOSITOL PHOSPHATE PHOSPHATASE ACTIVITY**	24	0.54	1.63	0.0171	0.0534
**STEROL TRANSPORTER ACTIVITY**	16	0.59	1.63	0.0252	0.0535

**MLL1 2 COMPLEX**	21	0.55	1.63	0.0208	0.0539
**PROTON TRANSPORTING V TYPE ATPASE COMPLEX**	21	0.56	1.63	0.0202	0.0540
**POSITIVE REGULATION OF LYMPHOCYTE MIGRATION**	22	-0.60	-1.91	0.0022	0.0541
**NUCLEOTIDE SUGAR BIOSYNTHETIC PROCESS**	18	0.58	1.63	0.0243	0.0543
**MONOSACCHARIDE METABOLIC PROCESS**	157	0.39	1.62	0.0000	0.0545
**MACROMOLECULE TRANSMEMBRANE TRANSPORTER ACTIVITY**	21	0.56	1.62	0.0102	0.0553
**NEGATIVE REGULATION OF TRANSFERASE ACTIVITY**	309	0.36	1.62	0.0000	0.0559
**CELLULAR RESPONSE TO STARVATION**	101	0.40	1.62	0.0030	0.0562
**FERRIC IRON TRANSPORT**	33	0.49	1.62	0.0189	0.0562
**REGULATION OF LEUKOCYTE CHEMOTAXIS**	82	-0.45	-1.91	0.0000	0.0562
**PIGMENT BIOSYNTHETIC PROCESS**	41	0.48	1.62	0.0095	0.0563
**REGULATION OF MITOTIC CELL CYCLE**	408	0.34	1.62	0.0000	0.0564
**RNA POLYMERASE BINDING**	28	0.51	1.62	0.0152	0.0566
**REGULATION OF ESTABLISHMENT OF PLANAR POLARITY**	106	0.40	1.62	0.0043	0.0567
**LIPID DIGESTION**	20	0.57	1.62	0.0190	0.0567
**CARBOHYDRATE TRANSMEMBRANE TRANSPORT**	18	0.57	1.62	0.0169	0.0575
**PEPTIDYL THREONINE MODIFICATION**	43	0.47	1.62	0.0159	0.0576
**DNA REPAIR**	379	0.35	1.62	0.0000	0.0576
**PROTON TRANSPORTING TWO SECTOR ATPASE COMPLEX PROTON TRANSPORTING DOMAIN**	15	0.61	1.62	0.0322	0.0576
**ATPASE ACTIVITY**	368	0.34	1.61	0.0000	0.0577
**PRE AUTOPHAGOSOMAL STRUCTURE**	21	0.55	1.62	0.0269	0.0577
**EMBRYONIC FORELIMB MORPHOGENESIS**	29	-0.57	-1.91	0.0000	0.0577
**VACUOLE FUSION**	17	0.58	1.61	0.0201	0.0577
**COFACTOR TRANSPORTER ACTIVITY**	18	0.57	1.61	0.0204	0.0577
**REGULATION OF RELEASE OF SEQUESTERED CALCIUM ION INTO CYTOSOL BY SARCOPLASMIC RETICULUM**	23	0.54	1.61	0.0256	0.0579
**NEGATIVE REGULATION OF AUTOPHAGY**	45	0.46	1.61	0.0170	0.0579
**CARBOHYDRATE CATABOLIC PROCESS**	94	0.41	1.61	0.0058	0.0580

**PHOSPHORIC ESTER HYDROLASE ACTIVITY**	330	0.35	1.61	0.0000	0.0582
**NUCLEOCYTOPLASMIC TRANSPORTER ACTIVITY**	22	0.56	1.61	0.0256	0.0583
**PROTEIN LIPID COMPLEX SUBUNIT ORGANIZATION**	31	0.51	1.61	0.0165	0.0583
**REGULATION OF CELL CYCLE PROCESS**	480	0.34	1.61	0.0000	0.0583
**PYRIMIDINE RIBONUCLEOSIDE METABOLIC PROCESS**	25	0.53	1.61	0.0247	0.0592
**TRANSLATION INITIATION FACTOR BINDING**	24	0.54	1.61	0.0178	0.0595
**SMALL MOLECULE BIOSYNTHETIC PROCESS**	389	0.34	1.61	0.0000	0.0600
**LEUKOCYTE APOPTOTIC PROCESS**	18	0.58	1.61	0.0211	0.0614
**MISFOLDED OR INCOMPLETELY SYNTHESIZED PROTEIN CATABOLIC PROCESS**	15	0.60	1.60	0.0259	0.0618
**PROTEIN REFOLDING**	16	-0.65	-1.89	0.0000	0.0618
**RETROGRADE TRANSPORT VESICLE RECYCLING WITHIN GOLGI**	15	0.60	1.60	0.0294	0.0620
**PROTEIN AUTOUBIQUITINATION**	40	0.47	1.60	0.0033	0.0621
**POSITIVE REGULATION OF GENE EXPRESSION EPIGENETIC**	56	0.44	1.60	0.0048	0.0625
**PYRIMIDINE CONTAINING COMPOUND BIOSYNTHETIC PROCESS**	34	0.49	1.60	0.0223	0.0628
**VIRAL LIFE CYCLE**	242	0.36	1.60	0.0000	0.0630
**TRIVALENT INORGANIC CATION TRANSPORT**	33	0.49	1.60	0.0172	0.0632
**INTERFERON GAMMA MEDIATED SIGNALING PATHWAY**	43	-0.49	-1.89	0.0000	0.0635
**ORGANONITROGEN COMPOUND CATABOLIC PROCESS**	298	0.35	1.60	0.0000	0.0639
**PHOSPHATASE REGULATOR ACTIVITY**	73	0.43	1.60	0.0078	0.0639
**PROTEIN KINASE COMPLEX**	80	0.41	1.60	0.0090	0.0645
**CUL3 RING UBIQUITIN LIGASE COMPLEX**	57	0.43	1.60	0.0099	0.0645
**PROTEIN DEPHOSPHORYLATION**	175	0.37	1.60	0.0014	0.0650
**TRANSFERASE ACTIVITY TRANSFERRING HEXOSYL GROUPS**	153	0.37	1.60	0.0014	0.0651
**NUCLEAR REPLICATION FORK**	34	0.48	1.59	0.0216	0.0656
**REGULATION OF MACROPHAGE DERIVED FOAM CELL DIFFERENTIATION**	25	0.52	1.59	0.0348	0.0656
**ORGANIC ANION TRANSMEMBRANE TRANSPORTER ACTIVITY**	155	0.37	1.59	0.0014	0.0662

**ORGANIC ACID TRANSMEMBRANE TRANSPORT**	95	0.40	1.59	0.0015	0.0663
**RNA POLYMERASE II CARBOXY TERMINAL DOMAIN KINASE ACTIVITY**	15	0.60	1.59	0.0249	0.0664
**TRANSCRIPTION COACTIVATOR ACTIVITY**	266	0.35	1.59	0.0000	0.0665
**REGULATION OF DNA TEMPLATED TRANSCRIPTION ELONGATION**	36	0.47	1.59	0.0149	0.0666
**ENERGY COUPLED PROTON TRANSPORT DOWN ELECTROCHEMICAL GRADIENT**	17	0.57	1.59	0.0215	0.0671
**REGULATION OF SENSORY PERCEPTION OF PAIN**	34	0.49	1.59	0.0173	0.0673
**REGULATION OF PROTEIN PHOSPHATASE TYPE 2A ACTIVITY**	22	0.54	1.59	0.0254	0.0675
**CLATHRIN COATED VESICLE**	133	0.38	1.59	0.0014	0.0679
**PROTEIN LIPID COMPLEX REMODELING**	21	0.54	1.59	0.0231	0.0679
**REGULATION OF SENSORY PERCEPTION**	34	0.49	1.59	0.0148	0.0679
**VASODILATION**	24	0.52	1.59	0.0215	0.0680
**POSITIVE REGULATION OF MULTI ORGANISM PROCESS**	138	0.38	1.59	0.0000	0.0688
**REGULATION OF STEROL TRANSPORT**	35	0.47	1.58	0.0165	0.0698
**PLASMA LIPOPROTEIN PARTICLE CLEARANCE**	21	0.54	1.58	0.0426	0.0699
**NEGATIVE REGULATION OF CELLULAR CATABOLIC PROCESS**	137	0.37	1.58	0.0055	0.0700
**CELLULAR MODIFIED AMINO ACID METABOLIC PROCESS**	189	0.36	1.58	0.0013	0.0701
**PLASMA LIPOPROTEIN PARTICLE REMODELING**	21	0.54	1.58	0.0365	0.0705
**PURINE CONTAINING COMPOUND METABOLIC PROCESS**	347	0.34	1.58	0.0000	0.0707
**ATP SYNTHESIS COUPLED PROTON TRANSPORT**	17	0.57	1.58	0.0270	0.0707
**ORGANELLE FISSION**	414	0.34	1.58	0.0000	0.0708
**MODULATION BY HOST OF VIRAL PROCESS**	15	0.57	1.58	0.0457	0.0708
**RNA STABILIZATION**	30	0.49	1.58	0.0322	0.0708
**MACROMOLECULAR COMPLEX REMODELING**	21	0.54	1.58	0.0243	0.0709
**LIPID BIOSYNTHETIC PROCESS**	466	0.33	1.58	0.0000	0.0717
**LYMPHOCYTE APOPTOTIC PROCESS**	15	0.60	1.58	0.0297	0.0717
**G PROTEIN COUPLED PURINERGIC RECEPTOR SIGNALING PATHWAY**	18	0.56	1.58	0.0314	0.0718

**VITAMIN TRANSPORT**	29	0.49	1.58	0.0229	0.0718
**NEGATIVE REGULATION OF BLOOD PRESSURE**	42	0.46	1.58	0.0159	0.0718
**NEGATIVE REGULATION OF BLOOD CIRCULATION**	33	0.49	1.58	0.0251	0.0718
**NEGATIVE REGULATION OF MITOCHONDRION ORGANIZATION**	34	0.49	1.58	0.0085	0.0718
**CARBOHYDRATE TRANSPORTER ACTIVITY**	36	0.48	1.58	0.0178	0.0718
**UBIQUITIN LIKE PROTEIN CONJUGATING ENZYME ACTIVITY**	25	0.52	1.58	0.0321	0.0719
**POSITIVE REGULATION OF PROTEASOMAL PROTEIN CATABOLIC PROCESS**	94	0.39	1.58	0.0107	0.0724
**DICARBOXYLIC ACID METABOLIC PROCESS**	92	0.39	1.58	0.0060	0.0725
**EARLY ENDOSOME**	255	0.35	1.57	0.0000	0.0733
**NUCLEOTIDE BINDING DOMAIN LEUCINE RICH REPEAT CONTAINING RECEPTOR SIGNALING PATHWAY**	25	0.52	1.57	0.0202	0.0734
**NEGATIVE REGULATION OF PEPTIDYL SERINE PHOSPHORYLATION**	22	0.53	1.57	0.0251	0.0735
**MODIFIED AMINO ACID TRANSPORT**	23	0.52	1.57	0.0271	0.0736
**HISTONE MRNA METABOLIC PROCESS**	26	0.52	1.57	0.0261	0.0738
**ORGANELLE FUSION**	112	0.39	1.57	0.0029	0.0739
**ATPASE BINDING**	66	0.42	1.57	0.0121	0.0742
**PHAGOSOME ACIDIFICATION**	24	0.52	1.57	0.0220	0.0745
**RNA POLYMERASE CORE ENZYME BINDING**	18	0.56	1.57	0.0327	0.0745
**MAINTENANCE OF LOCATION**	122	0.38	1.57	0.0014	0.0750
**REGULATION OF NEUTROPHIL CHEMOTAXIS**	24	-0.58	-1.87	0.0022	0.0754
**THREONINE TYPE PEPTIDASE ACTIVITY**	20	0.53	1.57	0.0377	0.0756
**MITOCHONDRIAL GENOME MAINTENANCE**	18	0.55	1.57	0.0365	0.0766
**REGULATION OF CELLULAR AMINO ACID METABOLIC PROCESS**	60	0.42	1.57	0.0180	0.0767
**DEFENSE RESPONSE TO VIRUS**	120	-0.41	-1.86	0.0000	0.0770
**PIGMENT GRANULE LOCALIZATION**	22	0.52	1.56	0.0347	0.0776
**ORGANIC CATION TRANSMEMBRANE TRANSPORTER ACTIVITY**	15	0.58	1.56	0.0363	0.0776
**ENERGY RESERVE METABOLIC PROCESS**	60	0.42	1.56	0.0127	0.0776
**RESPONSE TO INCREASED OXYGEN LEVELS**	22	0.53	1.56	0.0369	0.0776

**THYMUS DEVELOPMENT**	43	0.45	1.56	0.0142	0.0777
**CCR CHEMOKINE RECEPTOR BINDING**	22	-0.58	-1.86	0.0000	0.0778
**REGULATION OF TELOMERE MAINTENANCE**	57	0.42	1.56	0.0202	0.0778
**T CELL ACTIVATION INVOLVED IN IMMUNE RESPONSE**	48	-0.48	-1.86	0.0054	0.0779
**REGULATION OF RELEASE OF CYTOCHROME C FROM MITOCHONDRIA**	39	0.46	1.56	0.0241	0.0780
**TRANSCRIPTION FACTOR TFIID COMPLEX**	18	0.55	1.56	0.0315	0.0781
**HYDROLASE ACTIVITY ACTING ON CARBON NITROGEN BUT NOT PEPTIDE BONDS IN LINEAR AMIDES**	74	0.41	1.56	0.0102	0.0781
**1 PHOSPHATIDYLINOSITOL BINDING**	17	0.56	1.56	0.0370	0.0781
**REGULATION OF PROTEIN CATABOLIC PROCESS**	349	0.33	1.56	0.0000	0.0788
**PROTEIN PHOSPHATASE 2A BINDING**	26	0.51	1.56	0.0315	0.0789
**ELECTRON CARRIER ACTIVITY**	98	0.40	1.56	0.0085	0.0789
**AMMONIUM ION METABOLIC PROCESS**	151	0.37	1.56	0.0027	0.0792
**PROTEIN LOCALIZATION TO CELL SURFACE**	21	0.53	1.56	0.0382	0.0792
**ANION TRANSPORT**	441	0.33	1.56	0.0000	0.0792
**THYROID HORMONE RECEPTOR BINDING**	26	0.50	1.56	0.0235	0.0793
**REGULATION OF CHROMOSOME SEGREGATION**	74	0.40	1.56	0.0091	0.0793
**EARLY ENDOSOME MEMBRANE**	89	0.40	1.56	0.0118	0.0794
**SMALL NUCLEAR RIBONUCLEOPROTEIN COMPLEX**	51	0.43	1.56	0.0228	0.0794
**POSITIVE REGULATION OF PROTEOLYSIS**	323	0.34	1.56	0.0000	0.0794
**INTERACTION WITH SYMBIONT**	43	0.45	1.56	0.0144	0.0803
**LIPASE ACTIVITY**	107	0.39	1.56	0.0044	0.0804
**RESPONSE TO HYPEROXIA**	22	0.53	1.56	0.0374	0.0804
**PYRIMIDINE RIBONUCLEOTIDE METABOLIC PROCESS**	22	0.53	1.56	0.0253	0.0805
**REGULATION OF CHOLESTEROL TRANSPORT**	35	0.47	1.55	0.0242	0.0806
**VOLTAGE GATED POTASSIUM CHANNEL ACTIVITY**	88	-0.42	-1.84	0.0000	0.0808
**REGULATION OF CELLULAR KETONE METABOLIC PROCESS**	149	0.36	1.55	0.0041	0.0809
**MEMBRANE PROTEIN PROTEOLYSIS**	29	0.49	1.55	0.0233	0.0810
**PROTEASOME COMPLEX**	69	0.41	1.55	0.0138	0.0810
**TELOMERIC DNA BINDING**	25	0.51	1.55	0.0309	0.0810

**ADAPTIVE IMMUNE RESPONSE**	185	-0.37	-1.85	0.0000	0.0810
**FC RECEPTOR SIGNALING PATHWAY**	169	0.36	1.55	0.0027	0.0811
**REGULATION OF PROTEASOMAL UBIQUITIN DEPENDENT PROTEIN CATABOLIC PROCESS**	133	0.37	1.55	0.0056	0.0816
**APOPTOTIC MITOCHONDRIAL CHANGES**	45	0.45	1.55	0.0174	0.0818
**REGULATION OF NEUTROPHIL MIGRATION**	29	-0.55	-1.85	0.0000	0.0825
**NEGATIVE REGULATION OF RELEASE OF CYTOCHROME C FROM MITOCHONDRIA**	16	0.57	1.55	0.0357	0.0831
**COLLAGEN TRIMER**	75	-0.43	-1.83	0.0000	0.0833
**CELLULAR RESPONSE TO INTERFERON GAMMA**	81	-0.43	-1.83	0.0000	0.0834
**LIPID STORAGE**	25	0.51	1.55	0.0399	0.0836
**POSITIVE REGULATION OF CELLULAR AMIDE METABOLIC PROCESS**	102	0.38	1.55	0.0099	0.0838
**RESPONSE TO INTERFERON GAMMA**	101	-0.41	-1.83	0.0000	0.0847
**MAST CELL ACTIVATION**	19	-0.61	-1.84	0.0023	0.0848
**PROTEIN DESTABILIZATION**	30	0.49	1.55	0.0267	0.0849
**GLUCAN METABOLIC PROCESS**	46	0.44	1.55	0.0155	0.0849
**CHROMATIN MODIFICATION**	454	0.32	1.55	0.0000	0.0849
**SPLICEOSOMAL TRI SNRNP COMPLEX**	23	0.52	1.55	0.0275	0.0850
**INTRAMOLECULAR TRANSFERASE ACTIVITY**	22	0.53	1.55	0.0537	0.0850
**POSITIVE REGULATION OF DENDRITIC SPINE DEVELOPMENT**	34	0.48	1.54	0.0252	0.0852
**PROTEIN LOCALIZATION TO ENDOPLASMIC RETICULUM**	103	0.39	1.54	0.0117	0.0853
**REGULATION OF ENDOTHELIAL CELL CHEMOTAXIS**	16	-0.64	-1.83	0.0023	0.0855
**NEGATIVE REGULATION OF CARBOHYDRATE METABOLIC PROCESS**	39	0.45	1.54	0.0157	0.0863
**NEGATIVE REGULATION OF TRANSLATIONAL INITIATION**	20	0.53	1.54	0.0409	0.0867
**PROTON TRANSPORTING TWO SECTOR ATPASE COMPLEX CATALYTIC DOMAIN**	16	0.56	1.54	0.0388	0.0867
**NUCLEAR ENVELOPE**	356	0.33	1.54	0.0000	0.0867
**NUCLEOSOME BINDING**	39	0.45	1.54	0.0291	0.0868
**CYSTEINE TYPE ENDOPEPTIDASE ACTIVITY**	71	0.41	1.54	0.0111	0.0868
**ORGANIC ACID TRANSMEMBRANE TRANSPORTER ACTIVITY**	127	0.37	1.54	0.0043	0.0868

**PHOSPHATIDYLINOSITOL 3 PHOSPHATE BINDING**	29	0.49	1.54	0.0334	0.0869
**LIVER REGENERATION**	17	0.55	1.54	0.0444	0.0875
**PROTEIN PHOSPHATASE TYPE 2A REGULATOR ACTIVITY**	19	0.55	1.54	0.0501	0.0875
**SCF UBIQUITIN LIGASE COMPLEX**	31	0.48	1.54	0.0464	0.0876
**REGULATION OF EXOSOMAL SECRETION**	15	0.57	1.54	0.0426	0.0877
**POSITIVE REGULATION OF G1 S TRANSITION OF MITOTIC CELL CYCLE**	22	0.52	1.54	0.0497	0.0881
**REGULATION OF PROTEIN LOCALIZATION TO CELL SURFACE**	24	0.51	1.54	0.0268	0.0882
**FORELIMB MORPHOGENESIS**	35	-0.52	-1.82	0.0000	0.0883
**POSITIVE REGULATION OF ALPHA BETA T CELL ACTIVATION**	42	-0.49	-1.82	0.0025	0.0883
**RNA POLYMERASE II CORE BINDING**	15	0.58	1.54	0.0266	0.0885
**GLUTAMINE METABOLIC PROCESS**	23	0.52	1.54	0.0364	0.0887
**ATP DEPENDENT DNA HELICASE ACTIVITY**	29	0.48	1.54	0.0372	0.0890
**DNA HELICASE ACTIVITY**	45	0.44	1.54	0.0263	0.0892
**COVALENT CHROMATIN MODIFICATION**	294	0.33	1.53	0.0012	0.0895
**PHOSPHATIDYLINOSITOL BIOSYNTHETIC PROCESS**	110	0.37	1.53	0.0058	0.0899
**CELLULAR RESPONSE TO TOPOLOGICALLY INCORRECT PROTEIN**	108	0.38	1.53	0.0132	0.0900
**PHOSPHATIDYLGLYCEROL METABOLIC PROCESS**	29	0.48	1.53	0.0200	0.0900
**LIPID LOCALIZATION**	232	0.34	1.53	0.0000	0.0904
**DICARBOXYLIC ACID TRANSMEMBRANE TRANSPORTER ACTIVITY**	31	0.47	1.53	0.0372	0.0908
**NEGATIVE REGULATION OF SMOOTH MUSCLE CONTRACTION**	15	0.57	1.53	0.0435	0.0911
**CELLULAR GLUCAN METABOLIC PROCESS**	46	0.44	1.53	0.0145	0.0911
**PYRIMIDINE NUCLEOTIDE BIOSYNTHETIC PROCESS**	26	0.49	1.53	0.0347	0.0915
**T CELL RECEPTOR SIGNALING PATHWAY**	124	0.37	1.53	0.0072	0.0915
**POSITIVE REGULATION OF CELL GROWTH**	134	0.37	1.53	0.0043	0.0916
**PRECATALYTIC SPLICEOSOME**	19	0.54	1.53	0.0489	0.0920
**UBIQUITIN LIKE PROTEIN BINDING**	106	0.38	1.53	0.0088	0.0924
**ORGANELLE TRANSPORT ALONG MICROTUBULE**	49	0.43	1.53	0.0194	0.0924
**DRUG BINDING**	97	0.38	1.53	0.0130	0.0929

**NUCLEAR UBIQUITIN LIGASE COMPLEX**	38	0.45	1.53	0.0256	0.0934
**REGULATION OF DNA METABOLIC PROCESS**	292	0.33	1.53	0.0000	0.0936
**GLUCOSE METABOLIC PROCESS**	101	0.38	1.53	0.0121	0.0937
**CALCIUM DEPENDENT CYSTEINE TYPE ENDOPEPTIDASE ACTIVITY**	18	0.54	1.52	0.0381	0.0939
**PHOSPHOPROTEIN PHOSPHATASE ACTIVITY**	164	0.35	1.52	0.0123	0.0944
**PH REDUCTION**	33	0.47	1.52	0.0361	0.0953
**TRANS GOLGI NETWORK TRANSPORT VESICLE**	25	0.49	1.52	0.0527	0.0964
**NEUROTRANSMITTER UPTAKE**	15	0.56	1.52	0.0525	0.0971
**PHOSPHOTRANSFERASE ACTIVITY FOR OTHER SUBSTITUTED PHOSPHATE GROUPS**	17	0.54	1.52	0.0421	0.0987
**NEGATIVE REGULATION OF LEUKOCYTE APOPTOTIC PROCESS**	35	-0.51	-1.80	0.0000	0.0990
**DISULFIDE OXIDOREDUCTASE ACTIVITY**	29	0.48	1.51	0.0368	0.1007
**ACYLGLYCEROL CATABOLIC PROCESS**	25	0.49	1.51	0.0390	0.1009
**TRANSCRIPTIONAL REPRESSOR COMPLEX**	67	0.40	1.51	0.0195	0.1010
**POSITIVE REGULATION OF CARDIAC MUSCLE CELL PROLIFERATION**	17	0.55	1.51	0.0511	0.1017
**REGULATION OF AUTOPHAGY**	219	0.34	1.51	0.0013	0.1019
**POSITIVE REGULATION OF CD4 POSITIVE ALPHA BETA T CELL ACTIVATION**	24	-0.55	-1.80	0.0047	0.1024
**RETINOIC ACID METABOLIC PROCESS**	16	-0.62	-1.79	0.0046	0.1025
**PROTEIN TARGETING TO MEMBRANE**	133	0.36	1.51	0.0100	0.1029
**REGULATION OF DNA REPLICATION**	143	0.36	1.51	0.0055	0.1034
**G2 DNA DAMAGE CHECKPOINT**	30	0.46	1.51	0.0390	0.1039
**CARBON OXYGEN LYASE ACTIVITY**	52	0.42	1.51	0.0243	0.1042
**REGULATION OF CHEMOTAXIS**	162	-0.38	-1.79	0.0000	0.1046
**REGULATION OF POSITIVE CHEMOTAXIS**	21	-0.57	-1.79	0.0023	0.1051
**FERROUS IRON BINDING**	19	0.53	1.51	0.0436	0.1053
**INTESTINAL ABSORPTION**	25	0.50	1.51	0.0485	0.1057
**R SMAD BINDING**	20	-0.57	-1.79	0.0144	0.1060
**U2 SNRNP**	16	0.54	1.51	0.0545	0.1064
**EXECUTION PHASE OF APOPTOSIS**	50	0.42	1.50	0.0244	0.1067
**NEUTRAL LIPID CATABOLIC PROCESS**	25	0.49	1.50	0.0566	0.1067
**LOCALIZATION WITHIN MEMBRANE**	111	0.37	1.50	0.0074	0.1077
**3 5 CYCLIC AMP PHOSPHODIESTERASE ACTIVITY**	15	0.56	1.50	0.0480	0.1082

**HYDROLASE ACTIVITY HYDROLYZING O GLYCOSYL COMPOUNDS**	75	0.39	1.50	0.0137	0.1084
**PURINERGIC RECEPTOR ACTIVITY**	23	0.51	1.50	0.0498	0.1084
**GLUCAN BIOSYNTHETIC PROCESS**	16	0.54	1.50	0.0503	0.1084
**REGULATION OF CARDIAC MUSCLE CONTRACTION**	64	0.41	1.50	0.0214	0.1085
**REGULATION OF SISTER CHROMATID SEGREGATION**	57	0.41	1.50	0.0283	0.1096
**REGULATION OF CELL MIGRATION INVOLVED IN SPROUTING ANGIOGENESIS**	19	-0.58	-1.78	0.0093	0.1099
**PROTEIN K63 LINKED DEUBIQUITINATION**	21	0.51	1.50	0.0371	0.1111
**OXIDOREDUCTASE ACTIVITY OXIDIZING METAL IONS**	17	0.55	1.50	0.0348	0.1114
**REGULATION OF T HELPER CELL DIFFERENTIATION**	22	-0.56	-1.77	0.0068	0.1115
**HEXOSE METABOLIC PROCESS**	134	0.36	1.50	0.0135	0.1116
**NUCLEAR MEMBRANE**	239	0.33	1.50	0.0013	0.1117
**NUCLEAR TRANSCRIPTIONAL REPRESSOR COMPLEX**	19	0.51	1.50	0.0494	0.1118
**ENDOPLASMIC RETICULUM GOLGI INTERMEDIATE COMPARTMENT MEMBRANE**	52	0.42	1.50	0.0264	0.1118
**ER ASSOCIATED UBIQUITIN DEPENDENT PROTEIN CATABOLIC PROCESS**	57	0.41	1.50	0.0266	0.1118
**MODULATION BY SYMBIONT OF HOST CELLULAR PROCESS**	24	0.50	1.50	0.0412	0.1119
**LEUKOCYTE CHEMOTAXIS**	94	-0.40	-1.77	0.0000	0.1120
**PROTEIN METHYLATION**	99	0.37	1.49	0.0231	0.1121
**POLYSACCHARIDE METABOLIC PROCESS**	65	0.40	1.49	0.0188	0.1122
**TOLL LIKE RECEPTOR SIGNALING PATHWAY**	77	0.39	1.49	0.0138	0.1122
**ENDORIBONUCLEASE ACTIVITY**	39	0.44	1.49	0.0257	0.1131
**ATP HYDROLYSIS COUPLED TRANSMEMBRANE TRANSPORT**	34	0.46	1.49	0.0419	0.1131
**NEGATIVE REGULATION OF CATABOLIC PROCESS**	182	0.34	1.49	0.0026	0.1133
**POLYSACCHARIDE BIOSYNTHETIC PROCESS**	30	0.47	1.49	0.0458	0.1134
**REGULATION OF MRNA SPLICING VIA SPLICEOSOME**	46	0.42	1.49	0.0349	0.1135
**PROTEIN PHOSPHATASE TYPE 2A COMPLEX**	17	0.54	1.49	0.0559	0.1141

**RESPONSE TO LIPOPROTEIN PARTICLE**	19	0.52	1.49	0.0624	0.1141
**ANTIGEN PROCESSING AND PRESENTATION OF PEPTIDE ANTIGEN**	140	0.35	1.49	0.0128	0.1147
**ORGANOPHOSPHATE CATABOLIC PROCESS**	102	0.37	1.49	0.0101	0.1150
**PHOSPHOLIPASE ACTIVITY**	88	0.37	1.49	0.0176	0.1150
**NON CANONICAL WNT SIGNALING PATHWAY**	136	0.36	1.49	0.0129	0.1152
**CATION SUGAR SYMPORTER ACTIVITY**	15	0.55	1.49	0.0554	0.1153
**GLYCOGEN BIOSYNTHETIC PROCESS**	16	0.54	1.49	0.0731	0.1154
**XENOPHAGY**	81	0.38	1.49	0.0233	0.1155
**POSITIVE REGULATION OF AUTOPHAGY**	65	0.40	1.49	0.0369	0.1161
**DNA INTEGRITY CHECKPOINT**	122	0.36	1.48	0.0101	0.1181
**PROTEIN ALKYLATION**	99	0.37	1.48	0.0117	0.1183
**3 5 EXONUCLEASE ACTIVITY**	44	0.43	1.48	0.0329	0.1190
**CONDENSED CHROMOSOME**	164	0.34	1.48	0.0056	0.1191
**INO80 TYPE COMPLEX**	18	0.52	1.48	0.0451	0.1192
**PHOSPHOLIPID DEPHOSPHORYLATION**	25	0.49	1.48	0.0604	0.1193
**LIPID TRANSPORTER ACTIVITY**	96	0.37	1.48	0.0201	0.1195
**CELLULAR ALDEHYDE METABOLIC PROCESS**	72	0.39	1.48	0.0211	0.1196
**NEGATIVE REGULATION OF MITOTIC NUCLEAR DIVISION**	28	0.47	1.48	0.0510	0.1197
**DICARBOXYLIC ACID TRANSPORT**	68	0.39	1.48	0.0262	0.1211
**CYTOPLASMIC PATTERN RECOGNITION RECEPTOR SIGNALING PATHWAY**	30	0.46	1.48	0.0376	0.1212
**ACETYL COA METABOLIC PROCESS**	25	0.49	1.48	0.0466	0.1213
**REGULATION OF VIRAL RELEASE FROM HOST CELL**	27	0.48	1.48	0.0509	0.1214
**THYROID HORMONE METABOLIC PROCESS**	16	0.54	1.48	0.0699	0.1220
**TRANSCRIPTIONALLY ACTIVE CHROMATIN**	18	0.52	1.48	0.0552	0.1227
**POSITIVE REGULATION OF ACTIN CYTOSKELETON REORGANIZATION**	15	0.53	1.48	0.0569	0.1228
**ESTROGEN METABOLIC PROCESS**	15	0.54	1.48	0.0635	0.1228
**PROTEIN LOCALIZATION TO CYTOSKELETON**	25	0.48	1.48	0.0450	0.1229
**PHAGOSOME MATURATION**	35	0.45	1.48	0.0417	0.1229
**RESPONSE TO ISCHEMIA**	24	0.49	1.48	0.0414	0.1233
**ESTROGEN RECEPTOR BINDING**	35	0.44	1.48	0.0448	0.1233
**ORGANELLE LOCALIZATION**	359	0.31	1.47	0.0000	0.1257
**MANNOSYLATION**	25	0.48	1.47	0.0509	0.1280

**FILOPODIUM ASSEMBLY**	16	-0.60	-1.75	0.0069	0.1280
**POSITIVE REGULATION OF HEART GROWTH**	24	0.48	1.47	0.0568	0.1281
**FAT CELL DIFFERENTIATION**	98	0.37	1.47	0.0165	0.1282
**CELLULAR KETONE METABOLIC PROCESS**	55	0.40	1.47	0.0385	0.1282
**MONOCARBOXYLIC ACID TRANSPORT**	110	0.36	1.47	0.0144	0.1282
**ANAPHASE PROMOTING COMPLEX**	18	0.53	1.47	0.0496	0.1282
**REACTIVE NITROGEN SPECIES METABOLIC PROCESS**	16	0.53	1.47	0.0713	0.1284
**HYDROGEN TRANSPORT**	115	0.35	1.47	0.0142	0.1286
**S ACYLTRANSFERASE ACTIVITY**	27	0.47	1.47	0.0491	0.1288
**CELL DIVISION**	399	0.31	1.47	0.0000	0.1288
**GLYCOLIPID BINDING**	18	0.51	1.47	0.0589	0.1289
**SINGLE ORGANISM MEMBRANE FUSION**	113	0.36	1.47	0.0086	0.1290
**GLYCOLIPID BIOSYNTHETIC PROCESS**	52	0.41	1.47	0.0403	0.1292
**REGULATION OF ALTERNATIVE MRNA SPLICING VIA SPLICEOSOME**	28	0.46	1.47	0.0529	0.1302
**INTRINSIC APOPTOTIC SIGNALING PATHWAY**	122	0.35	1.47	0.0126	0.1302
**LIPOSACCHARIDE METABOLIC PROCESS**	95	0.37	1.47	0.0198	0.1303
**NEUROPEPTIDE HORMONE ACTIVITY**	27	-0.53	-1.75	0.0074	0.1310
**VESICLE LOCALIZATION**	203	0.33	1.46	0.0040	0.1314
**REGULATION OF SULFUR METABOLIC PROCESS**	17	0.53	1.46	0.0747	0.1316
**REGULATION OF TRANSCRIPTION ELONGATION FROM RNA POLYMERASE II PROMOTER**	22	0.49	1.46	0.0489	0.1319
**POSITIVE REGULATION OF GROWTH**	213	0.33	1.46	0.0013	0.1323
**REGULATION OF T CELL MIGRATION**	20	-0.56	-1.74	0.0089	0.1330
**CYTOKINE ACTIVITY**	165	-0.36	-1.74	0.0000	0.1336
**REGULATION OF CHROMATIN BINDING**	15	0.54	1.46	0.0651	0.1336
**DELAYED RECTIFIER POTASSIUM CHANNEL ACTIVITY**	36	-0.50	-1.74	0.0070	0.1340
**NEGATIVE REGULATION OF RESPONSE TO REACTIVE OXYGEN SPECIES**	17	-0.59	-1.74	0.0069	0.1341
**GOLGI TO ENDOSOME TRANSPORT**	17	0.52	1.46	0.0646	0.1346
**REGULATION OF TRANSCRIPTION FROM RNA POLYMERASE I PROMOTER**	23	0.49	1.46	0.0526	0.1347
**REGULATION OF CELL PROLIFERATION INVOLVED IN HEART MORPHOGENESIS**	15	0.54	1.46	0.0655	0.1349
**TRANSCRIPTION FROM RNA POLYMERASE III PROMOTER**	36	0.44	1.46	0.0379	0.1352

**TRANSPORT VESICLE**	283	0.32	1.46	0.0012	0.1378
**NEGATIVE REGULATION OF NUCLEOCYTOPLASMIC TRANSPORT**	62	0.39	1.45	0.0294	0.1378
**POSITIVE REGULATION OF PEPTIDYL THREONINE PHOSPHORYLATION**	23	0.49	1.45	0.0561	0.1379
**CELLULAR RESPONSE TO REACTIVE NITROGEN SPECIES**	18	0.51	1.45	0.0757	0.1379
**AMMONIUM TRANSMEMBRANE TRANSPORTER ACTIVITY**	26	0.47	1.45	0.0559	0.1379
**AMINOPEPTIDASE ACTIVITY**	37	0.44	1.45	0.0460	0.1379
**ADRENERGIC RECEPTOR SIGNALING PATHWAY**	18	0.51	1.45	0.0757	0.1381
**NEGATIVE REGULATION OF LIPID STORAGE**	17	0.52	1.45	0.0829	0.1382
**REGULATION OF LIPOPROTEIN LIPASE ACTIVITY**	15	0.54	1.45	0.0761	0.1383
**REGULATION OF FATTY ACID METABOLIC PROCESS**	72	0.38	1.45	0.0280	0.1384
**REGULATION OF DENDRITE EXTENSION**	18	0.51	1.45	0.0636	0.1387
**PYRIMIDINE NUCLEOSIDE BIOSYNTHETIC PROCESS**	25	0.47	1.45	0.0567	0.1394
**MAGNESIUM ION BINDING**	172	0.34	1.45	0.0134	0.1406
**GABA RECEPTOR BINDING**	15	0.54	1.45	0.0726	0.1413
**NEGATIVE REGULATION OF CELLULAR PROTEIN CATABOLIC PROCESS**	57	0.39	1.45	0.0469	0.1416
**CYTOPLASMIC SEQUESTERING OF PROTEIN**	35	0.43	1.45	0.0510	0.1417
**REGULATION OF PROTEIN TARGETING**	267	0.32	1.45	0.0038	0.1418
**CHOLESTEROL EFFLUX**	26	0.47	1.45	0.0528	0.1418
**RESPONSE TO OXIDATIVE STRESS**	306	0.31	1.45	0.0025	0.1419
**OLIGOSACCHARIDE METABOLIC PROCESS**	57	0.39	1.45	0.0484	0.1420
**HISTONE METHYLTRANSFERASE COMPLEX**	60	0.39	1.45	0.0475	0.1421
**REGULATION OF PROTEIN COMPLEX ASSEMBLY**	322	0.31	1.45	0.0037	0.1423
**PURINE CONTAINING COMPOUND BIOSYNTHETIC PROCESS**	120	0.35	1.45	0.0201	0.1423
**SPHINGOLIPID METABOLIC PROCESS**	117	0.35	1.45	0.0215	0.1435
**NEGATIVE REGULATION OF ORGANELLE ORGANIZATION**	330	0.31	1.45	0.0012	0.1436
**PYRIMIDINE NUCLEOSIDE METABOLIC PROCESS**	39	0.43	1.44	0.0475	0.1447
**POLY PYRIMIDINE TRACT BINDING**	17	0.52	1.44	0.0668	0.1447

**ANTIGEN RECEPTOR MEDIATED SIGNALING PATHWAY**	148	0.34	1.44	0.0120	0.1451
**CELL CYCLE CHECKPOINT**	163	0.33	1.44	0.0195	0.1451
**PROTEIN KINASE C BINDING**	47	-0.45	-1.72	0.0000	0.1451
**LYASE ACTIVITY**	151	0.34	1.44	0.0148	0.1454
**STRESS ACTIVATED PROTEIN KINASE SIGNALING CASCADE**	89	0.37	1.44	0.0164	0.1454
**DNA DOUBLE STRAND BREAK PROCESSING**	17	0.53	1.44	0.0727	0.1456
**NEGATIVE REGULATION OF CELL CYCLE PROCESS**	181	0.33	1.44	0.0121	0.1456
**GOLGI CISTERNA**	75	0.38	1.44	0.0501	0.1459
**UDP GLYCOSYLTRANSFERASE ACTIVITY**	105	0.36	1.44	0.0271	0.1460
**NUCLEAR TRANSCRIPTION FACTOR COMPLEX**	112	0.35	1.44	0.0236	0.1461
**INTRINSIC APOPTOTIC SIGNALING PATHWAY BY P53 CLASS MEDIATOR**	42	0.42	1.44	0.0463	0.1462
**MYELIN SHEATH**	148	0.34	1.44	0.0127	0.1462
**NEGATIVE REGULATION OF VASCULATURE DEVELOPMENT**	75	-0.41	-1.72	0.0060	0.1465
**CELLULAR MONOVALENT INORGANIC CATION HOMEOSTASIS**	82	0.37	1.44	0.0198	0.1465
**REGULATION OF COENZYME METABOLIC PROCESS**	45	0.41	1.44	0.0533	0.1466
**POSITIVE REGULATION OF ACUTE INFLAMMATORY RESPONSE**	27	-0.52	-1.72	0.0098	0.1475
**RESPONSE TO ARSENIC CONTAINING SUBSTANCE**	29	0.46	1.44	0.0583	0.1476
**NEGATIVE REGULATION OF LYMPHOCYTE APOPTOTIC PROCESS**	24	-0.52	-1.72	0.0101	0.1484
**POSITIVE REGULATION OF COAGULATION**	24	0.46	1.44	0.0561	0.1490
**LYTIC VACUOLE**	436	0.30	1.44	0.0000	0.1490
**VESICLE MEMBRANE**	450	0.30	1.44	0.0000	0.1493
**PROTEIN METHYLTRANSFERASE ACTIVITY**	68	0.38	1.44	0.0463	0.1495
**ER TO GOLGI TRANSPORT VESICLE**	46	0.41	1.44	0.0413	0.1495
**PYRIMIDINE NUCLEOSIDE TRIPHOSPHATE BIOSYNTHETIC PROCESS**	15	0.53	1.44	0.0734	0.1496
**HISTONE H4 ACETYLATION**	40	0.42	1.44	0.0417	0.1498
**NEUROPEPTIDE RECEPTOR ACTIVITY**	42	-0.46	-1.72	0.0052	0.1499
**SERINE FAMILY AMINO ACID METABOLIC PROCESS**	37	0.43	1.43	0.0495	0.1514

**TRANSFERASE ACTIVITY TRANSFERRING GLYCOSYL GROUPS**	216	0.32	1.43	0.0080	0.1526
**PCG PROTEIN COMPLEX**	39	0.42	1.43	0.0621	0.1528
**CELLULAR RESPONSE TO UV**	54	0.40	1.43	0.0427	0.1533
**REGULATION OF GLUCONEOGENESIS**	34	0.44	1.43	0.0507	0.1536
**POSITIVE REGULATION OF DENDRITE EXTENSION**	18	0.51	1.43	0.0819	0.1538
**PHOSPHORIC DIESTER HYDROLASE ACTIVITY**	86	0.36	1.43	0.0176	0.1551
**PROTEIN LOCALIZATION TO GOLGI APPARATUS**	23	0.48	1.43	0.0675	0.1562
**CYTOPLASMIC SEQUESTERING OF TRANSCRIPTION FACTOR**	15	0.54	1.43	0.0833	0.1568
**REGULATION OF VACUOLE ORGANIZATION**	37	0.42	1.43	0.0597	0.1572
**TRANSCRIPTION FACTOR BINDING**	457	0.30	1.43	0.0011	0.1573
**NEGATIVE REGULATION OF LIPID TRANSPORT**	25	0.46	1.43	0.0699	0.1575
**NEGATIVE REGULATION OF RESPONSE TO NUTRIENT LEVELS**	30	0.44	1.43	0.0658	0.1575
**PROTEIN DISULFIDE OXIDOREDUCTASE ACTIVITY**	22	0.49	1.43	0.0720	0.1576
**MODIFICATION BY SYMBIONT OF HOST MORPHOLOGY OR PHYSIOLOGY**	38	0.42	1.43	0.0529	0.1576
**POSITIVE REGULATION OF BLOOD PRESSURE**	34	0.44	1.43	0.0761	0.1577
**APOPTOTIC SIGNALING PATHWAY**	242	0.32	1.43	0.0104	0.1577
**REGULATION OF CELLULAR PROTEIN LOCALIZATION**	479	0.30	1.42	0.0000	0.1581
**DRUG TRANSMEMBRANE TRANSPORT**	17	0.52	1.42	0.0688	0.1586
**IMPORT INTO CELL**	34	0.43	1.42	0.0600	0.1589
**PHOSPHATIDYLINOSITOL METABOLIC PROCESS**	176	0.33	1.42	0.0154	0.1591
**POLYSACCHARIDE CATABOLIC PROCESS**	20	0.49	1.42	0.0860	0.1595
**CYTOSOLIC RIBOSOME**	85	0.36	1.42	0.0432	0.1598
**CELLULAR PIGMENTATION**	41	0.42	1.42	0.0613	0.1599
**DOUBLE STRAND BREAK REPAIR**	124	0.34	1.42	0.0247	0.1599
**CELL CYCLE PHASE TRANSITION**	213	0.32	1.42	0.0078	0.1600
**POSITIVE REGULATION OF WOUND HEALING**	42	0.42	1.42	0.0604	0.1608
**AGGRESOME**	29	0.44	1.42	0.0670	0.1621
**NEGATIVE REGULATION OF RESPONSE TO CYTOKINE STIMULUS**	36	0.43	1.42	0.0548	0.1622

**MAINTENANCE OF PROTEIN LOCALIZATION IN ORGANELLE**	28	0.45	1.42	0.0592	0.1627
**ORGANIC HYDROXY COMPOUND TRANSPORT**	139	0.34	1.42	0.0156	0.1628
**PROTEIN EXPORT FROM NUCLEUS**	29	0.45	1.42	0.0546	0.1634
**DNA REPLICATION**	166	0.33	1.42	0.0080	0.1639
**MEMBRANE LIPID BIOSYNTHETIC PROCESS**	96	0.35	1.42	0.0330	0.1639
**PALMITOYLTRANSFERASE ACTIVITY**	31	0.43	1.42	0.0723	0.1641
**POLYUBIQUITIN BINDING**	36	0.42	1.42	0.0710	0.1647
**REGULATION OF BLOOD VESSEL ENDOTHELIAL CELL MIGRATION**	49	-0.44	-1.70	0.0132	0.1655
**MAINTENANCE OF LOCATION IN CELL**	84	0.36	1.41	0.0352	0.1662
**JNK CASCADE**	72	0.37	1.41	0.0490	0.1669
**PROTEIN HYDROXYLATION**	18	-0.56	-1.70	0.0128	0.1675
**REGULATION OF ISOTYPE SWITCHING**	20	-0.54	-1.70	0.0100	0.1689
**NUCLEAR CHROMOSOME TELOMERIC REGION**	95	0.35	1.41	0.0389	0.1702
**DNA DEPENDENT DNA REPLICATION**	77	0.36	1.41	0.0438	0.1703
**REGULATION OF ESTABLISHMENT OF PROTEIN LOCALIZATION TO MITOCHONDRION**	110	0.35	1.41	0.0345	0.1703
**REGULATION OF INTERLEUKIN 10 PRODUCTION**	37	-0.46	-1.69	0.0076	0.1704
**U12 TYPE SPLICEOSOMAL COMPLEX**	23	0.47	1.41	0.0730	0.1704
**MULTICELLULAR ORGANISM GROWTH**	67	0.38	1.41	0.0339	0.1705
**NEGATIVE REGULATION OF CANONICAL WNT SIGNALING PATHWAY**	152	0.34	1.41	0.0204	0.1709
**REGULATION OF COFACTOR METABOLIC PROCESS**	45	0.41	1.41	0.0753	0.1710
**POSITIVE REGULATION OF CELL CYCLE G1 S PHASE TRANSITION**	25	0.46	1.41	0.0767	0.1711
**REGULATION OF LIPID METABOLIC PROCESS**	245	0.31	1.41	0.0087	0.1711
**NEGATIVE REGULATION OF LIPID CATABOLIC PROCESS**	18	0.50	1.41	0.0863	0.1713
**REGULATION OF MEMBRANE PROTEIN ECTODOMAIN PROTEOLYSIS**	20	-0.54	-1.69	0.0070	0.1715
**POSITIVE REGULATION OF INTERLEUKIN 1 BETA PRODUCTION**	21	-0.53	-1.69	0.0263	0.1718
**CENTROSOME**	408	0.30	1.41	0.0000	0.1729
**NEGATIVE REGULATION OF KIDNEY DEVELOPMENT**	17	-0.57	-1.69	0.0113	0.1730

**EXTRACELLULAR MATRIX STRUCTURAL CONSTITUENT**	67	-0.41	-1.68	0.0057	0.1737
**NEGATIVE REGULATION OF CELLULAR AMIDE METABOLIC PROCESS**	119	0.34	1.41	0.0262	0.1737
**NON RECOMBINATIONAL REPAIR**	53	0.40	1.40	0.0437	0.1742
**DEMETHYLASE ACTIVITY**	31	0.43	1.40	0.0815	0.1744
**REGULATION OF LEUKOCYTE APOPTOTIC PROCESS**	66	-0.41	-1.68	0.0057	0.1756
**CHROMOSOMAL REGION**	264	0.31	1.40	0.0102	0.1759
**MYELOID LEUKOCYTE MIGRATION**	79	-0.39	-1.68	0.0000	0.1759
**DETECTION OF MECHANICAL STIMULUS INVOLVED IN SENSORY PERCEPTION**	22	-0.53	-1.68	0.0113	0.1763
**POSITIVE REGULATION OF SYNAPSE ASSEMBLY**	55	0.39	1.40	0.0554	0.1765
**REGULATION OF TRANSLATION IN RESPONSE TO STRESS**	19	0.49	1.40	0.1076	0.1775
**RESPONSE TO HYDROGEN PEROXIDE**	92	0.36	1.40	0.0421	0.1777
**POSITIVE REGULATION OF I KAPPAB KINASE NF KAPPAB SIGNALING**	157	0.32	1.40	0.0254	0.1782
**ESTABLISHMENT OF PROTEIN LOCALIZATION TO ENDOPLASMIC RETICULUM**	85	0.36	1.40	0.0460	0.1784
**SERINE TYPE EXOPEPTIDASE ACTIVITY**	17	0.50	1.40	0.1164	0.1793
**SULFUR AMINO ACID METABOLIC PROCESS**	39	0.42	1.40	0.0715	0.1796
**NUCLEOSOMAL DNA BINDING**	27	0.44	1.40	0.0829	0.1797
**PROTEIN STABILIZATION**	120	0.34	1.40	0.0258	0.1799
**PROTEIN HOMOTETRAMERIZATION**	55	0.38	1.40	0.0696	0.1799
**REGULATION OF INTRACELLULAR PROTEIN TRANSPORT**	332	0.30	1.40	0.0085	0.1800
**POSITIVE REGULATION OF CARDIAC MUSCLE TISSUE DEVELOPMENT**	26	0.45	1.40	0.0876	0.1800
**ANTIGEN PROCESSING AND PRESENTATION**	161	0.33	1.40	0.0189	0.1803
**REGULATION OF CARBOHYDRATE METABOLIC PROCESS**	149	0.33	1.40	0.0262	0.1804
**DOUBLE STRANDED RNA BINDING**	54	0.38	1.40	0.0670	0.1812
**RIBONUCLEOPROTEIN GRANULE**	132	0.33	1.40	0.0323	0.1812
**U2 TYPE SPLICEOSOMAL COMPLEX**	25	0.46	1.39	0.0807	0.1819
**REGULATION OF VIRAL TRANSCRIPTION**	56	0.39	1.39	0.0512	0.1819
**MUSCLE CELL CELLULAR HOMEOSTASIS**	18	0.49	1.39	0.0927	0.1827
**PROTEIN LOCALIZATION TO CHROMOSOME**	41	0.41	1.39	0.0564	0.1828

**FATTY ACID BINDING**	27	0.44	1.39	0.0840	0.1828
**HISTONE UBIQUITINATION**	28	0.45	1.39	0.0666	0.1829
**CHROMOSOME TELOMERIC REGION**	123	0.34	1.39	0.0281	0.1838
**VOLTAGE GATED ION CHANNEL ACTIVITY**	177	-0.35	-1.67	0.0000	0.1840
**NUCLEAR NUCLEOSOME**	15	-0.59	-1.67	0.0214	0.1840
**REGULATION OF SYNAPTIC VESICLE TRANSPORT**	29	0.43	1.39	0.0848	0.1845
**NEGATIVE REGULATION OF RESPONSE TO EXTRACELLULAR STIMULUS**	30	0.44	1.39	0.0786	0.1847
**KINETOCHORE**	97	0.35	1.39	0.0419	0.1855
**PHOSPHATIDYLINOSITOL 3 KINASE COMPLEX**	17	0.51	1.39	0.0979	0.1863
**POSITIVE REGULATION OF RESPONSE TO DNA DAMAGE STIMULUS**	54	0.38	1.39	0.0661	0.1863
**PYRIMIDINE NUCLEOSIDE TRIPHOSPHATE METABOLIC PROCESS**	17	0.51	1.39	0.0810	0.1865
**REGULATION OF MULTI ORGANISM PROCESS**	391	0.29	1.39	0.0047	0.1867
**S ADENOSYLMETHIONINE METABOLIC PROCESS**	18	0.50	1.39	0.0810	0.1867
**MANNOSYLTRANSFERASE ACTIVITY**	18	0.49	1.39	0.1082	0.1871
**GENE SILENCING BY RNA**	108	0.34	1.39	0.0398	0.1873
**ORGANIC CATION TRANSPORT**	19	0.48	1.39	0.0909	0.1873
**CORTICAL CYTOSKELETON**	65	0.37	1.39	0.0631	0.1886
**CELLULAR MODIFIED AMINO ACID BIOSYNTHETIC PROCESS**	46	0.40	1.39	0.0522	0.1890
**TRANSLATION REGULATOR ACTIVITY**	30	0.43	1.39	0.0890	0.1891
**DRUG TRANSPORT**	22	0.47	1.38	0.0889	0.1898
**DRUG TRANSPORTER ACTIVITY**	18	0.49	1.38	0.1009	0.1898
**LEUKOCYTE MIGRATION**	223	-0.33	-1.65	0.0000	0.1905
**REGULATION OF ALPHA BETA T CELL DIFFERENTIATION**	40	-0.46	-1.66	0.0079	0.1906
**INTERMEDIATE FILAMENT**	124	-0.35	-1.66	0.0000	0.1911
**NEGATIVE REGULATION OF T CELL DIFFERENTIATION**	27	-0.50	-1.66	0.0075	0.1912
**REGULATION OF MONOCYTE CHEMOTAXIS**	18	-0.55	-1.66	0.0092	0.1918
**CHROMOSOME SEGREGATION**	225	0.31	1.38	0.0130	0.1929
**CARTILAGE CONDENSATION**	20	-0.53	-1.66	0.0162	0.1933
**REGULATION OF LIPID TRANSPORT**	87	0.35	1.38	0.0496	0.1936
**PYRIMIDINE CONTAINING COMPOUND METABOLIC PROCESS**	61	0.37	1.38	0.0635	0.1936

**GLUTAMINE FAMILY AMINO ACID METABOLIC PROCESS**	60	0.37	1.38	0.0581	0.1938
**GTPASE ACTIVITY**	206	0.32	1.38	0.0146	0.1942
**PRE MRNA BINDING**	21	0.47	1.38	0.0930	0.1944
**POSITIVE REGULATION OF SODIUM ION TRANSPORT**	30	0.44	1.38	0.0873	0.1944
**MITOCHONDRION MORPHOGENESIS**	17	0.50	1.38	0.1014	0.1944
**AMMONIUM TRANSMEMBRANE TRANSPORT**	22	0.47	1.38	0.1045	0.1946
**FATTY ACID BIOSYNTHETIC PROCESS**	92	0.35	1.38	0.0477	0.1947
**HYDROLASE ACTIVITY ACTING ON CARBON NITROGEN BUT NOT PEPTIDE BONDS**	123	0.33	1.38	0.0371	0.1947
**TRANSITION METAL ION TRANSPORT**	96	0.35	1.38	0.0524	0.1947
**REGULATION OF DNA REPAIR**	67	0.37	1.38	0.0552	0.1949
**CYTOPLASMIC MICROTUBULE**	43	0.40	1.38	0.0832	0.1955
**NEGATIVE REGULATION OF LIPASE ACTIVITY**	15	0.51	1.38	0.1103	0.1955
**CYTOSKELETON DEPENDENT CYTOKINESIS**	32	0.42	1.38	0.0835	0.1958
**PHOSPHOLIPASE BINDING**	18	0.48	1.38	0.0964	0.1960
**NAD METABOLIC PROCESS**	51	0.38	1.38	0.0598	0.1962
**PROTEIN LOCALIZATION TO LYSOSOME**	16	0.50	1.38	0.1020	0.1963
**RIBONUCLEOTIDE CATABOLIC PROCESS**	26	0.44	1.38	0.1048	0.1963
**CELLULAR RESPONSE TO ESTROGEN STIMULUS**	37	0.41	1.38	0.0846	0.1965
**PROTEIN K48 LINKED DEUBIQUITINATION**	17	0.51	1.38	0.0973	0.1969
**CHROMOSOME LOCALIZATION**	49	0.39	1.37	0.0604	0.1978
**DNA DEPENDENT ATPASE ACTIVITY**	70	0.37	1.37	0.0465	0.2009
**INTESTINAL EPITHELIAL CELL DIFFERENTIATION**	15	0.50	1.37	0.1200	0.2018
**PURINERGIC RECEPTOR SIGNALING PATHWAY**	26	0.45	1.37	0.0953	0.2019
**NUCLEAR IMPORT**	112	0.34	1.37	0.0313	0.2020
**NUCLEAR EUCHROMATIN**	20	0.47	1.37	0.0855	0.2025
**CHROMATIN SILENCING**	60	-0.41	-1.65	0.0000	0.2028
**REGULATION OF ALPHA BETA T CELL PROLIFERATION**	17	-0.55	-1.64	0.0256	0.2029
**INSULIN RECEPTOR SIGNALING PATHWAY**	72	0.36	1.37	0.0595	0.2032
**MODIFICATION OF MORPHOLOGY OR PHYSIOLOGY OF OTHER ORGANISM**	71	0.37	1.37	0.0646	0.2035
**COENZYME A METABOLIC PROCESS**	17	0.49	1.37	0.1138	0.2039

**G PROTEIN COUPLED CHEMOATTRACTANT RECEPTOR ACTIVITY**	23	-0.51	-1.64	0.0143	0.2041
**MITOTIC CYTOKINESIS**	25	0.45	1.37	0.0959	0.2044
**GUANYL NUCLEOTIDE BINDING**	332	0.30	1.37	0.0074	0.2044
**REGULATION OF CELLULAR AMINE METABOLIC PROCESS**	79	0.35	1.37	0.0574	0.2056
**HYDROGEN ION TRANSMEMBRANE TRANSPORT**	86	0.35	1.37	0.0652	0.2056
**MONOCYTE CHEMOTAXIS**	27	-0.48	-1.62	0.0177	0.2056
**MEMBRANE PROTEIN ECTODOMAIN PROTEOLYSIS**	17	0.49	1.37	0.1020	0.2058
**CYTOPLASMIC MRNA PROCESSING BODY**	63	0.37	1.37	0.0470	0.2060
**INTRAMOLECULAR OXIDOREDUCTASE ACTIVITY**	48	0.39	1.37	0.0770	0.2064
**ADAPTIVE IMMUNE RESPONSE BASED ON SOMATIC RECOMBINATION OF IMMUNE RECEPTORS BUILT FROM IMMUNOGLOBULIN SUPERFAMILY DOMAINS**	97	-0.36	-1.62	0.0000	0.2067
**REGULATION OF LEUKOCYTE MIGRATION**	132	-0.35	-1.63	0.0000	0.2068
**CARDIAC RIGHT VENTRICLE MORPHOGENESIS**	15	-0.58	-1.62	0.0313	0.2073
**WNT ACTIVATED RECEPTOR ACTIVITY**	21	0.46	1.36	0.0926	0.2074
**NEGATIVE REGULATION OF LYMPHOCYTE DIFFERENTIATION**	34	-0.45	-1.63	0.0193	0.2075
**AMIDE TRANSMEMBRANE TRANSPORTER ACTIVITY**	17	-0.56	-1.63	0.0147	0.2081
**RESPONSE TO PROTOZOAN**	18	-0.54	-1.62	0.0267	0.2088
**PYRIMIDINE NUCLEOSIDE CATABOLIC PROCESS**	15	0.50	1.36	0.1243	0.2090
**PROXIMAL DISTAL PATTERN FORMATION**	31	-0.48	-1.63	0.0025	0.2096
**CELL AGGREGATION**	20	-0.53	-1.63	0.0238	0.2097
**TRANSFERASE ACTIVITY TRANSFERRING SULFUR CONTAINING GROUPS**	56	0.37	1.36	0.0761	0.2098
**NEGATIVE REGULATION OF TELOMERE MAINTENANCE**	23	0.45	1.36	0.0947	0.2100
**AMINO ACID TRANSPORT**	114	0.33	1.36	0.0394	0.2100
**NUCLEOBASE CONTAINING COMPOUND KINASE ACTIVITY**	42	0.39	1.36	0.0862	0.2101

**REGULATION OF SYMBIOSIS ENCOMPASSING MUTUALISM THROUGH PARASITISM**	171	0.32	1.36	0.0284	0.2102
**REGULATION OF NUCLEASE ACTIVITY**	22	-0.52	-1.64	0.0158	0.2107
**POSITIVE REGULATION OF TRANSLATIONAL INITIATION**	20	0.47	1.36	0.1043	0.2108
**SPERM CAPACITATION**	16	0.49	1.36	0.1248	0.2108
**NUCLEOSIDE DIPHOSPHATE METABOLIC PROCESS**	73	0.35	1.36	0.0712	0.2109
**THYMIC T CELL SELECTION**	19	-0.53	-1.63	0.0389	0.2110
**KIDNEY MESENCHYME DEVELOPMENT**	18	-0.54	-1.63	0.0194	0.2116
**INACTIVATION OF MAPK ACTIVITY**	26	0.44	1.36	0.1123	0.2119
**TRANSLATION REPRESSOR ACTIVITY**	17	0.49	1.36	0.1052	0.2119
**REGULATION OF B CELL APOPTOTIC PROCESS**	17	-0.56	-1.63	0.0137	0.2121
**AMINE METABOLIC PROCESS**	120	0.33	1.36	0.0424	0.2122
**DNA DIRECTED RNA POLYMERASE III COMPLEX**	18	0.48	1.36	0.1197	0.2128
**SWI SNF SUPERFAMILY TYPE COMPLEX**	64	0.36	1.36	0.0615	0.2138
**REGULATION OF TUMOR NECROSIS FACTOR BIOSYNTHETIC PROCESS**	16	-0.55	-1.61	0.0494	0.2141
**NEGATIVE REGULATION OF CYCLIC NUCLEOTIDE METABOLIC PROCESS**	40	-0.44	-1.62	0.0078	0.2142
**REGULATION OF RESPONSE TO DNA DAMAGE STIMULUS**	129	0.33	1.36	0.0414	0.2143
**SPINDLE POLE**	107	0.33	1.36	0.0389	0.2150
**POSITIVE REGULATION OF CELLULAR COMPONENT BIOGENESIS**	359	0.29	1.36	0.0083	0.2150
**ANION TRANSMEMBRANE TRANSPORTER ACTIVITY**	258	0.30	1.36	0.0266	0.2152
**REGULATION OF MICROTUBULE POLYMERIZATION OR DEPOLYMERIZATION**	149	0.32	1.36	0.0341	0.2153
**SINGLE STRANDED RNA BINDING**	59	0.37	1.36	0.0826	0.2154
**PROTEIN OLIGOMERIZATION**	379	0.29	1.36	0.0095	0.2155
**POSITIVE REGULATION OF TRANSCRIPTION INITIATION FROM RNA POLYMERASE II PROMOTER**	17	0.49	1.36	0.1135	0.2155
**MAP KINASE KINASE KINASE ACTIVITY**	17	0.48	1.35	0.1222	0.2157
**REGULATION OF MRNA METABOLIC PROCESS**	100	0.34	1.35	0.0494	0.2158
**HISTONE BINDING**	132	0.32	1.35	0.0401	0.2158
**TERMINAL BOUTON**	62	0.37	1.35	0.0740	0.2158

**CELLULAR PROTEIN COMPLEX LOCALIZATION**	18	0.49	1.35	0.1224	0.2169
**POSITIVE REGULATION OF MITOTIC CELL CYCLE**	110	0.34	1.35	0.0466	0.2171
**QUATERNARY AMMONIUM GROUP TRANSPORT**	16	0.49	1.35	0.1322	0.2175
**ORGANIC HYDROXY COMPOUND CATABOLIC PROCESS**	59	0.37	1.35	0.0783	0.2180
**NEGATIVE REGULATION OF MUSCLE CONTRACTION**	22	0.46	1.35	0.1139	0.2181
**IN UTERO EMBRYONIC DEVELOPMENT**	280	0.30	1.35	0.0120	0.2186
**SOMATIC RECOMBINATION OF IMMUNOGLOBULIN GENE SEGMENTS**	17	0.48	1.35	0.1261	0.2197
**CELL CYCLE G1 S PHASE TRANSITION**	93	0.34	1.35	0.0606	0.2198
**ORGANIC HYDROXY COMPOUND METABOLIC PROCESS**	419	0.29	1.35	0.0083	0.2200
**CELL GROWTH**	120	0.33	1.35	0.0416	0.2201
**LYSOPHOSPHOLIPASE ACTIVITY**	17	0.48	1.35	0.1290	0.2207
**MITOPHAGY IN RESPONSE TO MITOCHONDRIAL DEPOLARIZATION**	109	0.33	1.35	0.0440	0.2211
**MEMBRANE DOCKING**	59	0.37	1.35	0.0768	0.2223
**ANTIOXIDANT ACTIVITY**	59	0.37	1.35	0.0744	0.2224
**AUTOPHAGOSOME**	64	0.36	1.35	0.0788	0.2226
**REGULATION OF GLUCOSE METABOLIC PROCESS**	96	0.34	1.35	0.0590	0.2227
**REGULATION OF RETINOIC ACID RECEPTOR SIGNALING PATHWAY**	15	-0.56	-1.59	0.0300	0.2232
**REGULATION OF MEMBRANE PERMEABILITY**	55	0.37	1.35	0.0814	0.2232
**LONG TERM SYNAPTIC POTENTIATION**	37	-0.45	-1.60	0.0080	0.2234
**POSITIVE REGULATION OF NEURAL PRECURSOR CELL PROLIFERATION**	39	-0.43	-1.60	0.0294	0.2236
**VITAMIN METABOLIC PROCESS**	100	0.33	1.35	0.0495	0.2239
**NEGATIVE REGULATION OF CYTOKINE PRODUCTION INVOLVED IN IMMUNE RESPONSE**	17	-0.53	-1.60	0.0267	0.2245
**ANION TRANSMEMBRANE TRANSPORT**	225	0.30	1.35	0.0159	0.2245
**T CELL SELECTION**	32	-0.45	-1.61	0.0132	0.2251
**REGULATION OF SYSTEMIC ARTERIAL BLOOD PRESSURE BY HORMONE**	32	-0.45	-1.59	0.0176	0.2251
**REGULATION OF IMMUNOGLOBULIN PRODUCTION**	39	-0.43	-1.60	0.0148	0.2252
**LEFT RIGHT PATTERN FORMATION**	19	-0.52	-1.60	0.0276	0.2253

**TAXIS**	394	-0.30	-1.60	0.0000	0.2254
**NEUROPEPTIDE SIGNALING PATHWAY**	90	-0.37	-1.59	0.0000	0.2263
**PROTEIN TETRAMERIZATION**	106	0.33	1.34	0.0552	0.2268
**POTASSIUM CHANNEL ACTIVITY**	115	-0.35	-1.60	0.0000	0.2272
**RESPONSE TO VIRUS**	196	-0.32	-1.59	0.0000	0.2273
**RESPONSE TO XENOBIOTIC STIMULUS**	67	0.35	1.34	0.0691	0.2276
**RESPONSE TO MITOCHONDRIAL DEPOLARISATION**	109	0.33	1.34	0.0534	0.2276
**POSITIVE REGULATION OF POTASSIUM ION TRANSPORT**	34	0.41	1.34	0.0880	0.2277
**REGULATION OF CYTOPLASMIC TRANSPORT**	430	0.28	1.34	0.0106	0.2277
**POSITIVE REGULATION OF PROTEIN COMPLEX ASSEMBLY**	172	0.31	1.34	0.0360	0.2277
**HYDROGEN ION TRANSMEMBRANE TRANSPORTER ACTIVITY**	83	0.35	1.34	0.0620	0.2277
**ORGAN REGENERATION**	78	0.35	1.34	0.0729	0.2278
**RNA POLYMERASE III ACTIVITY**	18	0.48	1.34	0.1190	0.2283
**COLLAGEN FIBRIL ORGANIZATION**	35	-0.44	-1.60	0.0243	0.2287
**REGULATION OF NUCLEAR DIVISION**	141	0.32	1.34	0.0389	0.2287
**NEURON MATURATION**	27	-0.49	-1.59	0.0273	0.2291
**GRANULOCYTE MIGRATION**	60	-0.40	-1.58	0.0027	0.2308
**REGULATION OF FATTY ACID BIOSYNTHETIC PROCESS**	33	-0.45	-1.58	0.0127	0.2310
**NUCLEAR PERIPHERY**	103	0.33	1.34	0.0627	0.2316
**INTRINSIC APOPTOTIC SIGNALING PATHWAY IN RESPONSE TO DNA DAMAGE BY P53 CLASS MEDIATOR**	24	0.44	1.34	0.1086	0.2317
**B CELL HOMEOSTASIS**	19	0.46	1.34	0.1227	0.2319
**PROTEIN LOCALIZATION TO CILIUM**	22	0.46	1.34	0.1138	0.2320
**DEVELOPMENTAL PIGMENTATION**	36	0.41	1.34	0.0949	0.2329
**PROTEIN C TERMINUS BINDING**	169	0.31	1.34	0.0409	0.2339
**POSITIVE REGULATION OF CYTOKINE BIOSYNTHETIC PROCESS**	55	-0.39	-1.58	0.0110	0.2343
**REGULATION OF MYELINATION**	29	0.42	1.34	0.1150	0.2345
**POSITIVE REGULATION OF GLYCOGEN METABOLIC PROCESS**	15	-0.54	-1.57	0.0446	0.2347
**G PROTEIN COUPLED RECEPTOR ACTIVITY**	374	-0.29	-1.57	0.0000	0.2357
**REGULATION OF HYDROGEN PEROXIDE INDUCED CELL DEATH**	16	-0.54	-1.58	0.0305	0.2359
**NEUROPEPTIDE BINDING**	20	-0.51	-1.57	0.0501	0.2360

**IMMUNE EFFECTOR PROCESS**	356	-0.30	-1.57	0.0000	0.2363
**PARTURITION**	19	-0.52	-1.57	0.0315	0.2367
**G1 S TRANSITION OF MITOTIC CELL CYCLE**	93	0.34	1.33	0.0751	0.2379
**ALPHA TUBULIN BINDING**	22	0.46	1.33	0.1321	0.2381
**RESPONSE TO TOXIC SUBSTANCE**	214	0.30	1.33	0.0296	0.2382
**CELL REDOX HOMEOSTASIS**	60	0.36	1.33	0.0731	0.2383
**REGULATION OF B CELL MEDIATED IMMUNITY**	33	-0.45	-1.57	0.0129	0.2384
**NEURON FATE SPECIFICATION**	30	-0.47	-1.58	0.0102	0.2387
**SULFOTRANSFERASE ACTIVITY**	44	0.39	1.33	0.0918	0.2391
**REGULATION OF TRANSCRIPTION REGULATORY REGION DNA BINDING**	33	0.40	1.33	0.1084	0.2392
**CORTICAL ACTIN CYTOSKELETON**	46	0.38	1.33	0.0926	0.2393
**STEROID BINDING**	83	0.34	1.33	0.0714	0.2395
**CELLULAR AMINO ACID BIOSYNTHETIC PROCESS**	84	0.34	1.33	0.0701	0.2405
**REGULATION OF B CELL PROLIFERATION**	52	-0.39	-1.57	0.0164	0.2405
**NLS BEARING PROTEIN IMPORT INTO NUCLEUS**	21	0.45	1.33	0.1376	0.2411
**CARBOHYDRATE DERIVATIVE CATABOLIC PROCESS**	151	0.31	1.33	0.0474	0.2416
**PHOSPHOPROTEIN BINDING**	57	0.36	1.33	0.0836	0.2417
**REGULATION OF INFLAMMATORY RESPONSE TO ANTIGENIC STIMULUS**	17	-0.53	-1.57	0.0410	0.2417
**SPLICEOSOMAL COMPLEX ASSEMBLY**	35	0.41	1.33	0.1152	0.2420
**NEGATIVE REGULATION OF PRODUCTION OF MOLECULAR MEDIATOR OF IMMUNE RESPONSE**	23	-0.48	-1.56	0.0287	0.2423
**ETHANOLAMINE CONTAINING COMPOUND METABOLIC PROCESS**	78	0.34	1.33	0.0779	0.2424
**NEGATIVE REGULATION OF CYTOPLASMIC TRANSPORT**	103	0.33	1.33	0.0480	0.2424
**REGULATION OF STRIATED MUSCLE CONTRACTION**	76	0.35	1.33	0.0800	0.2431
**PHOSPHATIDYLINOSITOL BINDING**	186	0.30	1.33	0.0404	0.2432
**REGULATION OF CARDIAC MUSCLE CELL CONTRACTION**	27	0.43	1.33	0.1100	0.2433
**NUCLEAR CHROMOSOME**	413	0.28	1.33	0.0142	0.2440
**BETA CATENIN BINDING**	80	0.34	1.33	0.0654	0.2441
**COMPLEX OF COLLAGEN TRIMERS**	22	-0.49	-1.56	0.0323	0.2441
**MAST CELL GRANULE**	19	-0.50	-1.56	0.0316	0.2444
**RECEPTOR AGONIST ACTIVITY**	16	-0.54	-1.56	0.0547	0.2446

**ALCOHOL CATABOLIC PROCESS**	52	0.37	1.33	0.0866	0.2448
**VESICLE DOCKING**	49	0.37	1.33	0.0758	0.2449
**ACTIVE TRANSMEMBRANE TRANSPORTER ACTIVITY**	318	0.29	1.32	0.0255	0.2455
**PROTEIN HOMOOLIGOMERIZATION**	235	0.30	1.32	0.0341	0.2455
**REGULATION OF PROTEIN COMPLEX DISASSEMBLY**	179	0.31	1.32	0.0326	0.2456
**REGULATION OF INTERLEUKIN 2 BIOSYNTHETIC PROCESS**	16	-0.54	-1.56	0.0342	0.2456
**CELL CYCLE G2 M PHASE TRANSITION**	115	0.32	1.32	0.0629	0.2456
**SISTER CHROMATID SEGREGATION**	150	0.31	1.32	0.0455	0.2457
**WNT SIGNALING PATHWAY**	329	0.29	1.32	0.0134	0.2457
**REGULATION OF RESPONSE TO FOOD**	16	0.49	1.32	0.1456	0.2457
**REGULATION OF MITOCHONDRIAL OUTER MEMBRANE PERMEABILIZATION INVOLVED IN APOPTOTIC SIGNALING PATHWAY**	33	0.41	1.32	0.1113	0.2460
**REGULATION OF BINDING**	250	0.29	1.32	0.0287	0.2465
**POSITIVE REGULATION OF INSULIN SECRETION INVOLVED IN CELLULAR RESPONSE TO GLUCOSE STIMULUS**	23	0.45	1.32	0.1327	0.2466
**MODULATION BY VIRUS OF HOST MORPHOLOGY OR PHYSIOLOGY**	30	0.42	1.32	0.1269	0.2474
**ACETYLGLUCOSAMINYLTRANSFERASE ACTIVITY**	43	0.39	1.32	0.1100	0.2487
**RESPONSE TO REACTIVE OXYGEN SPECIES**	164	0.30	1.32	0.0505	0.2488
**DNA DAMAGE RESPONSE DETECTION OF DNA DAMAGE**	33	0.41	1.32	0.1045	0.2488
**PYRUVATE METABOLIC PROCESS**	57	0.36	1.32	0.1019	0.2488
**POSITIVE REGULATION OF NEURON PROJECTION DEVELOPMENT**	218	0.30	1.32	0.0326	0.2489
**INTRINSIC COMPONENT OF EXTERNAL SIDE OF PLASMA MEMBRANE**	17	0.47	1.32	0.1380	0.2490
**REGULATION OF LYMPHOCYTE APOPTOTIC PROCESS**	47	-0.40	-1.55	0.0186	0.2491
**CYCLIN DEPENDENT PROTEIN SERINE THREONINE KINASE REGULATOR ACTIVITY**	24	0.43	1.32	0.1237	0.2491
**RESPONSE TO IONIZING RADIATION**	122	0.32	1.32	0.0636	0.2492
**MUSCLE CELL FATE COMMITMENT**	15	-0.54	-1.55	0.0554	0.2503
**MACROMITOPHAGY**	109	0.33	1.32	0.0596	0.2505

**NEGATIVE REGULATION OF CELL DIVISION**	53	0.37	1.32	0.1003	0.2513
**REGULATION OF PROTEIN POLYMERIZATION**	147	0.31	1.32	0.0635	0.2515
**NEGATIVE REGULATION OF PROTEIN KINASE B SIGNALING**	34	0.41	1.32	0.1281	0.2515
**DORSAL VENTRAL NEURAL TUBE PATTERNING**	15	-0.55	-1.55	0.0404	0.2520
**RAB GUANYL NUCLEOTIDE EXCHANGE FACTOR ACTIVITY**	27	0.42	1.32	0.1261	0.2525
**POSITIVE REGULATION OF CANONICAL WNT SIGNALING PATHWAY**	110	0.32	1.32	0.0725	0.2530
**DNA RECOMBINATION**	163	0.31	1.32	0.0521	0.2530
**POTASSIUM ION TRANSMEMBRANE TRANSPORTER ACTIVITY**	138	-0.33	-1.55	0.0000	0.2536
**CYTOSOLIC LARGE RIBOSOMAL SUBUNIT**	47	0.37	1.32	0.1173	0.2540
**REGULATION OF MITOCHONDRIAL MEMBRANE POTENTIAL**	48	0.37	1.31	0.0789	0.2547
**SPINDLE ASSEMBLY**	59	0.36	1.31	0.0988	0.2547
**PIGMENT GRANULE ORGANIZATION**	20	0.46	1.31	0.1431	0.2555
**MYOSIN V BINDING**	16	0.47	1.31	0.1519	0.2556
**CELLULAR RESPONSE TO HYDROGEN PEROXIDE**	54	0.37	1.31	0.1058	0.2564
**ALPHA AMINO ACID BIOSYNTHETIC PROCESS**	69	0.35	1.31	0.0769	0.2567
**I KAPPAB KINASE NF KAPPAB SIGNALING**	61	0.35	1.31	0.1060	0.2573
**NEGATIVE REGULATION OF CALCIUM MEDIATED SIGNALING**	16	0.48	1.31	0.1491	0.2577
**AP TYPE MEMBRANE COAT ADAPTOR COMPLEX**	35	0.40	1.31	0.1140	0.2580
**SEX DETERMINATION**	22	-0.48	-1.54	0.0378	0.2583
**NEGATIVE REGULATION OF ALPHA BETA T CELL ACTIVATION**	18	-0.53	-1.54	0.0249	0.2599
**REGULATION OF REACTIVE OXYGEN SPECIES METABOLIC PROCESS**	125	0.31	1.31	0.0678	0.2599
**EXTERNAL SIDE OF PLASMA MEMBRANE**	183	-0.32	-1.54	0.0040	0.2600
**IRE1 MEDIATED UNFOLDED PROTEIN RESPONSE**	51	0.37	1.31	0.1130	0.2601
**POSITIVE REGULATION OF APOPTOTIC SIGNALING PATHWAY**	149	0.31	1.31	0.0440	0.2601
**NEPHRON TUBULE FORMATION**	18	-0.52	-1.54	0.0373	0.2602

**WNT SIGNALING PATHWAY CALCIUM MODULATING PATHWAY**	39	0.38	1.31	0.1099	0.2604
**HISTONE MONOUBIQUITINATION**	18	0.46	1.31	0.1450	0.2607
**REGIONALIZATION**	291	-0.30	-1.54	0.0000	0.2610
**CARBOHYDRATE TRANSPORT**	79	0.34	1.31	0.0921	0.2613
**UDP GALACTOSYLTRANSFERASE ACTIVITY**	25	0.42	1.31	0.1327	0.2621
**NEGATIVE REGULATION OF I KAPPAB KINASE NF KAPPAB SIGNALING**	46	0.37	1.31	0.1068	0.2622
**PURINE CONTAINING COMPOUND CATABOLIC PROCESS**	46	0.37	1.31	0.1188	0.2624
**CONDENSED CHROMOSOME CENTROMERIC REGION**	79	0.34	1.31	0.0849	0.2631
**POLYPEPTIDE N ACETYLGALACTOSAMINYLTRANSFERASE ACTIVITY**	17	0.47	1.31	0.1408	0.2632
**NEGATIVE REGULATION OF CYCLIN DEPENDENT PROTEIN KINASE ACTIVITY**	29	0.41	1.31	0.1330	0.2642
**LIPID HOMEOSTASIS**	97	0.32	1.30	0.0716	0.2643
**REGULATION OF MICROTUBULE BASED PROCESS**	202	0.29	1.31	0.0519	0.2643
**POSITIVE REGULATION OF FAT CELL DIFFERENTIATION**	44	0.37	1.30	0.1066	0.2643
**REGULATION OF DOUBLE STRAND BREAK REPAIR**	35	0.39	1.30	0.1195	0.2645
**REGULATION OF CELL GROWTH**	354	0.28	1.30	0.0169	0.2646
**TOXIN TRANSPORT**	36	0.39	1.30	0.1028	0.2646
**WNT PROTEIN BINDING**	28	0.41	1.30	0.1325	0.2658
**CELLULAR METABOLIC COMPOUND SALVAGE**	33	0.40	1.30	0.1109	0.2658
**MONOVALENT INORGANIC CATION HOMEOSTASIS**	106	0.32	1.30	0.0658	0.2659
**CYTOCHROME COMPLEX**	17	0.46	1.30	0.1690	0.2660
**TRANSLATION REGULATOR ACTIVITY NUCLEIC ACID BINDING**	16	0.47	1.30	0.1301	0.2660
**PIGMENTATION**	77	0.34	1.30	0.0956	0.2661
**PHOSPHATIDYLCHOLINE METABOLIC PROCESS**	59	0.36	1.30	0.1113	0.2661
**REGULATION OF TYROSINE PHOSPHORYLATION OF STAT1 PROTEIN**	16	-0.52	-1.53	0.0400	0.2664
**POSITIVE REGULATION OF FATTY ACID BIOSYNTHETIC PROCESS**	17	-0.51	-1.53	0.0404	0.2666

**VOLTAGE GATED CALCIUM CHANNEL ACTIVITY**	40	-0.42	-1.53	0.0054	0.2670
**POSITIVE REGULATION OF MRNA PROCESSING**	29	0.41	1.30	0.1426	0.2674
**POSITIVE REGULATION OF RECEPTOR ACTIVITY**	43	0.38	1.30	0.1111	0.2678
**POSITIVE REGULATION OF PEPTIDASE ACTIVITY**	134	0.31	1.30	0.0512	0.2679
**CLATHRIN VESICLE COAT**	22	0.44	1.30	0.1328	0.2679
**RNA POLYMERASE II TRANSCRIPTION FACTOR COMPLEX**	88	0.33	1.30	0.0856	0.2679
**RESPONSE TO INTERLEUKIN 4**	27	-0.46	-1.53	0.0282	0.2679
**RNA POLYMERASE II ACTIVATING TRANSCRIPTION FACTOR BINDING**	34	-0.43	-1.53	0.0332	0.2682
**NEGATIVE REGULATION OF DNA REPLICATION**	45	0.37	1.30	0.1151	0.2684
**REGULATION OF DENDRITE MORPHOGENESIS**	73	0.34	1.30	0.0921	0.2685
**POSITIVE REGULATION OF B CELL PROLIFERATION**	36	-0.43	-1.53	0.0129	0.2686
**COMPLEMENT ACTIVATION**	34	-0.43	-1.53	0.0254	0.2693
**POSITIVE REGULATION OF CELLULAR PROTEIN LOCALIZATION**	312	0.28	1.30	0.0348	0.2700
**REGULATION OF OXIDATIVE STRESS INDUCED INTRINSIC APOPTOTIC SIGNALING PATHWAY**	26	-0.45	-1.53	0.0381	0.2701
**POSITIVE REGULATION OF BLOOD VESSEL ENDOTHELIAL CELL MIGRATION**	24	-0.46	-1.52	0.0460	0.2705
**DETOXIFICATION**	65	0.35	1.30	0.0989	0.2709
**CYTOKINE MEDIATED SIGNALING PATHWAY**	368	-0.29	-1.53	0.0000	0.2711
**ESC E Z COMPLEX**	15	0.48	1.30	0.1574	0.2711
**ACTIVATION OF CYSTEINE TYPE ENDOPEPTIDASE ACTIVITY**	82	0.33	1.30	0.1060	0.2712
**POSITIVE REGULATION OF INTERLEUKIN 1 PRODUCTION**	26	-0.45	-1.52	0.0288	0.2720
**NUCLEOSIDE PHOSPHATE CATABOLIC PROCESS**	61	0.35	1.30	0.1027	0.2722
**PROLINE RICH REGION BINDING**	19	0.45	1.30	0.1476	0.2725
**COATED PIT**	61	0.35	1.30	0.1030	0.2725
**PYRIMIDINE NUCLEOBASE METABOLIC PROCESS**	17	0.47	1.30	0.1446	0.2725
**RESPONSE TO MUSCLE ACTIVITY**	17	0.45	1.30	0.1725	0.2726
**ADIPOSE TISSUE DEVELOPMENT**	31	0.41	1.30	0.1514	0.2727

**REGULATION OF ORGANELLE ASSEMBLY**	135	0.31	1.30	0.0720	0.2727
**POSITIVE REGULATION OF RELEASE OF CYTOCHROME C FROM MITOCHONDRIA**	24	0.43	1.29	0.1531	0.2736
**OXIDOREDUCTASE ACTIVITY ACTING ON SINGLE DONORS WITH INCORPORATION OF MOLECULAR OXYGEN**	25	-0.46	-1.52	0.0291	0.2741
**REGULATION OF CARDIAC MUSCLE TISSUE DEVELOPMENT**	42	0.38	1.29	0.1188	0.2746
**GLYCINE METABOLIC PROCESS**	15	0.48	1.29	0.1675	0.2747
**REGULATION OF CYTOKINESIS**	56	0.36	1.29	0.1138	0.2749
**REFLEX**	20	0.44	1.29	0.1488	0.2761
**NEGATIVE REGULATION OF BINDING**	111	0.32	1.29	0.0754	0.2763
**CELLULAR SODIUM ION HOMEOSTASIS**	18	0.46	1.29	0.1672	0.2764
**POSITIVE REGULATION OF INTRACELLULAR PROTEIN TRANSPORT**	226	0.29	1.29	0.0396	0.2764
**PROTEIN LOCALIZATION TO NUCLEUS**	140	0.31	1.29	0.0688	0.2773
**EXCITATORY POSTSYNAPTIC POTENTIAL**	27	0.41	1.29	0.1453	0.2775
**INTRACELLULAR RECEPTOR SIGNALING PATHWAY**	151	0.30	1.29	0.0624	0.2776
**PHOSPHATE TRANSMEMBRANE TRANSPORTER ACTIVITY**	27	0.42	1.29	0.1395	0.2779
**ACTIVATION OF INNATE IMMUNE RESPONSE**	178	0.30	1.29	0.0581	0.2781
**SYNTAXIN 1 BINDING**	16	0.47	1.29	0.1631	0.2784
**REGULATION OF CELLULAR RESPIRATION**	20	0.44	1.29	0.1528	0.2786
**ESTABLISHMENT OF LOCALIZATION BY MOVEMENT ALONG MICROTUBULE**	80	0.33	1.29	0.0953	0.2787
**REGULATION OF TRANSCRIPTION FACTOR IMPORT INTO NUCLEUS**	83	0.33	1.29	0.1043	0.2794
**REGULATION OF NUCLEOSIDE METABOLIC PROCESS**	44	0.37	1.29	0.1290	0.2795
**AXO DENDRITIC TRANSPORT**	33	0.40	1.29	0.1518	0.2796
**NEGATIVE REGULATION OF CELL CYCLE**	366	0.28	1.29	0.0256	0.2797
**REPLISOME**	26	0.41	1.29	0.1419	0.2799
**SPINDLE MICROTUBULE**	50	0.36	1.29	0.1086	0.2801
**POSITIVE REGULATION OF NUCLEASE ACTIVITY**	15	-0.53	-1.51	0.0468	0.2832
**LYMPHOCYTE MEDIATED IMMUNITY**	82	-0.35	-1.51	0.0093	0.2832
**NEGATIVE REGULATION OF JNK CASCADE**	30	0.40	1.29	0.1451	0.2835
**SAGA TYPE COMPLEX**	27	0.41	1.29	0.1388	0.2835

**REGULATION OF ATP METABOLIC PROCESS**	44	0.37	1.28	0.1284	0.2852
**REGULATION OF SYSTEMIC ARTERIAL BLOOD PRESSURE**	74	0.34	1.28	0.1031	0.2862
**NEGATIVE REGULATION OF CALCIUM ION TRANSPORT INTO CYTOSOL**	17	0.46	1.28	0.1661	0.2862
**NEGATIVE REGULATION OF CHROMOSOME ORGANIZATION**	85	0.33	1.28	0.1096	0.2864
**REGULATION OF SYNAPTIC TRANSMISSION GABAERGIC**	27	0.41	1.28	0.1546	0.2864
**RECYCLING ENDOSOME MEMBRANE**	38	0.38	1.28	0.1242	0.2865
**CYCLIC NUCLEOTIDE CATABOLIC PROCESS**	17	0.46	1.28	0.1589	0.2866
**PLASMA MEMBRANE RAFT**	79	0.33	1.28	0.0942	0.2867
**ALCOHOL METABOLIC PROCESS**	310	0.28	1.28	0.0380	0.2869
**RETINOID X RECEPTOR BINDING**	17	0.45	1.28	0.1849	0.2869
**NEGATIVE REGULATION OF SEQUENCE SPECIFIC DNA BINDING TRANSCRIPTION FACTOR ACTIVITY**	117	0.31	1.28	0.0858	0.2870
**PURINE NUCLEOSIDE BISPHOSPHATE BIOSYNTHETIC PROCESS**	16	0.46	1.28	0.1804	0.2870
**NUCLEAR MATRIX**	81	0.33	1.28	0.0978	0.2871
**REGULATION OF PROTEIN IMPORT**	160	0.29	1.28	0.0740	0.2882
**REGULATION OF MAMMARY GLAND EPITHELIAL CELL PROLIFERATION**	15	0.48	1.28	0.1746	0.2884
**NEGATIVE REGULATION OF INTRACELLULAR SIGNAL TRANSDUCTION**	395	0.27	1.28	0.0214	0.2893
**POSITIVE REGULATION OF FATTY ACID METABOLIC PROCESS**	33	0.39	1.28	0.1323	0.2897
**SPINDLE**	236	0.29	1.28	0.0556	0.2907
**EMBRYONIC HEMOPOIESIS**	16	0.46	1.28	0.1676	0.2922
**ATPASE ACTIVITY COUPLED TO MOVEMENT OF SUBSTANCES**	113	0.31	1.28	0.1094	0.2923
**NEGATIVE REGULATION OF STRIATED MUSCLE CELL APOPTOTIC PROCESS**	16	-0.52	-1.51	0.0377	0.2924
**REGULATION OF CELLULAR PH**	63	0.34	1.28	0.1095	0.2927
**INNATE IMMUNE RESPONSE**	411	-0.28	-1.51	0.0000	0.2932
**POSITIVE REGULATION OF TELOMERE MAINTENANCE**	40	0.37	1.28	0.1458	0.2933
**POSITIVE REGULATION OF INTERLEUKIN 10 PRODUCTION**	27	-0.45	-1.51	0.0308	0.2935

**OXIDOREDUCTASE ACTIVITY ACTING ON A HEME GROUP OF DONORS**	20	0.44	1.28	0.1609	0.2935
**INTRACILIARY TRANSPORT PARTICLE B**	17	0.45	1.28	0.1581	0.2942
**RESPONSE TO ACIDIC PH**	19	-0.49	-1.50	0.0501	0.2943
**RETINA VASCULATURE DEVELOPMENT IN CAMERA TYPE EYE**	15	0.48	1.28	0.1556	0.2944
**HYDROLASE ACTIVITY ACTING ON GLYCOSYL BONDS**	96	0.32	1.28	0.0882	0.2947
**PHOSPHATIDYLINOSITOL PHOSPHATE KINASE ACTIVITY**	15	0.47	1.27	0.1850	0.2949
**INTERCALATED DISC**	46	0.37	1.27	0.1374	0.2950
**RESPONSE TO FATTY ACID**	74	0.33	1.27	0.1035	0.2951
**IMMUNE RESPONSE REGULATING CELL SURFACE RECEPTOR SIGNALING PATHWAY**	241	0.28	1.27	0.0623	0.2956
**PROTEIN LIPID COMPLEX ASSEMBLY**	19	0.45	1.27	0.1564	0.2965
**REGULATION OF CARDIAC MUSCLE CELL DIFFERENTIATION**	17	0.45	1.27	0.1863	0.2970
**AU RICH ELEMENT BINDING**	23	0.42	1.27	0.1684	0.2976
**NEGATIVE REGULATION OF ORGANELLE ASSEMBLY**	20	0.44	1.27	0.1682	0.2999
**MONOAMINE TRANSPORT**	20	0.44	1.27	0.1710	0.3005
**REGULATION OF CANONICAL WNT SIGNALING PATHWAY**	220	0.29	1.27	0.0488	0.3019
**EPIDERMAL GROWTH FACTOR RECEPTOR BINDING**	27	0.41	1.27	0.1621	0.3020
**ENDOPLASMIC RETICULUM CALCIUM ION HOMEOSTASIS**	19	0.44	1.27	0.1847	0.3020
**NEGATIVE REGULATION OF OXIDATIVE STRESS INDUCED INTRINSIC APOPTOTIC SIGNALING PATHWAY**	18	-0.51	-1.50	0.0502	0.3020
**REGULATION OF RHODOPSIN MEDIATED SIGNALING PATHWAY**	25	0.42	1.27	0.1628	0.3021
**REGULATION OF CLATHRIN MEDIATED ENDOCYTOSIS**	15	0.47	1.27	0.1818	0.3021
**NEGATIVE REGULATION OF GENE EXPRESSION EPIGENETIC**	75	-0.36	-1.50	0.0176	0.3021
**ORGANIC ACID BIOSYNTHETIC PROCESS**	232	0.28	1.27	0.0539	0.3023
**MRNA CLEAVAGE**	20	0.44	1.27	0.1716	0.3023
**MITOTIC SISTER CHROMATID SEGREGATION**	74	0.33	1.27	0.1343	0.3023
**HISTONE METHYLATION**	75	0.33	1.27	0.1216	0.3024

**CELLULAR RESPONSE TO REACTIVE OXYGEN SPECIES**	93	0.32	1.27	0.0830	0.3024
**RIBONUCLEOSIDE BISPHOSPHATE BIOSYNTHETIC PROCESS**	16	0.46	1.27	0.1678	0.3026
**NEGATIVE REGULATION OF NUCLEAR DIVISION**	40	0.37	1.27	0.1510	0.3028
**OXIDOREDUCTASE ACTIVITY ACTING ON THE CH NH GROUP OF DONORS**	24	0.42	1.27	0.1531	0.3030
**NEGATIVE REGULATION OF WNT SIGNALING PATHWAY**	184	0.29	1.27	0.0692	0.3030
**NEGATIVE REGULATION OF INTERLEUKIN 1 PRODUCTION**	15	-0.51	-1.50	0.0585	0.3033
**EMBRYONIC AXIS SPECIFICATION**	32	-0.42	-1.49	0.0521	0.3033
**HORMONE RECEPTOR BINDING**	139	0.30	1.27	0.1086	0.3034
**NEGATIVE REGULATION OF DNA METABOLIC PROCESS**	90	0.32	1.27	0.1015	0.3035
**NEGATIVE REGULATION OF B CELL ACTIVATION**	26	-0.46	-1.50	0.0372	0.3036
**MICROTUBULE NUCLEATION**	18	0.45	1.27	0.1783	0.3037
**RETROMER COMPLEX**	19	0.44	1.27	0.1889	0.3047
**CORNIFIED ENVELOPE**	32	-0.43	-1.49	0.0372	0.3050
**RESPONSE TO NITRIC OXIDE**	19	0.44	1.26	0.1658	0.3052
**POSITIVE REGULATION OF CELL PROJECTION ORGANIZATION**	279	0.28	1.26	0.0640	0.3054
**CHROMOSOME CENTROMERIC REGION**	143	0.30	1.26	0.0845	0.3055
**CIRCADIAN RHYTHM**	123	0.31	1.26	0.0846	0.3056
**PROTEIN FOLDING**	187	0.29	1.26	0.0684	0.3056
**REGULATION OF CELL CYCLE G1 S PHASE TRANSITION**	126	0.31	1.26	0.0842	0.3057
**POSITIVE REGULATION OF TOR SIGNALING**	25	0.41	1.26	0.1740	0.3058
**STEROID HORMONE MEDIATED SIGNALING PATHWAY**	113	0.31	1.26	0.0891	0.3060
**HUMORAL IMMUNE RESPONSE MEDIATED BY CIRCULATING IMMUNOGLOBULIN**	27	-0.43	-1.49	0.0496	0.3061
**CELL DIFFERENTIATION IN SPINAL CORD**	50	-0.38	-1.49	0.0170	0.3068
**PEPTIDYL ARGININE MODIFICATION**	16	0.45	1.26	0.1853	0.3068
**CELL VOLUME HOMEOSTASIS**	26	0.41	1.26	0.1567	0.3069
**NUCLEAR INNER MEMBRANE**	46	0.36	1.26	0.1341	0.3070
**CHOLESTEROL HOMEOSTASIS**	52	0.35	1.26	0.1392	0.3071
**CELLULAR RESPONSE TO GLUCOSE STARVATION**	26	0.41	1.26	0.1678	0.3072

**REGULATION OF TRIGLYCERIDE BIOSYNTHETIC PROCESS**	16	0.47	1.26	0.1727	0.3082
**NEGATIVE REGULATION OF SIGNAL TRANSDUCTION BY P53 CLASS MEDIATOR**	26	0.41	1.26	0.1867	0.3084
**PHOSPHATIDYLINOSITOL 3 5 BISPHOSPHATE BINDING**	18	0.45	1.26	0.1898	0.3085
**POSITIVE REGULATION OF OSTEOCLAST DIFFERENTIATION**	21	-0.47	-1.49	0.0422	0.3085
**CARBOXYLIC ACID BIOSYNTHETIC PROCESS**	232	0.28	1.26	0.0608	0.3087
**PHOSPHOLIPID TRANSPORT**	56	0.35	1.26	0.1256	0.3087
**NEGATIVE REGULATION OF MACROAUTOPHAGY**	18	0.45	1.26	0.2034	0.3088
**INTRACELLULAR STEROID HORMONE RECEPTOR SIGNALING PATHWAY**	62	0.33	1.26	0.1262	0.3096
**CELLULAR RESPONSE TO EXTRACELLULAR STIMULUS**	169	0.29	1.26	0.0854	0.3097
**CELLULAR RESPONSE TO OXIDATIVE STRESS**	161	0.29	1.26	0.0862	0.3107
**CHROMATIN REMODELING**	118	0.30	1.26	0.0972	0.3113
**ONE CARBON METABOLIC PROCESS**	20	0.45	1.26	0.1893	0.3113
**TELOMERE MAINTENANCE VIA TELOMERASE**	15	0.47	1.26	0.1915	0.3115
**LONG TERM MEMORY**	27	0.41	1.26	0.1906	0.3116
**ATRIOVENTRICULAR VALVE MORPHOGENESIS**	16	-0.50	-1.48	0.0782	0.3135
**ENDORIBONUCLEASE COMPLEX**	20	0.43	1.26	0.1661	0.3144
**REGULATION OF OLIGODENDROCYTE DIFFERENTIATION**	27	0.40	1.26	0.1757	0.3145
**REGULATION OF T CELL DIFFERENTIATION**	90	-0.34	-1.48	0.0122	0.3145
**PRONUCLEUS**	15	0.46	1.26	0.2092	0.3146
**INCLUSION BODY**	63	0.33	1.26	0.1473	0.3146
**REGULATION OF CARDIAC MUSCLE CONTRACTION BY CALCIUM ION SIGNALING**	21	0.43	1.26	0.1855	0.3147
**INTRACELLULAR LIPID TRANSPORT**	19	0.44	1.26	0.1874	0.3148
**SEGMENT SPECIFICATION**	16	-0.52	-1.48	0.0611	0.3151
**NUCLEOSIDE BISPHOSPHATE BIOSYNTHETIC PROCESS**	16	0.46	1.25	0.1703	0.3154
**NEGATIVE REGULATION OF KINASE ACTIVITY**	230	0.28	1.25	0.0562	0.3158
**REGULATION OF JNK CASCADE**	146	0.30	1.25	0.0815	0.3161

**REGULATION OF LEUKOCYTE PROLIFERATION**	166	-0.30	-1.48	0.0035	0.3164
**EUCHROMATIN**	27	0.41	1.25	0.1711	0.3170
**REGULATION OF CIRCADIAN RHYTHM**	91	0.31	1.25	0.1108	0.3170
**POSITIVE REGULATION OF INNATE IMMUNE RESPONSE**	216	0.28	1.25	0.0663	0.3173
**AGING**	229	0.28	1.25	0.0701	0.3180
**SMOOTH MUSCLE CONTRACTION**	42	0.37	1.25	0.1469	0.3182
**PROTEIN EXIT FROM ENDOPLASMIC RETICULUM**	18	0.45	1.25	0.2076	0.3187
**REACTIVE OXYGEN SPECIES METABOLIC PROCESS**	84	0.32	1.25	0.1092	0.3194
**CELLULAR RESPONSE TO INORGANIC SUBSTANCE**	126	0.30	1.25	0.1215	0.3195
**REGULATION OF CELLULAR RESPONSE TO HEAT**	65	0.33	1.25	0.1309	0.3196
**HEPATICOBILIARY SYSTEM DEVELOPMENT**	117	0.30	1.25	0.1060	0.3197
**ACUTE INFLAMMATORY RESPONSE**	62	0.34	1.25	0.1626	0.3198
**NEGATIVE REGULATION OF CYTOSKELETON ORGANIZATION**	184	0.29	1.25	0.0844	0.3207
**RESPONSE TO EPIDERMAL GROWTH FACTOR**	28	0.40	1.25	0.1707	0.3208
**PYRIDOXAL PHOSPHATE BINDING**	48	0.36	1.25	0.1654	0.3211
**REGULATION OF INSULIN SECRETION INVOLVED IN CELLULAR RESPONSE TO GLUCOSE STIMULUS**	44	0.36	1.25	0.1661	0.3212
**MITOTIC CELL CYCLE CHECKPOINT**	117	0.31	1.25	0.1216	0.3218
**PHOSPHATIDYLINOSITOL DEPHOSPHORYLATION**	19	0.44	1.25	0.1993	0.3220
**CELLULAR RESPONSE TO STEROID HORMONE STIMULUS**	194	0.29	1.25	0.0760	0.3232
**CELL CYCLE ARREST**	132	0.30	1.25	0.1036	0.3239
**AXON CYTOPLASM**	30	0.39	1.25	0.1597	0.3247
**LYMPHOCYTE ACTIVATION INVOLVED IN IMMUNE RESPONSE**	80	-0.34	-1.48	0.0182	0.3252
**NEGATIVE REGULATION OF INNATE IMMUNE RESPONSE**	28	0.39	1.25	0.1728	0.3257
**CELL DIFFERENTIATION INVOLVED IN KIDNEY DEVELOPMENT**	35	-0.40	-1.47	0.0322	0.3257
**POSITIVE REGULATION OF LEUKOCYTE PROLIFERATION**	110	-0.33	-1.48	0.0000	0.3259
**PYRIMIDINE NUCLEOTIDE METABOLIC PROCESS**	38	0.37	1.25	0.1542	0.3262

**INTERSTRAND CROSS LINK REPAIR**	27	0.40	1.25	0.1786	0.3262
**REGULATION OF I KAPPAB KINASE NF KAPPAB SIGNALING**	205	0.28	1.24	0.0771	0.3268
**THYROID GLAND DEVELOPMENT**	23	-0.46	-1.47	0.0350	0.3270
**EXOPEPTIDASE ACTIVITY**	94	0.31	1.24	0.1354	0.3273
**OXIDOREDUCTASE ACTIVITY ACTING ON THE ALDEHYDE OR OXO GROUP OF DONORS**	40	0.37	1.24	0.1651	0.3276
**NADP BINDING**	38	0.38	1.24	0.1692	0.3283
**REGULATION OF VASCULATURE DEVELOPMENT**	216	-0.30	-1.47	0.0000	0.3286
**FIBRONECTIN BINDING**	22	-0.46	-1.47	0.0597	0.3286
**REGULATION OF MESONEPHROS DEVELOPMENT**	26	-0.43	-1.47	0.0437	0.3295
**ORGANELLE MEMBRANE FUSION**	84	0.32	1.24	0.1555	0.3297
**SOMATIC DIVERSIFICATION OF IMMUNOGLOBULINS**	22	0.41	1.24	0.1726	0.3298
**GLYCOSYLATION**	223	0.28	1.24	0.0714	0.3299
**14 3 3 PROTEIN BINDING**	17	-0.49	-1.47	0.0862	0.3301
**STEROL HOMEOSTASIS**	52	0.35	1.24	0.1483	0.3302
**AMIDE BINDING**	216	0.28	1.24	0.0901	0.3305
**HISTONE DEMETHYLASE ACTIVITY**	24	0.41	1.24	0.1872	0.3305
**TETRAHYDROFOLATE METABOLIC PROCESS**	16	0.45	1.24	0.1864	0.3306
**ORGANOPHOSPHATE ESTER TRANSPORT**	85	0.32	1.24	0.1166	0.3315
**CELL DIVISION SITE**	50	0.35	1.24	0.1591	0.3319
**MONOVALENT INORGANIC ANION HOMEOSTASIS**	16	0.45	1.24	0.1996	0.3320
**REGULATION OF NUCLEOCYTOPLASMIC TRANSPORT**	195	0.28	1.24	0.0978	0.3320
**REGULATION OF MRNA 3 END PROCESSING**	27	0.40	1.24	0.1772	0.3321
**REGULATION OF CYCLIN DEPENDENT PROTEIN KINASE ACTIVITY**	83	0.32	1.24	0.1403	0.3322
**CYTOKINESIS**	69	0.33	1.24	0.1451	0.3324
**POLYOL METABOLIC PROCESS**	86	0.32	1.24	0.1256	0.3327
**HORMONE MEDIATED SIGNALING PATHWAY**	143	0.29	1.24	0.1257	0.3329
**RUFFLE ORGANIZATION**	19	0.44	1.24	0.1946	0.3332
**POSITIVE REGULATION OF MRNA 3 END PROCESSING**	16	0.45	1.24	0.1969	0.3332

**REGULATION OF NEURAL PRECURSOR CELL PROLIFERATION**	69	-0.35	-1.46	0.0171	0.3333
**G PROTEIN COUPLED AMINE RECEPTOR ACTIVITY**	39	-0.39	-1.46	0.0350	0.3340
**MONOOXYGENASE ACTIVITY**	57	-0.36	-1.46	0.0222	0.3350
**CHROMATIN BINDING**	390	0.26	1.24	0.0486	0.3357
**SYNAPTIC TRANSMISSION GLUTAMATERGIC**	21	0.42	1.24	0.1642	0.3367
**RETINOIC ACID RECEPTOR BINDING**	27	0.39	1.24	0.1801	0.3373
**REGULATION OF PROTEIN LOCALIZATION TO NUCLEUS**	193	0.29	1.24	0.1011	0.3376
**RESPONSE TO OXYGEN RADICAL**	17	0.44	1.23	0.2142	0.3388
**SKELETAL MUSCLE CELL DIFFERENTIATION**	51	-0.38	-1.46	0.0339	0.3388
**FAT SOLUBLE VITAMIN METABOLIC PROCESS**	27	-0.43	-1.46	0.0564	0.3388
**REGULATION OF B CELL ACTIVATION**	91	-0.33	-1.46	0.0183	0.3390
**REGULATION OF DIGESTIVE SYSTEM PROCESS**	35	0.37	1.23	0.1821	0.3390
**PEPTIDE CROSS LINKING**	43	-0.39	-1.46	0.0347	0.3405
**GALACTOSYLTRANSFERASE ACTIVITY**	28	0.39	1.23	0.1973	0.3406
**RENAL WATER HOMEOSTASIS**	26	0.40	1.23	0.1993	0.3415
**RESPONSE TO EXTRACELLULAR STIMULUS**	405	0.26	1.23	0.0537	0.3415
**CONDENSED NUCLEAR CHROMOSOME CENTROMERIC REGION**	15	-0.51	-1.46	0.0813	0.3423
**PROTEIN MATURATION**	228	0.28	1.23	0.0926	0.3430
**REGULATION OF ANION TRANSPORT**	126	0.30	1.23	0.1097	0.3435
**REGULATION OF VIRAL ENTRY INTO HOST CELL**	25	0.41	1.23	0.1929	0.3435
**REGENERATION**	147	0.29	1.23	0.1213	0.3436
**POSITIVE REGULATION OF WNT SIGNALING PATHWAY**	143	0.29	1.23	0.1015	0.3436
**PATTERN RECOGNITION RECEPTOR SIGNALING PATHWAY**	98	0.31	1.23	0.1301	0.3437
**NEGATIVE REGULATION OF CHROMOSOME SEGREGATION**	25	0.41	1.23	0.1939	0.3437
**NEGATIVE REGULATION OF PLATELET ACTIVATION**	17	0.43	1.23	0.2142	0.3438
**PROTEIN LOCALIZATION TO MEMBRANE**	328	0.26	1.23	0.0636	0.3439
**ENDONUCLEASE ACTIVITY**	95	0.31	1.23	0.1230	0.3439
**CYTOPLASMIC MICROTUBULE ORGANIZATION**	38	0.36	1.23	0.1499	0.3440

**NEGATIVE REGULATION OF PHOSPHORYLATION**	386	0.26	1.23	0.0619	0.3441
**REGULATION OF NEURON APOPTOTIC PROCESS**	166	0.29	1.23	0.0910	0.3444
**POST TRANSLATIONAL PROTEIN MODIFICATION**	24	0.40	1.23	0.2145	0.3444
**ENDOTHELIAL CELL DIFFERENTIATION**	67	0.33	1.23	0.1461	0.3446
**PROTEIN COMPLEX INVOLVED IN CELL ADHESION**	30	-0.43	-1.45	0.0442	0.3455
**TUBULIN BINDING**	227	0.28	1.23	0.0875	0.3455
**GAMMA TUBULIN BINDING**	20	0.43	1.23	0.2143	0.3459
**EMBRYONIC PLACENTA MORPHOGENESIS**	22	-0.45	-1.45	0.0699	0.3460
**RAN GTPASE BINDING**	30	0.38	1.23	0.1905	0.3468
**CELLULAR RESPONSE TO LIPID**	412	0.26	1.23	0.0499	0.3469
**HISTONE METHYLTRANSFERASE ACTIVITY**	52	0.35	1.23	0.1708	0.3478
**STEROID HYDROXYLASE ACTIVITY**	18	-0.48	-1.45	0.0583	0.3484
**CEREBELLAR CORTEX FORMATION**	22	-0.45	-1.45	0.0707	0.3491
**DEATH RECEPTOR ACTIVITY**	20	-0.46	-1.44	0.0614	0.3491
**B CELL MEDIATED IMMUNITY**	51	-0.37	-1.45	0.0292	0.3496
**REGULATION OF MYOTUBE DIFFERENTIATION**	51	-0.38	-1.45	0.0382	0.3496
**MITOTIC DNA INTEGRITY CHECKPOINT**	84	0.31	1.23	0.1503	0.3498
**POLYOL TRANSPORT**	16	-0.49	-1.45	0.0678	0.3500
**HORMONE ACTIVITY**	96	-0.33	-1.45	0.0176	0.3507
**T CELL DIFFERENTIATION INVOLVED IN IMMUNE RESPONSE**	29	-0.44	-1.45	0.0510	0.3512
**LOW DENSITY LIPOPROTEIN PARTICLE RECEPTOR BINDING**	17	0.44	1.22	0.2338	0.3515
**RECOMBINATIONAL REPAIR**	57	0.33	1.22	0.1780	0.3516
**EPITHELIAL CELL FATE COMMITMENT**	15	0.44	1.22	0.2220	0.3521
**BLOC COMPLEX**	16	0.44	1.22	0.1971	0.3522
**NUCLEAR CHROMOSOME SEGREGATION**	189	0.28	1.22	0.0872	0.3524
**ERYTHROCYTE DEVELOPMENT**	17	-0.48	-1.44	0.0657	0.3538
**LIPOPROTEIN PARTICLE RECEPTOR BINDING**	22	0.41	1.22	0.2142	0.3553
**CARBOHYDRATE DERIVATIVE TRANSPORT**	47	0.35	1.22	0.1705	0.3555
**PROTEINACEOUS EXTRACELLULAR MATRIX**	322	-0.27	-1.44	0.0000	0.3556
**EXTRACELLULAR MATRIX**	386	-0.27	-1.44	0.0000	0.3578

**NEGATIVE REGULATION OF HOMOTYPIC CELL CELL ADHESION**	84	-0.33	-1.44	0.0237	0.3585
**POSITIVE REGULATION OF DEVELOPMENTAL GROWTH**	140	0.29	1.22	0.1337	0.3589
**ISOPRENOID BIOSYNTHETIC PROCESS**	25	0.40	1.22	0.2074	0.3589
**REGULATION OF PROTEIN BINDING**	149	0.29	1.22	0.1240	0.3591
**SEMAPHORIN RECEPTOR BINDING**	22	-0.46	-1.44	0.0612	0.3594
**POSITIVE REGULATION OF DEPHOSPHORYLATION**	44	0.35	1.22	0.1726	0.3600
**REGULATION OF INTERLEUKIN 1 BETA PRODUCTION**	37	-0.39	-1.44	0.0366	0.3606
**REGULATION OF TYPE I INTERFERON MEDIATED SIGNALING PATHWAY**	30	-0.41	-1.43	0.0536	0.3607
**REGULATION OF CELL CYCLE CHECKPOINT**	24	0.41	1.22	0.1829	0.3612
**PROTEIN MANNOSYLATION**	17	0.44	1.22	0.2139	0.3613
**RESPONSE TO IRON ION**	30	0.38	1.22	0.2079	0.3613
**REGULATION OF AUTOPHAGOSOME ASSEMBLY**	31	0.38	1.22	0.2085	0.3614
**ATPASE COMPLEX**	24	0.40	1.22	0.2003	0.3616
**REGULATION OF NEURON DEATH**	217	0.27	1.22	0.1088	0.3623
**NEGATIVE REGULATION OF REACTIVE OXYGEN SPECIES BIOSYNTHETIC PROCESS**	16	0.45	1.22	0.2236	0.3624
**CYTOPLASMIC STRESS GRANULE**	30	0.38	1.22	0.2091	0.3625
**PROTEIN PHOSPHORYLATED AMINO ACID BINDING**	23	0.41	1.22	0.2177	0.3627
**NEGATIVE REGULATION OF FIBROBLAST PROLIFERATION**	23	0.41	1.22	0.2281	0.3640
**CELL AGING**	55	0.34	1.21	0.1712	0.3647
**CELLULAR COMPONENT DISASSEMBLY INVOLVED IN EXECUTION PHASE OF APOPTOSIS**	39	0.36	1.21	0.1768	0.3648
**PHOSPHOLIPASE A2 ACTIVITY**	29	0.39	1.21	0.2000	0.3650
**NEGATIVE REGULATION OF LYMPHOCYTE MEDIATED IMMUNITY**	24	-0.44	-1.43	0.0588	0.3657
**CARBOXY TERMINAL DOMAIN PROTEIN KINASE COMPLEX**	20	0.42	1.21	0.2213	0.3665
**REGULATION OF DENDRITE DEVELOPMENT**	115	0.30	1.21	0.1360	0.3666
**REGULATION OF SMOOTH MUSCLE CELL MIGRATION**	46	0.34	1.21	0.1766	0.3667

**PROTEIN SERINE THREONINE KINASE ACTIVITY**	401	0.26	1.21	0.0648	0.3667
**PHOSPHATIDYLETHANOLAMINE ACYL CHAIN REMODELING**	22	0.40	1.21	0.2194	0.3681
**INTRAMOLECULAR OXIDOREDUCTASE ACTIVITY TRANSPOSING S S BONDS**	21	0.41	1.21	0.2101	0.3686
**SCAFFOLD PROTEIN BINDING**	43	0.35	1.21	0.1773	0.3699
**SNARE BINDING**	111	0.30	1.21	0.1360	0.3699
**CLATHRIN COAT**	43	0.35	1.21	0.1948	0.3700
**NEGATIVE REGULATION OF T CELL RECEPTOR SIGNALING PATHWAY**	15	0.46	1.21	0.2201	0.3700
**POSITIVE REGULATION OF NEURON DEATH**	57	0.33	1.21	0.1888	0.3702
**PROTEIN TYROSINE KINASE BINDING**	51	0.34	1.21	0.2048	0.3706
**LEUKOCYTE MEDIATED IMMUNITY**	118	-0.31	-1.43	0.0000	0.3709
**CELLULAR RESPONSE TO INSULIN STIMULUS**	135	0.29	1.21	0.1546	0.3710
**ATRIOVENTRICULAR VALVE DEVELOPMENT**	19	-0.47	-1.43	0.0771	0.3711
**NEGATIVE REGULATION OF BMP SIGNALING PATHWAY**	37	0.36	1.21	0.1957	0.3712
**REGULATION OF DEFENSE RESPONSE TO VIRUS BY HOST**	103	0.30	1.21	0.1437	0.3712
**CILIUM MOVEMENT**	24	0.40	1.21	0.2153	0.3713
**REGULATION OF DNA BIOSYNTHETIC PROCESS**	82	0.31	1.21	0.1634	0.3713
**AMINO ACID TRANSMEMBRANE TRANSPORT**	60	0.33	1.21	0.1861	0.3714
**MHC PROTEIN BINDING**	19	-0.47	-1.43	0.0679	0.3716
**CYCLIC NUCLEOTIDE BIOSYNTHETIC PROCESS**	32	-0.40	-1.43	0.0560	0.3716
**KINASE INHIBITOR ACTIVITY**	86	0.31	1.21	0.1597	0.3729
**OXIDOREDUCTASE ACTIVITY ACTING ON THE ALDEHYDE OR OXO GROUP OF DONORS NAD OR NADP AS ACCEPTOR**	31	0.37	1.21	0.2240	0.3730
**REGULATION OF CELL DIVISION**	238	0.27	1.21	0.0859	0.3731
**REGULATION OF CARDIAC MUSCLE CELL PROLIFERATION**	25	0.39	1.21	0.2185	0.3732
**REGULATION OF WNT SIGNALING PATHWAY**	288	0.27	1.21	0.0851	0.3737
**POSITIVE REGULATION OF EXOCYTOSIS**	75	0.32	1.21	0.1807	0.3740
**INTRINSIC COMPONENT OF ENDOPLASMIC RETICULUM MEMBRANE**	106	0.29	1.21	0.1506	0.3740

**CELL PROJECTION CYTOPLASM**	47	0.34	1.21	0.1897	0.3742
**TRIGLYCERIDE CATABOLIC PROCESS**	20	0.42	1.21	0.2104	0.3744
**IONOTROPIC GLUTAMATE RECEPTOR BINDING**	22	0.41	1.21	0.2211	0.3753
**GLYCOPROTEIN METABOLIC PROCESS**	306	0.26	1.20	0.0825	0.3754
**REGULATION OF SYNAPSE ASSEMBLY**	73	0.32	1.21	0.1816	0.3754
**H4 HISTONE ACETYLTRANSFERASE COMPLEX**	16	0.44	1.20	0.2540	0.3768
**MITOCHONDRIAL ELECTRON TRANSPORT CYTOCHROME C TO OXYGEN**	15	0.45	1.20	0.2447	0.3769
**RECYCLING ENDOSOME**	115	0.30	1.20	0.1542	0.3774
**BASOLATERAL PLASMA MEMBRANE**	191	0.27	1.20	0.1208	0.3779
**NEGATIVE REGULATION OF NEURON APOPTOTIC PROCESS**	117	0.29	1.20	0.1464	0.3780
**LIGAND DEPENDENT NUCLEAR RECEPTOR BINDING**	21	0.41	1.20	0.2407	0.3797
**MYD88 INDEPENDENT TOLL LIKE RECEPTOR SIGNALING PATHWAY**	28	0.39	1.20	0.2166	0.3800
**NEGATIVE REGULATION OF SMOOTH MUSCLE CELL MIGRATION**	15	0.44	1.20	0.2420	0.3800
**ANDROGEN RECEPTOR SIGNALING PATHWAY**	38	0.36	1.20	0.2022	0.3800
**DAMAGED DNA BINDING**	48	0.34	1.20	0.1894	0.3801
**SNRNA BINDING**	33	0.37	1.20	0.2147	0.3801
**ANTEROGRADE AXONAL TRANSPORT**	23	0.40	1.20	0.2373	0.3801
**CELLULAR RESPONSE TO EXTERNAL STIMULUS**	238	0.27	1.20	0.1127	0.3803
**REGULATION OF CHROMOSOME ORGANIZATION**	239	0.27	1.20	0.1158	0.3805
**INNER EAR MORPHOGENESIS**	87	-0.33	-1.42	0.0226	0.3805
**ENDOTHELIAL CELL DEVELOPMENT**	41	0.35	1.20	0.2146	0.3808
**REGULATION OF PRODUCTION OF MOLECULAR MEDIATOR OF IMMUNE RESPONSE**	83	-0.33	-1.42	0.0300	0.3814
**POSITIVE REGULATION OF INTRACELLULAR TRANSPORT**	325	0.26	1.20	0.0851	0.3818
**APICAL PLASMA MEMBRANE**	264	0.26	1.20	0.0948	0.3818
**RECEPTOR SIGNALING PROTEIN SERINE THREONINE KINASE ACTIVITY**	82	0.31	1.20	0.1813	0.3818
**LIPID TRANSLOCATION**	21	0.41	1.20	0.2424	0.3819
**CORECEPTOR ACTIVITY**	35	0.35	1.20	0.1924	0.3820
**CHROMOSOME SEPARATION**	18	0.42	1.20	0.2590	0.3827

**NECROTIC CELL DEATH**	27	0.39	1.20	0.2437	0.3827
**REGULATION OF DNA DEPENDENT DNA REPLICATION**	36	0.36	1.20	0.2029	0.3831
**POSITIVE REGULATION OF BINDING**	118	0.29	1.20	0.1591	0.3832
**REGULATION OF STRESS ACTIVATED PROTEIN KINASE SIGNALING CASCADE**	179	0.28	1.20	0.1265	0.3833
**CELLULAR RESPONSE TO IONIZING RADIATION**	43	0.34	1.20	0.1910	0.3833
**CHAPERONE BINDING**	69	0.32	1.20	0.2047	0.3835
**REGULATION OF ADAPTIVE IMMUNE RESPONSE**	105	-0.31	-1.42	0.0103	0.3839
**POTASSIUM ION TRANSPORT**	148	-0.30	-1.42	0.0139	0.3845
**NEGATIVE REGULATION OF RESPONSE TO OXIDATIVE STRESS**	31	-0.41	-1.41	0.0590	0.3849
**SPERM MOTILITY**	37	-0.39	-1.41	0.0541	0.3856
**NEURON FATE COMMITMENT**	64	-0.35	-1.42	0.0358	0.3857
**EAR MORPHOGENESIS**	107	-0.31	-1.41	0.0131	0.3863
**MICROTUBULE CYTOSKELETON ORGANIZATION**	276	0.26	1.20	0.1029	0.3867
**MACROMOLECULE DEACYLATION**	61	0.33	1.20	0.1966	0.3867
**MYOFILAMENT**	22	-0.45	-1.41	0.0782	0.3872
**POSITIVE REGULATION OF LIPID METABOLIC PROCESS**	119	0.29	1.19	0.1460	0.3874
**REGULATION OF TRANSCRIPTION INITIATION FROM RNA POLYMERASE II PROMOTER**	20	0.41	1.19	0.2285	0.3874
**POSITIVE REGULATION OF STRESS ACTIVATED PROTEIN KINASE SIGNALING CASCADE**	122	0.29	1.20	0.1394	0.3875
**RESPONSE TO EPINEPHRINE**	15	0.44	1.19	0.2429	0.3875
**HISTONE DEACETYLASE BINDING**	92	0.30	1.19	0.1699	0.3875
**PHOSPHOLIPASE C ACTIVITY**	29	0.38	1.19	0.2230	0.3876
**DYNEIN BINDING**	23	0.41	1.19	0.2385	0.3877
**REGULATION OF HEART MORPHOGENESIS**	29	0.38	1.19	0.2245	0.3877
**HSP70 PROTEIN BINDING**	25	-0.42	-1.41	0.0879	0.3885
**REGULATION OF CAMP MEDIATED SIGNALING**	23	-0.44	-1.41	0.0866	0.3889
**REGULATION OF DENDRITIC SPINE MORPHOGENESIS**	30	0.37	1.19	0.2145	0.3892
**PROTEIN HETEROOLIGOMERIZATION**	86	0.31	1.19	0.1619	0.3893
**RESPONSE TO INORGANIC SUBSTANCE**	422	0.25	1.19	0.0817	0.3894
**CELLULAR RESPONSE TO INTERLEUKIN 4**	23	-0.43	-1.41	0.0627	0.3898

**POSITIVE REGULATION OF MESONEPHROS DEVELOPMENT**	22	-0.44	-1.41	0.0852	0.3900
**REGULATION OF PH**	77	0.31	1.19	0.1980	0.3909
**CELLULAR RESPONSE TO ANTIBIOTIC**	16	-0.49	-1.41	0.0736	0.3915
**T TUBULE**	41	0.35	1.19	0.2056	0.3916
**REGULATION OF DEFENSE RESPONSE TO VIRUS**	154	0.28	1.19	0.1362	0.3918
**MONOSACCHARIDE TRANSPORT**	44	0.35	1.19	0.2237	0.3934
**DEMETHYLATION**	40	0.35	1.19	0.2042	0.3935
**RESPONSE TO INTERLEUKIN 1**	98	0.30	1.19	0.1617	0.3940
**PHOSPHATIDYLCHOLINE BIOSYNTHETIC PROCESS**	24	0.39	1.19	0.2381	0.3983
**AMINO SUGAR METABOLIC PROCESS**	34	0.37	1.19	0.2101	0.3985
**RETINOL METABOLIC PROCESS**	26	0.38	1.19	0.2399	0.4001
**CELL DIFFERENTIATION IN HINDBRAIN**	21	-0.44	-1.39	0.0775	0.4004
**RECEPTOR SIGNALING COMPLEX SCAFFOLD ACTIVITY**	20	-0.45	-1.39	0.0950	0.4005
**CERAMIDE METABOLIC PROCESS**	59	0.33	1.19	0.2112	0.4013
**INWARD RECTIFIER POTASSIUM CHANNEL ACTIVITY**	21	-0.45	-1.39	0.0853	0.4014
**REGULATION OF METANEPHROS DEVELOPMENT**	23	-0.43	-1.39	0.0900	0.4015
**OOCYTE DIFFERENTIATION**	32	-0.41	-1.40	0.0771	0.4017
**CYTOKINE RECEPTOR BINDING**	220	-0.28	-1.40	0.0000	0.4017
**REGULATION OF SMOOTHENED SIGNALING PATHWAY**	58	-0.34	-1.39	0.0480	0.4020
**RESPONSE TO TOPOLOGICALLY INCORRECT PROTEIN**	143	-0.29	-1.40	0.0189	0.4021
**ENDOTHELIUM DEVELOPMENT**	85	0.30	1.18	0.1758	0.4023
**ACTIN MYOSIN FILAMENT SLIDING**	35	-0.38	-1.39	0.0579	0.4024
**REGULATION OF MACROPHAGE DIFFERENTIATION**	20	0.41	1.18	0.2437	0.4025
**AMINO ACID TRANSMEMBRANE TRANSPORTER ACTIVITY**	71	0.31	1.18	0.1909	0.4025
**REGULATION OF RECEPTOR INTERNALIZATION**	33	-0.39	-1.40	0.0628	0.4025
**NEGATIVE REGULATION OF CELL AGING**	15	-0.50	-1.40	0.0973	0.4025
**POSITIVE REGULATION OF EXTRINSIC APOPTOTIC SIGNALING PATHWAY**	49	0.34	1.18	0.2083	0.4026
**POSITIVE REGULATION OF KIDNEY DEVELOPMENT**	40	-0.38	-1.40	0.0679	0.4026
**TRANSCRIPTION COREPRESSOR ACTIVITY**	179	0.27	1.18	0.1444	0.4032

**CELL ACTIVATION INVOLVED IN IMMUNE RESPONSE**	115	-0.31	-1.40	0.0202	0.4032
**NEGATIVE REGULATION OF LEUKOCYTE MEDIATED IMMUNITY**	33	-0.39	-1.40	0.0857	0.4036
**CELLULAR RESPONSE TO ORGANIC CYCLIC COMPOUND**	419	0.25	1.18	0.1051	0.4038
**REGULATION OF CELLULAR CARBOHYDRATE CATABOLIC PROCESS**	37	0.35	1.18	0.2326	0.4040
**NUCLEOTIDE EXCISION REPAIR DNA INCISION**	31	0.36	1.18	0.2541	0.4041
**POSITIVE REGULATION OF CELL MORPHOGENESIS INVOLVED IN DIFFERENTIATION**	150	0.28	1.18	0.1521	0.4041
**ANTERIOR POSTERIOR PATTERN SPECIFICATION**	185	-0.28	-1.39	0.0000	0.4042
**AMMONIUM TRANSPORT**	52	0.33	1.18	0.2173	0.4042
**POSITIVE REGULATION OF CYTOPLASMIC TRANSPORT**	254	0.26	1.18	0.1319	0.4049
**REGULATION OF GENE EXPRESSION EPIGENETIC**	180	0.27	1.18	0.1532	0.4052
**ISOPRENOID BINDING**	27	-0.42	-1.39	0.0844	0.4053
**LYMPHOCYTE HOMEOSTASIS**	44	0.34	1.18	0.2318	0.4055
**REGULATION OF HORMONE BIOSYNTHETIC PROCESS**	17	0.42	1.18	0.2533	0.4056
**RESPONSE TO TEMPERATURE STIMULUS**	130	0.29	1.18	0.1507	0.4056
**NEGATIVE REGULATION OF INTRACELLULAR TRANSPORT**	123	0.29	1.18	0.1784	0.4057
**NEGATIVE REGULATION OF ANTIGEN RECEPTOR MEDIATED SIGNALING PATHWAY**	19	0.41	1.18	0.2534	0.4069
**REGULATION OF SEQUENCE SPECIFIC DNA BINDING TRANSCRIPTION FACTOR ACTIVITY**	320	0.26	1.18	0.1137	0.4081
**POSITIVE REGULATION OF MYOTUBE DIFFERENTIATION**	25	-0.43	-1.39	0.0976	0.4082
**REGULATION OF TRANSCRIPTION FROM RNA POLYMERASE III PROMOTER**	18	0.42	1.18	0.2577	0.4083
**NEGATIVE REGULATION OF PROTEIN CATABOLIC PROCESS**	100	0.29	1.18	0.1744	0.4084
**SKELETAL MUSCLE CONTRACTION**	30	-0.40	-1.39	0.0762	0.4086
**REGULATION OF PLASMA MEMBRANE ORGANIZATION**	64	0.32	1.18	0.2140	0.4086
**AXON ENSHEATHMENT**	81	0.30	1.18	0.2054	0.4092

**POSITIVE REGULATION OF EMBRYONIC DEVELOPMENT**	32	0.36	1.18	0.2306	0.4094
**POSITIVE REGULATION OF POTASSIUM ION TRANSMEMBRANE TRANSPORTER ACTIVITY**	15	0.44	1.18	0.2384	0.4095
**REGULATION OF SIGNAL TRANSDUCTION BY P53 CLASS MEDIATOR**	138	0.28	1.18	0.1667	0.4097
**REGULATION OF ACTIN CYTOSKELETON REORGANIZATION**	30	0.37	1.18	0.2467	0.4098
**REGULATION OF CARBOHYDRATE BIOSYNTHETIC PROCESS**	81	0.30	1.18	0.1817	0.4099
**BASAL LAMINA**	18	0.41	1.18	0.2469	0.4101
**CELL FATE SPECIFICATION**	69	-0.33	-1.39	0.0537	0.4102
**TRANSPORT VESICLE MEMBRANE**	122	0.29	1.18	0.1637	0.4103
**MICROTUBULE BASED PROCESS**	409	0.25	1.18	0.1047	0.4104
**REGULATION OF ALPHA AMINO 3 HYDROXY 5 METHYL 4 ISOXAZOLE PROPIONATE SELECTIVE GLUTAMATE RECEPTOR ACTIVITY**	19	-0.45	-1.38	0.1000	0.4110
**PHOSPHOLIPID TRANSPORTER ACTIVITY**	45	0.34	1.18	0.2259	0.4113
**REGULATION OF DEPHOSPHORYLATION**	134	0.28	1.18	0.1593	0.4122
**POSITIVE REGULATION OF MUSCLE TISSUE DEVELOPMENT**	52	0.33	1.18	0.2197	0.4131
**ANION HOMEOSTASIS**	36	0.35	1.18	0.2347	0.4137
**REGULATION OF OXIDATIVE STRESS INDUCED CELL DEATH**	40	-0.37	-1.38	0.0873	0.4142
**PERICENTRIOLAR MATERIAL**	17	0.41	1.17	0.2440	0.4142
**NEGATIVE REGULATION OF PROTEIN COMPLEX DISASSEMBLY**	137	0.28	1.17	0.1696	0.4156
**NEGATIVE REGULATION OF CELLULAR RESPONSE TO OXIDATIVE STRESS**	31	-0.41	-1.38	0.0820	0.4158
**PHOSPHATIDYLINOSITOL PHOSPHATE BINDING**	111	0.29	1.17	0.1789	0.4161
**ODONTOGENESIS**	101	0.29	1.17	0.1797	0.4162
**NEGATIVE REGULATION OF LYASE ACTIVITY**	26	-0.42	-1.38	0.0887	0.4163
**POSITIVE REGULATION OF DNA REPLICATION**	78	0.31	1.17	0.1864	0.4169
**HORMONE METABOLIC PROCESS**	138	0.28	1.17	0.1868	0.4171
**REGULATION OF CARBOHYDRATE CATABOLIC PROCESS**	37	0.35	1.17	0.2335	0.4172
**RESPONSE TO LIGHT STIMULUS**	243	0.26	1.17	0.1354	0.4173

**REGULATION OF ENDOPLASMIC RETICULUM STRESS INDUCED INTRINSIC APOPTOTIC SIGNALING PATHWAY**	27	0.37	1.17	0.2365	0.4174
**REGULATION OF KIDNEY DEVELOPMENT**	54	-0.35	-1.38	0.0628	0.4175
**SITE OF DOUBLE STRAND BREAK**	29	0.38	1.17	0.2405	0.4194
**CALCIUM DEPENDENT PROTEIN BINDING**	53	0.32	1.17	0.2374	0.4197
**TELOMERE MAINTENANCE VIA TELOMERE LENGTHENING**	22	0.40	1.17	0.2542	0.4198
**CELLULAR RESPONSE TO KETONE**	66	0.31	1.17	0.1912	0.4202
**REGULATION OF T HELPER 1 TYPE IMMUNE RESPONSE**	19	-0.45	-1.38	0.0947	0.4208
**REGULATION OF APPETITE**	21	0.40	1.17	0.2658	0.4214
**PROTEIN LOCALIZATION TO CENTROSOME**	15	0.43	1.17	0.2597	0.4215
**DORSAL VENTRAL PATTERN FORMATION**	84	-0.32	-1.38	0.0457	0.4222
**REGULATION OF SYNAPTIC TRANSMISSION GLUTAMATERGIC**	47	0.33	1.17	0.2166	0.4229
**REGULATION OF CYTOSKELETON ORGANIZATION**	433	0.25	1.17	0.1018	0.4229
**RNA POLYMERASE II TRANSCRIPTION FACTOR BINDING**	96	0.29	1.17	0.1841	0.4230
**POSITIVE REGULATION OF CELL CYCLE PHASE TRANSITION**	57	0.32	1.17	0.2117	0.4230
**POSITIVE REGULATION OF DNA REPAIR**	31	0.36	1.17	0.2410	0.4230
**REGULATION OF T CELL PROLIFERATION**	114	-0.30	-1.37	0.0313	0.4246
**RESPONSE TO MANGANESE ION**	16	0.43	1.17	0.2683	0.4249
**GLUCOSAMINE CONTAINING COMPOUND METABOLIC PROCESS**	20	0.40	1.17	0.2626	0.4250
**POSITIVE REGULATION OF ENDOTHELIAL CELL PROLIFERATION**	63	0.31	1.17	0.2231	0.4251
**MAMMARY GLAND LOBULE DEVELOPMENT**	16	0.42	1.17	0.2857	0.4251
**REGULATION OF MEMBRANE LIPID DISTRIBUTION**	35	0.35	1.17	0.2186	0.4266
**PROTEIN POLYMERIZATION**	57	0.32	1.17	0.2083	0.4267
**LEUKOCYTE PROLIFERATION**	72	-0.33	-1.37	0.0471	0.4278
**REGULATION OF RESPONSE TO REACTIVE OXYGEN SPECIES**	30	-0.40	-1.37	0.1007	0.4283
**NATURAL KILLER CELL ACTIVATION**	37	-0.38	-1.37	0.0539	0.4286

**INDOLALKYLAMINE METABOLIC PROCESS**	15	-0.48	-1.37	0.1192	0.4286
**REGULATION OF PEPTIDYL THREONINE PHOSPHORYLATION**	34	0.36	1.16	0.2585	0.4289
**HINDLIMB MORPHOGENESIS**	36	-0.39	-1.37	0.0758	0.4299
**LYMPHOCYTE ACTIVATION**	292	-0.27	-1.36	0.0000	0.4300
**MALE MEIOSIS**	32	-0.39	-1.37	0.0821	0.4301
**NEGATIVE REGULATION OF CHEMOKINE PRODUCTION**	16	0.42	1.16	0.2817	0.4302
**REGULATION OF NECROTIC CELL DEATH**	23	0.39	1.16	0.2721	0.4304
**POSITIVE REGULATION OF CELL CYCLE**	284	0.25	1.16	0.1358	0.4307
**ALCOHOL TRANSMEMBRANE TRANSPORTER ACTIVITY**	22	-0.42	-1.37	0.0973	0.4307
**ENSHEATHMENT OF NEURONS**	81	0.30	1.16	0.2158	0.4308
**CALCIUM ION TRANSMEMBRANE TRANSPORT**	143	-0.29	-1.37	0.0328	0.4313
**RESPONSE TO INSULIN**	193	0.26	1.16	0.1696	0.4322
**REGULATION OF RECEPTOR ACTIVITY**	112	0.29	1.16	0.2041	0.4324
**REGULATION OF HEAT GENERATION**	15	-0.47	-1.36	0.1163	0.4325
**RESPONSE TO METAL ION**	297	0.25	1.16	0.1255	0.4330
**CHROMATIN DNA BINDING**	72	0.31	1.16	0.2282	0.4339
**REGULATION OF GENERATION OF PRECURSOR METABOLITES AND ENERGY**	79	0.30	1.16	0.2031	0.4342
**GLUTAMATE RECEPTOR BINDING**	34	0.35	1.16	0.2525	0.4343
**LEUKOCYTE CELL CELL ADHESION**	222	-0.27	-1.35	0.0083	0.4351
**CELLULAR RESPONSE TO PROSTAGLANDIN E STIMULUS**	18	0.41	1.16	0.2882	0.4354
**CELLULAR RESPONSE TO LEPTIN STIMULUS**	15	0.43	1.16	0.2807	0.4354
**AROMATIC AMINO ACID FAMILY CATABOLIC PROCESS**	17	-0.46	-1.35	0.1230	0.4358
**SUBPALLIUM DEVELOPMENT**	19	0.40	1.16	0.2815	0.4360
**POSITIVE REGULATION OF B CELL ACTIVATION**	61	-0.33	-1.35	0.0593	0.4363
**GLOMERULAR EPITHELIUM DEVELOPMENT**	18	-0.45	-1.36	0.0984	0.4363
**GROWTH FACTOR ACTIVITY**	142	-0.29	-1.36	0.0293	0.4364
**DOPAMINE RECEPTOR SIGNALING PATHWAY**	26	-0.40	-1.36	0.0882	0.4370
**B CELL PROLIFERATION**	28	-0.39	-1.35	0.0850	0.4371
**PML BODY**	81	0.30	1.16	0.2131	0.4377
**ATRIAL SEPTUM DEVELOPMENT**	17	-0.46	-1.36	0.1164	0.4378

**MUSCLE FILAMENT SLIDING**	35	-0.38	-1.36	0.0693	0.4385
**CYTOSOLIC TRANSPORT**	187	0.27	1.16	0.1751	0.4386
**INFLAMMATORY RESPONSE**	381	-0.26	-1.36	0.0000	0.4386
**LYMPHOCYTE DIFFERENTIATION**	187	-0.27	-1.36	0.0300	0.4390
**FUCOSYLATION**	20	-0.43	-1.35	0.1042	0.4391
**POSITIVE REGULATION OF NF KAPPAB TRANSCRIPTION FACTOR ACTIVITY**	117	0.28	1.16	0.2102	0.4395
**SYMPORTER ACTIVITY**	134	0.27	1.16	0.1997	0.4396
**CYCLIC NUCLEOTIDE PHOSPHODIESTERASE ACTIVITY**	25	0.38	1.16	0.2571	0.4396
**UNSATURATED FATTY ACID METABOLIC PROCESS**	73	0.30	1.16	0.2130	0.4398
**CEREBRAL CORTEX NEURON DIFFERENTIATION**	19	-0.44	-1.36	0.0993	0.4400
**ANION CATION SYMPORTER ACTIVITY**	50	0.32	1.16	0.2321	0.4401
**EAR DEVELOPMENT**	185	-0.28	-1.36	0.0225	0.4402
**REGULATION OF HETEROTYPIC CELL CELL ADHESION**	18	-0.45	-1.35	0.1190	0.4406
**PRESPLICEOSOME**	19	0.39	1.16	0.2819	0.4412
**STEROID HORMONE RECEPTOR BINDING**	75	0.30	1.16	0.2214	0.4413
**INTRINSIC APOPTOTIC SIGNALING PATHWAY IN RESPONSE TO ENDOPLASMIC RETICULUM STRESS**	29	0.35	1.16	0.2509	0.4413
**REGULATION OF OSTEOBLAST PROLIFERATION**	21	-0.43	-1.36	0.0773	0.4417
**CALCIUM INDEPENDENT CELL CELL ADHESION VIA PLASMA MEMBRANE CELL ADHESION MOLECULES**	19	-0.43	-1.35	0.1136	0.4422
**NUCLEOTIDE TRANSPORT**	23	0.39	1.16	0.2623	0.4422
**T CELL DIFFERENTIATION**	117	-0.29	-1.35	0.0373	0.4425
**ACTININ BINDING**	27	0.36	1.15	0.2697	0.4433
**REGULATION OF CYSTEINE TYPE ENDOPEPTIDASE ACTIVITY**	184	0.27	1.15	0.1809	0.4434
**POSITIVE T CELL SELECTION**	19	-0.45	-1.35	0.1214	0.4435
**LYSOSOME LOCALIZATION**	20	-0.43	-1.34	0.1117	0.4436
**POSITIVE REGULATION OF ALPHA BETA T CELL DIFFERENTIATION**	32	-0.38	-1.35	0.0866	0.4438
**CELL FATE COMMITMENT**	219	-0.26	-1.35	0.0042	0.4438
**CATION CHANNEL ACTIVITY**	277	-0.26	-1.34	0.0090	0.4442
**L ASCORBIC ACID BINDING**	19	-0.44	-1.35	0.1256	0.4442
**REGULATION OF RNA SPLICING**	79	0.30	1.15	0.2215	0.4444

**EPITHELIAL CELL DIFFERENTIATION INVOLVED IN KIDNEY DEVELOPMENT**	23	-0.42	-1.34	0.0993	0.4446
**SOLUTE PROTON ANTIPORTER ACTIVITY**	16	-0.46	-1.34	0.1132	0.4448
**CELL JUNCTION ASSEMBLY**	115	0.28	1.15	0.2025	0.4462
**MAMMARY GLAND ALVEOLUS DEVELOPMENT**	16	0.42	1.15	0.2910	0.4468
**HSP90 PROTEIN BINDING**	24	-0.42	-1.34	0.1090	0.4470
**REGULATION OF INTERLEUKIN 1 PRODUCTION**	46	-0.36	-1.34	0.0652	0.4476
**NEGATIVE REGULATION OF MITOTIC CELL CYCLE**	167	0.26	1.15	0.1729	0.4478
**REGULATION OF PHOSPHATASE ACTIVITY**	105	0.28	1.15	0.2125	0.4478
**REGULATION OF ORGAN MORPHOGENESIS**	232	0.26	1.15	0.1544	0.4485
**PROTEIN DEALKYLATION**	27	0.37	1.15	0.2513	0.4486
**POSITIVE REGULATION OF ORGANELLE ASSEMBLY**	45	0.33	1.15	0.2595	0.4486
**REGULATION OF LYMPHOCYTE DIFFERENTIATION**	113	-0.29	-1.34	0.0411	0.4490
**INDOLE CONTAINING COMPOUND METABOLIC PROCESS**	24	-0.41	-1.34	0.1104	0.4491
**POSITIVE REGULATION OF CARDIAC MUSCLE HYPERTROPHY**	21	0.39	1.15	0.2639	0.4507
**OXIDOREDUCTASE ACTIVITY ACTING ON THE CH CH GROUP OF DONORS NAD OR NADP AS ACCEPTOR**	21	0.40	1.15	0.2919	0.4510
**CYCLIN DEPENDENT PROTEIN KINASE HOLOENZYME COMPLEX**	28	0.37	1.15	0.2702	0.4512
**REGULATION OF CALCIUM ION TRANSMEMBRANE TRANSPORTER ACTIVITY**	67	0.31	1.15	0.2511	0.4512
**SINGLE STRANDED DNA BINDING**	74	0.30	1.15	0.2197	0.4514
**PDZ DOMAIN BINDING**	85	0.29	1.15	0.2345	0.4515
**APOPTOTIC PROCESS INVOLVED IN DEVELOPMENT**	18	0.40	1.15	0.2712	0.4520
**SIGNAL TRANSDUCTION BY PROTEIN PHOSPHORYLATION**	365	0.25	1.15	0.1532	0.4525
**MICROTUBULE POLYMERIZATION**	24	0.38	1.15	0.3058	0.4527
**POSITIVE REGULATION OF MRNA METABOLIC PROCESS**	42	0.33	1.15	0.2472	0.4543
**RENAL SYSTEM PROCESS**	86	0.29	1.15	0.2365	0.4549
**NEGATIVE REGULATION OF STRESS ACTIVATED MAPK CASCADE**	36	0.34	1.15	0.2534	0.4552

**POSITIVE REGULATION OF RECEPTOR INTERNALIZATION**	21	-0.42	-1.33	0.1394	0.4556
**GATED CHANNEL ACTIVITY**	299	-0.26	-1.33	0.0000	0.4560
**REGULATION OF LYMPHOCYTE MEDIATED IMMUNITY**	95	-0.30	-1.33	0.0406	0.4563
**CHLORIDE CHANNEL COMPLEX**	46	-0.35	-1.33	0.0802	0.4568
**REGULATION OF HISTONE DEACETYLATION**	19	-0.43	-1.33	0.1361	0.4570
**POSITIVE REGULATION OF T CELL PROLIFERATION**	74	-0.32	-1.33	0.0678	0.4574
**BHLH TRANSCRIPTION FACTOR BINDING**	26	-0.40	-1.33	0.0919	0.4576
**AXONAL GROWTH CONE**	19	-0.44	-1.33	0.1290	0.4579
**LEUKOCYTE ACTIVATION**	354	-0.25	-1.33	0.0057	0.4582
**HUMORAL IMMUNE RESPONSE**	105	-0.29	-1.33	0.0316	0.4583
**FIBROBLAST GROWTH FACTOR RECEPTOR SIGNALING PATHWAY**	75	0.30	1.14	0.2342	0.4587
**REGULATION OF COAGULATION**	84	0.29	1.14	0.2355	0.4588
**ACTIVATING TRANSCRIPTION FACTOR BINDING**	54	-0.33	-1.33	0.0771	0.4593
**REGULATION OF RESPONSE TO BIOTIC STIMULUS**	193	0.26	1.14	0.1994	0.4594
**RESPONSE TO MORPHINE**	26	-0.40	-1.33	0.1182	0.4602
**REGULATION OF DOUBLE STRAND BREAK REPAIR VIA HOMOLOGOUS RECOMBINATION**	17	0.41	1.14	0.2759	0.4602
**PHOSPHATIDYLINOSITOL BISPHOSPHATE BINDING**	68	0.30	1.14	0.2654	0.4602
**REGULATION OF ACTIN FILAMENT BASED MOVEMENT**	32	0.36	1.14	0.2689	0.4603
**CELL CELL CONTACT ZONE**	59	0.31	1.14	0.2324	0.4604
**POSITIVE REGULATION OF MUSCLE HYPERTROPHY**	21	0.39	1.14	0.2734	0.4605
**L AMINO ACID TRANSPORT**	52	0.32	1.14	0.2558	0.4606
**PHAGOCYTIC VESICLE**	70	0.30	1.14	0.2336	0.4606
**SULFUR COMPOUND CATABOLIC PROCESS**	38	0.34	1.14	0.2914	0.4607
**PITUITARY GLAND DEVELOPMENT**	42	-0.36	-1.32	0.0904	0.4607
**DNA REPAIR COMPLEX**	33	0.36	1.14	0.2796	0.4607
**POSITIVE REGULATION OF DNA METABOLIC PROCESS**	160	0.27	1.14	0.1953	0.4608
**MICROTUBULE BINDING**	166	0.27	1.14	0.1951	0.4608
**POSITIVE REGULATION OF CYCLIN DEPENDENT PROTEIN KINASE ACTIVITY**	31	0.35	1.14	0.2500	0.4608

**REGULATION OF APOPTOTIC SIGNALING PATHWAY**	324	0.25	1.14	0.1757	0.4609
**REGULATION OF SMOOTH MUSCLE CONTRACTION**	60	0.31	1.14	0.2562	0.4611
**PEPTIDYL PROLINE MODIFICATION**	46	0.32	1.14	0.2625	0.4612
**TOLL LIKE RECEPTOR 4 SIGNALING PATHWAY**	16	0.42	1.14	0.3045	0.4613
**CLATHRIN COAT OF COATED PIT**	16	0.42	1.14	0.3035	0.4613
**ADP BINDING**	30	-0.39	-1.32	0.0991	0.4614
**MONOCARBOXYLIC ACID BIOSYNTHETIC PROCESS**	143	0.27	1.14	0.2122	0.4629
**PHOTORECEPTOR CONNECTING CILIUM**	27	0.36	1.14	0.2832	0.4629
**EXTRACELLULAR STRUCTURE ORGANIZATION**	277	-0.26	-1.32	0.0138	0.4629
**CALCIUM ION TRANSMEMBRANE TRANSPORTER ACTIVITY**	121	-0.28	-1.32	0.0280	0.4631
**POSITIVE REGULATION OF HEART RATE**	22	0.38	1.14	0.2531	0.4631
**GENE SILENCING**	164	0.27	1.14	0.1907	0.4632
**POSITIVE REGULATION OF PROTEIN POLYMERIZATION**	78	0.30	1.14	0.2474	0.4633
**NEGATIVE REGULATION OF CELL CYCLE PHASE TRANSITION**	124	0.28	1.14	0.2486	0.4635
**REGULATION OF NEUROLOGICAL SYSTEM PROCESS**	64	0.31	1.14	0.2414	0.4636
**NEGATIVE REGULATION OF PROTEIN POLYMERIZATION**	43	0.33	1.14	0.2739	0.4636
**STEROID HORMONE RECEPTOR ACTIVITY**	55	0.32	1.14	0.2813	0.4637
**SISTER CHROMATID COHESION**	101	0.29	1.14	0.2475	0.4638
**CILIARY MEMBRANE**	69	-0.31	-1.32	0.0588	0.4642
**REGULATION OF CATECHOLAMINE SECRETION**	43	-0.35	-1.32	0.0951	0.4643
**CLATHRIN COATED ENDOCYTIC VESICLE**	46	0.33	1.14	0.2824	0.4652
**PHOSPHATE ION TRANSPORT**	17	-0.45	-1.32	0.1373	0.4652
**NEGATIVE REGULATION OF ERK1 AND ERK2 CASCADE**	47	-0.35	-1.32	0.0847	0.4653
**T HELPER 1 TYPE IMMUNE RESPONSE**	16	-0.45	-1.32	0.1484	0.4654
**RNA POLYMERASE II CORE PROMOTER SEQUENCE SPECIFIC DNA BINDING**	47	-0.34	-1.32	0.0758	0.4658
**APICAL PART OF CELL**	325	0.24	1.14	0.1594	0.4658
**VOLTAGE GATED SODIUM CHANNEL ACTIVITY**	19	-0.42	-1.32	0.1394	0.4659
**MEMBRANE RAFT ORGANIZATION**	16	0.41	1.14	0.3080	0.4661

**NEGATIVE REGULATION OF MYOTUBE DIFFERENTIATION**	18	-0.44	-1.32	0.1406	0.4667
**LEADING EDGE MEMBRANE**	120	-0.29	-1.32	0.0284	0.4668
**REGULATION OF WOUND HEALING**	115	0.28	1.14	0.2100	0.4670
**INTRASPECIES INTERACTION BETWEEN ORGANISMS**	41	-0.35	-1.32	0.1002	0.4670
**REGULATION OF MAP KINASE ACTIVITY**	292	0.25	1.14	0.1840	0.4672
**POSITIVE REGULATION OF REACTIVE OXYGEN SPECIES BIOSYNTHETIC PROCESS**	41	-0.35	-1.32	0.0928	0.4672
**CGMP METABOLIC PROCESS**	23	-0.41	-1.32	0.1259	0.4676
**REGULATION OF SMAD PROTEIN IMPORT INTO NUCLEUS**	15	0.42	1.14	0.3137	0.4677
**DNA STRAND ELONGATION**	26	0.37	1.14	0.2746	0.4685
**SECONDARY ACTIVE TRANSMEMBRANE TRANSPORTER ACTIVITY**	204	0.26	1.14	0.1836	0.4686
**MICROTUBULE BASED MOVEMENT**	159	0.26	1.14	0.2072	0.4689
**REGULATION OF PROTEIN KINASE B SIGNALING**	108	0.28	1.13	0.2290	0.4690
**REGULATION OF PATHWAY RESTRICTED SMAD PROTEIN PHOSPHORYLATION**	58	0.31	1.13	0.2637	0.4691
**ZINC ION HOMEOSTASIS**	20	0.40	1.13	0.2929	0.4708
**REGULATION OF PROTEIN SERINE THREONINE KINASE ACTIVITY**	424	0.24	1.13	0.1566	0.4720
**CELLULAR RESPONSE TO LIGHT STIMULUS**	77	0.30	1.13	0.2427	0.4724
**TRANSMEMBRANE RECEPTOR PROTEIN SERINE THREONINE KINASE ACTIVITY**	16	0.41	1.13	0.3081	0.4729
**SODIUM CHANNEL COMPLEX**	16	-0.45	-1.31	0.1290	0.4731
**CARDIAC EPITHELIAL TO MESENCHYMAL TRANSITION**	24	0.37	1.13	0.2964	0.4734
**COLUMNAR CUBOIDAL EPITHELIAL CELL DEVELOPMENT**	44	0.33	1.13	0.2624	0.4744
**ALPHA BETA T CELL DIFFERENTIATION**	42	-0.35	-1.31	0.0923	0.4750
**NEGATIVE REGULATION OF CARTILAGE DEVELOPMENT**	25	-0.41	-1.31	0.1357	0.4755
**NEGATIVE REGULATION OF STRESS ACTIVATED PROTEIN KINASE SIGNALING CASCADE**	36	0.34	1.13	0.2741	0.4755
**RESPONSE TO ISOQUINOLINE ALKALOID**	26	-0.40	-1.31	0.1456	0.4758
**COLLAGEN BINDING**	56	-0.33	-1.31	0.0909	0.4764
**ENDOCRINE PROCESS**	41	-0.35	-1.31	0.0970	0.4765

**NEGATIVE REGULATION OF CHONDROCYTE DIFFERENTIATION**	19	-0.43	-1.31	0.1363	0.4768
**NEGATIVE REGULATION OF PROTEIN BINDING**	68	0.30	1.13	0.2655	0.4771
**RESPONSE TO TRANSITION METAL NANOPARTICLE**	125	0.27	1.13	0.2245	0.4773
**NEGATIVE REGULATION OF CELL ACTIVATION**	134	-0.28	-1.31	0.0364	0.4774
**PRESYNAPTIC MEMBRANE**	54	0.32	1.13	0.2590	0.4776
**HISTONE DEUBIQUITINATION**	18	0.40	1.13	0.3210	0.4776
**ENDOCARDIAL CUSHION MORPHOGENESIS**	22	0.38	1.13	0.3127	0.4777
**CHROMATIN DISASSEMBLY**	15	-0.45	-1.31	0.1514	0.4777
**REGULATION OF CYTOKINE BIOSYNTHETIC PROCESS**	87	-0.30	-1.31	0.0592	0.4777
**REGULATION OF INTRINSIC APOPTOTIC SIGNALING PATHWAY BY P53 CLASS MEDIATOR**	20	0.39	1.13	0.3102	0.4779
**POSITIVE REGULATION OF FIBROBLAST PROLIFERATION**	51	0.31	1.13	0.2593	0.4781
**CLATHRIN COATED VESICLE MEMBRANE**	63	0.30	1.13	0.2692	0.4784
**CENTROSOME CYCLE**	41	0.33	1.13	0.2843	0.4785
**REGULATION OF FIBROBLAST PROLIFERATION**	75	0.29	1.13	0.2779	0.4789
**CENTRAL NERVOUS SYSTEM NEURON DIFFERENTIATION**	156	-0.27	-1.30	0.0511	0.4790
**CYCLIC NUCLEOTIDE METABOLIC PROCESS**	55	-0.33	-1.30	0.0899	0.4791
**INTERMEDIATE FILAMENT CYTOSKELETON**	163	-0.27	-1.30	0.0383	0.4799
**ACTIVATION OF PROTEIN KINASE B ACTIVITY**	18	-0.43	-1.30	0.1326	0.4800
**POSITIVE REGULATION OF B CELL MEDIATED IMMUNITY**	23	-0.40	-1.30	0.1442	0.4801
**SIGNAL TRANSDUCTION INVOLVED IN REGULATION OF GENE EXPRESSION**	18	-0.44	-1.30	0.1176	0.4803
**PROTEIN DNA COMPLEX DISASSEMBLY**	15	-0.45	-1.30	0.1477	0.4804
**REGULATION OF SYSTEMIC ARTERIAL BLOOD PRESSURE BY RENIN ANGIOTENSIN**	20	-0.43	-1.30	0.1075	0.4809
**RESPONSE TO COCAINE**	44	-0.35	-1.30	0.0782	0.4814
**NUCLEOTIDE RECEPTOR ACTIVITY**	19	0.40	1.13	0.2917	0.4820
**HEPARAN SULFATE SULFOTRANSFERASE ACTIVITY**	15	0.42	1.13	0.3019	0.4821

**RESPONSE TO RADIATION**	357	0.24	1.13	0.1823	0.4822
**SENSORY PERCEPTION OF MECHANICAL STIMULUS**	130	-0.28	-1.30	0.0517	0.4837
**REGULATION OF CENTROSOME DUPLICATION**	29	0.35	1.12	0.2942	0.4855
**EMBRYONIC DIGIT MORPHOGENESIS**	56	0.31	1.12	0.2726	0.4861
**NEUROMUSCULAR JUNCTION DEVELOPMENT**	34	0.34	1.12	0.2878	0.4861
**REGULATION OF HOMOTYPIC CELL CELL ADHESION**	246	-0.25	-1.30	0.0095	0.4862
**DIGESTIVE SYSTEM DEVELOPMENT**	130	-0.28	-1.29	0.0512	0.4868
**UNSATURATED FATTY ACID BIOSYNTHETIC PROCESS**	44	0.33	1.12	0.2824	0.4869
**POSITIVE REGULATION OF IMMUNOGLOBULIN MEDIATED IMMUNE RESPONSE**	23	-0.40	-1.30	0.1600	0.4871
**LEUKOCYTE HOMEOSTASIS**	53	0.32	1.12	0.2939	0.4871
**INTERACTION WITH HOST**	117	0.27	1.12	0.2402	0.4872
**SOCIAL BEHAVIOR**	41	-0.35	-1.29	0.1460	0.4872
**B CELL ACTIVATION**	112	-0.28	-1.29	0.0465	0.4876
**POSITIVE REGULATION OF ADHERENS JUNCTION ORGANIZATION**	20	-0.41	-1.29	0.1332	0.4876
**REGULATION OF MONONUCLEAR CELL MIGRATION**	15	-0.45	-1.29	0.1648	0.4890
**POSITIVE REGULATION OF VASCULAR ENDOTHELIAL GROWTH FACTOR RECEPTOR SIGNALING PATHWAY**	16	-0.44	-1.29	0.1528	0.4890
**NUCLEOSOME DISASSEMBLY**	15	-0.45	-1.29	0.1535	0.4891
**REGULATION OF LAMELLIPODIUM ORGANIZATION**	32	0.35	1.12	0.3089	0.4894
**NEGATIVE REGULATION OF PHOSPHATE METABOLIC PROCESS**	490	0.24	1.12	0.1604	0.4896
**REGULATION OF SYSTEMIC ARTERIAL BLOOD PRESSURE MEDIATED BY A CHEMICAL SIGNAL**	42	0.33	1.12	0.2798	0.4897
**POSITIVE REGULATION OF ESTABLISHMENT OF PROTEIN LOCALIZATION**	441	0.24	1.12	0.1849	0.4900
**REGULATION OF RESPONSE TO CYTOKINE STIMULUS**	117	0.27	1.12	0.2514	0.4900
**FATTY ACID DERIVATIVE BIOSYNTHETIC PROCESS**	36	-0.36	-1.29	0.1096	0.4901
**KERATINIZATION**	38	-0.36	-1.29	0.1081	0.4910

**RETINAL GANGLION CELL AXON GUIDANCE**	18	0.39	1.12	0.3131	0.4910
**SENSORY PERCEPTION OF TASTE**	32	-0.37	-1.29	0.1371	0.4913
**NEGATIVE REGULATION OF INTRINSIC APOPTOTIC SIGNALING PATHWAY**	77	-0.30	-1.29	0.0548	0.4914
**NEGATIVE REGULATION OF PHOSPHORUS METABOLIC PROCESS**	490	0.24	1.12	0.1771	0.4916
**POLYSACCHARIDE BINDING**	19	0.38	1.12	0.3134	0.4917
**PRIMARY AMINO COMPOUND METABOLIC PROCESS**	15	-0.44	-1.29	0.1701	0.4919
**CELLULAR RESPONSE TO HEAT**	28	0.36	1.12	0.3046	0.4920
**PROTEIN KINASE C ACTIVITY**	15	-0.45	-1.29	0.1442	0.4922
**POSITIVE REGULATION OF MYOBLAST DIFFERENTIATION**	21	0.39	1.12	0.3028	0.4923
**MONOCARBOXYLIC ACID TRANSMEMBRANE TRANSPORTER ACTIVITY**	40	0.33	1.12	0.2815	0.4925
**TERPENOID METABOLIC PROCESS**	87	-0.29	-1.29	0.0638	0.4925
**POSITIVE REGULATION OF SEQUESTERING OF CALCIUM ION**	15	0.41	1.12	0.3277	0.4927
**CELLULAR RESPONSE TO DEXAMETHASONE STIMULUS**	24	0.37	1.12	0.3014	0.4932
**CALCIUM CHANNEL COMPLEX**	54	0.32	1.12	0.3011	0.4936
**CELL CORTEX PART**	100	0.28	1.12	0.2478	0.4943
**TRANSFORMING GROWTH FACTOR BETA RECEPTOR BINDING**	48	0.32	1.12	0.3057	0.4944
**PROTEIN DEGLYCOSYLATION**	19	0.39	1.12	0.3215	0.4944
**RESPONSE TO CALCIUM ION**	106	0.28	1.12	0.2588	0.4947
**REGULATION OF PROTEIN HOMOOLIGOMERIZATION**	16	0.41	1.12	0.3256	0.4948
**HOMEOSTASIS OF NUMBER OF CELLS**	150	0.26	1.12	0.2460	0.4950
**RECEPTOR INTERNALIZATION**	46	0.32	1.12	0.2670	0.4955
**POSITIVE REGULATION OF RESPONSE TO EXTERNAL STIMULUS**	259	-0.25	-1.28	0.0099	0.4961
**CANONICAL WNT SIGNALING PATHWAY**	92	0.28	1.12	0.2582	0.4965
**GOLGI CISTERNA MEMBRANE**	59	0.30	1.12	0.2879	0.4966
**REGULATION OF GLUCOSE TRANSPORT**	83	0.28	1.12	0.2758	0.4967
**NEGATIVE REGULATION OF PROTEIN SERINE THREONINE KINASE ACTIVITY**	118	0.27	1.12	0.2621	0.4974
**NADH METABOLIC PROCESS**	34	0.34	1.12	0.3073	0.4985
**ACTIVE ION TRANSMEMBRANE TRANSPORTER ACTIVITY**	160	0.26	1.12	0.2350	0.4987

**REGULATION OF VASODILATION**	46	0.32	1.11	0.2894	0.4989
**RETINOIC ACID RECEPTOR SIGNALING PATHWAY**	17	0.40	1.11	0.3043	0.4990
**TRANSCRIPTIONAL ACTIVATOR ACTIVITY RNA POLYMERASE II CORE PROMOTER PROXIMAL REGION SEQUENCE SPECIFIC BINDING**	205	-0.26	-1.28	0.0171	0.5001
**RUFFLE MEMBRANE**	72	-0.30	-1.28	0.0873	0.5009
**PHAGOCYTIC CUP**	16	-0.44	-1.28	0.1701	0.5013
**CHEMOKINE BINDING**	19	-0.43	-1.28	0.1597	0.5014
**NEGATIVE REGULATION OF INTRINSIC APOPTOTIC SIGNALING PATHWAY BY P53 CLASS MEDIATOR**	17	0.40	1.11	0.2958	0.5015
**REGULATION OF CHOLESTEROL METABOLIC PROCESS**	21	0.38	1.11	0.3237	0.5023
**NEGATIVE REGULATION OF RESPONSE TO EXTERNAL STIMULUS**	246	0.25	1.11	0.2358	0.5024
**INTRINSIC APOPTOTIC SIGNALING PATHWAY IN RESPONSE TO DNA DAMAGE**	57	0.30	1.11	0.2846	0.5025
**REGULATION OF HEART GROWTH**	35	0.34	1.11	0.3108	0.5027
**MESONEPHROS DEVELOPMENT**	84	0.29	1.11	0.2964	0.5030
**NUCLEOBASE METABOLIC PROCESS**	34	0.34	1.11	0.3157	0.5031
**NUCLEOTIDE PHOSPHORYLATION**	53	0.31	1.11	0.2973	0.5032
**NEGATIVE REGULATION OF NEURON DEATH**	149	0.26	1.11	0.2497	0.5039
**CYTOSKELETON DEPENDENT INTRACELLULAR TRANSPORT**	95	0.28	1.11	0.2533	0.5045
**DEOXYRIBONUCLEOTIDE METABOLIC PROCESS**	29	0.35	1.11	0.3089	0.5046
**POSITIVE REGULATION OF GLIAL CELL DIFFERENTIATION**	29	0.36	1.11	0.2991	0.5047
**CELLULAR RESPONSE TO BIOTIC STIMULUS**	145	0.26	1.11	0.2479	0.5047
**POSITIVE REGULATION OF TELOMERE MAINTENANCE VIA TELOMERE LENGTHENING**	30	0.35	1.11	0.3179	0.5049
**TRANSMEMBRANE RECEPTOR PROTEIN KINASE ACTIVITY**	78	0.29	1.11	0.2817	0.5049
**ICOSANOID BIOSYNTHETIC PROCESS**	36	-0.36	-1.28	0.1211	0.5050
**PARASYMPATHETIC NERVOUS SYSTEM DEVELOPMENT**	15	0.41	1.11	0.3488	0.5053
**PROTEIN DEMETHYLATION**	27	0.37	1.11	0.3026	0.5054

**NEGATIVE REGULATION OF COAGULATION**	47	0.32	1.11	0.2980	0.5055
**RNA DEPENDENT DNA BIOSYNTHETIC PROCESS**	19	0.39	1.11	0.3214	0.5056
**G PROTEIN COUPLED RECEPTOR BINDING**	219	-0.25	-1.28	0.0340	0.5056
**NUCLEOTIDE TRANSMEMBRANE TRANSPORTER ACTIVITY**	17	0.40	1.11	0.3345	0.5065
**FOREBRAIN REGIONALIZATION**	25	-0.39	-1.28	0.1531	0.5071
**REGULATION OF PROTEIN SUMOYLATION**	18	0.38	1.11	0.3315	0.5074
**CELL MORPHOGENESIS INVOLVED IN NEURON DIFFERENTIATION**	330	-0.24	-1.27	0.0107	0.5076
**EMBRYONIC HINDLIMB MORPHOGENESIS**	29	-0.38	-1.28	0.1393	0.5081
**AMPA GLUTAMATE RECEPTOR COMPLEX**	26	0.36	1.11	0.3077	0.5085
**REGULATION OF LEUKOCYTE MEDIATED IMMUNITY**	130	-0.27	-1.27	0.0433	0.5087
**POSITIVE REGULATION OF VASCULATURE DEVELOPMENT**	121	-0.28	-1.27	0.0625	0.5090
**RESPONSE TO ACTIVITY**	63	0.30	1.11	0.2935	0.5097
**CHEMOATTRACTANT ACTIVITY**	22	-0.40	-1.27	0.1462	0.5101
**TRANSCRIPTIONAL ACTIVATOR ACTIVITY RNA POLYMERASE II TRANSCRIPTION REGULATORY REGION SEQUENCE SPECIFIC BINDING**	288	-0.24	-1.27	0.0164	0.5102
**GLUCOCORTICOID METABOLIC PROCESS**	15	-0.44	-1.27	0.1792	0.5108
**NEUROMUSCULAR PROCESS**	90	-0.29	-1.27	0.0927	0.5109
**RESPONSE TO NERVE GROWTH FACTOR**	36	0.34	1.11	0.3197	0.5117
**CENTROSOME DUPLICATION**	28	0.35	1.11	0.3122	0.5117
**EYE PHOTORECEPTOR CELL DEVELOPMENT**	27	0.36	1.11	0.3271	0.5119
**AMINE BIOSYNTHETIC PROCESS**	18	0.39	1.11	0.3270	0.5121
**POSITIVE REGULATION OF TYPE I INTERFERON PRODUCTION**	66	0.30	1.10	0.2839	0.5139
**REGULATION OF ERBB SIGNALING PATHWAY**	75	0.29	1.10	0.2756	0.5140
**REGULATION OF INNATE IMMUNE RESPONSE**	304	0.24	1.10	0.2455	0.5145
**SPERM PRINCIPAL PIECE**	15	0.41	1.10	0.3203	0.5146
**ECTODERMAL PLACODE MORPHOGENESIS**	15	0.41	1.10	0.3223	0.5147
**HYDROGEN PEROXIDE CATABOLIC PROCESS**	15	0.41	1.10	0.3467	0.5147

CGMP BINDING	16	-0.44	-1.27	0.1942	0.5149
POSITIVE REGULATION OF PROTEIN BINDING	68	0.29	1.10	0.3151	0.5170
NEGATIVE REGULATION OF CELL CELL ADHESION	115	-0.28	-1.26	0.0775	0.5172
SOMITE DEVELOPMENT	73	-0.30	-1.26	0.1066	0.5173
VENTRICULAR SYSTEM DEVELOPMENT	24	0.36	1.10	0.3344	0.5175
APICAL JUNCTION ASSEMBLY	36	0.33	1.10	0.2927	0.5176
RECEPTOR SIGNALING PROTEIN ACTIVITY	157	0.26	1.10	0.2713	0.5176
BRUSH BORDER MEMBRANE	49	0.32	1.10	0.3060	0.5178
NEGATIVE REGULATION OF ADAPTIVE IMMUNE RESPONSE	28	-0.38	-1.26	0.1847	0.5184
POSITIVE REGULATION OF PROTEIN DEACETYLATION	16	-0.44	-1.26	0.1663	0.5197
STEROID DEHYDROGENASE ACTIVITY	22	0.37	1.10	0.3357	0.5198
POSITIVE REGULATION OF NEURON DIFFERENTIATION	289	0.24	1.10	0.2346	0.5198
ORGANIC HYDROXY COMPOUND BIOSYNTHETIC PROCESS	155	0.26	1.10	0.2575	0.5200
RESPONSE TO NUTRIENT	179	0.25	1.10	0.2676	0.5201
RECEPTOR CLUSTERING	40	-0.35	-1.27	0.1303	0.5204
SIDE OF MEMBRANE	333	-0.24	-1.27	0.0169	0.5209
N GLYCAN PROCESSING	18	0.39	1.10	0.3531	0.5212
RESPONSE TO CAFFEINE	18	0.39	1.10	0.3407	0.5215
SINGLE ORGANISM CELL ADHESION	401	-0.24	-1.27	0.0114	0.5217
NEGATIVE REGULATION OF ACTIN FILAMENT POLYMERIZATION	32	0.35	1.10	0.3333	0.5225
ECTODERMAL PLACODE FORMATION	15	0.41	1.10	0.3397	0.5230
NEGATIVE REGULATION OF MUSCLE CELL APOPTOTIC PROCESS	29	-0.37	-1.26	0.1711	0.5243
NEGATIVE REGULATION OF PROTEIN COMPLEX ASSEMBLY	86	0.29	1.10	0.2779	0.5244
POSITIVE REGULATION OF EPITHELIAL CELL PROLIFERATION	139	0.26	1.10	0.2666	0.5247
PROTEOGLYCAN BINDING	24	-0.39	-1.26	0.1675	0.5255
RESPONSE TO AMMONIUM ION	48	0.31	1.10	0.3164	0.5259
REGULATION OF ORGANIC ACID TRANSPORT	46	0.32	1.10	0.3349	0.5260
DNA CONFORMATION CHANGE	187	0.25	1.10	0.2666	0.5261
CELLULAR RESPONSE TO CALCIUM ION	41	0.32	1.10	0.3242	0.5262
L AMINO ACID TRANSMEMBRANE TRANSPORTER ACTIVITY	48	0.31	1.10	0.3242	0.5264

DENDRITE MORPHOGENESIS	38	-0.34	-1.26	0.1464	0.5265
ESTABLISHMENT OF PROTEIN LOCALIZATION TO PLASMA MEMBRANE	85	0.28	1.10	0.2958	0.5270
DE NOVO PROTEIN FOLDING	17	-0.43	-1.26	0.1915	0.5271
CELL PROLIFERATION IN FOREBRAIN	27	-0.38	-1.26	0.1757	0.5271
RECEPTOR ACTIVATOR ACTIVITY	32	0.34	1.10	0.3262	0.5272
SODIUM INDEPENDENT ORGANIC ANION TRANSPORT	15	-0.45	-1.26	0.1801	0.5275
HEAT SHOCK PROTEIN BINDING	77	0.29	1.10	0.3306	0.5277
ANTIPORTER ACTIVITY	63	0.29	1.10	0.2908	0.5278
MONOSACCHARIDE BINDING	59	-0.31	-1.26	0.1380	0.5279
B CELL DIFFERENTIATION	75	-0.30	-1.26	0.1045	0.5282
NEGATIVE REGULATION OF SECRETION	176	-0.26	-1.26	0.0444	0.5288
POSITIVE REGULATION OF LAMELLIPODIUM ORGANIZATION	22	0.36	1.09	0.3405	0.5300
POSITIVE REGULATION OF ATPASE ACTIVITY	36	0.33	1.09	0.3273	0.5307
REGULATION OF DNA BINDING	81	0.28	1.09	0.2987	0.5312
OSTEOBLAST DIFFERENTIATION	119	0.27	1.09	0.2966	0.5315
ACTIVATION OF PHOSPHOLIPASE C ACTIVITY	26	-0.38	-1.25	0.1805	0.5317
DNA DEPENDENT DNA REPLICATION MAINTENANCE OF FIDELITY	19	0.38	1.09	0.3530	0.5317
ADHERENS JUNCTION ASSEMBLY	30	0.34	1.09	0.3288	0.5317
REGULATION OF DEFENSE RESPONSE TO VIRUS BY VIRUS	26	0.36	1.09	0.3087	0.5321
TRANSMISSION OF NERVE IMPULSE	51	-0.32	-1.25	0.1025	0.5324
INOSITOL PHOSPHATE PHOSPHATASE ACTIVITY	19	0.38	1.09	0.3519	0.5326
PHOSPHATIDYLCHOLINE ACYL CHAIN REMODELING	25	0.36	1.09	0.3322	0.5326
REGULATION OF N METHYL D ASPARTATE SELECTIVE GLUTAMATE RECEPTOR ACTIVITY	15	-0.44	-1.25	0.1820	0.5327
REGULATION OF INTERFERON GAMMA PRODUCTION	81	-0.29	-1.25	0.0943	0.5328
NEURON PROJECTION MORPHOGENESIS	353	-0.23	-1.25	0.0233	0.5328
MEMBRANE DEPOLARIZATION DURING ACTION POTENTIAL	37	-0.34	-1.25	0.1390	0.5330
LEUKOCYTE MEDIATED CYTOTOXICITY	21	-0.41	-1.25	0.1725	0.5331
CYTOKINE PRODUCTION INVOLVED IN IMMUNE RESPONSE	16	-0.42	-1.25	0.1882	0.5332

**POSITIVE REGULATION OF CHROMATIN MODIFICATION**	75	-0.30	-1.25	0.0855	0.5332
**ALCOHOL BINDING**	95	-0.28	-1.25	0.0798	0.5334
**REGULATION OF MUSCLE HYPERTROPHY**	34	0.33	1.09	0.3398	0.5336
**MICROTUBULE BUNDLE FORMATION**	43	-0.34	-1.25	0.1308	0.5339
**DIENCEPHALON DEVELOPMENT**	77	-0.29	-1.25	0.0972	0.5340
**ECTODERMAL PLACODE DEVELOPMENT**	15	0.41	1.09	0.3554	0.5343
**RESPONSE TO DIETARY EXCESS**	21	0.37	1.09	0.3570	0.5347
**PROTEOGLYCAN METABOLIC PROCESS**	78	0.28	1.09	0.2945	0.5348
**PEPTIDYL CYSTEINE MODIFICATION**	19	0.38	1.09	0.3516	0.5348
**POSITIVE REGULATION OF HEART CONTRACTION**	34	0.33	1.09	0.3090	0.5349
**NECROPTOTIC PROCESS**	20	0.38	1.09	0.3243	0.5350
**RESPONSE TO LEPTIN**	18	0.39	1.09	0.3467	0.5354
**NEGATIVE REGULATION OF BLOOD VESSEL ENDOTHELIAL CELL MIGRATION**	22	-0.39	-1.25	0.1636	0.5360
**SENSORY PERCEPTION OF CHEMICAL STIMULUS**	57	-0.30	-1.25	0.1170	0.5365
**CELLULAR COMPONENT ASSEMBLY INVOLVED IN MORPHOGENESIS**	197	0.25	1.09	0.2768	0.5366
**NEGATIVE REGULATION OF ERBB SIGNALING PATHWAY**	39	0.32	1.09	0.3317	0.5368
**REGULATION OF NEUROTRANSMITTER RECEPTOR ACTIVITY**	30	-0.36	-1.25	0.1717	0.5371
**REGULATION OF ACTIN FILAMENT LENGTH**	130	0.26	1.09	0.2837	0.5373
**REGULATION OF ANTIGEN PROCESSING AND PRESENTATION**	16	-0.43	-1.24	0.1856	0.5384
**PERIPHERAL NERVOUS SYSTEM DEVELOPMENT**	64	-0.31	-1.24	0.1173	0.5388
**PROTEIN ACTIVATION CASCADE**	55	-0.31	-1.24	0.1202	0.5391
**COPPER ION BINDING**	43	0.31	1.09	0.3039	0.5393
**POTASSIUM CHANNEL COMPLEX**	90	-0.29	-1.24	0.1075	0.5395
**TRANSCRIPTION FACTOR ACTIVITY RNA POLYMERASE II DISTAL ENHANCER SEQUENCE SPECIFIC BINDING**	82	-0.29	-1.24	0.1194	0.5396
**TRABECULA FORMATION**	23	0.36	1.09	0.3257	0.5396
**POSITIVE REGULATION OF PHOSPHOLIPASE ACTIVITY**	50	-0.31	-1.24	0.1193	0.5400
**POSITIVE REGULATION OF LYMPHOCYTE DIFFERENTIATION**	66	-0.30	-1.24	0.1379	0.5415
**COPULATION**	15	-0.43	-1.24	0.1835	0.5416

**SOMITOGENESIS**	59	-0.31	-1.24	0.1429	0.5419
**NEURON PROJECTION GUIDANCE**	183	-0.25	-1.24	0.0412	0.5419
**CARBOHYDRATE DERIVATIVE TRANSPORTER ACTIVITY**	32	0.34	1.09	0.3333	0.5421
**STRUCTURAL CONSTITUENT OF CYTOSKELETON**	78	-0.29	-1.24	0.1147	0.5422
**CAMP BIOSYNTHETIC PROCESS**	16	-0.43	-1.24	0.2222	0.5423
**LEUKOTRIENE BIOSYNTHETIC PROCESS**	15	-0.44	-1.24	0.2046	0.5425
**POSITIVE REGULATION OF MITOCHONDRIAL OUTER MEMBRANE PERMEABILIZATION INVOLVED IN APOPTOTIC SIGNALING PATHWAY**	28	0.35	1.09	0.3456	0.5427
**INTEGRIN MEDIATED SIGNALING PATHWAY**	78	-0.29	-1.24	0.1371	0.5432
**RHYTHMIC PROCESS**	267	0.24	1.09	0.2619	0.5432
**REGULATION OF ANION TRANSMEMBRANE TRANSPORT**	27	0.34	1.09	0.3408	0.5433
**PROTEIN N LINKED GLYCOSYLATION**	67	0.29	1.09	0.3199	0.5433
**GLANDULAR EPITHELIAL CELL DEVELOPMENT**	18	0.39	1.09	0.3770	0.5434
**ANTIGEN PROCESSING AND PRESENTATION OF PEPTIDE ANTIGEN VIA MHC CLASS II**	71	0.29	1.08	0.3198	0.5435
**RESPONSE TO CADMIUM ION**	31	0.34	1.08	0.3624	0.5437
**DETECTION OF TEMPERATURE STIMULUS**	15	0.40	1.08	0.3512	0.5438
**REGULATION OF CELL CELL ADHESION**	313	-0.24	-1.24	0.0405	0.5440
**NEGATIVE REGULATION OF ENDOTHELIAL CELL MIGRATION**	36	-0.35	-1.24	0.1658	0.5441
**CARDIOCYTE DIFFERENTIATION**	94	0.27	1.08	0.3100	0.5441
**REGULATION OF RESPONSE TO OXIDATIVE STRESS**	57	-0.31	-1.24	0.1429	0.5442
**NEURAL TUBE PATTERNING**	32	-0.35	-1.23	0.1646	0.5447
**REGULATION OF KERATINOCYTE PROLIFERATION**	22	-0.39	-1.23	0.1908	0.5448
**NUCLEAR HETEROCHROMATIN**	29	-0.36	-1.23	0.1471	0.5449
**EXPLORATION BEHAVIOR**	22	-0.39	-1.23	0.1800	0.5452
**CELL FATE DETERMINATION**	41	-0.33	-1.24	0.1279	0.5452
**POSITIVE REGULATION OF CALCIUM ION IMPORT**	50	-0.32	-1.23	0.1440	0.5454
**NEGATIVE REGULATION OF HORMONE SECRETION**	72	-0.30	-1.23	0.1078	0.5454

**NEGATIVE REGULATION OF G PROTEIN COUPLED RECEPTOR PROTEIN SIGNALING PATHWAY**	37	-0.33	-1.23	0.1503	0.5458
**REGULATION OF INTRINSIC APOPTOTIC SIGNALING PATHWAY**	126	-0.26	-1.23	0.0572	0.5459
**SPINAL CORD DEVELOPMENT**	97	-0.28	-1.23	0.1075	0.5460
**TRANSCRIPTION FACTOR ACTIVITY DIRECT LIGAND REGULATED SEQUENCE SPECIFIC DNA BINDING**	44	0.31	1.08	0.3443	0.5461
**METHYLATED HISTONE BINDING**	43	0.31	1.08	0.3375	0.5461
**POSITIVE REGULATION OF INTRINSIC APOPTOTIC SIGNALING PATHWAY**	45	0.32	1.08	0.3442	0.5461
**PHOSPHATIDYLINOSITOL ACYL CHAIN REMODELING**	16	0.40	1.08	0.3479	0.5461
**REGULATION OF TRANSMEMBRANE TRANSPORT**	389	-0.23	-1.23	0.0062	0.5462
**POSITIVE REGULATION OF LOCOMOTION**	371	-0.23	-1.23	0.0352	0.5463
**RESPONSE TO GAMMA RADIATION**	44	0.32	1.08	0.3311	0.5464
**DIGESTIVE TRACT MORPHOGENESIS**	44	-0.32	-1.23	0.1578	0.5464
**RESPONSE TO INTERFERON ALPHA**	19	-0.41	-1.23	0.1881	0.5465
**FOREBRAIN DEVELOPMENT**	337	-0.23	-1.23	0.0266	0.5468
**MEMBRANE MICRODOMAIN**	266	0.24	1.08	0.2605	0.5469
**CLATHRIN COATED ENDOCYTIC VESICLE MEMBRANE**	33	0.33	1.08	0.3555	0.5470
**REGULATION OF NEUROBLAST PROLIFERATION**	26	-0.36	-1.23	0.1803	0.5473
**DEOXYRIBONUCLEOSIDE TRIPHOSPHATE METABOLIC PROCESS**	15	0.40	1.08	0.3421	0.5473
**POSITIVE REGULATION OF CELL ACTIVATION**	234	-0.24	-1.23	0.0412	0.5474
**PHOSPHOLIPID BINDING**	326	0.23	1.08	0.2693	0.5474
**RESPONSE TO TRANSFORMING GROWTH FACTOR BETA**	135	0.26	1.08	0.3090	0.5475
**DYNEIN COMPLEX**	24	0.36	1.08	0.3569	0.5475
**NEGATIVE REGULATION OF HEMOPOIESIS**	109	-0.27	-1.23	0.0932	0.5476
**VENTRICULAR SEPTUM DEVELOPMENT**	51	0.30	1.08	0.3328	0.5477
**STEROL BINDING**	41	-0.33	-1.23	0.1649	0.5484
**POSITIVE REGULATION OF DNA BINDING**	39	0.32	1.08	0.3444	0.5493
**CYTOSOLIC SMALL RIBOSOMAL SUBUNIT**	31	0.34	1.08	0.3540	0.5501

**REGULATION OF CELLULAR EXTRAVASATION**	21	-0.38	-1.22	0.1971	0.5501
**RECEPTOR METABOLIC PROCESS**	73	0.28	1.08	0.3025	0.5515
**CHD TYPE COMPLEX**	16	0.39	1.08	0.3704	0.5517
**NEGATIVE REGULATION OF SMOOTHENED SIGNALING PATHWAY**	23	-0.38	-1.22	0.1840	0.5517
**ACIDIC AMINO ACID TRANSPORT**	21	0.37	1.08	0.3484	0.5518
**CEREBELLAR CORTEX MORPHOGENESIS**	29	-0.36	-1.22	0.1694	0.5525
**PROTEIN DNA COMPLEX SUBUNIT ORGANIZATION**	155	0.25	1.08	0.2935	0.5527
**COLUMNAR CUBOIDAL EPITHELIAL CELL DIFFERENTIATION**	103	0.27	1.08	0.2994	0.5529
**POSITIVE REGULATION OF CYTOKINE PRODUCTION INVOLVED IN IMMUNE RESPONSE**	29	0.34	1.08	0.3475	0.5530
**REGULATION OF CENTROSOME CYCLE**	34	0.33	1.08	0.3591	0.5531
**EMBRYONIC PATTERN SPECIFICATION**	56	-0.31	-1.22	0.1264	0.5531
**DNA CATABOLIC PROCESS**	24	0.36	1.08	0.3596	0.5541
**POSITIVE REGULATION OF TRANSMEMBRANE RECEPTOR PROTEIN SERINE THREONINE KINASE SIGNALING PATHWAY**	93	0.27	1.08	0.3170	0.5548
**STEROID METABOLIC PROCESS**	185	0.25	1.08	0.2922	0.5565
**PHOTORECEPTOR OUTER SEGMENT**	61	-0.30	-1.22	0.1441	0.5576
**TRABECULA MORPHOGENESIS**	38	0.33	1.08	0.3492	0.5588
**POSITIVE REGULATION OF LYMPHOCYTE MEDIATED IMMUNITY**	59	-0.30	-1.22	0.1390	0.5594
**HISTONE ACETYLTRANSFERASE BINDING**	26	-0.37	-1.22	0.2086	0.5595
**POSITIVE REGULATION OF CELL JUNCTION ASSEMBLY**	23	-0.38	-1.22	0.2128	0.5599
**POSITIVE REGULATION OF CELL CELL ADHESION**	192	-0.25	-1.22	0.0769	0.5599
**CENTROMERE COMPLEX ASSEMBLY**	30	-0.36	-1.21	0.1769	0.5599
**REGULATION OF VASCULAR ENDOTHELIAL GROWTH FACTOR RECEPTOR SIGNALING PATHWAY**	27	-0.36	-1.21	0.1859	0.5600
**EPIDERMIS MORPHOGENESIS**	26	-0.37	-1.21	0.2063	0.5603
**SYNAPTIC SIGNALING**	392	-0.23	-1.21	0.0440	0.5605
**POSITIVE REGULATION OF LEUKOCYTE DIFFERENTIATION**	112	-0.27	-1.21	0.0836	0.5606
**REGULATION OF CELL CYCLE G2 M PHASE TRANSITION**	50	0.30	1.07	0.3499	0.5607

**REGULATION OF CELLULAR SENESCENCE**	24	-0.37	-1.22	0.1843	0.5609
**ANION ANION ANTIPORTER ACTIVITY**	20	0.36	1.07	0.3687	0.5609
**ZYMOGEN ACTIVATION**	97	0.27	1.07	0.3304	0.5612
**REGULATION OF RESPIRATORY GASEOUS EXCHANGE**	22	-0.38	-1.21	0.2009	0.5613
**PROTEIN TRANSPORT ALONG MICROTUBULE**	23	-0.37	-1.22	0.2117	0.5614
**PROTEIN IMPORT INTO NUCLEUS TRANSLOCATION**	27	-0.36	-1.21	0.1827	0.5615
**POSITIVE REGULATION OF INFLAMMATORY RESPONSE**	98	-0.27	-1.22	0.1185	0.5617
**REGULATION OF CYTOKINE PRODUCTION INVOLVED IN IMMUNE RESPONSE**	49	-0.30	-1.21	0.1769	0.5621
**CELLULAR HORMONE METABOLIC PROCESS**	81	0.28	1.07	0.3042	0.5621
**POSITIVE REGULATION OF LIPID BIOSYNTHETIC PROCESS**	62	-0.30	-1.21	0.1875	0.5622
**ANDROGEN METABOLIC PROCESS**	24	-0.37	-1.21	0.1873	0.5622
**KINESIN COMPLEX**	47	-0.32	-1.20	0.1654	0.5622
**DNA METHYLATION INVOLVED IN GAMETE GENERATION**	18	-0.40	-1.21	0.1995	0.5623
**PEPTIDE HORMONE RECEPTOR BINDING**	17	-0.41	-1.22	0.1905	0.5623
**REGULATION OF ACTIN FILAMENT BASED PROCESS**	279	0.24	1.07	0.2929	0.5624
**POSITIVE REGULATION OF T HELPER CELL DIFFERENTIATION**	16	-0.42	-1.20	0.2250	0.5625
**REGULATION OF HEMOPOIESIS**	267	-0.23	-1.21	0.0563	0.5626
**NUCLEOBASE BIOSYNTHETIC PROCESS**	15	0.40	1.07	0.3735	0.5628
**KERATINOCYTE DIFFERENTIATION**	82	-0.28	-1.21	0.1366	0.5629
**PHOTOTRANSDUCTION**	40	-0.33	-1.22	0.1557	0.5629
**REGULATION OF RECEPTOR BINDING**	15	0.40	1.07	0.3590	0.5631
**OXIDOREDUCTASE ACTIVITY ACTING ON PAIRED DONORS WITH INCORPORATION OR REDUCTION OF MOLECULAR OXYGEN**	105	-0.27	-1.21	0.1137	0.5631
**NEGATIVE REGULATION OF LEUKOCYTE DIFFERENTIATION**	72	-0.29	-1.21	0.1269	0.5632
**POSITIVE REGULATION OF GLUCOSE TRANSPORT**	35	-0.33	-1.21	0.1700	0.5633
**GERM CELL NUCLEUS**	20	-0.39	-1.21	0.2105	0.5636
**POSITIVE REGULATION OF CELL ADHESION**	312	-0.23	-1.21	0.0459	0.5641

**AROMATIC AMINO ACID FAMILY METABOLIC PROCESS**	25	-0.36	-1.21	0.1760	0.5642
**RESPONSE TO SALT STRESS**	18	-0.40	-1.21	0.2028	0.5643
**K63 LINKED POLYUBIQUITIN BINDING**	15	0.40	1.07	0.3724	0.5644
**CELL CELL JUNCTION ASSEMBLY**	66	0.29	1.07	0.3245	0.5646
**METALLOPEPTIDASE ACTIVITY**	166	0.25	1.07	0.3356	0.5649
**REGULATION OF MUSCLE CONTRACTION**	142	0.26	1.07	0.3186	0.5651
**NEGATIVE REGULATION OF SIGNAL TRANSDUCTION IN ABSENCE OF LIGAND**	29	-0.35	-1.21	0.1881	0.5652
**NEGATIVE REGULATION OF NUCLEOTIDE METABOLIC PROCESS**	59	-0.30	-1.20	0.1799	0.5654
**MRNA SPLICE SITE SELECTION**	16	0.39	1.07	0.3759	0.5660
**REGULATION OF NIK NF KAPPAB SIGNALING**	34	0.33	1.07	0.3585	0.5662
**EMBRYO IMPLANTATION**	36	-0.33	-1.20	0.1933	0.5664
**ANTIGEN PROCESSING AND PRESENTATION OF PEPTIDE OR POLYSACCHARIDE ANTIGEN VIA MHC CLASS II**	71	0.29	1.07	0.3370	0.5669
**CELLULAR RESPONSE TO RADIATION**	115	0.26	1.07	0.3247	0.5669
**CYTOPLASMIC REGION**	252	0.24	1.07	0.3053	0.5670
**NEGATIVE REGULATION OF MAPK CASCADE**	135	0.26	1.07	0.3353	0.5670
**REGULATION OF HORMONE LEVELS**	423	0.23	1.07	0.2909	0.5670
**ALPHA BETA T CELL ACTIVATION**	48	-0.31	-1.20	0.1723	0.5671
**POSITIVE REGULATION OF TYROSINE PHOSPHORYLATION OF STAT5 PROTEIN**	15	0.40	1.07	0.3714	0.5671
**REGULATION OF CHONDROCYTE DIFFERENTIATION**	44	-0.32	-1.20	0.1578	0.5674
**ENDOTHELIAL CELL MIGRATION**	52	-0.31	-1.20	0.1886	0.5674
**RESPONSE TO PLATELET DERIVED GROWTH FACTOR**	15	-0.43	-1.20	0.2169	0.5677
**RESPONSE TO WATER**	15	-0.42	-1.20	0.2437	0.5678
**NEGATIVE REGULATION OF INTRINSIC APOPTOTIC SIGNALING PATHWAY IN RESPONSE TO DNA DAMAGE**	23	-0.37	-1.20	0.2118	0.5683
**SENSORY PERCEPTION**	427	-0.22	-1.20	0.0136	0.5684
**REGULATION OF NITRIC OXIDE BIOSYNTHETIC PROCESS**	47	-0.31	-1.20	0.1851	0.5686
**CHAPERONE MEDIATED PROTEIN FOLDING**	40	-0.32	-1.20	0.1787	0.5687
**SMAD PROTEIN SIGNAL TRANSDUCTION**	55	-0.30	-1.20	0.1550	0.5688

**NONMOTILE PRIMARY CILIUM**	114	-0.26	-1.20	0.0952	0.5696
**POSITIVE REGULATION OF CALCIUM ION TRANSPORT**	99	-0.27	-1.19	0.1415	0.5698
**NEGATIVE REGULATION OF NEURON DIFFERENTIATION**	178	-0.24	-1.19	0.0952	0.5699
**PATTERN SPECIFICATION PROCESS**	384	-0.22	-1.19	0.0252	0.5705
**LEUKOCYTE DIFFERENTIATION**	262	-0.23	-1.19	0.0351	0.5705
**RESPONSE TO IMMOBILIZATION STRESS**	20	-0.38	-1.19	0.2244	0.5705
**MALE MEIOSIS I**	15	-0.41	-1.19	0.2387	0.5706
**METANEPHROS DEVELOPMENT**	78	-0.28	-1.20	0.1441	0.5707
**NEGATIVE REGULATION OF EXTRINSIC APOPTOTIC SIGNALING PATHWAY IN ABSENCE OF LIGAND**	29	-0.35	-1.20	0.1995	0.5707
**POSITIVE REGULATION OF TUMOR NECROSIS FACTOR SUPERFAMILY CYTOKINE PRODUCTION**	54	-0.31	-1.20	0.1760	0.5708
**DENDRITIC SPINE ORGANIZATION**	17	-0.40	-1.19	0.2477	0.5709
**SOLUTE CATION SYMPORTER ACTIVITY**	93	-0.27	-1.19	0.1346	0.5710
**SYNAPSE ASSEMBLY**	65	-0.29	-1.19	0.1667	0.5710
**REGULATION OF MORPHOGENESIS OF A BRANCHING STRUCTURE**	53	-0.30	-1.19	0.1816	0.5712
**POSITIVE REGULATION OF AMINE TRANSPORT**	32	-0.35	-1.19	0.2228	0.5713
**NUCLEAR CHROMATIN**	229	0.24	1.07	0.3085	0.5714
**INTRACILIARY TRANSPORT**	23	-0.37	-1.19	0.2273	0.5716
**NEGATIVE REGULATION OF PROTEIN PROCESSING**	28	-0.35	-1.19	0.2117	0.5717
**REGULATION OF GLYCOPROTEIN METABOLIC PROCESS**	33	0.33	1.07	0.3601	0.5724
**REGULATION OF ENDOTHELIAL CELL MIGRATION**	110	-0.26	-1.19	0.1137	0.5737
**CHROMATIN**	352	0.23	1.07	0.2848	0.5739
**REGULATION OF TELOMERE MAINTENANCE VIA TELOMERE LENGTHENING**	44	0.31	1.07	0.3526	0.5740
**NEGATIVE REGULATION OF LEUKOCYTE MIGRATION**	28	0.34	1.07	0.3694	0.5745
**ESTABLISHMENT OF EPITHELIAL CELL POLARITY**	21	0.36	1.07	0.3699	0.5750
**NEGATIVE REGULATION OF LIPID METABOLIC PROCESS**	61	0.29	1.07	0.3510	0.5751
**FEMALE SEX DIFFERENTIATION**	106	-0.26	-1.19	0.1443	0.5752

**REGULATION OF TUMOR NECROSIS FACTOR SUPERFAMILY CYTOKINE PRODUCTION**	94	-0.27	-1.19	0.1344	0.5754
**MULTI MULTICELLULAR ORGANISM PROCESS**	185	-0.24	-1.19	0.0837	0.5756
**REGULATION OF INTERLEUKIN 5 PRODUCTION**	17	0.39	1.06	0.3636	0.5771
**PURINERGIC NUCLEOTIDE RECEPTOR SIGNALING PATHWAY**	20	0.37	1.06	0.3933	0.5772
**TOR SIGNALING**	15	0.40	1.06	0.3933	0.5773
**CENTRIOLAR SATELLITE**	20	0.37	1.06	0.3768	0.5780
**MICROTUBULE ASSOCIATED COMPLEX**	108	0.27	1.06	0.3438	0.5781
**POSITIVE REGULATION OF MAPK CASCADE**	415	0.22	1.06	0.2937	0.5782
**POSITIVE REGULATION OF RESPONSE TO CYTOKINE STIMULUS**	29	0.34	1.06	0.3704	0.5783
**MUSCLE HYPERTROPHY**	28	0.34	1.06	0.3780	0.5783
**NEGATIVE REGULATION OF EPITHELIAL CELL APOPTOTIC PROCESS**	34	0.33	1.06	0.3901	0.5800
**RESPONSE TO COLD**	40	0.31	1.06	0.3618	0.5800
**CLATHRIN ADAPTOR COMPLEX**	25	0.35	1.06	0.3882	0.5802
**ORGAN INDUCTION**	16	0.39	1.06	0.3948	0.5802
**POSITIVE REGULATION OF DEFENSE RESPONSE**	316	0.23	1.06	0.3207	0.5803
**SH2 DOMAIN BINDING**	26	-0.35	-1.18	0.2253	0.5804
**REGULATION OF PHOSPHOLIPASE ACTIVITY**	61	-0.29	-1.18	0.1888	0.5804
**TRANSLATION ELONGATION FACTOR ACTIVITY**	18	0.38	1.06	0.3986	0.5805
**LRR DOMAIN BINDING**	15	0.39	1.06	0.3759	0.5806
**DENDRITIC CELL DIFFERENTIATION**	29	-0.34	-1.18	0.2161	0.5810
**CELLULAR RESPONSE TO HORMONE STIMULUS**	489	0.22	1.06	0.3014	0.5818
**NEGATIVE REGULATION OF APOPTOTIC SIGNALING PATHWAY**	181	0.25	1.06	0.3250	0.5819
**NEGATIVE REGULATION OF WOUND HEALING**	56	0.29	1.06	0.3514	0.5820
**EXTRACELLULAR MATRIX BINDING**	48	-0.30	-1.18	0.1917	0.5829
**MAINTENANCE OF CELL NUMBER**	126	0.26	1.06	0.3526	0.5829
**CELL ACTIVATION**	494	-0.22	-1.18	0.0365	0.5832
**REGULATION OF LEUKOCYTE DIFFERENTIATION**	202	-0.24	-1.18	0.0870	0.5836
**MALE GERM CELL NUCLEUS**	16	-0.41	-1.18	0.2483	0.5837
**REGULATION OF AXON GUIDANCE**	39	-0.32	-1.18	0.2030	0.5839

**NEGATIVE REGULATION OF RESPONSE TO BIOTIC STIMULUS**	26	0.34	1.06	0.3873	0.5846
**NEUROPILIN BINDING**	15	-0.41	-1.18	0.2261	0.5848
**ARTERY MORPHOGENESIS**	49	0.30	1.06	0.3575	0.5848
**REGULATION OF ATPASE ACTIVITY**	52	0.30	1.06	0.3899	0.5849
**SKELETAL MUSCLE ORGAN DEVELOPMENT**	124	-0.25	-1.18	0.1329	0.5853
**TRIGLYCERIDE LIPASE ACTIVITY**	17	0.38	1.06	0.3949	0.5853
**CELL CORTEX REGION**	15	-0.41	-1.18	0.2318	0.5864
**REGULATION OF ESTABLISHMENT OF PROTEIN LOCALIZATION TO PLASMA MEMBRANE**	43	0.31	1.06	0.3581	0.5873
**ZINC II ION TRANSPORT**	25	0.34	1.06	0.3761	0.5874
**RHO PROTEIN SIGNAL TRANSDUCTION**	43	-0.31	-1.17	0.1814	0.5884
**COCHLEA DEVELOPMENT**	38	-0.32	-1.18	0.1933	0.5886
**POSITIVE REGULATION OF TYROSINE PHOSPHORYLATION OF STAT3 PROTEIN**	34	-0.34	-1.18	0.2163	0.5886
**AMINE CATABOLIC PROCESS**	19	-0.38	-1.18	0.2257	0.5887
**TISSUE MIGRATION**	75	-0.27	-1.18	0.1631	0.5887
**RESPONSE TO ALKALOID**	128	-0.25	-1.18	0.1337	0.5894
**LYMPHOCYTE COSTIMULATION**	55	-0.30	-1.18	0.1763	0.5903
**CATION TRANSPORTING ATPASE ACTIVITY**	56	0.29	1.06	0.3555	0.5905
**MAMMARY GLAND MORPHOGENESIS**	39	-0.32	-1.17	0.2085	0.5905
**NEGATIVE REGULATION OF ANION TRANSPORT**	31	0.34	1.06	0.3789	0.5907
**ALCOHOL BIOSYNTHETIC PROCESS**	98	0.27	1.05	0.3602	0.5908
**POSITIVE REGULATION OF ORGAN GROWTH**	35	0.32	1.06	0.4047	0.5909
**TRANSFORMING GROWTH FACTOR BETA RECEPTOR SIGNALING PATHWAY**	92	0.26	1.05	0.3613	0.5911
**OMEGA PEPTIDASE ACTIVITY**	15	0.39	1.05	0.3856	0.5912
**PHENOL CONTAINING COMPOUND METABOLIC PROCESS**	70	0.28	1.05	0.3509	0.5912
**ORGAN MATURATION**	16	-0.41	-1.17	0.2368	0.5914
**JAK STAT CASCADE**	43	-0.31	-1.17	0.1924	0.5914
**PEPTIDE TRANSPORT**	67	-0.28	-1.17	0.1750	0.5922
**ANDROGEN RECEPTOR BINDING**	37	0.32	1.05	0.3577	0.5924
**NOSE DEVELOPMENT**	15	-0.41	-1.17	0.2512	0.5929
**ION ANTIPORTER ACTIVITY**	43	0.30	1.05	0.3690	0.5930
**MYOSIN FILAMENT**	20	-0.38	-1.17	0.2447	0.5932
**BODY FLUID SECRETION**	67	-0.28	-1.17	0.1630	0.5935

**CENTRAL NERVOUS SYSTEM PROJECTION NEURON AXONOGENESIS**	21	-0.38	-1.17	0.2488	0.5936
**POSITIVE REGULATION OF SEQUENCE SPECIFIC DNA BINDING TRANSCRIPTION FACTOR ACTIVITY**	206	0.24	1.05	0.3712	0.5940
**REGULATION OF ASTROCYTE DIFFERENTIATION**	26	-0.35	-1.17	0.2402	0.5941
**B CELL RECEPTOR SIGNALING PATHWAY**	31	-0.34	-1.17	0.2081	0.5941
**DRUG METABOLIC PROCESS**	19	0.37	1.05	0.3975	0.5943
**SMOOTH MUSCLE TISSUE DEVELOPMENT**	15	0.39	1.05	0.3950	0.5947
**NEGATIVE REGULATION OF CELL PROJECTION ORGANIZATION**	133	-0.25	-1.17	0.1628	0.5948
**REGULATION OF AMINE TRANSPORT**	70	-0.28	-1.17	0.1821	0.5951
**POSITIVE REGULATION OF BIOMINERAL TISSUE DEVELOPMENT**	36	-0.33	-1.17	0.2187	0.5951
**NEGATIVE REGULATION OF PROTEIN MATURATION**	28	-0.35	-1.17	0.2494	0.5954
**METAL ION TRANSMEMBRANE TRANSPORTER ACTIVITY**	393	-0.22	-1.17	0.0387	0.5956
**POSITIVE REGULATION OF PROTEIN EXPORT FROM NUCLEUS**	17	-0.39	-1.17	0.2511	0.5958
**MYOSIN COMPLEX**	60	-0.29	-1.17	0.1902	0.5958
**REGULATION OF DENDRITIC SPINE DEVELOPMENT**	54	0.29	1.05	0.3910	0.5962
**CELL CELL ADHESION VIA PLASMA MEMBRANE ADHESION MOLECULES**	157	-0.24	-1.17	0.1215	0.5963
**BLOOD VESSEL REMODELING**	30	0.33	1.05	0.3970	0.5964
**POSITIVE REGULATION OF CARBOHYDRATE METABOLIC PROCESS**	68	0.28	1.05	0.3756	0.5964
**STAT CASCADE**	43	-0.31	-1.17	0.2264	0.5965
**ORGANIC ACID SODIUM SYMPORTER ACTIVITY**	27	0.34	1.05	0.3752	0.5967
**TRANSCRIPTIONAL REPRESSOR ACTIVITY RNA POLYMERASE II ACTIVATING TRANSCRIPTION FACTOR BINDING**	52	-0.29	-1.16	0.2102	0.5972
**POSITIVE REGULATION OF VASCULAR ENDOTHELIAL GROWTH FACTOR PRODUCTION**	24	0.35	1.05	0.4043	0.5972
**LYSINE N METHYLTRANSFERASE ACTIVITY**	46	0.30	1.05	0.3968	0.5972
**SKIN DEVELOPMENT**	185	-0.23	-1.16	0.1176	0.5990

**REGULATION OF ENERGY HOMEOSTASIS**	17	0.37	1.05	0.3869	0.5991
**POSITIVE REGULATION OF NEUROTRANSMITTER TRANSPORT**	16	0.38	1.05	0.4178	0.6015
**ORGAN GROWTH**	63	-0.28	-1.16	0.1718	0.6026
**REGULATION OF ENDOCRINE PROCESS**	43	-0.31	-1.16	0.2272	0.6027
**POSITIVE REGULATION OF RELEASE OF SEQUESTERED CALCIUM ION INTO CYTOSOL**	35	-0.31	-1.16	0.2317	0.6028
**NEGATIVE REGULATION OF CELL ADHESION**	192	-0.24	-1.16	0.0898	0.6033
**LOCOMOTORY BEHAVIOR**	165	-0.24	-1.16	0.1255	0.6034
**RNA POLYMERASE II TRANSCRIPTION COACTIVATOR ACTIVITY**	35	-0.33	-1.16	0.2293	0.6041
**REGULATION OF SYNAPSE ORGANIZATION**	105	0.26	1.05	0.3752	0.6045
**CALCIUM ION REGULATED EXOCYTOSIS OF NEUROTRANSMITTER**	31	-0.34	-1.16	0.2123	0.6052
**NEGATIVE REGULATION OF POTASSIUM ION TRANSMEMBRANE TRANSPORT**	21	-0.37	-1.16	0.2579	0.6053
**REGULATION OF SKELETAL MUSCLE TISSUE DEVELOPMENT**	47	-0.31	-1.16	0.2109	0.6054
**CEREBELLAR PURKINJE CELL LAYER DEVELOPMENT**	22	-0.37	-1.16	0.2411	0.6055
**MYELOID CELL DEVELOPMENT**	35	-0.32	-1.16	0.2143	0.6057
**VENTRAL SPINAL CORD DEVELOPMENT**	43	-0.31	-1.16	0.2464	0.6062
**POSITIVE REGULATION OF PRODUCTION OF MOLECULAR MEDIATOR OF IMMUNE RESPONSE**	54	-0.30	-1.16	0.2280	0.6067
**POSITIVE REGULATION OF REACTIVE OXYGEN SPECIES METABOLIC PROCESS**	73	-0.28	-1.15	0.2046	0.6082
**FOREBRAIN CELL MIGRATION**	58	-0.29	-1.15	0.2099	0.6087
**HYALURONAN METABOLIC PROCESS**	25	-0.35	-1.15	0.2517	0.6095
**REGULATION OF NEURON MIGRATION**	28	0.33	1.04	0.3798	0.6100
**DIOXYGENASE ACTIVITY**	79	0.27	1.04	0.3828	0.6100
**RIBONUCLEOSIDE DIPHOSPHATE METABOLIC PROCESS**	57	0.29	1.04	0.3956	0.6103
**HEART DEVELOPMENT**	435	0.22	1.04	0.3381	0.6105
**SODIUM ION HOMEOSTASIS**	25	0.35	1.04	0.3989	0.6111
**POSITIVE REGULATION OF MICROTUBULE POLYMERIZATION**	18	0.37	1.04	0.3907	0.6116
**MYELINATION IN PERIPHERAL NERVOUS SYSTEM**	21	0.35	1.04	0.4030	0.6116

**RESPONSE TO CORTICOSTERONE**	25	-0.35	-1.15	0.2536	0.6123
**NEGATIVE REGULATION OF MAP KINASE ACTIVITY**	69	0.28	1.04	0.3802	0.6129
**POSITIVE REGULATION OF IMMUNOGLOBULIN PRODUCTION**	25	-0.35	-1.15	0.2518	0.6132
**METANEPHRIC NEPHRON MORPHOGENESIS**	21	-0.35	-1.15	0.2586	0.6138
**DNA PACKAGING**	123	-0.25	-1.15	0.1783	0.6147
**CHROMATIN ASSEMBLY OR DISASSEMBLY**	109	-0.25	-1.15	0.1869	0.6156
**PHOSPHATE ION TRANSMEMBRANE TRANSPORTER ACTIVITY**	16	-0.40	-1.15	0.3064	0.6156
**NEGATIVE REGULATION OF RESPONSE TO ENDOPLASMIC RETICULUM STRESS**	33	0.32	1.04	0.3972	0.6160
**CYSTEINE TYPE ENDOPEPTIDASE INHIBITOR ACTIVITY INVOLVED IN APOPTOTIC PROCESS**	19	-0.37	-1.15	0.2740	0.6161
**SEGMENTATION**	85	-0.27	-1.15	0.2026	0.6162
**OVARIAN FOLLICLE DEVELOPMENT**	54	-0.29	-1.15	0.2119	0.6165
**ENDOCYTIC VESICLE MEMBRANE**	121	0.25	1.04	0.3858	0.6165
**POSITIVE REGULATION OF INTERFERON GAMMA PRODUCTION**	57	-0.29	-1.15	0.2294	0.6165
**PROTEIN LIPID COMPLEX**	32	0.32	1.04	0.3898	0.6165
**SPINDLE CHECKPOINT**	24	0.35	1.04	0.4178	0.6165
**MESENCHYMAL TO EPITHELIAL TRANSITION**	15	-0.40	-1.15	0.2931	0.6167
**REGULATION OF HORMONE METABOLIC PROCESS**	23	0.34	1.04	0.3997	0.6168
**REGULATION OF STAT CASCADE**	126	-0.25	-1.15	0.1433	0.6171
**REGULATION OF CALCIUM MEDIATED SIGNALING**	68	0.28	1.04	0.4053	0.6174
**REGULATION OF JAK STAT CASCADE**	126	-0.25	-1.15	0.1464	0.6175
**MITOTIC G2 M TRANSITION CHECKPOINT**	16	0.38	1.04	0.4097	0.6176
**REGULATION OF VASCULAR PERMEABILITY**	26	-0.35	-1.15	0.2730	0.6176
**IMMUNOGLOBULIN PRODUCTION**	33	0.32	1.04	0.4157	0.6177
**MOTOR ACTIVITY**	100	-0.25	-1.15	0.1941	0.6180
**GLYCOSAMINOGLYCAN BINDING**	179	-0.24	-1.15	0.1288	0.6181
**REGULATION OF ACTIVIN RECEPTOR SIGNALING PATHWAY**	20	0.37	1.04	0.4125	0.6214
**CYTOKINE RECEPTOR ACTIVITY**	83	-0.27	-1.14	0.2059	0.6242
**ASSOCIATIVE LEARNING**	68	-0.27	-1.14	0.2203	0.6247

**REGULATION OF CYTOSOLIC CALCIUM ION CONCENTRATION**	186	-0.23	-1.14	0.1434	0.6249
**REGULATION OF TRANSFORMING GROWTH FACTOR BETA PRODUCTION**	23	0.35	1.04	0.3989	0.6250
**TRANSMEMBRANE RECEPTOR PROTEIN TYROSINE KINASE SIGNALING PATHWAY**	459	0.22	1.04	0.3811	0.6252
**POSITIVE REGULATION OF G PROTEIN COUPLED RECEPTOR PROTEIN SIGNALING PATHWAY**	23	-0.35	-1.14	0.2699	0.6255
**HEART VALVE DEVELOPMENT**	33	-0.32	-1.14	0.2537	0.6255
**REGULATION OF INSULIN LIKE GROWTH FACTOR RECEPTOR SIGNALING PATHWAY**	20	-0.36	-1.14	0.2768	0.6257
**REGULATION OF ENDOTHELIAL CELL DIFFERENTIATION**	27	0.33	1.04	0.4125	0.6257
**DETECTION OF LIGHT STIMULUS**	54	-0.29	-1.14	0.2245	0.6259
**ORGANIC HYDROXY COMPOUND TRANSMEMBRANE TRANSPORTER ACTIVITY**	53	0.29	1.04	0.3957	0.6261
**ADULT BEHAVIOR**	122	-0.24	-1.14	0.1846	0.6262
**CELLULAR BIOGENIC AMINE CATABOLIC PROCESS**	19	-0.38	-1.14	0.2854	0.6267
**NEGATIVE REGULATION OF IMMUNE RESPONSE**	94	-0.25	-1.13	0.1854	0.6268
**NEGATIVE REGULATION OF DENDRITE DEVELOPMENT**	24	-0.35	-1.14	0.2824	0.6269
**POSITIVE REGULATION OF SMOOTHENED SIGNALING PATHWAY**	23	-0.35	-1.14	0.2957	0.6270
**CARDIAC ATRIUM DEVELOPMENT**	28	-0.34	-1.13	0.2736	0.6273
**NUCLEUS LOCALIZATION**	20	-0.36	-1.13	0.2998	0.6274
**EMBRYONIC ORGAN MORPHOGENESIS**	261	-0.22	-1.13	0.1033	0.6275
**KERATIN FILAMENT**	50	-0.29	-1.14	0.2367	0.6275
**EXTRACELLULAR MATRIX DISASSEMBLY**	67	-0.28	-1.13	0.2325	0.6275
**RETINOL DEHYDROGENASE ACTIVITY**	15	-0.39	-1.14	0.2933	0.6275
**RUFFLE**	143	-0.24	-1.13	0.1855	0.6276
**PHAGOCYTOSIS ENGULFMENT**	16	-0.38	-1.14	0.2757	0.6277
**DEFENSE RESPONSE TO OTHER ORGANISM**	335	-0.22	-1.13	0.1067	0.6277
**ACTIN MONOMER BINDING**	23	-0.35	-1.13	0.2938	0.6277
**NEGATIVE REGULATION OF IMMUNE EFFECTOR PROCESS**	79	-0.27	-1.13	0.2175	0.6278
**MAMMARY GLAND DEVELOPMENT**	113	-0.25	-1.14	0.1698	0.6278
**OVULATION CYCLE**	104	-0.25	-1.14	0.1863	0.6280

**VIRUS RECEPTOR ACTIVITY**	61	-0.28	-1.13	0.2158	0.6281
**IRON ION BINDING**	104	-0.25	-1.13	0.1787	0.6283
**ENDOCRINE SYSTEM DEVELOPMENT**	118	-0.24	-1.13	0.1749	0.6284
**POSITIVE REGULATION OF JAK STAT CASCADE**	64	-0.27	-1.13	0.2203	0.6285
**POSITIVE REGULATION OF NEUROBLAST PROLIFERATION**	19	-0.38	-1.14	0.3022	0.6285
**MYELOID LEUKOCYTE ACTIVATION**	87	-0.26	-1.14	0.1914	0.6287
**METANEPHROS MORPHOGENESIS**	28	-0.33	-1.13	0.2902	0.6291
**POSITIVE REGULATION OF CHOLESTEROL TRANSPORT**	16	0.38	1.03	0.4234	0.6293
**LEUKOCYTE DEGRANULATION**	27	-0.34	-1.14	0.2754	0.6294
**GROWTH**	371	0.22	1.03	0.3691	0.6296
**REGULATION OF CELL ACTIVATION**	387	-0.21	-1.14	0.0988	0.6297
**NEGATIVE REGULATION OF FATTY ACID METABOLIC PROCESS**	17	-0.38	-1.13	0.2804	0.6304
**HETEROPHILIC CELL CELL ADHESION VIA PLASMA MEMBRANE CELL ADHESION MOLECULES**	41	-0.31	-1.13	0.2574	0.6310
**NEGATIVE REGULATION OF DNA BIOSYNTHETIC PROCESS**	25	0.34	1.03	0.4194	0.6353
**GLUTAMINE FAMILY AMINO ACID CATABOLIC PROCESS**	23	0.34	1.03	0.4335	0.6354
**POSITIVE REGULATION OF CELL CYCLE PROCESS**	211	0.23	1.03	0.3799	0.6356
**ATPASE REGULATOR ACTIVITY**	27	-0.32	-1.13	0.2824	0.6357
**REGULATION OF HOMEOSTATIC PROCESS**	397	0.22	1.03	0.3770	0.6359
**CARDIAC SEPTUM DEVELOPMENT**	82	0.26	1.03	0.4003	0.6363
**PEPTIDYL SERINE MODIFICATION**	137	0.25	1.03	0.4027	0.6364
**NEGATIVE REGULATION OF INTRACELLULAR PROTEIN TRANSPORT**	83	0.27	1.03	0.3962	0.6365
**CELL SURFACE RECEPTOR SIGNALING PATHWAY INVOLVED IN CELL CELL SIGNALING**	68	0.28	1.03	0.4168	0.6366
**POSITIVE REGULATION OF STEROL TRANSPORT**	16	0.38	1.03	0.4319	0.6367
**REGULATION OF MRNA CATABOLIC PROCESS**	26	0.33	1.03	0.4213	0.6369
**MITOTIC SPINDLE ORGANIZATION**	56	0.28	1.03	0.4081	0.6371
**CELLULAR RESPONSE TO PROSTAGLANDIN STIMULUS**	22	0.34	1.03	0.4081	0.6372
**REGULATION OF ICOSANOID SECRETION**	17	0.37	1.03	0.4155	0.6385

**PHOSPHATIDYLGLYCEROL ACYL CHAIN REMODELING**	16	0.38	1.03	0.4412	0.6388
**MOTOR NEURON AXON GUIDANCE**	26	-0.34	-1.12	0.2802	0.6394
**MICROTUBULE ORGANIZING CENTER PART**	116	0.25	1.03	0.3907	0.6419
**SPLEEN DEVELOPMENT**	34	0.32	1.03	0.4185	0.6426
**LUNG ALVEOLUS DEVELOPMENT**	40	0.30	1.03	0.4119	0.6431
**CHROMOSOME ORGANIZATION INVOLVED IN MEIOTIC CELL CYCLE**	41	-0.30	-1.12	0.2918	0.6434
**PEPTIDYL LYSINE METHYLATION**	62	0.28	1.03	0.4111	0.6437
**SECRETORY GRANULE ORGANIZATION**	25	0.34	1.03	0.4159	0.6449
**POSITIVE REGULATION OF MYELOID LEUKOCYTE DIFFERENTIATION**	45	-0.29	-1.12	0.2601	0.6462
**REGULATION OF PROTEIN KINASE A SIGNALING**	17	-0.37	-1.12	0.3191	0.6471
**CELLULAR RESPONSE TO ALKALOID**	31	-0.31	-1.12	0.2776	0.6474
**REGULATION OF LIPID BIOSYNTHETIC PROCESS**	117	-0.25	-1.12	0.2158	0.6478
**EPITHELIAL CELL DIFFERENTIATION**	441	0.21	1.02	0.4057	0.6481
**REGULATION OF CELL ADHESION MEDIATED BY INTEGRIN**	35	-0.31	-1.12	0.2960	0.6481
**POSITIVE REGULATION OF PATHWAY RESTRICTED SMAD PROTEIN PHOSPHORYLATION**	47	0.29	1.02	0.4121	0.6484
**ISOPRENOID METABOLIC PROCESS**	106	-0.25	-1.12	0.2055	0.6485
**NEUTRAL AMINO ACID TRANSMEMBRANE TRANSPORTER ACTIVITY**	28	0.33	1.02	0.4164	0.6486
**CELLULAR DEFENSE RESPONSE**	43	-0.30	-1.12	0.2857	0.6486
**CARTILAGE DEVELOPMENT INVOLVED IN ENDOCHONDRAL BONE MORPHOGENESIS**	18	-0.37	-1.12	0.2766	0.6487
**REGULATION OF FIBROBLAST MIGRATION**	24	0.34	1.02	0.4281	0.6488
**LUNG CELL DIFFERENTIATION**	24	0.33	1.02	0.4350	0.6488
**CENTRAL NERVOUS SYSTEM NEURON AXONOGENESIS**	25	-0.33	-1.12	0.2760	0.6488
**CELLULAR GLUCOSE HOMEOSTASIS**	71	-0.27	-1.12	0.2278	0.6489
**NEGATIVE REGULATION OF RECEPTOR MEDIATED ENDOCYTOSIS**	16	0.38	1.02	0.4288	0.6490
**POSITIVE REGULATION OF EPITHELIAL CELL DIFFERENTIATION**	50	-0.28	-1.12	0.2500	0.6490
**ENDOPLASMIC RETICULUM SUBCOMPARTMENT**	15	0.38	1.02	0.4262	0.6491

**EXTRINSIC APOPTOTIC SIGNALING PATHWAY IN ABSENCE OF LIGAND**	26	-0.34	-1.12	0.2895	0.6491
**TRANSCRIPTION COFACTOR BINDING**	21	0.35	1.02	0.4375	0.6493
**SYNAPTIC VESICLE CYCLE**	83	-0.26	-1.12	0.2423	0.6493
**RESPONSE TO ATP**	28	-0.34	-1.12	0.2879	0.6497
**SIGNAL TRANSDUCTION IN ABSENCE OF LIGAND**	26	-0.34	-1.12	0.3096	0.6498
**ADAPTATION OF SIGNALING PATHWAY**	20	-0.37	-1.12	0.3318	0.6499
**HEART PROCESS**	82	0.26	1.02	0.4311	0.6506
**NEGATIVE REGULATION OF T CELL PROLIFERATION**	40	-0.30	-1.12	0.2656	0.6507
**REGULATION OF CGMP METABOLIC PROCESS**	27	-0.34	-1.12	0.2815	0.6508
**NONMOTILE PRIMARY CILIUM ASSEMBLY**	19	0.36	1.02	0.4433	0.6511
**ENDOPEPTIDASE ACTIVITY**	344	0.22	1.02	0.4082	0.6513
**PERIPHERAL NERVOUS SYSTEM AXON ENSHEATHMENT**	21	0.35	1.02	0.4561	0.6520
**PROTEIN PHOSPHATASE BINDING**	107	0.25	1.02	0.4061	0.6533
**GLUTAMATE METABOLIC PROCESS**	26	0.33	1.02	0.4364	0.6534
**POSITIVE REGULATION OF ATP METABOLIC PROCESS**	21	0.35	1.02	0.4325	0.6535
**SIGNAL TRANSDUCTION BY P53 CLASS MEDIATOR**	103	0.25	1.02	0.4149	0.6535
**ENDOCARDIAL CUSHION FORMATION**	15	0.38	1.02	0.4086	0.6536
**AMMONIUM ION BINDING**	63	0.28	1.02	0.4071	0.6537
**DEACETYLASE ACTIVITY**	51	0.28	1.02	0.4179	0.6537
**CEREBRAL CORTEX DEVELOPMENT**	99	0.25	1.02	0.4032	0.6537
**STRUCTURE SPECIFIC DNA BINDING**	102	0.25	1.02	0.4242	0.6538
**BASAL PLASMA MEMBRANE**	31	0.32	1.02	0.4512	0.6539
**REGULATION OF PEPTIDASE ACTIVITY**	322	0.22	1.02	0.4095	0.6539
**TISSUE REMODELING**	83	0.26	1.02	0.4364	0.6541
**MONOCARBOXYLIC ACID BINDING**	47	0.29	1.02	0.4080	0.6542
**TUMOR NECROSIS FACTOR MEDIATED SIGNALING PATHWAY**	105	0.25	1.02	0.4184	0.6547
**POSITIVE REGULATION OF CELL DEVELOPMENT**	438	0.21	1.02	0.4325	0.6548
**MODULATION OF GROWTH OF SYMBIONT INVOLVED IN INTERACTION WITH HOST**	16	0.37	1.02	0.4363	0.6550
**EPIDERMAL CELL DIFFERENTIATION**	120	-0.24	-1.11	0.2331	0.6554
**POSITIVE REGULATION OF SMOOTH MUSCLE CELL MIGRATION**	28	0.33	1.02	0.4527	0.6555

**POSITIVE REGULATION OF PROTEIN TARGETING TO MEMBRANE**	18	0.36	1.02	0.4530	0.6556
**REGULATION OF EPITHELIAL CELL APOPTOTIC PROCESS**	55	0.28	1.02	0.4237	0.6557
**POSITIVE REGULATION OF NEURON APOPTOTIC PROCESS**	40	0.30	1.02	0.4213	0.6578
**CENTRIOLE**	81	0.26	1.02	0.4275	0.6580
**RAC PROTEIN SIGNAL TRANSDUCTION**	16	0.38	1.02	0.4441	0.6583
**POSITIVE REGULATION OF IMMUNE EFFECTOR PROCESS**	131	-0.24	-1.11	0.2145	0.6595
**POSITIVE REGULATION OF CYTOSKELETON ORGANIZATION**	158	0.24	1.01	0.4333	0.6610
**REGULATION OF CELLULAR COMPONENT SIZE**	298	0.22	1.01	0.4220	0.6611
**POSITIVE REGULATION OF KINASE ACTIVITY**	423	0.21	1.01	0.4456	0.6614
**REGULATION OF T CELL MEDIATED CYTOTOXICITY**	18	0.36	1.01	0.4207	0.6614
**SYNAPTIC TRANSMISSION DOPAMINERGIC**	16	0.36	1.01	0.4444	0.6615
**REGULATION OF PLATELET ACTIVATION**	29	0.32	1.01	0.4315	0.6616
**REGULATION OF FAT CELL DIFFERENTIATION**	95	0.25	1.01	0.4290	0.6617
**TRANSCRIPTIONAL REPRESSOR ACTIVITY RNA POLYMERASE II CORE PROMOTER PROXIMAL REGION SEQUENCE SPECIFIC BINDING**	90	0.26	1.01	0.4399	0.6617
**MONOSACCHARIDE CATABOLIC PROCESS**	50	0.29	1.01	0.4295	0.6617
**POSITIVE REGULATION OF NITRIC OXIDE SYNTHASE ACTIVITY**	18	0.36	1.01	0.4426	0.6618
**PHAGOCYTIC VESICLE MEMBRANE**	45	0.29	1.01	0.4362	0.6618
**NEGATIVE REGULATION OF MYOBLAST DIFFERENTIATION**	23	0.33	1.01	0.4283	0.6621
**G PROTEIN COUPLED RECEPTOR SIGNALING PATHWAY COUPLED TO CYCLIC NUCLEOTIDE SECOND MESSENGER**	156	-0.23	-1.11	0.2128	0.6632
**BINDING OF SPERM TO ZONA PELLUCIDA**	29	0.32	1.01	0.4216	0.6636
**ORGANOPHOSPHATE ESTER TRANSMEMBRANE TRANSPORTER ACTIVITY**	21	0.34	1.01	0.4462	0.6642
**CADHERIN BINDING**	28	0.32	1.01	0.4574	0.6654

**POSITIVE REGULATION OF CHROMOSOME SEGREGATION**	21	0.35	1.01	0.4480	0.6654
**TRANSCRIPTION FACTOR ACTIVITY RNA POLYMERASE II CORE PROMOTER PROXIMAL REGION SEQUENCE SPECIFIC BINDING**	293	-0.21	-1.11	0.1667	0.6655
**KINASE REGULATOR ACTIVITY**	174	0.23	1.01	0.4198	0.6657
**GROWTH FACTOR BINDING**	116	0.25	1.01	0.4298	0.6659
**DENDRITIC CELL MIGRATION**	17	-0.38	-1.11	0.3318	0.6662
**LAMININ BINDING**	27	-0.33	-1.11	0.3161	0.6667
**DNA ALKYLATION**	44	-0.29	-1.11	0.2961	0.6669
**DNA METHYLATION**	44	-0.29	-1.11	0.2816	0.6670
**REGULATION OF AXONOGENESIS**	159	-0.23	-1.11	0.2081	0.6672
**REGULATION OF NUCLEOTIDE CATABOLIC PROCESS**	33	0.31	1.01	0.4520	0.6672
**REGULATION OF CYCLIC NUCLEOTIDE METABOLIC PROCESS**	143	-0.23	-1.11	0.2000	0.6678
**REGULATION OF STEROID METABOLIC PROCESS**	64	0.27	1.01	0.4621	0.6680
**REGULATION OF CYTOKINE PRODUCTION INVOLVED IN INFLAMMATORY RESPONSE**	17	-0.38	-1.10	0.3296	0.6691
**POSITIVE REGULATION OF STEM CELL PROLIFERATION**	53	-0.27	-1.10	0.2965	0.6693
**NEGATIVE REGULATION OF LEUKOCYTE PROLIFERATION**	56	-0.28	-1.10	0.2787	0.6696
**ARF GUANYL NUCLEOTIDE EXCHANGE FACTOR ACTIVITY**	23	0.33	1.01	0.4470	0.6699
**SPERM EGG RECOGNITION**	39	0.30	1.01	0.4511	0.6701
**PROTEIN LOCALIZATION TO CELL PERIPHERY**	136	0.24	1.01	0.4476	0.6706
**CARBOHYDRATE HOMEOSTASIS**	154	-0.23	-1.10	0.2211	0.6707
**PERICARDIUM DEVELOPMENT**	17	-0.38	-1.10	0.3079	0.6708
**NEGATIVE REGULATION OF IMMUNE SYSTEM PROCESS**	310	-0.21	-1.10	0.1724	0.6709
**LIMBIC SYSTEM DEVELOPMENT**	97	-0.25	-1.10	0.2605	0.6713
**COCHLEA MORPHOGENESIS**	20	-0.36	-1.10	0.3558	0.6715
**SYNAPSE ORGANIZATION**	136	-0.23	-1.10	0.2140	0.6716
**PODOSOME**	23	-0.35	-1.10	0.2974	0.6716
**TRANSCRIPTION FACTOR ACTIVITY RNA POLYMERASE II TRANSCRIPTION FACTOR BINDING**	122	0.24	1.01	0.4517	0.6729

**REGULATION OF GLUTAMATE SECRETION**	15	0.36	1.01	0.4365	0.6730
**HISTONE H3 DEACETYLATION**	20	0.35	1.01	0.4316	0.6732
**REGULATION OF INSULIN RECEPTOR SIGNALING PATHWAY**	40	-0.30	-1.10	0.2846	0.6737
**HISTONE EXCHANGE**	32	-0.31	-1.10	0.3144	0.6738
**REGULATION OF PEPTIDE SECRETION**	189	-0.22	-1.10	0.2190	0.6738
**RHO GUANYL NUCLEOTIDE EXCHANGE FACTOR ACTIVITY**	62	-0.27	-1.10	0.2590	0.6745
**ENTRY INTO CELL OF OTHER ORGANISM INVOLVED IN SYMBIOTIC INTERACTION**	76	-0.25	-1.10	0.2676	0.6753
**REPLICATION FORK**	53	0.28	1.01	0.4609	0.6754
**REGULATION OF MUSCLE CELL APOPTOTIC PROCESS**	40	-0.30	-1.10	0.2952	0.6755
**AXIS SPECIFICATION**	86	-0.26	-1.10	0.2209	0.6759
**MEMBRANE DEPOLARIZATION**	57	-0.27	-1.10	0.3055	0.6760
**REGULATION OF STRIATED MUSCLE CELL APOPTOTIC PROCESS**	20	-0.35	-1.10	0.3287	0.6761
**SARCOMERE ORGANIZATION**	25	-0.33	-1.09	0.3540	0.6761
**ENTRY INTO OTHER ORGANISM INVOLVED IN SYMBIOTIC INTERACTION**	76	-0.25	-1.10	0.2922	0.6762
**PYRIDINE CONTAINING COMPOUND BIOSYNTHETIC PROCESS**	19	-0.35	-1.10	0.3473	0.6763
**LAMELLAR BODY**	17	-0.37	-1.10	0.3254	0.6766
**TRANSCRIPTION FACTOR COMPLEX**	265	0.22	1.00	0.4261	0.6768
**BRUSH BORDER**	93	0.25	1.00	0.4359	0.6768
**REGULATION OF ACUTE INFLAMMATORY RESPONSE**	65	-0.27	-1.10	0.2373	0.6772
**POSITIVE REGULATION OF ADAPTIVE IMMUNE RESPONSE**	64	-0.27	-1.10	0.2800	0.6779
**NEGATIVE REGULATION OF DEFENSE RESPONSE TO VIRUS**	16	0.37	1.00	0.4413	0.6780
**NEGATIVE REGULATION OF NOTCH SIGNALING PATHWAY**	24	-0.34	-1.09	0.3070	0.6781
**SOMATIC DIVERSIFICATION OF IMMUNE RECEPTORS VIA GERMLINE RECOMBINATION WITHIN A SINGLE LOCUS**	27	0.32	1.00	0.4476	0.6783
**NEGATIVE REGULATION OF HOMEOSTATIC PROCESS**	107	0.25	1.00	0.4466	0.6784
**POSITIVE REGULATION OF PEPTIDYL TYROSINE PHOSPHORYLATION**	147	0.24	1.00	0.4498	0.6785

**REGULATION OF PHOSPHOLIPASE C ACTIVITY**	37	-0.30	-1.10	0.3112	0.6787
**PROTEIN HETEROTETRAMERIZATION**	20	0.35	1.00	0.4598	0.6788
**REGULATION OF HISTONE H3 K9 METHYLATION**	15	-0.38	-1.09	0.3386	0.6795
**MOVEMENT IN ENVIRONMENT OF OTHER ORGANISM INVOLVED IN SYMBIOTIC INTERACTION**	76	-0.25	-1.09	0.2672	0.6798
**EPITHELIAL STRUCTURE MAINTENANCE**	19	-0.36	-1.09	0.3174	0.6801
**RESPONSE TO EXOGENOUS DSRNA**	34	0.30	1.00	0.4570	0.6806
**REGULATION OF DOPAMINE SECRETION**	22	-0.35	-1.09	0.3244	0.6806
**REGULATION OF VESICLE MEDIATED TRANSPORT**	414	0.21	1.00	0.4681	0.6808
**MITOGEN ACTIVATED PROTEIN KINASE BINDING**	24	0.33	1.00	0.4740	0.6808
**REGULATION OF MICROTUBULE POLYMERIZATION**	31	0.31	1.00	0.4499	0.6809
**DNA SECONDARY STRUCTURE BINDING**	20	-0.35	-1.09	0.3611	0.6812
**NEGATIVE REGULATION OF CELL CYCLE G2 M PHASE TRANSITION**	21	0.35	1.00	0.4489	0.6813
**RESPONSE TO ACID CHEMICAL**	281	0.22	1.00	0.4631	0.6814
**REGULATION OF STEM CELL PROLIFERATION**	78	-0.25	-1.09	0.2832	0.6819
**ENTRY INTO HOST**	76	-0.25	-1.09	0.2653	0.6821
**REGULATION OF T CELL RECEPTOR SIGNALING PATHWAY**	26	0.33	1.00	0.4509	0.6822
**REGULATION OF SYNAPTIC VESICLE EXOCYTOSIS**	19	0.34	1.00	0.4522	0.6822
**NEURON MIGRATION**	102	-0.24	-1.09	0.2799	0.6824
**BICARBONATE TRANSMEMBRANE TRANSPORTER ACTIVITY**	17	0.36	1.00	0.4536	0.6824
**GLUCOSE HOMEOSTASIS**	154	-0.23	-1.09	0.2148	0.6828
**DENDRITE MEMBRANE**	19	-0.35	-1.09	0.3539	0.6829
**VESICLE CYTOSKELETAL TRAFFICKING**	35	0.30	1.00	0.4868	0.6829
**NEGATIVE REGULATION OF ORGANIC ACID TRANSPORT**	17	0.37	1.00	0.4541	0.6829
**MUSCLE ORGAN MORPHOGENESIS**	67	-0.27	-1.09	0.3219	0.6832
**NEGATIVE REGULATION OF EPITHELIAL CELL MIGRATION**	50	-0.28	-1.09	0.3039	0.6832
**E BOX BINDING**	30	-0.32	-1.09	0.3118	0.6838
**FEMALE GAMETE GENERATION**	80	-0.26	-1.09	0.3015	0.6845
**RESPONSE TO TUMOR NECROSIS FACTOR**	197	0.23	1.00	0.4763	0.6847

**STEROL BIOSYNTHETIC PROCESS**	37	0.30	1.00	0.4631	0.6847
**INSULIN RECEPTOR BINDING**	29	0.32	1.00	0.4692	0.6848
**SMALL GTPASE MEDIATED SIGNAL TRANSDUCTION**	308	0.22	1.00	0.4490	0.6849
**MESONEPHRIC TUBULE MORPHOGENESIS**	49	0.28	1.00	0.4590	0.6853
**OSTEOCLAST DIFFERENTIATION**	28	0.32	1.00	0.4655	0.6877
**PHOSPHATIDYLCHOLINE BINDING**	18	0.35	1.00	0.4500	0.6878
**ROUGH ENDOPLASMIC RETICULUM MEMBRANE**	20	0.34	1.00	0.4551	0.6880
**POSITIVE REGULATION OF NUCLEOSIDE METABOLIC PROCESS**	21	0.35	1.00	0.4660	0.6881
**SOMATIC CELL DNA RECOMBINATION**	27	0.32	1.00	0.4699	0.6882
**MITOGEN ACTIVATED PROTEIN KINASE KINASE BINDING**	15	0.36	1.00	0.4576	0.6883
**PIGMENT CELL DIFFERENTIATION**	26	0.32	1.00	0.4564	0.6884
**NEGATIVE REGULATION OF DEVELOPMENTAL GROWTH**	78	-0.25	-1.09	0.2790	0.6899
**NEGATIVE REGULATION OF STEROID METABOLIC PROCESS**	20	-0.34	-1.09	0.3237	0.6901
**SODIUM CHANNEL ACTIVITY**	35	-0.30	-1.08	0.3264	0.6911
**CELLULAR RESPONSE TO FATTY ACID**	46	0.29	1.00	0.4737	0.6912
**ANTIGEN BINDING**	38	-0.29	-1.08	0.3166	0.6919
**REGULATION OF TRANSMEMBRANE RECEPTOR PROTEIN SERINE THREONINE KINASE SIGNALING PATHWAY**	182	0.23	0.99	0.4641	0.6921
**POTASSIUM ION HOMEOSTASIS**	17	0.36	0.99	0.4461	0.6922
**OXIDOREDUCTASE ACTIVITY ACTING ON THE CH NH GROUP OF DONORS NAD OR NADP AS ACCEPTOR**	15	0.37	0.99	0.4841	0.6924
**POSITIVE REGULATION OF STAT CASCADE**	64	-0.27	-1.08	0.3031	0.6930
**DOPAMINE METABOLIC PROCESS**	22	0.34	0.99	0.4476	0.6931
**RESPONSE TO ORGANOPHOSPHORUS**	126	-0.23	-1.08	0.2862	0.6932
**ESTABLISHMENT OF ENDOTHELIAL BARRIER**	28	0.32	0.99	0.4783	0.6933
**ENDOCARDIAL CUSHION DEVELOPMENT**	31	0.31	0.99	0.4697	0.6933
**PROTEIN SELF ASSOCIATION**	36	0.30	0.99	0.4679	0.6933
**CAMERA TYPE EYE MORPHOGENESIS**	98	0.25	0.99	0.4820	0.6934
**REGULATION OF PROTEIN INSERTION INTO MITOCHONDRIAL MEMBRANE**	23	0.32	0.99	0.4741	0.6935

INVOLVED IN APOPTOTIC SIGNALING PATHWAY					
EXTRINSIC COMPONENT OF ORGANELLE MEMBRANE	20	0.34	0.99	0.4764	0.6936
CELLULAR RESPONSE TO INTERLEUKIN 1	71	0.26	0.99	0.4639	0.6936
POSITIVE REGULATION OF PHOSPHATASE ACTIVITY	27	0.32	0.99	0.4917	0.6936
POSITIVE REGULATION OF HEMOPOIESIS	140	-0.23	-1.08	0.2625	0.6939
CELLULAR RESPONSE TO ESTRADIOL STIMULUS	28	0.31	0.99	0.4795	0.6948
MYOBLAST DIFFERENTIATION	35	-0.30	-1.08	0.3122	0.6951
EPIDERMIS DEVELOPMENT	218	-0.22	-1.08	0.2375	0.6954
CEREBRAL CORTEX RADIALLY ORIENTED CELL MIGRATION	26	-0.32	-1.08	0.3546	0.6954
POSITIVE REGULATION OF CELL MATRIX ADHESION	38	-0.29	-1.08	0.3152	0.6956
I BAND	110	-0.24	-1.08	0.2730	0.6958
VIRAL ENTRY INTO HOST CELL	76	-0.25	-1.08	0.3068	0.6962
T CELL MEDIATED IMMUNITY	20	-0.34	-1.08	0.3425	0.6965
MOVEMENT IN HOST ENVIRONMENT	76	-0.25	-1.08	0.3251	0.6965
CYTOLYSIS	15	-0.38	-1.08	0.3592	0.6965
REGULATION OF HORMONE SECRETION	239	-0.21	-1.08	0.2364	0.6966
PRIMARY ALCOHOL METABOLIC PROCESS	40	-0.29	-1.08	0.3621	0.6967
POSITIVE REGULATION OF PEPTIDYL SERINE PHOSPHORYLATION	77	-0.26	-1.07	0.3138	0.6968
MUSCLE ORGAN DEVELOPMENT	255	-0.21	-1.08	0.2545	0.6969
EMBRYONIC ORGAN DEVELOPMENT	378	-0.20	-1.08	0.1716	0.6969
REGULATION OF CELL AGING	30	-0.31	-1.07	0.3563	0.6970
MYELOID CELL ACTIVATION INVOLVED IN IMMUNE RESPONSE	37	-0.30	-1.08	0.3222	0.6971
TRANSCRIPTIONAL ACTIVATOR ACTIVITY RNA POLYMERASE II DISTAL ENHANCER SEQUENCE SPECIFIC BINDING	23	-0.34	-1.08	0.3510	0.6973
CALCIUM DEPENDENT PHOSPHOLIPID BINDING	48	-0.28	-1.08	0.3217	0.6973
ENTRY INTO HOST CELL	76	-0.25	-1.08	0.3077	0.6974
REGULATION OF TELOMERASE ACTIVITY	36	0.30	0.99	0.4508	0.6974
CEREBRAL CORTEX RADIAL GLIA GUIDED MIGRATION	17	-0.36	-1.08	0.3357	0.6975

**CARTILAGE DEVELOPMENT**	139	-0.22	-1.07	0.2420	0.6976
**NEGATIVE REGULATION OF CYTOKINE PRODUCTION**	181	-0.22	-1.07	0.2621	0.6977
**AMEBOIDAL TYPE CELL MIGRATION**	143	-0.23	-1.07	0.2809	0.6977
**CALCIUM ION IMPORT**	53	-0.27	-1.07	0.3194	0.6978
**POSITIVE REGULATION OF SKELETAL MUSCLE TISSUE DEVELOPMENT**	25	-0.33	-1.07	0.3540	0.6980
**CELLULAR RESPONSE TO CARBOHYDRATE STIMULUS**	69	-0.26	-1.08	0.2845	0.6981
**REGULATION OF INTERLEUKIN 6 BIOSYNTHETIC PROCESS**	15	-0.38	-1.07	0.3684	0.6983
**SOMATIC DIVERSIFICATION OF IMMUNE RECEPTORS**	33	0.30	0.99	0.4668	0.6983
**ENDOCYTIC RECYCLING**	23	0.33	0.99	0.4735	0.6984
**NEPHRIC DUCT DEVELOPMENT**	15	-0.38	-1.07	0.3601	0.6985
**NEUTRAL AMINO ACID TRANSPORT**	30	0.31	0.99	0.4680	0.6991
**EMBRYONIC MORPHOGENESIS**	496	-0.20	-1.07	0.2044	0.6992
**NITRIC OXIDE SYNTHASE BINDING**	18	0.34	0.99	0.4804	0.6996
**NEGATIVE REGULATION OF HYDROLASE ACTIVITY**	327	0.21	0.99	0.5048	0.7003
**REGULATION OF TUMOR NECROSIS FACTOR MEDIATED SIGNALING PATHWAY**	40	0.29	0.99	0.4881	0.7004
**RENAL SYSTEM PROCESS INVOLVED IN REGULATION OF SYSTEMIC ARTERIAL BLOOD PRESSURE**	18	0.36	0.99	0.4727	0.7010
**MACROPHAGE DIFFERENTIATION**	17	-0.35	-1.07	0.3457	0.7014
**POSITIVE REGULATION OF PROTEIN LOCALIZATION TO CELL PERIPHERY**	33	0.30	0.99	0.4678	0.7021
**CORE PROMOTER BINDING**	133	0.23	0.99	0.4854	0.7023
**BLOOD COAGULATION INTRINSIC PATHWAY**	16	0.36	0.99	0.4648	0.7033
**PEPTIDASE ACTIVATOR ACTIVITY**	34	0.30	0.99	0.4677	0.7052
**SPERMATID DIFFERENTIATION**	105	0.24	0.99	0.4846	0.7053
**POSITIVE REGULATION OF VIRAL GENOME REPLICATION**	27	0.32	0.99	0.4736	0.7054
**REGULATION OF NEURON PROJECTION DEVELOPMENT**	384	0.21	0.99	0.5023	0.7056
**MULTICELLULAR ORGANISMAL MACROMOLECULE METABOLIC PROCESS**	71	-0.25	-1.07	0.3183	0.7058
**RELEASE OF CYTOCHROME C FROM MITOCHONDRIA**	17	0.35	0.99	0.4608	0.7059

**G PROTEIN BETA GAMMA SUBUNIT COMPLEX BINDING**	21	0.34	0.98	0.4860	0.7060
**REGULATION OF MYOBLAST DIFFERENTIATION**	45	0.29	0.99	0.4927	0.7060
**PROTEIN INSERTION INTO MEMBRANE**	17	0.36	0.98	0.4786	0.7070
**EPITHELIAL CELL APOPTOTIC PROCESS**	24	0.32	0.98	0.4553	0.7071
**RESPONSE TO LEAD ION**	20	0.34	0.98	0.4876	0.7072
**CARBON CARBON LYASE ACTIVITY**	49	0.28	0.98	0.4944	0.7073
**REGULATION OF CARDIAC MUSCLE CONTRACTION BY REGULATION OF THE RELEASE OF SEQUESTERED CALCIUM ION**	17	0.36	0.98	0.4836	0.7073
**AXONEME ASSEMBLY**	26	-0.32	-1.07	0.3367	0.7075
**POSITIVE REGULATION OF PROTEIN LOCALIZATION TO PLASMA MEMBRANE**	33	0.30	0.98	0.4850	0.7081
**CELLULAR RESPONSE TO TOXIC SUBSTANCE**	24	-0.33	-1.07	0.3515	0.7086
**REPRODUCTIVE BEHAVIOR**	26	-0.32	-1.07	0.3454	0.7087
**TUMOR NECROSIS FACTOR RECEPTOR SUPERFAMILY BINDING**	42	-0.28	-1.07	0.3496	0.7089
**PROTEIN KINASE A BINDING**	36	0.30	0.98	0.4968	0.7095
**REGULATION OF IMMUNOGLOBULIN SECRETION**	15	0.36	0.98	0.4621	0.7098
**POSITIVE REGULATION OF MYELOID LEUKOCYTE MEDIATED IMMUNITY**	15	0.37	0.98	0.4922	0.7100
**ATP GENERATION FROM ADP**	36	0.29	0.98	0.4891	0.7103
**COMPLEMENT BINDING**	15	0.38	0.98	0.4753	0.7113
**RESPONSE TO FLUID SHEAR STRESS**	32	0.30	0.98	0.4958	0.7118
**PROTEIN KINASE C ACTIVATING G PROTEIN COUPLED RECEPTOR SIGNALING PATHWAY**	31	0.30	0.98	0.4912	0.7120
**CATION CHANNEL COMPLEX**	157	-0.22	-1.06	0.2852	0.7141
**SEMAPHORIN PLEXIN SIGNALING PATHWAY**	35	-0.30	-1.06	0.3604	0.7146
**POSITIVE REGULATION OF VASOCONSTRICTION**	34	-0.30	-1.06	0.3598	0.7150
**POSITIVE REGULATION OF NERVOUS SYSTEM DEVELOPMENT**	407	0.21	0.98	0.5483	0.7154
**POSITIVE REGULATION OF SYNAPTIC TRANSMISSION**	104	0.24	0.98	0.4772	0.7155
**POSITIVE REGULATION OF STEROID METABOLIC PROCESS**	21	0.33	0.98	0.4992	0.7155
**ODONTOGENESIS OF DENTIN CONTAINING TOOTH**	73	0.26	0.98	0.4931	0.7162

**SARCOLEMMA**	113	0.24	0.98	0.4978	0.7163
**ACTIN NUCLEATION**	19	0.35	0.98	0.4913	0.7169
**NEGATIVE REGULATION OF CATECHOLAMINE SECRETION**	16	-0.37	-1.06	0.3846	0.7170
**VASCULAR ENDOTHELIAL GROWTH FACTOR RECEPTOR SIGNALING PATHWAY**	73	-0.25	-1.06	0.3219	0.7179
**REGULATION OF CHROMATIN ORGANIZATION**	133	-0.23	-1.06	0.3060	0.7180
**RESPONSE TO PH**	36	-0.30	-1.06	0.3564	0.7184
**OOGENESIS**	53	-0.27	-1.06	0.3360	0.7188
**NEGATIVE REGULATION OF CELL SUBSTRATE ADHESION**	46	0.28	0.98	0.5093	0.7189
**POSITIVE REGULATION OF DNA RECOMBINATION**	16	-0.37	-1.06	0.3643	0.7190
**POSITIVE REGULATION OF PROTEIN INSERTION INTO MITOCHONDRIAL MEMBRANE INVOLVED IN APOPTOTIC SIGNALING PATHWAY**	23	0.32	0.98	0.4733	0.7191
**DNA METHYLATION OR DEMETHYLATION**	52	-0.27	-1.06	0.3636	0.7194
**DECIDUALIZATION**	18	-0.36	-1.06	0.3759	0.7194
**REGULATION OF WATER LOSS VIA SKIN**	15	-0.37	-1.06	0.3806	0.7196
**NEGATIVE REGULATION OF CELL DEVELOPMENT**	276	-0.20	-1.06	0.2896	0.7197
**NEGATIVE REGULATION OF OSSIFICATION**	64	0.27	0.98	0.4946	0.7204
**FIBROBLAST GROWTH FACTOR BINDING**	23	0.33	0.98	0.4967	0.7215
**REGULATION OF SUBSTRATE ADHESION DEPENDENT CELL SPREADING**	37	0.29	0.98	0.4691	0.7218
**NEGATIVE REGULATION OF DEFENSE RESPONSE**	122	0.24	0.98	0.4972	0.7220
**CARDIAC MUSCLE CELL DIFFERENTIATION**	72	0.25	0.98	0.5008	0.7220
**PROTEIN SERINE THREONINE KINASE INHIBITOR ACTIVITY**	29	0.30	0.97	0.4846	0.7221
**ACID AMINO ACID LIGASE ACTIVITY**	19	0.34	0.97	0.4921	0.7234
**MUSCLE TISSUE DEVELOPMENT**	254	-0.21	-1.06	0.2805	0.7236
**DNA REPLICATION INDEPENDENT NUCLEOSOME ASSEMBLY**	35	0.29	0.97	0.4763	0.7237
**BETA CATENIN DESTRUCTION COMPLEX DISASSEMBLY**	21	0.33	0.97	0.5069	0.7239
**PROTEIN PALMITOYLATION**	22	0.33	0.97	0.4809	0.7239

**NEGATIVE REGULATION OF ENDOTHELIAL CELL APOPTOTIC PROCESS**	26	0.32	0.97	0.4947	0.7241
**REGULATION OF EXCRETION**	25	-0.31	-1.06	0.3599	0.7243
**REGULATION OF HUMORAL IMMUNE RESPONSE**	42	-0.28	-1.06	0.3720	0.7254
**REGULATION OF CILIUM ASSEMBLY**	47	0.27	0.97	0.5066	0.7260
**HIPPO SIGNALING**	24	0.32	0.97	0.4833	0.7267
**TRANSMITTER GATED CHANNEL ACTIVITY**	25	-0.32	-1.06	0.3668	0.7282
**CELL MORPHOGENESIS INVOLVED IN DIFFERENTIATION**	465	-0.19	-1.06	0.2532	0.7284
**SIGNAL PEPTIDE PROCESSING**	20	0.34	0.97	0.5062	0.7285
**REGULATION OF SMOOTH MUSCLE CELL PROLIFERATION**	91	0.25	0.97	0.5184	0.7285
**ADP METABOLIC PROCESS**	42	0.28	0.97	0.5153	0.7293
**BILE ACID METABOLIC PROCESS**	27	0.31	0.97	0.5025	0.7293
**CELL SUBSTRATE JUNCTION ASSEMBLY**	38	0.29	0.97	0.5126	0.7293
**STEROID BIOSYNTHETIC PROCESS**	96	0.24	0.97	0.5122	0.7294
**GASTRULATION WITH MOUTH FORMING SECOND**	27	0.31	0.97	0.5083	0.7303
**BILE ACID BIOSYNTHETIC PROCESS**	18	0.35	0.97	0.5009	0.7304
**RESPONSE TO DRUG**	390	0.21	0.97	0.5421	0.7305
**CELL JUNCTION ORGANIZATION**	167	0.22	0.97	0.5124	0.7305
**REGULATION OF ACTIN FILAMENT BUNDLE ASSEMBLY**	71	0.26	0.97	0.4945	0.7306
**REGULATION OF MUSCLE SYSTEM PROCESS**	186	0.22	0.97	0.5463	0.7315
**REGULATION OF PHOSPHOPROTEIN PHOSPHATASE ACTIVITY**	53	0.27	0.97	0.5064	0.7316
**REGULATION OF MUSCLE TISSUE DEVELOPMENT**	93	0.24	0.97	0.5313	0.7317
**RESPONSE TO INTERLEUKIN 6**	23	0.32	0.97	0.5009	0.7319
**NEGATIVE REGULATION OF POTASSIUM ION TRANSPORT**	32	-0.30	-1.05	0.3790	0.7322
**RENAL TUBULE DEVELOPMENT**	73	-0.25	-1.05	0.3650	0.7325
**PHOTORECEPTOR DISC MEMBRANE**	15	-0.38	-1.05	0.3916	0.7326
**TELENCEPHALON GLIAL CELL MIGRATION**	17	-0.36	-1.05	0.3583	0.7328
**REGULATION OF VASOCONSTRICTION**	62	-0.26	-1.05	0.3704	0.7331
**REGULATION OF CARTILAGE DEVELOPMENT**	59	-0.27	-1.05	0.3716	0.7332
**COBALAMIN METABOLIC PROCESS**	17	0.35	0.97	0.5091	0.7334

**AXON EXTENSION**	35	-0.29	-1.05	0.3639	0.7338
**REGULATION OF EPIDERMAL GROWTH FACTOR ACTIVATED RECEPTOR ACTIVITY**	21	0.33	0.97	0.4966	0.7339
**HISTONE H3 K4 METHYLATION**	28	0.30	0.97	0.4914	0.7340
**NEGATIVE REGULATION OF AMINE TRANSPORT**	25	-0.32	-1.05	0.3611	0.7343
**REGULATION OF BMP SIGNALING PATHWAY**	67	0.26	0.97	0.5185	0.7343
**FEEDING BEHAVIOR**	85	-0.24	-1.05	0.3354	0.7344
**NEGATIVE REGULATION OF PEPTIDE SECRETION**	47	-0.27	-1.05	0.3653	0.7344
**SITE OF POLARIZED GROWTH**	138	-0.22	-1.05	0.3227	0.7345
**CELLULAR COMPONENT MAINTENANCE**	16	-0.37	-1.05	0.4119	0.7346
**REGULATION OF STRIATED MUSCLE CELL DIFFERENTIATION**	79	-0.25	-1.05	0.3544	0.7348
**PHOTORECEPTOR INNER SEGMENT**	33	-0.30	-1.05	0.3623	0.7352
**EMBRYONIC DIGESTIVE TRACT DEVELOPMENT**	30	-0.31	-1.05	0.3890	0.7352
**PHOSPHATIDYLINOSITOL 4 5 BISPHOSPHATE BINDING**	48	0.28	0.97	0.5135	0.7360
**SINGLE ORGANISM BEHAVIOR**	343	-0.20	-1.05	0.2727	0.7364
**CILIUM MORPHOGENESIS**	157	0.22	0.97	0.5463	0.7366
**G1 DNA DAMAGE CHECKPOINT**	61	0.26	0.97	0.4938	0.7367
**CELLULAR RESPONSE TO BMP STIMULUS**	87	0.24	0.96	0.5110	0.7383
**METALLOEXOPEPTIDASE ACTIVITY**	46	0.27	0.96	0.5255	0.7385
**ACTIVATION OF IMMUNE RESPONSE**	332	0.21	0.96	0.5603	0.7385
**DNA REPLICATION INDEPENDENT NUCLEOSOME ORGANIZATION**	35	0.29	0.96	0.5261	0.7385
**POSITIVE REGULATION OF ACTIN FILAMENT POLYMERIZATION**	58	0.27	0.96	0.5228	0.7386
**BLOOD COAGULATION FIBRIN CLOT FORMATION**	23	0.32	0.96	0.5053	0.7388
**RESPONSE TO INTERFERON BETA**	20	-0.33	-1.05	0.3886	0.7388
**RESPONSE TO PROSTAGLANDIN E**	23	0.33	0.96	0.4958	0.7389
**RESPIRATORY GASEOUS EXCHANGE**	45	0.28	0.96	0.5233	0.7389
**POSITIVE REGULATION OF RNA SPLICING**	22	0.32	0.96	0.5075	0.7394
**NEPHRON EPITHELIUM DEVELOPMENT**	87	-0.24	-1.05	0.3449	0.7402
**POSITIVE REGULATION OF ERK1 AND ERK2 CASCADE**	148	0.22	0.96	0.5335	0.7405
**PRESYNAPSE**	265	0.21	0.96	0.5637	0.7405

**REGULATION OF DEVELOPMENTAL GROWTH**	263	0.21	0.96	0.5687	0.7412
**DEFENSE RESPONSE TO BACTERIUM**	134	-0.23	-1.05	0.3382	0.7414
**GLYCERALDEHYDE 3 PHOSPHATE METABOLIC PROCESS**	16	0.35	0.96	0.5094	0.7415
**GLAND DEVELOPMENT**	371	0.20	0.96	0.5780	0.7420
**NEURON SPINE**	110	0.24	0.96	0.5258	0.7421
**TELOMERE ORGANIZATION**	75	0.25	0.96	0.5339	0.7421
**REGULATION OF EXTRINSIC APOPTOTIC SIGNALING PATHWAY**	141	0.23	0.96	0.5629	0.7422
**TELOMERASE HOLOENZYME COMPLEX**	19	0.34	0.96	0.5018	0.7423
**REGULATION OF NF KAPPAB IMPORT INTO NUCLEUS**	41	0.28	0.96	0.5141	0.7428
**ACTIVATION OF PROTEIN KINASE ACTIVITY**	242	0.21	0.96	0.5661	0.7429
**HIPPOCAMPUS DEVELOPMENT**	70	-0.25	-1.04	0.3952	0.7433
**SECRETORY GRANULE MEMBRANE**	74	0.25	0.96	0.5457	0.7435
**NEURON PROJECTION EXTENSION**	49	-0.27	-1.04	0.4158	0.7436
**PROTEIN KINASE B SIGNALING**	34	-0.29	-1.04	0.3614	0.7438
**POSITIVE REGULATION OF TRANSMEMBRANE TRANSPORT**	121	-0.22	-1.04	0.3370	0.7441
**REGULATION OF EPITHELIAL CELL DIFFERENTIATION**	111	-0.23	-1.04	0.3442	0.7441
**EXTRINSIC COMPONENT OF CYTOPLASMIC SIDE OF PLASMA MEMBRANE**	89	-0.24	-1.04	0.3363	0.7442
**POSITIVE REGULATION OF STRIATED MUSCLE CELL DIFFERENTIATION**	47	-0.27	-1.04	0.3911	0.7446
**POSITIVE REGULATION OF CALCIUM ION DEPENDENT EXOCYTOSIS**	24	0.31	0.96	0.5061	0.7449
**POSITIVE REGULATION OF CIRCADIAN RHYTHM**	20	-0.34	-1.04	0.3785	0.7450
**RESPONSE TO STEROID HORMONE**	450	0.20	0.96	0.5751	0.7459
**SYNAPTIC VESICLE ENDOCYTOSIS**	17	0.35	0.96	0.5120	0.7461
**RAC GTPASE BINDING**	34	-0.29	-1.04	0.4090	0.7462
**CHONDROCYTE DEVELOPMENT**	20	0.33	0.96	0.5347	0.7467
**PROTEIN BINDING INVOLVED IN CELL ADHESION**	17	-0.36	-1.04	0.3995	0.7468
**MULTI ORGANISM BEHAVIOR**	68	-0.25	-1.04	0.3675	0.7476
**ENDOPLASMIC RETICULUM TO CYTOSOL TRANSPORT**	19	0.34	0.96	0.5105	0.7478
**LAMELLIPODIUM**	159	-0.22	-1.04	0.3346	0.7479
**TETRAPYRROLE BINDING**	85	-0.24	-1.04	0.3571	0.7480

**SECOND MESSENGER MEDIATED SIGNALING**	144	0.23	0.96	0.5684	0.7480
**GROWTH FACTOR RECEPTOR BINDING**	113	0.23	0.96	0.5477	0.7481
**BINDING BRIDGING**	157	0.22	0.96	0.5658	0.7481
**HYDROLASE ACTIVITY ACTING ON CARBON NITROGEN BUT NOT PEPTIDE BONDS IN CYCLIC AMIDINES**	26	0.31	0.96	0.5169	0.7483
**NEGATIVE REGULATION OF EPITHELIAL CELL DIFFERENTIATION**	34	0.30	0.96	0.5632	0.7483
**REGULATION OF RESPONSE TO INTERFERON GAMMA**	21	0.33	0.96	0.5299	0.7484
**L ALPHA AMINO ACID TRANSMEMBRANE TRANSPORT**	30	0.30	0.96	0.5395	0.7485
**HAIR CELL DIFFERENTIATION**	32	0.29	0.96	0.5287	0.7486
**REGULATION OF PRI MIRNA TRANSCRIPTION FROM RNA POLYMERASE II PROMOTER**	16	0.35	0.96	0.5063	0.7487
**CLUSTER OF ACTIN BASED CELL PROJECTIONS**	122	0.23	0.96	0.5396	0.7492
**EXCITATORY SYNAPSE**	179	-0.21	-1.04	0.3553	0.7496
**UNFOLDED PROTEIN BINDING**	73	-0.25	-1.04	0.3839	0.7497
**REGULATION OF T CELL APOPTOTIC PROCESS**	27	-0.31	-1.04	0.4020	0.7499
**REGULATION OF MEGAKARYOCYTE DIFFERENTIATION**	16	-0.36	-1.04	0.3941	0.7509
**INOSITOL PHOSPHATE METABOLIC PROCESS**	52	0.27	0.95	0.5462	0.7511
**ENDOSOME LUMEN**	20	-0.33	-1.04	0.3850	0.7517
**NEURON PROJECTION TERMINUS**	124	0.23	0.95	0.5623	0.7518
**REGULATION OF PROTEIN IMPORT INTO NUCLEUS TRANSLOCATION**	19	-0.34	-1.04	0.3865	0.7518
**POSITIVE REGULATION OF GLIOGENESIS**	43	0.27	0.95	0.5219	0.7520
**FILOPODIUM MEMBRANE**	18	-0.35	-1.04	0.4227	0.7524
**NEUTROPHIL MEDIATED IMMUNITY**	17	0.35	0.95	0.5036	0.7531
**REGULATION OF BONE REMODELING**	39	0.29	0.95	0.5250	0.7536
**LIPID PHOSPHORYLATION**	93	0.24	0.95	0.5676	0.7538
**NEGATIVE REGULATION OF NERVOUS SYSTEM DEVELOPMENT**	243	-0.20	-1.04	0.3584	0.7541
**ALKALI METAL ION BINDING**	20	0.33	0.95	0.5347	0.7550
**REGULATION OF SISTER CHROMATID COHESION**	17	0.34	0.95	0.5203	0.7551
**SIGNAL RELEASE**	158	-0.22	-1.03	0.3370	0.7566

**NEGATIVE REGULATION OF AXONOGENESIS**	63	-0.26	-1.03	0.3946	0.7567
**ENDOCRINE PANCREAS DEVELOPMENT**	38	0.28	0.95	0.5378	0.7568
**NEGATIVE REGULATION OF INTERLEUKIN 6 PRODUCTION**	31	-0.30	-1.03	0.3883	0.7570
**RESPONSE TO BMP**	87	0.24	0.95	0.5680	0.7577
**ENZYME INHIBITOR ACTIVITY**	307	0.21	0.95	0.5960	0.7578
**REGULATION OF CELL KILLING**	53	0.26	0.95	0.5433	0.7578
**CYTOKINE PRODUCTION**	105	-0.23	-1.03	0.3555	0.7579
**ESTABLISHMENT OR MAINTENANCE OF EPITHELIAL CELL APICAL BASAL POLARITY**	24	0.31	0.95	0.5309	0.7580
**REGULATION OF ORGAN GROWTH**	63	0.26	0.95	0.5572	0.7581
**REGULATION OF P38MAPK CASCADE**	25	0.31	0.95	0.5415	0.7581
**PYRIMIDINE CONTAINING COMPOUND CATABOLIC PROCESS**	24	0.31	0.95	0.5273	0.7584
**NICOTINAMIDE NUCLEOTIDE BIOSYNTHETIC PROCESS**	15	0.35	0.95	0.5195	0.7585
**LENS FIBER CELL DIFFERENTIATION**	24	0.32	0.95	0.5193	0.7587
**NEGATIVE REGULATION OF STAT CASCADE**	43	0.27	0.95	0.5320	0.7587
**POSITIVE REGULATION OF PHOSPHATIDYLINOSITOL 3 KINASE SIGNALING**	58	0.26	0.95	0.5485	0.7598
**NEURONAL POSTSYNAPTIC DENSITY**	47	0.27	0.95	0.5532	0.7600
**DNA STRAND ELONGATION INVOLVED IN DNA REPLICATION**	21	0.32	0.95	0.5406	0.7602
**NEGATIVE REGULATION OF ALCOHOL BIOSYNTHETIC PROCESS**	17	-0.34	-1.03	0.4015	0.7602
**REGULATION OF MYELOID CELL DIFFERENTIATION**	157	-0.21	-1.03	0.3626	0.7604
**METANEPHRIC NEPHRON DEVELOPMENT**	31	-0.30	-1.03	0.3922	0.7607
**POSITIVE REGULATION OF SUBSTRATE ADHESION DEPENDENT CELL SPREADING**	26	0.31	0.95	0.5317	0.7610
**POSITIVE REGULATION OF CELL KILLING**	32	0.29	0.95	0.5349	0.7611
**PLATELET DEGRANULATION**	98	0.24	0.95	0.5637	0.7611
**BETA CATENIN TCF COMPLEX ASSEMBLY**	35	-0.29	-1.03	0.4373	0.7612
**RESPONSE TO MUSCLE STRETCH**	19	-0.34	-1.03	0.4092	0.7612
**APPENDAGE DEVELOPMENT**	155	-0.21	-1.03	0.3827	0.7613
**NEGATIVE REGULATION OF TRANSPORT**	406	0.20	0.95	0.6339	0.7614
**VESICLE LUMEN**	89	-0.24	-1.03	0.3773	0.7615

**FIBRINOLYSIS**	20	0.33	0.95	0.5513	0.7616
**REGULATION OF PHAGOCYTOSIS**	59	-0.25	-1.03	0.4227	0.7617
**NEGATIVE REGULATION OF PROTEIN SECRETION**	93	-0.23	-1.03	0.3842	0.7618
**STRAND DISPLACEMENT**	22	-0.33	-1.03	0.3911	0.7620
**NEGATIVE REGULATION OF STRIATED MUSCLE CELL DIFFERENTIATION**	25	-0.31	-1.03	0.3986	0.7620
**NEGATIVE REGULATION OF CYTOKINE SECRETION**	35	-0.29	-1.03	0.3960	0.7622
**EPITHELIAL CELL PROLIFERATION**	82	-0.24	-1.03	0.3509	0.7622
**REGULATION OF CGMP BIOSYNTHETIC PROCESS**	20	-0.34	-1.03	0.4319	0.7628
**NARROW PORE CHANNEL ACTIVITY**	15	-0.36	-1.03	0.3867	0.7629
**TEMPERATURE HOMEOSTASIS**	23	0.32	0.95	0.5469	0.7630
**GAP JUNCTION CHANNEL ACTIVITY**	15	-0.36	-1.03	0.4123	0.7633
**REGULATION OF CELL MATURATION**	17	0.34	0.94	0.5437	0.7634
**TRANSITION METAL ION TRANSMEMBRANE TRANSPORTER ACTIVITY**	37	-0.28	-1.03	0.3760	0.7636
**SODIUM ION TRANSMEMBRANE TRANSPORTER ACTIVITY**	128	-0.22	-1.03	0.3651	0.7637
**GTPASE BINDING**	273	0.21	0.94	0.6231	0.7637
**DENDRITE DEVELOPMENT**	74	-0.24	-1.03	0.4090	0.7638
**NEGATIVE REGULATION OF NEURAL PRECURSOR CELL PROLIFERATION**	20	-0.33	-1.03	0.4083	0.7639
**REGULATION OF BLOOD PRESSURE**	153	0.22	0.95	0.5919	0.7639
**TRANSMEMBRANE RECEPTOR PROTEIN TYROSINE KINASE ACTIVITY**	62	0.26	0.95	0.5541	0.7639
**METHYL CPG BINDING**	16	-0.35	-1.03	0.4223	0.7641
**NEGATIVE REGULATION OF HEMATOPOIETIC PROGENITOR CELL DIFFERENTIATION**	16	-0.36	-1.02	0.4297	0.7642
**NEGATIVE REGULATION OF TISSUE REMODELING**	17	-0.35	-1.02	0.4123	0.7645
**SOLUTE PROTON SYMPORTER ACTIVITY**	25	-0.31	-1.02	0.3929	0.7647
**NEGATIVE REGULATION OF JAK STAT CASCADE**	43	0.27	0.94	0.5377	0.7652
**REGULATION OF HEART CONTRACTION**	209	0.21	0.94	0.5839	0.7653
**NEGATIVE REGULATION OF RESPONSE TO WOUNDING**	142	0.22	0.94	0.5945	0.7654
**REGULATION OF CELL CYCLE ARREST**	91	0.24	0.94	0.5575	0.7655
**ACTIVATION OF JUN KINASE ACTIVITY**	30	0.30	0.94	0.5382	0.7657
**POSITIVE REGULATION OF BLOOD CIRCULATION**	89	-0.23	-1.02	0.3943	0.7665

**STEROL METABOLIC PROCESS**	107	0.23	0.94	0.6085	0.7666
**CAMP METABOLIC PROCESS**	33	0.29	0.94	0.5353	0.7666
**POTASSIUM CHANNEL REGULATOR ACTIVITY**	43	0.27	0.94	0.5366	0.7675
**RESPONSE TO OXYGEN LEVELS**	284	0.21	0.94	0.6248	0.7676
**OLIGODENDROCYTE DEVELOPMENT**	30	0.30	0.94	0.5546	0.7683
**REGULATION OF ODONTOGENESIS**	23	-0.31	-1.02	0.4161	0.7686
**MICROTUBULE PLUS END**	16	0.34	0.94	0.5394	0.7686
**AXON**	390	0.20	0.94	0.6316	0.7687
**RECEPTOR REGULATOR ACTIVITY**	44	0.27	0.94	0.5585	0.7688
**NEUROMUSCULAR JUNCTION**	49	0.26	0.94	0.5461	0.7688
**CELL DIFFERENTIATION INVOLVED IN EMBRYONIC PLACENTA DEVELOPMENT**	25	0.31	0.94	0.5751	0.7689
**REGULATION OF ENDOCYTOSIS**	179	0.21	0.94	0.6003	0.7689
**CELL MATURATION**	110	0.23	0.94	0.5851	0.7690
**REGULATION OF RESPIRATORY SYSTEM PROCESS**	16	0.35	0.94	0.5290	0.7691
**POSITIVE REGULATION OF CALCIUM ION TRANSMEMBRANE TRANSPORTER ACTIVITY**	31	0.29	0.94	0.5381	0.7691
**EXOCYTOSIS**	278	0.21	0.94	0.6247	0.7692
**REGULATION OF TYROSINE PHOSPHORYLATION OF STAT5 PROTEIN**	19	0.34	0.94	0.5372	0.7693
**REGULATION OF ACTIN NUCLEATION**	25	0.31	0.94	0.5461	0.7693
**CALCIUM MEDIATED SIGNALING**	76	0.25	0.94	0.5857	0.7697
**REGULATION OF ANATOMICAL STRUCTURE SIZE**	427	0.20	0.94	0.6435	0.7698
**MICROTUBULE**	318	0.20	0.94	0.6201	0.7699
**PROTEIN HETERODIMERIZATION ACTIVITY**	376	0.20	0.94	0.6525	0.7701
**POSITIVE REGULATION OF GLUCOSE METABOLIC PROCESS**	34	-0.29	-1.02	0.4077	0.7701
**PROTEIN DNA COMPLEX**	114	-0.22	-1.02	0.3731	0.7704
**INNERVATION**	19	0.33	0.94	0.5262	0.7706
**SENSORY ORGAN MORPHOGENESIS**	228	-0.20	-1.02	0.3722	0.7715
**LIMB DEVELOPMENT**	155	-0.21	-1.02	0.3784	0.7719
**BEHAVIOR**	463	-0.18	-1.02	0.3627	0.7720
**POSITIVE REGULATION OF REPRODUCTIVE PROCESS**	50	0.26	0.94	0.5604	0.7722
**CELL LEADING EDGE**	322	-0.19	-1.02	0.3706	0.7722
**EXTRINSIC APOPTOTIC SIGNALING PATHWAY**	82	0.24	0.94	0.5710	0.7722

**POSTTRANSCRIPTIONAL GENE SILENCING**	40	0.28	0.94	0.5554	0.7723
**REGULATION OF OSTEOCLAST DIFFERENTIATION**	55	-0.26	-1.02	0.4006	0.7725
**REGULATION OF ESTABLISHMENT OR MAINTENANCE OF CELL POLARITY**	20	0.32	0.94	0.5380	0.7726
**NEGATIVE REGULATION OF AXON EXTENSION**	36	-0.28	-1.02	0.4235	0.7729
**REGULATION OF CARDIAC CONDUCTION**	64	0.25	0.94	0.5712	0.7730
**RESPONSE TO AMINE**	43	0.27	0.94	0.5475	0.7730
**NEGATIVE REGULATION OF CELL MORPHOGENESIS INVOLVED IN DIFFERENTIATION**	109	-0.22	-1.02	0.3947	0.7730
**PRESYNAPTIC PROCESS INVOLVED IN SYNAPTIC TRANSMISSION**	105	-0.23	-1.02	0.4151	0.7731
**CAMP BINDING**	22	0.32	0.94	0.5526	0.7732
**BASAL PART OF CELL**	48	0.27	0.94	0.5759	0.7737
**CELLULAR SENESCENCE**	27	0.30	0.94	0.5628	0.7739
**PHOSPHATIDYLETHANOLAMINE METABOLIC PROCESS**	16	0.35	0.94	0.5745	0.7739
**REGULATION OF CYSTEINE TYPE ENDOPEPTIDASE ACTIVITY INVOLVED IN APOPTOTIC SIGNALING PATHWAY**	18	-0.34	-1.02	0.4371	0.7740
**EYE MORPHOGENESIS**	128	0.22	0.93	0.5940	0.7751
**PHOSPHATASE INHIBITOR ACTIVITY**	27	0.30	0.93	0.5484	0.7752
**POSITIVE REGULATION OF BMP SIGNALING PATHWAY**	28	0.30	0.93	0.5638	0.7753
**INTERMEDIATE FILAMENT BASED PROCESS**	40	0.27	0.93	0.5539	0.7753
**ZINC ION TRANSMEMBRANE TRANSPORTER ACTIVITY**	22	0.32	0.93	0.5445	0.7755
**HISTONE LYSINE N METHYLTRANSFERASE ACTIVITY**	41	0.28	0.93	0.5594	0.7755
**NEGATIVE REGULATION OF PROTEIN TYROSINE KINASE ACTIVITY**	19	0.32	0.93	0.5255	0.7756
**PHOSPHOTRANSFERASE ACTIVITY PHOSPHATE GROUP AS ACCEPTOR**	34	0.28	0.93	0.5792	0.7768
**PLATELET ALPHA GRANULE**	71	0.25	0.93	0.5930	0.7769
**REGULATION OF GLIAL CELL PROLIFERATION**	19	0.33	0.93	0.5722	0.7769
**SODIUM ION TRANSPORT**	135	0.22	0.93	0.5942	0.7770
**CARBOXY LYASE ACTIVITY**	35	-0.28	-1.01	0.4163	0.7773

**ANCHORED COMPONENT OF MEMBRANE**	123	-0.22	-1.02	0.4150	0.7775
**DEVELOPMENTAL GROWTH INVOLVED IN MORPHOGENESIS**	98	-0.23	-1.01	0.4199	0.7777
**SCAVENGER RECEPTOR ACTIVITY**	39	-0.27	-1.02	0.4339	0.7778
**TRANSMEMBRANE RECEPTOR PROTEIN SERINE THREONINE KINASE SIGNALING PATHWAY**	177	0.22	0.93	0.6274	0.7779
**NEGATIVE REGULATION OF BEHAVIOR**	15	0.34	0.93	0.5400	0.7780
**REGULATION OF CAMP METABOLIC PROCESS**	119	-0.22	-1.02	0.4022	0.7781
**ANCHORED COMPONENT OF PLASMA MEMBRANE**	30	0.29	0.93	0.5445	0.7781
**PRIMARY CILIUM**	173	-0.21	-1.02	0.3976	0.7784
**BETA AMYLOID BINDING**	32	0.29	0.93	0.5733	0.7784
**REGULATION OF EPITHELIAL CELL PROLIFERATION**	258	0.21	0.93	0.6380	0.7796
**NADP METABOLIC PROCESS**	24	0.31	0.93	0.5607	0.7798
**CELL CORTEX**	208	0.21	0.93	0.6171	0.7801
**NUCLEOBASE CONTAINING COMPOUND TRANSMEMBRANE TRANSPORTER ACTIVITY**	29	0.30	0.93	0.5635	0.7802
**LIPOPOLYSACCHARIDE BINDING**	15	0.35	0.93	0.5547	0.7803
**REGULATION OF MUSCLE ORGAN DEVELOPMENT**	93	0.24	0.93	0.5973	0.7805
**REGULATION OF ORGAN FORMATION**	32	0.28	0.93	0.5535	0.7807
**REGULATION OF SODIUM ION TRANSPORT**	67	0.24	0.93	0.5716	0.7808
**POSITIVE REGULATION OF INTERLEUKIN 2 PRODUCTION**	29	0.29	0.93	0.5668	0.7810
**ATPASE COUPLED ION TRANSMEMBRANE TRANSPORTER ACTIVITY**	67	0.25	0.93	0.5926	0.7826
**SYNAPTIC MEMBRANE**	244	-0.20	-1.01	0.4089	0.7827
**OLFACTORY LOBE DEVELOPMENT**	34	-0.28	-1.01	0.4319	0.7829
**POSITIVE REGULATION OF PHAGOCYTOSIS**	41	-0.27	-1.01	0.4457	0.7830
**CALCIUM ION TRANSPORT**	204	-0.20	-1.01	0.4432	0.7831
**SKELETAL SYSTEM DEVELOPMENT**	423	-0.19	-1.01	0.4198	0.7832
**ICOSANOID TRANSPORT**	20	-0.32	-1.01	0.4408	0.7834
**PLATELET MORPHOGENESIS**	17	-0.34	-1.01	0.4104	0.7834
**GUANOSINE CONTAINING COMPOUND METABOLIC PROCESS**	38	0.27	0.93	0.6000	0.7838

**REGULATION OF DNA DAMAGE RESPONSE SIGNAL TRANSDUCTION BY P53 CLASS MEDIATOR**	27	-0.30	-1.01	0.4152	0.7838
**HETEROCHROMATIN**	62	-0.25	-1.01	0.4318	0.7839
**ARTERY DEVELOPMENT**	72	0.25	0.93	0.6076	0.7839
**NEGATIVE REGULATION OF MUSCLE TISSUE DEVELOPMENT**	31	-0.29	-1.01	0.4430	0.7842
**ER NUCLEUS SIGNALING PATHWAY**	29	0.30	0.93	0.5693	0.7845
**RESPONSE TO HEAT**	76	0.24	0.93	0.5886	0.7847
**POSTSYNAPTIC MEMBRANE**	191	-0.20	-1.01	0.4357	0.7851
**REGULATION OF TRANSCRIPTION INVOLVED IN CELL FATE COMMITMENT**	19	0.32	0.93	0.5379	0.7852
**CEREBRAL CORTEX CELL MIGRATION**	40	-0.28	-1.01	0.4380	0.7853
**NOTCH RECEPTOR PROCESSING**	15	-0.35	-1.01	0.4480	0.7853
**PEPTIDYL TYROSINE DEPHOSPHORYLATION**	94	0.23	0.93	0.6050	0.7853
**RESPONSE TO VITAMIN D**	32	-0.29	-1.01	0.4444	0.7855
**NEGATIVE REGULATION OF DNA BINDING**	38	0.28	0.93	0.5928	0.7856
**HETEROTYPIC CELL CELL ADHESION**	24	-0.30	-1.01	0.4574	0.7862
**CELL PROJECTION MEMBRANE**	264	-0.20	-1.00	0.4688	0.7880
**NEGATIVE REGULATION OF TRANSMEMBRANE RECEPTOR PROTEIN SERINE THREONINE KINASE SIGNALING PATHWAY**	86	0.24	0.92	0.6102	0.7881
**REGULATION OF INTERLEUKIN 12 PRODUCTION**	45	-0.27	-1.01	0.4165	0.7882
**PROTEIN TARGETING TO PLASMA MEMBRANE**	22	-0.32	-1.00	0.4665	0.7883
**CAMERA TYPE EYE PHOTORECEPTOR CELL DIFFERENTIATION**	15	-0.35	-1.01	0.4296	0.7888
**MYELOID DENDRITIC CELL ACTIVATION**	22	-0.32	-1.00	0.4681	0.7888
**PHARYNGEAL SYSTEM DEVELOPMENT**	17	-0.34	-1.01	0.4377	0.7889
**CHONDROCYTE DIFFERENTIATION**	58	-0.25	-1.00	0.4273	0.7891
**NEGATIVE REGULATION OF OSTEOBLAST DIFFERENTIATION**	38	-0.28	-1.00	0.4507	0.7892
**CONTRACTILE FIBER**	191	-0.20	-1.00	0.4083	0.7892
**EXTRACELLULAR MATRIX COMPONENT**	114	-0.22	-1.00	0.4247	0.7893
**MAST CELL MEDIATED IMMUNITY**	17	-0.34	-1.00	0.4576	0.7894
**REGULATION OF GLYCOGEN METABOLIC PROCESS**	31	-0.29	-1.00	0.4586	0.7895
**GLAND MORPHOGENESIS**	94	-0.23	-1.01	0.4218	0.7895
**ENDODEOXYRIBONUCLEASE ACTIVITY**	35	-0.28	-1.00	0.4678	0.7896

**PLASMA MEMBRANE RECEPTOR COMPLEX**	158	-0.21	-1.00	0.4317	0.7896
**NUCLEOSIDE TRIPHOSPHATASE REGULATOR ACTIVITY**	291	-0.19	-1.00	0.4732	0.7896
**ANTERIOR POSTERIOR AXIS SPECIFICATION**	46	-0.27	-1.00	0.4462	0.7898
**CYSTEINE TYPE ENDOPEPTIDASE REGULATOR ACTIVITY INVOLVED IN APOPTOTIC PROCESS**	34	-0.28	-1.00	0.4192	0.7899
**INORGANIC ANION TRANSPORT**	116	-0.22	-1.01	0.4028	0.7899
**POSITIVE REGULATION OF NF KAPPAB IMPORT INTO NUCLEUS**	23	-0.32	-1.00	0.4314	0.7900
**LOW DENSITY LIPOPROTEIN PARTICLE BINDING**	15	-0.35	-1.00	0.4554	0.7900
**POSITIVE REGULATION OF PROTEIN LOCALIZATION TO NUCLEUS**	117	0.22	0.92	0.6223	0.7902
**PASSIVE TRANSMEMBRANE TRANSPORTER ACTIVITY**	420	-0.19	-1.00	0.4552	0.7902
**POSITIVE REGULATION OF INTERLEUKIN 6 PRODUCTION**	61	0.25	0.92	0.5907	0.7903
**HIGH DENSITY LIPOPROTEIN PARTICLE**	21	0.31	0.92	0.5390	0.7903
**PYRIDINE NUCLEOTIDE BIOSYNTHETIC PROCESS**	15	0.35	0.92	0.5605	0.7904
**INFLAMMATORY RESPONSE TO ANTIGENIC STIMULUS**	23	-0.31	-1.00	0.4567	0.7907
**RESPONSE TO STEROL**	21	-0.31	-1.00	0.4344	0.7926
**NEGATIVE REGULATION OF NF KAPPAB TRANSCRIPTION FACTOR ACTIVITY**	53	0.25	0.92	0.6035	0.7942
**EPITHELIAL TO MESENCHYMAL TRANSITION**	54	0.26	0.92	0.5894	0.7943
**INOSITOL PHOSPHATE MEDIATED SIGNALING**	17	0.34	0.92	0.5729	0.7943
**LACTATION**	40	-0.26	-1.00	0.4655	0.7943
**SPINAL CORD MOTOR NEURON DIFFERENTIATION**	31	-0.28	-1.00	0.4450	0.7943
**POSITIVE REGULATION OF LEUKOCYTE MEDIATED IMMUNITY**	72	-0.24	-1.00	0.4790	0.7944
**RESPONSE TO ANGIOTENSIN**	15	-0.35	-1.00	0.4512	0.7945
**NEUROTRANSMITTER RECEPTOR ACTIVITY**	61	-0.25	-1.00	0.4583	0.7946
**REGULATION OF SKELETAL MUSCLE CELL DIFFERENTIATION**	15	-0.35	-1.00	0.4736	0.7947

**TRANSCRIPTIONAL REPRESSOR ACTIVITY RNA POLYMERASE II TRANSCRIPTION REGULATORY REGION SEQUENCE SPECIFIC BINDING**	145	-0.21	-1.00	0.4415	0.7947
**PEPTIDE CATABOLIC PROCESS**	21	0.31	0.92	0.5561	0.7948
**CELL SURFACE RECEPTOR SIGNALING PATHWAY INVOLVED IN HEART DEVELOPMENT**	16	-0.34	-1.00	0.4447	0.7948
**EXTRINSIC COMPONENT OF PLASMA MEMBRANE**	125	-0.22	-1.00	0.4526	0.7949
**REGULATION OF PROTEIN ACTIVATION CASCADE**	29	-0.29	-1.00	0.4442	0.7949
**REGULATION OF CYTOKINE SECRETION**	112	-0.22	-0.99	0.4605	0.7951
**EXTRINSIC APOPTOTIC SIGNALING PATHWAY VIA DEATH DOMAIN RECEPTORS**	29	-0.29	-1.00	0.4730	0.7952
**HEPARIN BINDING**	138	-0.21	-1.00	0.4667	0.7958
**SYNAPTONEMAL COMPLEX ORGANIZATION**	16	-0.35	-0.99	0.4528	0.7959
**NEGATIVE REGULATION OF CELL CYCLE G1 S PHASE TRANSITION**	81	0.24	0.92	0.6136	0.7970
**NEURONAL ACTION POTENTIAL**	26	-0.30	-0.99	0.4716	0.7971
**SUPEROXIDE METABOLIC PROCESS**	31	0.29	0.92	0.6180	0.7971
**POSITIVE REGULATION OF DNA BIOSYNTHETIC PROCESS**	53	0.26	0.92	0.5714	0.7973
**MULTICELLULAR ORGANISMAL SIGNALING**	116	-0.22	-0.99	0.4919	0.7978
**REGULATION OF SMALL GTPASE MEDIATED SIGNAL TRANSDUCTION**	242	-0.20	-0.99	0.4820	0.7980
**CALCIUM CHANNEL REGULATOR ACTIVITY**	36	0.28	0.92	0.6065	0.7983
**MULTI ORGANISM REPRODUCTIVE BEHAVIOR**	20	-0.31	-0.99	0.4939	0.7991
**REGULATION OF T CELL MEDIATED IMMUNITY**	44	0.27	0.92	0.5839	0.8006
**NEUROMUSCULAR PROCESS CONTROLLING BALANCE**	47	-0.26	-0.99	0.4613	0.8008
**FATTY ACID DERIVATIVE TRANSPORT**	20	-0.32	-0.99	0.4714	0.8009
**MUSCULOSKELETAL MOVEMENT**	40	-0.27	-0.99	0.4571	0.8010
**REGULATION OF CYTOKINE PRODUCTION**	480	-0.18	-0.99	0.5000	0.8011
**INTERMEDIATE FILAMENT ORGANIZATION**	20	-0.31	-0.99	0.4758	0.8013
**SMAD BINDING**	65	-0.24	-0.99	0.4724	0.8015
**T CELL PROLIFERATION**	31	0.28	0.92	0.5921	0.8016

**POSITIVE REGULATION OF PROTEIN TYROSINE KINASE ACTIVITY**	34	-0.28	-0.99	0.4925	0.8017
**DEVELOPMENT OF PRIMARY SEXUAL CHARACTERISTICS**	184	-0.20	-0.99	0.5057	0.8020
**ACTIN MEDIATED CELL CONTRACTION**	69	-0.24	-0.99	0.4535	0.8023
**NOTOCHORD DEVELOPMENT**	18	-0.32	-0.99	0.4654	0.8024
**RESPONSE TO PURINE CONTAINING COMPOUND**	148	-0.20	-0.99	0.5037	0.8024
**MULTICELLULAR ORGANISMAL MOVEMENT**	40	-0.27	-0.99	0.4566	0.8026
**EXOCYTIC VESICLE**	130	0.22	0.92	0.6278	0.8029
**REGULATION OF RECEPTOR MEDIATED ENDOCYTOSIS**	70	0.24	0.92	0.6184	0.8031
**REGULATION OF MITOCHONDRIAL MEMBRANE PERMEABILITY INVOLVED IN APOPTOTIC PROCESS**	16	0.34	0.92	0.5749	0.8032
**REGULATION OF AMINO ACID TRANSPORT**	25	0.30	0.92	0.5892	0.8035
**CILIUM ORGANIZATION**	144	0.22	0.92	0.6474	0.8035
**CHROMATIN SILENCING AT RDNA**	18	0.33	0.92	0.5739	0.8038
**CORE PROMOTER PROXIMAL REGION DNA BINDING**	289	-0.19	-0.98	0.5079	0.8043
**POSITIVE REGULATION OF CELL DIVISION**	117	-0.21	-0.99	0.5017	0.8043
**POSITIVE REGULATION OF CYCLIC NUCLEOTIDE METABOLIC PROCESS**	100	-0.22	-0.99	0.4774	0.8044
**VENTRICULAR CARDIAC MUSCLE CELL ACTION POTENTIAL**	15	-0.35	-0.99	0.4735	0.8044
**LEARNING**	122	-0.22	-0.99	0.4928	0.8052
**OVULATION CYCLE PROCESS**	80	-0.23	-0.99	0.4711	0.8055
**SPHINGOLIPID BIOSYNTHETIC PROCESS**	65	0.24	0.91	0.6092	0.8057
**CLATHRIN MEDIATED ENDOCYTOSIS**	35	0.28	0.91	0.5648	0.8058
**RNA POLYMERASE II TRANSCRIPTION COFACTOR ACTIVITY**	83	0.23	0.91	0.6114	0.8060
**CYSTEINE TYPE ENDOPEPTIDASE INHIBITOR ACTIVITY**	40	-0.27	-0.99	0.4721	0.8060
**POSITIVE REGULATION OF T CELL MEDIATED IMMUNITY**	27	0.29	0.91	0.5793	0.8061
**CYCLIN DEPENDENT PROTEIN KINASE ACTIVITY**	30	0.28	0.91	0.6034	0.8064
**COGNITION**	226	0.21	0.91	0.6900	0.8065
**RENAL SYSTEM VASCULATURE DEVELOPMENT**	19	0.32	0.91	0.5743	0.8067

**NEGATIVE REGULATION OF GROWTH**	209	0.21	0.91	0.6827	0.8067
**VERY LOW DENSITY LIPOPROTEIN PARTICLE**	16	0.33	0.91	0.5827	0.8067
**METALLOCARBOXYPEPTIDASE ACTIVITY**	25	-0.29	-0.99	0.4758	0.8067
**REGULATION OF PROTEIN DEACETYLATION**	28	0.29	0.91	0.6144	0.8067
**NEGATIVE REGULATION OF RESPONSE TO DNA DAMAGE STIMULUS**	45	0.26	0.91	0.5918	0.8067
**REGULATION OF SYNAPSE STRUCTURE OR ACTIVITY**	219	0.20	0.91	0.6706	0.8068
**DEVELOPMENTAL GROWTH**	302	0.20	0.91	0.7099	0.8070
**OXIDOREDUCTASE ACTIVITY ACTING ON THE CH NH2 GROUP OF DONORS**	16	0.33	0.91	0.5888	0.8070
**EPITHELIAL CELL DEVELOPMENT**	172	0.21	0.91	0.6635	0.8070
**DEOXYRIBONUCLEASE ACTIVITY**	49	-0.25	-0.98	0.4826	0.8070
**PROTEIN TYROSINE PHOSPHATASE ACTIVITY**	97	0.23	0.91	0.6257	0.8072
**NEUROTRANSMITTER METABOLIC PROCESS**	22	0.31	0.91	0.5946	0.8073
**RESPONSE TO AMINO ACID**	99	0.23	0.91	0.6366	0.8073
**SOLUTE SODIUM SYMPORTER ACTIVITY**	46	0.26	0.91	0.5895	0.8080
**ENTEROENDOCRINE CELL DIFFERENTIATION**	19	-0.32	-0.98	0.4582	0.8084
**POSITIVE REGULATION OF CYTOKINE PRODUCTION**	316	-0.19	-0.98	0.5077	0.8084
**REGULATION OF BIOMINERAL TISSUE DEVELOPMENT**	68	-0.24	-0.98	0.4715	0.8087
**NEGATIVE REGULATION OF CELL GROWTH**	156	0.21	0.91	0.6539	0.8104
**BRAIN MORPHOGENESIS**	32	0.28	0.91	0.6151	0.8111
**DEAMINASE ACTIVITY**	25	0.29	0.91	0.6021	0.8113
**CELLULAR RESPONSE TO LITHIUM ION**	17	0.33	0.91	0.5722	0.8113
**COP9 SIGNALOSOME**	28	0.29	0.91	0.5973	0.8114
**PHOSPHOLIPID TRANSLOCATING ATPASE ACTIVITY**	16	0.33	0.91	0.5949	0.8116
**POSTREPLICATION REPAIR**	43	0.26	0.91	0.5987	0.8116
**SODIUM CHANNEL REGULATOR ACTIVITY**	30	0.29	0.91	0.5932	0.8117
**MAIN AXON**	50	0.26	0.91	0.6018	0.8120
**NEGATIVE REGULATION OF VIRAL TRANSCRIPTION**	21	0.31	0.91	0.5784	0.8122
**QUATERNARY AMMONIUM GROUP BINDING**	44	-0.26	-0.98	0.4761	0.8123

**MICROVILLUS ORGANIZATION**	18	-0.33	-0.98	0.4863	0.8123
**REGULATION OF TYROSINE PHOSPHORYLATION OF STAT PROTEIN**	63	0.24	0.91	0.6235	0.8128
**INNER EAR RECEPTOR STEREOCILIUM ORGANIZATION**	20	-0.31	-0.98	0.4528	0.8129
**MACROLIDE BINDING**	16	0.34	0.91	0.5851	0.8131
**NEGATIVE REGULATION OF PROTEOLYSIS**	267	0.20	0.91	0.7036	0.8133
**POSITIVE REGULATION OF EPITHELIAL CELL MIGRATION**	96	0.23	0.91	0.6383	0.8133
**POSITIVE REGULATION OF RESPONSE TO WOUNDING**	140	-0.21	-0.98	0.5110	0.8133
**NEURAL TUBE FORMATION**	87	0.23	0.91	0.6350	0.8134
**RESPONSE TO GONADOTROPIN**	25	0.29	0.91	0.5829	0.8135
**REGULATION OF FEEDING BEHAVIOR**	19	0.31	0.91	0.6096	0.8135
**REGULATION OF POSTSYNAPTIC MEMBRANE POTENTIAL**	54	0.25	0.91	0.6315	0.8136
**POSITIVE REGULATION OF MICROTUBULE POLYMERIZATION OR DEPOLYMERIZATION**	22	0.31	0.91	0.6034	0.8137
**PLATELET AGGREGATION**	36	-0.27	-0.98	0.4974	0.8137
**FK506 BINDING**	16	0.34	0.91	0.5697	0.8138
**HEART TRABECULA MORPHOGENESIS**	25	0.29	0.91	0.5889	0.8138
**REGULATION OF MUSCLE CELL DIFFERENTIATION**	142	0.21	0.91	0.6806	0.8138
**DEVELOPMENTAL PROGRAMMED CELL DEATH**	20	-0.32	-0.98	0.4629	0.8138
**REGULATION OF PEPTIDYL TYROSINE PHOSPHORYLATION**	194	0.20	0.91	0.6881	0.8138
**REGULATION OF EPITHELIAL TO MESENCHYMAL TRANSITION**	61	0.25	0.90	0.6157	0.8148
**CELLULAR RESPONSE TO PH**	15	-0.34	-0.98	0.4881	0.8149
**CYCLIC NUCLEOTIDE MEDIATED SIGNALING**	45	0.26	0.90	0.6124	0.8158
**POSITIVE REGULATION OF NUCLEOCYTOPLASMIC TRANSPORT**	111	0.22	0.90	0.6662	0.8167
**HINDBRAIN MORPHOGENESIS**	38	-0.27	-0.98	0.4706	0.8170
**POSITIVE REGULATION OF LIPID TRANSPORT**	48	0.26	0.90	0.6077	0.8171
**SKELETAL SYSTEM MORPHOGENESIS**	186	-0.20	-0.98	0.5160	0.8173

**OXIDOREDUCTASE ACTIVITY ACTING ON PAIRED DONORS WITH INCORPORATION OR REDUCTION OF MOLECULAR OXYGEN NAD P H AS ONE DONOR AND INCORPORATION OF ONE ATOM OF OXYGEN**	24	-0.30	-0.98	0.4774	0.8179
**SMOOTH MUSCLE CELL DIFFERENTIATION**	29	0.28	0.90	0.6174	0.8181
**GLANDULAR EPITHELIAL CELL DIFFERENTIATION**	38	0.27	0.90	0.6149	0.8183
**REGULATION OF IMMUNE EFFECTOR PROCESS**	349	0.19	0.90	0.7347	0.8185
**VENTRICULAR CARDIAC MUSCLE TISSUE DEVELOPMENT**	43	-0.26	-0.98	0.5065	0.8186
**REGULATION OF ERK1 AND ERK2 CASCADE**	208	-0.20	-0.98	0.5190	0.8186
**REGULATION OF VACUOLAR TRANSPORT**	27	0.29	0.90	0.6082	0.8188
**REGULATION OF MAST CELL ACTIVATION INVOLVED IN IMMUNE RESPONSE**	26	-0.30	-0.97	0.5025	0.8193
**SENSORY ORGAN DEVELOPMENT**	463	-0.18	-0.97	0.5822	0.8195
**ORGANIC ACID BINDING**	175	0.21	0.90	0.7130	0.8198
**CONNECTIVE TISSUE DEVELOPMENT**	185	-0.20	-0.98	0.5413	0.8199
**OXIDOREDUCTASE ACTIVITY ACTING ON PAIRED DONORS WITH INCORPORATION OR REDUCTION OF MOLECULAR OXYGEN 2 OXOGLUTARATE AS ONE DONOR AND INCORPORATION OF ONE ATOM EACH OF OXYGEN INTO BOTH DONORS**	39	-0.27	-0.97	0.4756	0.8200
**ENDOCHONDRAL OSSIFICATION**	24	0.29	0.90	0.6047	0.8201
**NEGATIVE REGULATION OF CELLULAR RESPONSE TO TRANSFORMING GROWTH FACTOR BETA STIMULUS**	54	0.25	0.90	0.6128	0.8201
**TRANSCRIPTIONAL REPRESSOR ACTIVITY RNA POLYMERASE II TRANSCRIPTION FACTOR BINDING**	80	-0.23	-0.97	0.5117	0.8203
**REGULATION OF CALCIUM ION IMPORT**	93	-0.22	-0.97	0.5250	0.8206
**POSITIVE REGULATION OF CATION TRANSMEMBRANE TRANSPORT**	89	-0.23	-0.97	0.5235	0.8206
**PALATE DEVELOPMENT**	78	-0.23	-0.98	0.5126	0.8206
**NEGATIVE REGULATION OF CELL MATRIX ADHESION**	26	-0.29	-0.97	0.5035	0.8209

**C21 STEROID HORMONE METABOLIC PROCESS**	20	-0.32	-0.97	0.5228	0.8215
**FOREBRAIN GENERATION OF NEURONS**	64	-0.23	-0.97	0.5084	0.8216
**RESPONSE TO MINERALOCORTICOID**	34	-0.27	-0.97	0.5124	0.8216
**RESPONSE TO DSRNA**	59	0.25	0.90	0.6160	0.8217
**SPROUTING ANGIOGENESIS**	44	-0.26	-0.97	0.5079	0.8220
**WIDE PORE CHANNEL ACTIVITY**	22	0.30	0.90	0.5923	0.8221
**ENTRAINMENT OF CIRCADIAN CLOCK BY PHOTOPERIOD**	17	0.33	0.90	0.6048	0.8221
**HOMOTYPIC CELL CELL ADHESION**	48	-0.25	-0.97	0.5273	0.8224
**DIVALENT INORGANIC CATION TRANSMEMBRANE TRANSPORTER ACTIVITY**	157	-0.20	-0.97	0.5424	0.8228
**REGULATION OF CELL MATRIX ADHESION**	82	-0.23	-0.97	0.5100	0.8230
**NEURON PROJECTION DEVELOPMENT**	484	-0.18	-0.97	0.5691	0.8231
**RESPONSE TO KETONE**	166	0.21	0.90	0.6957	0.8236
**BONE RESORPTION**	19	0.32	0.90	0.5956	0.8237
**REGULATION OF RESPONSE TO WOUNDING**	364	0.19	0.90	0.7608	0.8237
**NEGATIVE REGULATION OF MUSCLE CELL DIFFERENTIATION**	53	0.25	0.90	0.6187	0.8238
**TISSUE REGENERATION**	47	0.25	0.90	0.6174	0.8239
**REGULATION OF VASCULAR ENDOTHELIAL GROWTH FACTOR PRODUCTION**	29	0.29	0.90	0.6145	0.8239
**WW DOMAIN BINDING**	25	0.29	0.90	0.6130	0.8241
**REGULATION OF NATURAL KILLER CELL ACTIVATION**	22	-0.31	-0.97	0.5059	0.8242
**CONDENSED NUCLEAR CHROMOSOME**	75	0.23	0.90	0.6519	0.8243
**NEGATIVE REGULATION OF ESTABLISHMENT OF PROTEIN LOCALIZATION**	181	0.21	0.90	0.6927	0.8243
**NEGATIVE REGULATION OF RECEPTOR ACTIVITY**	27	0.29	0.90	0.6000	0.8243
**SIGNAL TRANSDUCTION IN RESPONSE TO DNA DAMAGE**	82	0.23	0.90	0.6628	0.8243
**LAMELLIPODIUM ORGANIZATION**	35	0.27	0.90	0.5944	0.8243
**EYE DEVELOPMENT**	304	0.19	0.90	0.7454	0.8244
**MYELOID LEUKOCYTE DIFFERENTIATION**	88	0.23	0.90	0.6491	0.8244
**GUANYL NUCLEOTIDE EXCHANGE FACTOR ACTIVITY**	262	0.20	0.90	0.7367	0.8246
**GENETIC IMPRINTING**	18	0.32	0.90	0.5870	0.8247

**PHOSPHATIDYLINOSITOL 4 PHOSPHATE BINDING**	19	0.31	0.90	0.6122	0.8248
**BONE MORPHOGENESIS**	74	-0.23	-0.97	0.4884	0.8248
**REGULATION OF CATION CHANNEL ACTIVITY**	84	0.23	0.90	0.6667	0.8248
**REGULATION OF T CELL CYTOKINE PRODUCTION**	18	0.32	0.89	0.5974	0.8248
**RESPONSE TO ANTIBIOTIC**	42	0.27	0.89	0.6390	0.8249
**POSITIVE REGULATION OF PROTEIN OLIGOMERIZATION**	18	-0.32	-0.97	0.4717	0.8250
**REGULATION OF CELLULAR RESPONSE TO INSULIN STIMULUS**	53	-0.25	-0.97	0.5245	0.8251
**KIDNEY VASCULATURE DEVELOPMENT**	19	0.32	0.90	0.6161	0.8251
**REGULATION OF CATENIN IMPORT INTO NUCLEUS**	26	0.29	0.90	0.5986	0.8253
**POSITIVE REGULATION OF CALCIUM ION TRANSPORT INTO CYTOSOL**	48	-0.25	-0.97	0.5346	0.8256
**MUSCLE STRUCTURE DEVELOPMENT**	400	-0.18	-0.97	0.5714	0.8258
**MIRNA BINDING**	16	-0.33	-0.97	0.5023	0.8259
**PROTEOGLYCAN BIOSYNTHETIC PROCESS**	56	0.24	0.89	0.6425	0.8259
**MATING BEHAVIOR**	20	-0.31	-0.96	0.5245	0.8261
**ACTIN FILAMENT BASED PROCESS**	404	-0.18	-0.97	0.6331	0.8263
**REGULATION OF ACTIVATED T CELL PROLIFERATION**	32	-0.27	-0.96	0.5066	0.8267
**BILE ACID AND BILE SALT TRANSPORT**	23	-0.30	-0.96	0.4903	0.8267
**REGULATION OF INTRINSIC APOPTOTIC SIGNALING PATHWAY IN RESPONSE TO DNA DAMAGE**	29	-0.28	-0.96	0.5327	0.8269
**VENTRICULAR SEPTUM MORPHOGENESIS**	28	0.28	0.89	0.6246	0.8270
**LABYRINTHINE LAYER BLOOD VESSEL DEVELOPMENT**	18	0.31	0.89	0.6317	0.8270
**RESPONSE TO OSMOTIC STRESS**	57	-0.24	-0.97	0.5123	0.8271
**ANATOMICAL STRUCTURE MATURATION**	35	-0.27	-0.97	0.4962	0.8272
**MITOTIC SPINDLE ASSEMBLY**	33	0.27	0.89	0.6053	0.8273
**REGULATION OF MAST CELL DEGRANULATION**	26	-0.30	-0.97	0.4905	0.8273
**CORE PROMOTER SEQUENCE SPECIFIC DNA BINDING**	87	0.23	0.89	0.6476	0.8274
**STARTLE RESPONSE**	24	-0.29	-0.97	0.4965	0.8274
**CILIARY BASAL BODY**	66	0.24	0.89	0.6530	0.8274

**CARDIAC VENTRICLE MORPHOGENESIS**	57	-0.24	-0.97	0.5387	0.8276
**RESPONSE TO PROGESTERONE**	44	-0.25	-0.96	0.4744	0.8277
**NEGATIVE REGULATION OF ENDOPLASMIC RETICULUM STRESS INDUCED INTRINSIC APOPTOTIC SIGNALING PATHWAY**	17	0.33	0.89	0.6293	0.8278
**REGULATION OF REACTIVE OXYGEN SPECIES BIOSYNTHETIC PROCESS**	58	-0.24	-0.96	0.5324	0.8280
**CORONARY VASCULATURE DEVELOPMENT**	35	0.27	0.89	0.6466	0.8281
**REGULATION OF MULTICELLULAR ORGANISM GROWTH**	60	0.24	0.89	0.6443	0.8281
**NEURAL CREST CELL MIGRATION**	49	0.25	0.89	0.6486	0.8282
**HEART GROWTH**	24	0.29	0.89	0.6117	0.8282
**SODIUM ION TRANSMEMBRANE TRANSPORT**	87	0.23	0.89	0.6844	0.8282
**CELLULAR RESPONSE TO ABIOTIC STIMULUS**	228	0.20	0.89	0.7388	0.8283
**REGULATION OF DNA TEMPLATED TRANSCRIPTION IN RESPONSE TO STRESS**	60	0.24	0.89	0.6524	0.8284
**POSITIVE REGULATION OF CELLULAR RESPONSE TO TRANSFORMING GROWTH FACTOR BETA STIMULUS**	24	0.30	0.89	0.5906	0.8285
**POSITIVE REGULATION OF TRANSFORMING GROWTH FACTOR BETA RECEPTOR SIGNALING PATHWAY**	24	0.30	0.89	0.6088	0.8286
**RETINA MORPHOGENESIS IN CAMERA TYPE EYE**	42	-0.26	-0.96	0.5302	0.8286
**POSITIVE REGULATION OF NATURAL KILLER CELL ACTIVATION**	16	-0.33	-0.96	0.5304	0.8287
**TRIGLYCERIDE RICH LIPOPROTEIN PARTICLE**	16	0.33	0.89	0.6124	0.8287
**GASTRULATION**	140	0.21	0.89	0.7106	0.8288
**POSITIVE REGULATION OF BEHAVIOR**	24	-0.29	-0.96	0.5138	0.8288
**NEGATIVE REGULATION OF TRANSFORMING GROWTH FACTOR BETA RECEPTOR SIGNALING PATHWAY**	54	0.25	0.89	0.6334	0.8289
**PHOSPHATIDYLINOSITOL 3 4 BISPHOSPHATE BINDING**	18	-0.33	-0.96	0.5211	0.8292
**ION GATED CHANNEL ACTIVITY**	40	-0.25	-0.96	0.5230	0.8294
**POSITIVE REGULATION OF CHONDROCYTE DIFFERENTIATION**	19	-0.31	-0.96	0.5332	0.8296

**NEUROMUSCULAR SYNAPTIC TRANSMISSION**	26	0.29	0.89	0.6070	0.8298
**POSITIVE REGULATION OF ACTIN FILAMENT BUNDLE ASSEMBLY**	46	0.26	0.89	0.6302	0.8299
**DOPAMINERGIC NEURON DIFFERENTIATION**	27	-0.29	-0.96	0.5155	0.8300
**REGULATION OF GENE EXPRESSION BY GENETIC IMPRINTING**	15	0.33	0.89	0.6061	0.8315
**POSITIVE REGULATION OF HOMEOSTATIC PROCESS**	191	0.20	0.89	0.7041	0.8318
**REGULATION OF REGULATED SECRETORY PATHWAY**	114	-0.20	-0.96	0.5106	0.8320
**POSITIVE REGULATION OF SECRETION**	323	0.19	0.89	0.7712	0.8321
**REGULATION OF POLYSACCHARIDE METABOLIC PROCESS**	39	-0.26	-0.96	0.5087	0.8323
**PROTEIN LIPID COMPLEX BINDING**	22	-0.30	-0.96	0.4809	0.8326
**REGULATION OF CELL MORPHOGENESIS INVOLVED IN DIFFERENTIATION**	316	0.19	0.89	0.7843	0.8331
**MICROTUBULE CYTOSKELETON ORGANIZATION INVOLVED IN MITOSIS**	33	0.27	0.89	0.6238	0.8342
**HEMOSTASIS**	265	-0.19	-0.96	0.6448	0.8350
**DORSAL SPINAL CORD DEVELOPMENT**	18	0.32	0.89	0.6170	0.8351
**INTRACILIARY TRANSPORT PARTICLE**	27	0.29	0.89	0.6189	0.8352
**NEPHRON DEVELOPMENT**	109	-0.21	-0.96	0.5401	0.8352
**DEVELOPMENTAL CELL GROWTH**	71	-0.23	-0.96	0.5422	0.8352
**ADENYLATE CYCLASE INHIBITING G PROTEIN COUPLED RECEPTOR SIGNALING PATHWAY**	63	-0.23	-0.96	0.5197	0.8360
**FACE DEVELOPMENT**	47	0.25	0.89	0.6527	0.8363
**ADENYLATE CYCLASE MODULATING G PROTEIN COUPLED RECEPTOR SIGNALING PATHWAY**	130	-0.21	-0.96	0.5677	0.8363
**REGULATION OF MEMBRANE POTENTIAL**	325	0.19	0.88	0.7716	0.8375
**RESPONSE TO DEXAMETHASONE**	30	0.28	0.88	0.6401	0.8384
**PEPTIDYL LYSINE TRIMETHYLATION**	17	0.32	0.88	0.6177	0.8389
**REGULATION OF CELL FATE COMMITMENT**	24	0.29	0.88	0.6221	0.8397
**CELLULAR RESPONSE TO NUTRIENT**	38	0.26	0.88	0.6633	0.8399
**DNA BINDING BENDING**	20	-0.31	-0.95	0.5187	0.8399
**ERBB2 SIGNALING PATHWAY**	35	0.27	0.88	0.6483	0.8400
**UTERUS DEVELOPMENT**	18	-0.31	-0.95	0.5151	0.8401

**POSITIVE REGULATION OF TOLL LIKE RECEPTOR SIGNALING PATHWAY**	18	0.32	0.88	0.6277	0.8401
**ACTIVATION OF ADENYLATE CYCLASE ACTIVITY**	35	0.27	0.88	0.6486	0.8402
**REGULATION OF PLATELET AGGREGATION**	15	0.33	0.88	0.6136	0.8404
**POSITIVE REGULATION OF CAMP METABOLIC PROCESS**	82	-0.22	-0.95	0.5537	0.8405
**NEGATIVE REGULATION OF POTASSIUM ION TRANSMEMBRANE TRANSPORTER ACTIVITY**	16	-0.33	-0.95	0.5406	0.8406
**GANGLIOSIDE METABOLIC PROCESS**	23	-0.29	-0.95	0.5263	0.8408
**DEVELOPMENTAL MATURATION**	168	0.20	0.88	0.7058	0.8410
**REGULATION OF CATION TRANSMEMBRANE TRANSPORT**	190	-0.19	-0.95	0.5859	0.8412
**MATERNAL PROCESS INVOLVED IN FEMALE PREGNANCY**	54	-0.24	-0.95	0.5567	0.8413
**SERTOLI CELL DIFFERENTIATION**	17	-0.32	-0.95	0.5024	0.8414
**SYNAPTIC VESICLE LOCALIZATION**	98	-0.21	-0.95	0.5531	0.8415
**CELL KILLING**	29	-0.28	-0.95	0.5371	0.8415
**IONOTROPIC GLUTAMATE RECEPTOR COMPLEX**	44	-0.25	-0.95	0.5118	0.8415
**REGULATION OF EPITHELIAL CELL MIGRATION**	157	-0.20	-0.95	0.6194	0.8419
**NEGATIVE REGULATION OF EXTRINSIC APOPTOTIC SIGNALING PATHWAY**	91	0.22	0.88	0.6574	0.8421
**POSITIVE REGULATION OF TRANSPORTER ACTIVITY**	70	0.23	0.88	0.6528	0.8422
**NEURAL TUBE DEVELOPMENT**	138	-0.20	-0.95	0.5779	0.8422
**POSITIVE REGULATION OF PHOSPHOPROTEIN PHOSPHATASE ACTIVITY**	16	-0.33	-0.95	0.5159	0.8428
**NEGATIVE REGULATION OF INFLAMMATORY RESPONSE**	89	0.22	0.88	0.6770	0.8438
**SH3 SH2 ADAPTOR ACTIVITY**	46	0.25	0.88	0.6508	0.8438
**AXON PART**	204	0.20	0.88	0.7522	0.8453
**PANCREAS DEVELOPMENT**	67	0.23	0.88	0.6641	0.8453
**GLOMERULUS DEVELOPMENT**	47	0.25	0.88	0.6651	0.8454
**GLYCOSPHINGOLIPID METABOLIC PROCESS**	58	0.24	0.88	0.6703	0.8455
**ADENYLYLTRANSFERASE ACTIVITY**	20	0.31	0.88	0.6170	0.8456
**POSITIVE REGULATION OF RESPONSE TO BIOTIC STIMULUS**	36	0.26	0.88	0.6455	0.8456

**MULTIVESICULAR BODY**	32	0.27	0.88	0.6512	0.8461
**CELLULAR RESPONSE TO INTERLEUKIN 6**	19	0.30	0.88	0.6181	0.8463
**REGULATION OF PROTEIN EXIT FROM ENDOPLASMIC RETICULUM**	17	0.31	0.88	0.6043	0.8464
**OSSIFICATION**	233	0.20	0.88	0.7686	0.8465
**DEATH RECEPTOR BINDING**	15	0.32	0.88	0.6293	0.8466
**NEGATIVE REGULATION OF SYNAPTIC TRANSMISSION**	57	0.24	0.88	0.6526	0.8472
**MONOVALENT INORGANIC CATION TRANSPORT**	394	0.19	0.88	0.8031	0.8472
**REPLACEMENT OSSIFICATION**	24	0.29	0.88	0.6728	0.8473
**REGULATION OF NOTCH SIGNALING PATHWAY**	58	-0.24	-0.95	0.5503	0.8480
**CD4 POSITIVE ALPHA BETA T CELL ACTIVATION**	33	-0.27	-0.95	0.5402	0.8482
**PLASMA MEMBRANE ORGANIZATION**	183	0.20	0.87	0.7601	0.8485
**REGULATION OF CELL SIZE**	160	-0.20	-0.95	0.6250	0.8486
**REGULATION OF OSSIFICATION**	162	0.20	0.87	0.7397	0.8486
**SIALYLTRANSFERASE ACTIVITY**	18	-0.32	-0.95	0.5385	0.8486
**HISTONE DEACETYLASE COMPLEX**	55	0.24	0.87	0.6799	0.8486
**INSULIN LIKE GROWTH FACTOR BINDING**	25	-0.28	-0.95	0.5094	0.8487
**G PROTEIN ALPHA SUBUNIT BINDING**	19	0.31	0.87	0.6267	0.8489
**BRANCHING INVOLVED IN URETERIC BUD MORPHOGENESIS**	40	0.26	0.87	0.6388	0.8491
**POSITIVE REGULATION OF ANION TRANSPORT**	56	-0.24	-0.95	0.5430	0.8491
**POSITIVE REGULATION OF CELL SUBSTRATE ADHESION**	92	-0.21	-0.95	0.5770	0.8492
**TRANSPORTER COMPLEX**	295	-0.18	-0.95	0.6750	0.8497
**REGULATION OF ENDOTHELIAL CELL PROLIFERATION**	91	0.22	0.87	0.6869	0.8499
**MEGAKARYOCYTE DIFFERENTIATION**	18	0.32	0.87	0.6318	0.8504
**REGULATION OF MUSCLE ADAPTATION**	59	0.24	0.87	0.6733	0.8504
**BONE REMODELING**	33	0.27	0.87	0.6645	0.8515
**REGULATION OF INTERLEUKIN 8 SECRETION**	15	0.33	0.87	0.6133	0.8515
**CILIARY TRANSITION ZONE**	18	0.31	0.87	0.6310	0.8517
**AMELOGENESIS**	20	0.31	0.87	0.6373	0.8518
**SCHWANN CELL DEVELOPMENT**	24	0.29	0.87	0.6582	0.8522
**REGULATION OF PROTEIN EXPORT FROM NUCLEUS**	29	-0.28	-0.94	0.5488	0.8529

**NEGATIVE REGULATION OF EXTRINSIC APOPTOTIC SIGNALING PATHWAY VIA DEATH DOMAIN RECEPTORS**	31	0.27	0.87	0.6632	0.8532
**POSITIVE REGULATION OF CELLULAR RESPONSE TO INSULIN STIMULUS**	20	-0.31	-0.94	0.5346	0.8533
**ERBB SIGNALING PATHWAY**	74	0.23	0.87	0.6957	0.8536
**BLOOD VESSEL ENDOTHELIAL CELL MIGRATION**	23	-0.29	-0.94	0.5467	0.8536
**SARCOPLASMIC RETICULUM MEMBRANE**	34	0.27	0.87	0.6738	0.8537
**RESPONSE TO BACTERIUM**	388	-0.18	-0.94	0.6513	0.8539
**CALCIUM ION TRANSMEMBRANE IMPORT INTO CYTOSOL**	35	-0.26	-0.94	0.5510	0.8547
**CALCIUM ION IMPORT INTO CYTOSOL**	35	-0.26	-0.94	0.5475	0.8552
**GERM CELL DEVELOPMENT**	175	-0.19	-0.94	0.6173	0.8555
**CYTOSKELETAL ADAPTOR ACTIVITY**	16	0.32	0.87	0.6471	0.8555
**MICROTUBULE POLYMERIZATION OR DEPOLYMERIZATION**	35	0.26	0.87	0.6684	0.8556
**NEGATIVE REGULATION OF GLIOGENESIS**	33	0.27	0.87	0.6794	0.8558
**DENDRITIC SPINE DEVELOPMENT**	19	-0.30	-0.94	0.5231	0.8559
**RESPONSE TO CARBOHYDRATE**	155	-0.20	-0.94	0.6494	0.8559
**CHROMOSOME CONDENSATION**	28	-0.28	-0.94	0.5446	0.8560
**PHOSPHATIDYLSERINE BINDING**	31	-0.27	-0.94	0.5436	0.8561
**POSITIVE REGULATION OF TRANSFORMING GROWTH FACTOR BETA PRODUCTION**	15	-0.33	-0.94	0.5432	0.8565
**BONE GROWTH**	19	-0.31	-0.94	0.5619	0.8566
**RETINA HOMEOSTASIS**	52	-0.24	-0.94	0.5380	0.8567
**REGULATION OF METAL ION TRANSPORT**	296	-0.18	-0.94	0.6968	0.8568
**RESPONSE TO MECHANICAL STIMULUS**	190	-0.19	-0.94	0.6627	0.8569
**REGULATION OF LEUKOCYTE DEGRANULATION**	35	-0.26	-0.94	0.5620	0.8572
**PHOTOPERIODISM**	22	-0.30	-0.94	0.5624	0.8572
**REGULATION OF ENDOTHELIAL CELL APOPTOTIC PROCESS**	38	-0.25	-0.94	0.5783	0.8572
**POSITIVE REGULATION OF LEUKOCYTE APOPTOTIC PROCESS**	23	-0.29	-0.94	0.5314	0.8574
**REGULATION OF PEPTIDYL SERINE PHOSPHORYLATION**	104	0.21	0.87	0.7411	0.8576
**REGULATION OF PEPTIDE TRANSPORT**	228	-0.19	-0.93	0.6773	0.8577

**PROSTAGLANDIN BIOSYNTHETIC PROCESS**	17	-0.31	-0.93	0.5553	0.8577
**SIALYLATION**	18	-0.32	-0.93	0.5797	0.8578
**POSITIVE REGULATION OF SMOOTH MUSCLE CONTRACTION**	30	0.27	0.87	0.6527	0.8578
**ATP DEPENDENT CHROMATIN REMODELING**	53	0.24	0.87	0.6915	0.8578
**CALCIUM ION REGULATED EXOCYTOSIS**	72	-0.22	-0.94	0.5954	0.8578
**PHOSPHOLIPASE C ACTIVATING G PROTEIN COUPLED RECEPTOR SIGNALING PATHWAY**	75	-0.22	-0.93	0.5884	0.8578
**REGULATION OF DNA RECOMBINATION**	52	-0.24	-0.94	0.6021	0.8579
**SYNAPTIC TRANSMISSION CHOLINERGIC**	32	-0.27	-0.93	0.5729	0.8579
**DETECTION OF MECHANICAL STIMULUS**	38	-0.25	-0.93	0.5711	0.8579
**POSITIVE REGULATION OF MYELOID CELL DIFFERENTIATION**	72	-0.22	-0.94	0.5994	0.8580
**MORPHOGENESIS OF EMBRYONIC EPITHELIUM**	123	-0.20	-0.93	0.5926	0.8581
**DETECTION OF VISIBLE LIGHT**	40	-0.25	-0.93	0.5308	0.8581
**ACETYLCHOLINE RECEPTOR ACTIVITY**	26	-0.27	-0.93	0.5714	0.8581
**POSITIVE REGULATION OF CARTILAGE DEVELOPMENT**	28	-0.27	-0.93	0.5787	0.8582
**POSITIVE REGULATION OF NUCLEOTIDE METABOLIC PROCESS**	121	-0.20	-0.93	0.6689	0.8582
**REGULATION OF BEHAVIOR**	62	-0.23	-0.93	0.6169	0.8582
**REGULATION OF GRANULOCYTE DIFFERENTIATION**	16	-0.32	-0.94	0.5553	0.8583
**POSITIVE REGULATION OF CHROMOSOME ORGANIZATION**	129	-0.20	-0.93	0.6776	0.8583
**REGULATION OF BODY FLUID LEVELS**	445	-0.17	-0.94	0.7647	0.8583
**EXTRACELLULAR LIGAND GATED ION CHANNEL ACTIVITY**	69	-0.22	-0.93	0.5802	0.8585
**SERINE HYDROLASE ACTIVITY**	182	-0.19	-0.93	0.6638	0.8585
**SUBSTRATE DEPENDENT CELL MIGRATION**	24	0.29	0.87	0.6582	0.8585
**FEMALE MEIOTIC DIVISION**	21	0.30	0.87	0.6615	0.8585
**RESPONSE TO AUDITORY STIMULUS**	22	0.29	0.87	0.6427	0.8585
**RESPONSE TO MOLECULE OF BACTERIAL ORIGIN**	286	-0.18	-0.93	0.7413	0.8586
**CELLULAR RESPONSE TO PEPTIDE**	246	0.19	0.87	0.7915	0.8588
**REGULATION OF TYROSINE PHOSPHORYLATION OF STAT3 PROTEIN**	41	-0.26	-0.93	0.5539	0.8588
**CELL BODY**	456	0.18	0.87	0.8495	0.8588

**CELL ADHESION MOLECULE BINDING**	172	-0.19	-0.93	0.6846	0.8589
**AORTA MORPHOGENESIS**	21	0.29	0.86	0.6603	0.8590
**NEGATIVE REGULATION OF CELL CYCLE ARREST**	16	-0.32	-0.93	0.5357	0.8590
**NON MEMBRANE SPANNING PROTEIN TYROSINE KINASE ACTIVITY**	38	-0.25	-0.93	0.5787	0.8590
**REGULATION OF EARLY ENDOSOME TO LATE ENDOSOME TRANSPORT**	15	0.32	0.87	0.6479	0.8591
**GLIAL CELL DEVELOPMENT**	68	0.23	0.86	0.6863	0.8591
**EMBRYONIC CAMERA TYPE EYE MORPHOGENESIS**	23	0.29	0.87	0.6356	0.8591
**POSITIVE REGULATION OF ACTIVATED T CELL PROLIFERATION**	22	-0.30	-0.93	0.5660	0.8591
**HEMATOPOIETIC PROGENITOR CELL DIFFERENTIATION**	85	0.22	0.87	0.7289	0.8591
**CELLULAR RESPONSE TO EPIDERMAL GROWTH FACTOR STIMULUS**	23	0.29	0.87	0.6761	0.8591
**REGULATION OF LEUKOCYTE MEDIATED CYTOTOXICITY**	45	0.25	0.87	0.6700	0.8591
**PROSTANOID BIOSYNTHETIC PROCESS**	17	-0.31	-0.93	0.5439	0.8592
**PEPTIDYL TYROSINE AUTOPHOSPHORYLATION**	34	-0.26	-0.93	0.5514	0.8592
**POSITIVE REGULATION OF MULTICELLULAR ORGANISM GROWTH**	27	0.27	0.86	0.6640	0.8593
**MALE GENITALIA DEVELOPMENT**	20	-0.30	-0.93	0.5298	0.8593
**CELL CELL ADHERENS JUNCTION**	50	0.24	0.87	0.6759	0.8593
**ACETYLCHOLINE GATED CHANNEL COMPLEX**	19	0.30	0.87	0.6353	0.8594
**REGULATION OF PROTEIN ACETYLATION**	54	-0.24	-0.93	0.5982	0.8595
**POSITIVE REGULATION OF AXONOGENESIS**	64	0.23	0.87	0.6916	0.8596
**DETECTION OF ABIOTIC STIMULUS**	107	-0.20	-0.93	0.6530	0.8597
**REGULATION OF RUFFLE ASSEMBLY**	16	-0.32	-0.93	0.5759	0.8597
**MIDBRAIN DEVELOPMENT**	81	0.22	0.86	0.7058	0.8598
**REGULATION OF ENDOPLASMIC RETICULUM UNFOLDED PROTEIN RESPONSE**	25	-0.28	-0.94	0.5526	0.8599
**REGULATION OF HISTONE H3 K4 METHYLATION**	24	0.29	0.86	0.6667	0.8600
**POSITIVE REGULATION OF NUCLEOTIDE CATABOLIC PROCESS**	16	0.32	0.86	0.6250	0.8600
**ORGAN OR TISSUE SPECIFIC IMMUNE RESPONSE**	21	-0.29	-0.93	0.5498	0.8604

**POSITIVE REGULATION OF CELL CYCLE ARREST**	73	0.23	0.86	0.7224	0.8606
**REGULATION OF EPIDERMIS DEVELOPMENT**	57	-0.23	-0.92	0.6145	0.8607
**LAMELLIPODIUM ASSEMBLY**	29	0.27	0.86	0.6729	0.8609
**ENDOCYTOSIS**	429	0.18	0.86	0.8495	0.8610
**GANGLIOSIDE BIOSYNTHETIC PROCESS**	15	-0.32	-0.93	0.5481	0.8611
**RESPONSE TO CAMP**	96	-0.21	-0.93	0.6453	0.8611
**ADRENERGIC RECEPTOR BINDING**	19	0.31	0.86	0.6667	0.8612
**BLOOD VESSEL MORPHOGENESIS**	338	0.19	0.86	0.8225	0.8612
**GLUTAMATE RECEPTOR ACTIVITY**	26	-0.28	-0.93	0.5870	0.8612
**NEGATIVE REGULATION OF FAT CELL DIFFERENTIATION**	39	-0.25	-0.93	0.5772	0.8614
**HEART MORPHOGENESIS**	201	-0.18	-0.92	0.7169	0.8615
**WOUND HEALING**	413	-0.17	-0.93	0.8239	0.8616
**OVULATION**	16	-0.32	-0.93	0.5561	0.8617
**REGULATION OF CARDIAC MUSCLE CELL ACTION POTENTIAL**	19	0.30	0.86	0.6593	0.8617
**POSITIVE REGULATION OF SMOOTH MUSCLE CELL PROLIFERATION**	54	0.24	0.86	0.6972	0.8620
**NEGATIVE REGULATION OF TRANSPORTER ACTIVITY**	62	0.23	0.86	0.6948	0.8624
**CELLULAR RESPONSE TO DRUG**	62	0.23	0.86	0.7190	0.8624
**DORSAL VENTRAL AXIS SPECIFICATION**	18	0.30	0.86	0.6172	0.8626
**NEGATIVE REGULATION OF CALCIUM ION IMPORT**	21	0.30	0.86	0.6615	0.8626
**ANGIOGENESIS**	273	0.19	0.86	0.8239	0.8627
**NEGATIVE REGULATION OF CELLULAR PROTEIN LOCALIZATION**	116	0.21	0.86	0.7717	0.8635
**REGULATION OF GLIAL CELL DIFFERENTIATION**	55	0.24	0.86	0.7143	0.8635
**REGULATION OF TISSUE REMODELING**	59	0.23	0.86	0.7132	0.8639
**NEURAL CREST CELL DIFFERENTIATION**	73	0.23	0.86	0.7034	0.8641
**FIBROBLAST GROWTH FACTOR RECEPTOR BINDING**	26	0.27	0.86	0.6752	0.8642
**NEGATIVE REGULATION OF OXIDOREDUCTASE ACTIVITY**	22	0.29	0.86	0.6855	0.8642
**RESPONSE TO GROWTH FACTOR**	443	0.18	0.86	0.8546	0.8643
**VIRAL GENOME REPLICATION**	17	0.31	0.86	0.6373	0.8644
**NEGATIVE REGULATION OF STEM CELL DIFFERENTIATION**	41	-0.26	-0.92	0.5698	0.8645
**RESPONSE TO ZINC ION**	40	0.25	0.86	0.6937	0.8650

**EMBRYONIC HEART TUBE MORPHOGENESIS**	58	0.23	0.86	0.6953	0.8650
**HYPOTHALAMUS DEVELOPMENT**	24	0.28	0.86	0.6807	0.8653
**SERINE TYPE ENDOPEPTIDASE INHIBITOR ACTIVITY**	75	0.22	0.86	0.7353	0.8653
**NEUROEPITHELIAL CELL DIFFERENTIATION**	58	0.23	0.86	0.7228	0.8658
**SENSORY PERCEPTION OF TEMPERATURE STIMULUS**	20	0.29	0.86	0.6903	0.8662
**NEGATIVE REGULATION OF SMOOTH MUSCLE CELL PROLIFERATION**	33	0.27	0.86	0.6571	0.8663
**MICROVILLUS MEMBRANE**	16	0.31	0.86	0.6705	0.8665
**T CELL HOMEOSTASIS**	29	0.27	0.86	0.6922	0.8668
**RESPONSE TO AMPHETAMINE**	27	0.27	0.86	0.6759	0.8669
**POSITIVE REGULATION OF INTERLEUKIN 6 SECRETION**	15	-0.31	-0.92	0.5753	0.8672
**NEGATIVE REGULATION OF NEUROLOGICAL SYSTEM PROCESS**	15	-0.32	-0.92	0.5386	0.8679
**POST EMBRYONIC DEVELOPMENT**	79	0.22	0.85	0.7259	0.8683
**POSITIVE REGULATION OF ERYTHROCYTE DIFFERENTIATION**	21	0.29	0.85	0.6509	0.8684
**ENDOPLASMIC RETICULUM LUMEN**	179	0.20	0.85	0.8016	0.8686
**TUBE FORMATION**	122	0.21	0.85	0.7621	0.8688
**CARDIAC MYOFIBRIL ASSEMBLY**	16	0.31	0.85	0.6402	0.8689
**EXCRETION**	40	0.25	0.85	0.6855	0.8694
**ENTRAINMENT OF CIRCADIAN CLOCK**	23	0.29	0.85	0.6650	0.8697
**TRANSFERASE ACTIVITY TRANSFERRING PENTOSYL GROUPS**	43	0.25	0.85	0.7105	0.8698
**CELLULAR RESPONSE TO CAMP**	47	0.24	0.85	0.6878	0.8699
**RESPONSE TO PEPTIDE**	368	0.18	0.85	0.9038	0.8700
**CHONDROITIN SULFATE PROTEOGLYCAN BIOSYNTHETIC PROCESS**	28	-0.27	-0.92	0.5659	0.8701
**REGULATION OF ANOIKIS**	22	0.29	0.85	0.6549	0.8703
**MULTICELLULAR ORGANISM METABOLIC PROCESS**	84	0.22	0.85	0.7400	0.8704
**PHOSPHATIDYLINOSITOL 3 KINASE ACTIVITY**	67	0.23	0.85	0.7469	0.8704
**REGULATION OF STEROID BIOSYNTHETIC PROCESS**	45	-0.24	-0.92	0.6228	0.8706
**REGULATION OF RENAL SODIUM EXCRETION**	20	-0.30	-0.92	0.5700	0.8708
**REGULATION OF NON CANONICAL WNT SIGNALING PATHWAY**	19	-0.30	-0.92	0.5585	0.8708

**REGULATION OF NUCLEOTIDE METABOLIC PROCESS**	193	-0.19	-0.92	0.7450	0.8713
**VITAMIN BINDING**	70	-0.22	-0.92	0.6543	0.8715
**NEURON DEATH**	37	-0.25	-0.92	0.5960	0.8716
**DIVALENT INORGANIC CATION TRANSPORT**	244	-0.18	-0.92	0.6820	0.8716
**POSITIVE REGULATION OF ENDOCYTOSIS**	101	-0.21	-0.92	0.6585	0.8716
**ACETYLCHOLINE BINDING**	20	0.29	0.85	0.6602	0.8718
**CATION TRANSPORTING ATPASE COMPLEX**	16	-0.32	-0.92	0.5674	0.8719
**EXCITATORY EXTRACELLULAR LIGAND GATED ION CHANNEL ACTIVITY**	50	-0.24	-0.92	0.5944	0.8721
**ADHERENS JUNCTION ORGANIZATION**	66	0.23	0.85	0.7365	0.8721
**REGULATION OF VIRAL GENOME REPLICATION**	58	0.23	0.85	0.7296	0.8722
**REGULATION OF BLOOD CIRCULATION**	277	0.19	0.85	0.8497	0.8722
**PROTEIN TYROSINE KINASE ACTIVITY**	161	0.20	0.85	0.7926	0.8723
**PROTEIN COMPLEX SCAFFOLD**	55	-0.23	-0.92	0.6274	0.8725
**REGULATION OF DELAYED RECTIFIER POTASSIUM CHANNEL ACTIVITY**	18	-0.31	-0.92	0.5357	0.8727
**LAMELLIPODIUM MEMBRANE**	18	-0.30	-0.92	0.5953	0.8728
**DEFENSE RESPONSE TO GRAM POSITIVE BACTERIUM**	50	0.24	0.85	0.7299	0.8730
**NEGATIVE REGULATION OF CELLULAR RESPONSE TO GROWTH FACTOR STIMULUS**	107	0.21	0.85	0.7699	0.8732
**PEPTIDYL ASPARAGINE MODIFICATION**	35	0.26	0.85	0.6883	0.8732
**HORMONE BIOSYNTHETIC PROCESS**	42	0.25	0.85	0.7056	0.8733
**CELLULAR EXTRAVASATION**	23	-0.29	-0.91	0.5952	0.8754
**AUDITORY RECEPTOR CELL DEVELOPMENT**	16	-0.31	-0.91	0.5660	0.8757
**RESPONSE TO LITHIUM ION**	26	0.27	0.85	0.6877	0.8758
**POSITIVE REGULATION OF PURINE NUCLEOTIDE METABOLIC PROCESS**	121	-0.20	-0.91	0.6523	0.8760
**HYDROGEN PEROXIDE METABOLIC PROCESS**	24	0.28	0.85	0.7053	0.8760
**REGULATION OF MAST CELL ACTIVATION**	33	-0.26	-0.91	0.6263	0.8764
**EPITHELIAL CELL MORPHOGENESIS**	40	-0.25	-0.91	0.6178	0.8765
**POSITIVE REGULATION OF MUSCLE CELL DIFFERENTIATION**	78	0.22	0.85	0.7604	0.8765
**SPINDLE MIDZONE**	24	0.28	0.85	0.6952	0.8773

**CARDIAC CHAMBER MORPHOGENESIS**	99	-0.21	-0.91	0.6625	0.8774
**SINGLE FERTILIZATION**	92	0.21	0.85	0.7655	0.8781
**REGULATION OF TRANSCRIPTION FROM RNA POLYMERASE II PROMOTER IN RESPONSE TO HYPOXIA**	29	0.27	0.84	0.7116	0.8781
**VOLTAGE GATED CALCIUM CHANNEL COMPLEX**	36	0.26	0.84	0.7089	0.8782
**NEGATIVE REGULATION OF ENDOCYTOSIS**	36	0.25	0.84	0.6991	0.8790
**PROTEIN ADP RIBOSYLATION**	15	0.31	0.84	0.6583	0.8792
**REGULATION OF CIRCADIAN SLEEP WAKE CYCLE**	24	0.28	0.84	0.7029	0.8793
**FIBRIL ORGANIZATION**	18	0.30	0.84	0.6876	0.8794
**MYOTUBE CELL DEVELOPMENT**	24	0.28	0.84	0.6776	0.8796
**REGULATION OF CELLULAR RESPONSE TO TRANSFORMING GROWTH FACTOR BETA STIMULUS**	85	0.21	0.84	0.7799	0.8796
**PHOTORECEPTOR CELL DEVELOPMENT**	35	0.26	0.84	0.7114	0.8796
**LYSOSOME ORGANIZATION**	42	0.24	0.84	0.6861	0.8797
**NEGATIVE REGULATION OF CHEMOTAXIS**	48	0.24	0.84	0.7200	0.8798
**HISTONE METHYLTRANSFERASE ACTIVITY H3 K4 SPECIFIC**	16	0.30	0.84	0.6774	0.8799
**POSITIVE REGULATION OF TELOMERASE ACTIVITY**	26	0.28	0.84	0.7031	0.8800
**MULTICELLULAR ORGANISMAL WATER HOMEOSTASIS**	45	0.25	0.84	0.7348	0.8801
**RAB GTPASE BINDING**	111	0.21	0.84	0.7703	0.8802
**CELLULAR RESPONSE TO GONADOTROPIN STIMULUS**	15	0.31	0.84	0.6865	0.8804
**NEGATIVE REGULATION OF TELOMERE MAINTENANCE VIA TELOMERE LENGTHENING**	15	0.31	0.84	0.6667	0.8804
**NONRIBOSOMAL PEPTIDE BIOSYNTHETIC PROCESS**	15	0.31	0.84	0.6753	0.8814
**VASCULOGENESIS**	53	-0.23	-0.91	0.6519	0.8815
**OXYGEN BINDING**	20	-0.30	-0.91	0.5687	0.8818
**ENDOTHELIAL CELL PROLIFERATION**	22	0.28	0.84	0.6848	0.8818
**EPHRIN RECEPTOR SIGNALING PATHWAY**	78	-0.21	-0.91	0.6719	0.8823
**DIVALENT INORGANIC CATION HOMEOSTASIS**	315	0.18	0.84	0.8630	0.8825
**EPITHELIAL TUBE BRANCHING INVOLVED IN LUNG MORPHOGENESIS**	24	-0.28	-0.91	0.5754	0.8828

**CERAMIDE BIOSYNTHETIC PROCESS**	33	0.25	0.84	0.6969	0.8829
**VASCULATURE DEVELOPMENT**	437	0.18	0.84	0.9022	0.8829
**NITRIC OXIDE MEDIATED SIGNAL TRANSDUCTION**	18	0.30	0.84	0.7059	0.8832
**NEGATIVE REGULATION OF GTPASE ACTIVITY**	37	0.26	0.84	0.7013	0.8848
**NEGATIVE REGULATION OF LOCOMOTION**	239	0.19	0.84	0.8727	0.8851
**NEGATIVE REGULATION OF CYSTEINE TYPE ENDOPEPTIDASE ACTIVITY**	77	0.22	0.84	0.7590	0.8851
**REGULATION OF TRANSFORMING GROWTH FACTOR BETA RECEPTOR SIGNALING PATHWAY**	85	0.21	0.84	0.7700	0.8852
**RESPONSE TO PROSTAGLANDIN**	29	0.26	0.84	0.7286	0.8852
**POSITIVE REGULATION OF POTASSIUM ION TRANSMEMBRANE TRANSPORT**	26	0.27	0.84	0.7186	0.8865
**DNA MODIFICATION**	66	-0.22	-0.91	0.6607	0.8868
**DNA SYNTHESIS INVOLVED IN DNA REPAIR**	57	-0.23	-0.91	0.6541	0.8872
**POSITIVE REGULATION OF GLYCOPROTEIN METABOLIC PROCESS**	15	0.31	0.84	0.6636	0.8875
**PIGMENT GRANULE**	93	0.21	0.83	0.7809	0.8880
**CELLULAR RESPONSE TO GAMMA RADIATION**	18	0.30	0.83	0.6805	0.8883
**MIDBODY**	115	0.20	0.83	0.7994	0.8887
**MULTICELLULAR ORGANISMAL HOMEOSTASIS**	225	0.19	0.83	0.8555	0.8894
**CATION CATION ANTIPORTER ACTIVITY**	23	0.28	0.83	0.7110	0.8895
**ADENYLATE CYCLASE ACTIVATING G PROTEIN COUPLED RECEPTOR SIGNALING PATHWAY**	65	0.22	0.83	0.7835	0.8896
**BICARBONATE TRANSPORT**	26	0.27	0.83	0.7109	0.8896
**PROTEIN O LINKED GLYCOSYLATION**	82	-0.21	-0.91	0.6687	0.8897
**KIDNEY MORPHOGENESIS**	78	0.22	0.83	0.7564	0.8898
**EXTRINSIC COMPONENT OF MEMBRANE**	219	0.19	0.83	0.8514	0.8899
**EMBRYONIC SKELETAL SYSTEM MORPHOGENESIS**	85	-0.20	-0.90	0.6667	0.8902
**POSITIVE REGULATION OF TRANSCRIPTION FROM RNA POLYMERASE II PROMOTER IN RESPONSE TO STRESS**	20	-0.29	-0.91	0.5658	0.8903
**RESPONSE TO WOUNDING**	495	0.17	0.83	0.9394	0.8906
**CARDIAC CELL DEVELOPMENT**	47	0.24	0.83	0.7483	0.8909

**WATER HOMEOSTASIS**	55	0.23	0.83	0.7539	0.8913
**RETINA LAYER FORMATION**	20	-0.29	-0.90	0.5586	0.8913
**GAMETE GENERATION**	468	0.17	0.83	0.9244	0.8924
**MEMBRANE LIPID CATABOLIC PROCESS**	22	0.28	0.83	0.6782	0.8924
**METENCEPHALON DEVELOPMENT**	94	0.21	0.83	0.7815	0.8925
**REGULATION OF FATTY ACID TRANSPORT**	24	0.28	0.83	0.6979	0.8930
**MUSCLE SYSTEM PROCESS**	267	0.18	0.83	0.8876	0.8933
**DENDRITE**	419	0.17	0.83	0.9263	0.8934
**LYTIC VACUOLE ORGANIZATION**	42	0.24	0.83	0.7330	0.8936
**NEUROTRANSMITTER BINDING**	25	-0.27	-0.90	0.6020	0.8942
**UROGENITAL SYSTEM DEVELOPMENT**	272	0.18	0.83	0.8745	0.8950
**AORTA DEVELOPMENT**	39	0.25	0.83	0.7164	0.8951
**SIGNALING ADAPTOR ACTIVITY**	68	0.22	0.83	0.7684	0.8970
**REGULATION OF EPIDERMAL CELL DIFFERENTIATION**	40	-0.25	-0.90	0.6267	0.8972
**HOMOPHILIC CELL ADHESION VIA PLASMA MEMBRANE ADHESION MOLECULES**	111	-0.20	-0.90	0.6962	0.8978
**CYTOKINE BINDING**	84	-0.21	-0.90	0.7200	0.8979
**NEGATIVE REGULATION OF INTERFERON GAMMA PRODUCTION**	27	-0.27	-0.90	0.6381	0.8984
**AUDITORY RECEPTOR CELL DIFFERENTIATION**	26	0.27	0.83	0.7188	0.8987
**REGULATION OF TRANSCRIPTION INVOLVED IN G1 S TRANSITION OF MITOTIC CELL CYCLE**	22	0.28	0.82	0.7005	0.8997
**NEGATIVE REGULATION OF PEPTIDASE ACTIVITY**	195	0.19	0.82	0.8619	0.8999
**CELLULAR RESPONSE TO NITROGEN COMPOUND**	461	0.17	0.82	0.9266	0.9002
**RESPIRATORY SYSTEM DEVELOPMENT**	185	0.19	0.82	0.8652	0.9014
**PROTEIN COMPLEX LOCALIZATION**	44	-0.24	-0.90	0.6234	0.9017
**TELENCEPHALON DEVELOPMENT**	214	-0.18	-0.90	0.7764	0.9021
**NEURONAL CELL BODY MEMBRANE**	20	-0.29	-0.90	0.6139	0.9022
**SEX DIFFERENTIATION**	231	-0.18	-0.90	0.7845	0.9024
**ALDO KETO REDUCTASE NADP ACTIVITY**	16	-0.31	-0.90	0.6147	0.9028
**POSITIVE REGULATION OF PROTEIN SERINE THREONINE KINASE ACTIVITY**	260	0.18	0.82	0.8935	0.9028
**CELLULAR RESPONSE TO ALCOHOL**	103	0.21	0.82	0.8133	0.9029
**INORGANIC ANION TRANSMEMBRANE TRANSPORTER ACTIVITY**	114	-0.20	-0.90	0.7252	0.9029
**GTP METABOLIC PROCESS**	18	0.29	0.82	0.7226	0.9033

**MESENCHYMAL CELL DIFFERENTIATION**	129	0.20	0.82	0.8272	0.9040
**REGULATION OF URINE VOLUME**	18	0.29	0.82	0.6767	0.9047
**AMIDE TRANSPORT**	89	-0.20	-0.90	0.7034	0.9051
**LIGAND GATED CHANNEL ACTIVITY**	132	-0.19	-0.89	0.7463	0.9059
**POSITIVE REGULATION OF PROTEIN MATURATION**	15	-0.32	-0.89	0.5845	0.9059
**MEMORY**	91	-0.20	-0.89	0.6909	0.9062
**PHOTORECEPTOR CELL MAINTENANCE**	32	0.25	0.82	0.7685	0.9064
**RECEPTOR COMPLEX**	304	0.18	0.82	0.9102	0.9068
**ENDOCYTIC VESICLE**	212	0.19	0.82	0.8838	0.9068
**SYNTAXIN BINDING**	83	0.21	0.82	0.8219	0.9069
**REGULATION OF MULTICELLULAR ORGANISMAL METABOLIC PROCESS**	32	-0.25	-0.89	0.6168	0.9069
**MALE GAMETE GENERATION**	377	0.17	0.82	0.9249	0.9070
**REGULATION OF VOLTAGE GATED CALCIUM CHANNEL ACTIVITY**	24	0.27	0.82	0.7367	0.9070
**PROTEIN DEACETYLASE ACTIVITY**	40	0.24	0.82	0.7615	0.9071
**REGULATION OF TYPE I INTERFERON PRODUCTION**	100	0.20	0.82	0.8394	0.9073
**POSITIVE REGULATION OF INTERFERON BETA PRODUCTION**	30	0.26	0.82	0.7125	0.9074
**REGULATION OF TRANSPORTER ACTIVITY**	185	0.19	0.82	0.8570	0.9082
**INTEGRIN BINDING**	98	-0.20	-0.89	0.7059	0.9088
**RESPONSE TO CORTICOSTEROID**	164	0.19	0.82	0.8768	0.9089
**VESICLE MEDIATED TRANSPORT BETWEEN ENDOSOMAL COMPARTMENTS**	17	0.30	0.82	0.7133	0.9090
**ANTIMICROBIAL HUMORAL RESPONSE**	26	-0.27	-0.89	0.6073	0.9094
**POSITIVE REGULATION OF CELL CYCLE G2 M PHASE TRANSITION**	15	-0.32	-0.89	0.5962	0.9094
**T CELL DIFFERENTIATION IN THYMUS**	42	-0.23	-0.89	0.6620	0.9097
**NEGATIVE REGULATION OF MYELOID CELL DIFFERENTIATION**	72	-0.21	-0.89	0.7309	0.9098
**CONNEXON COMPLEX**	17	0.30	0.81	0.7158	0.9099
**REGULATION OF ION HOMEOSTASIS**	178	-0.18	-0.89	0.7953	0.9101
**RESPONSE TO FIBROBLAST GROWTH FACTOR**	104	-0.20	-0.89	0.7294	0.9101
**POSITIVE REGULATION OF ION TRANSPORT**	219	-0.18	-0.89	0.7768	0.9102
**PRESYNAPTIC ACTIVE ZONE**	29	0.26	0.81	0.7409	0.9103
**FOREBRAIN NEURON DEVELOPMENT**	32	-0.25	-0.89	0.6235	0.9105
**EATING BEHAVIOR**	30	-0.25	-0.89	0.6044	0.9106

**MICROTUBULE MOTOR ACTIVITY**	53	-0.22	-0.89	0.6747	0.9108
**REGULATION OF NATURAL KILLER CELL MEDIATED IMMUNITY**	26	-0.26	-0.89	0.6145	0.9111
**PLATELET ALPHA GRANULE LUMEN**	52	0.23	0.81	0.7793	0.9115
**NEUROTROPHIN SIGNALING PATHWAY**	22	-0.28	-0.89	0.6310	0.9116
**EMBRYONIC PLACENTA DEVELOPMENT**	82	0.21	0.81	0.8072	0.9118
**REGULATION OF PROTEIN SECRETION**	332	-0.17	-0.88	0.9021	0.9119
**REGULATION OF SUPEROXIDE METABOLIC PROCESS**	20	0.29	0.81	0.7196	0.9122
**CARDIAC MUSCLE TISSUE MORPHOGENESIS**	52	-0.22	-0.88	0.6783	0.9122
**REGULATION OF LIPASE ACTIVITY**	80	0.21	0.81	0.8169	0.9123
**REGULATION OF ACTION POTENTIAL**	38	0.24	0.81	0.7600	0.9125
**POSITIVE REGULATION OF INSULIN SECRETION**	58	-0.22	-0.88	0.7075	0.9126
**GRANULOCYTE ACTIVATION**	18	-0.30	-0.88	0.6197	0.9128
**POSITIVE REGULATION OF VASODILATION**	31	0.26	0.81	0.7446	0.9128
**REGULATION OF HEMATOPOIETIC PROGENITOR CELL DIFFERENTIATION**	29	0.25	0.81	0.7554	0.9128
**GLUTAMATE SECRETION**	28	0.26	0.81	0.7471	0.9128
**TRANSFERASE ACTIVITY TRANSFERRING AMINO ACYL GROUPS**	18	-0.29	-0.88	0.6222	0.9129
**APICOLATERAL PLASMA MEMBRANE**	15	-0.31	-0.89	0.6227	0.9130
**REGULATION OF PROTEIN OLIGOMERIZATION**	29	0.25	0.81	0.7443	0.9130
**HINDBRAIN DEVELOPMENT**	127	0.20	0.81	0.8484	0.9130
**POSITIVE REGULATION OF CALCIUM ION TRANSMEMBRANE TRANSPORT**	55	-0.22	-0.88	0.7033	0.9130
**MESENCHYME DEVELOPMENT**	176	-0.18	-0.88	0.8312	0.9131
**REGULATION OF NITRIC OXIDE SYNTHASE ACTIVITY**	44	-0.24	-0.89	0.6585	0.9132
**MAMMARY GLAND DUCT MORPHOGENESIS**	28	-0.25	-0.88	0.6441	0.9132
**VENTRICULAR CARDIAC MUSCLE CELL DIFFERENTIATION**	18	-0.30	-0.89	0.6194	0.9133
**ASTROCYTE DEVELOPMENT**	17	-0.30	-0.88	0.5911	0.9133
**REGULATION OF CELL PROJECTION ASSEMBLY**	133	0.19	0.81	0.8577	0.9133
**CILIARY PART**	236	-0.18	-0.88	0.8465	0.9134
**RESPONSE TO VITAMIN A**	19	-0.29	-0.88	0.6441	0.9135
**HMG BOX DOMAIN BINDING**	17	-0.30	-0.88	0.6241	0.9136
**LEUKOTRIENE METABOLIC PROCESS**	21	-0.28	-0.88	0.6237	0.9136

**RESPONSE TO AXON INJURY**	42	-0.23	-0.88	0.6821	0.9136
**MONOVALENT INORGANIC CATION TRANSMEMBRANE TRANSPORTER ACTIVITY**	333	0.17	0.81	0.9322	0.9136
**CEREBELLAR CORTEX DEVELOPMENT**	44	-0.23	-0.88	0.6712	0.9137
**REGULATION OF G PROTEIN COUPLED RECEPTOR PROTEIN SIGNALING PATHWAY**	117	-0.20	-0.89	0.7647	0.9138
**VISUAL BEHAVIOR**	47	-0.23	-0.88	0.6898	0.9138
**CIRCULATORY SYSTEM PROCESS**	338	-0.17	-0.88	0.8865	0.9140
**NEGATIVE REGULATION OF VIRAL GENOME REPLICATION**	36	-0.24	-0.88	0.6731	0.9140
**REGULATION OF SYNAPTIC TRANSMISSION DOPAMINERGIC**	16	-0.30	-0.88	0.6232	0.9141
**RESPONSE TO ETHANOL**	123	-0.19	-0.88	0.7645	0.9142
**VENTRAL SPINAL CORD INTERNEURON DIFFERENTIATION**	16	-0.30	-0.88	0.6062	0.9143
**POSITIVE REGULATION OF HISTONE H3 K4 METHYLATION**	15	-0.30	-0.87	0.6315	0.9144
**ATP DEPENDENT MICROTUBULE MOTOR ACTIVITY**	16	-0.30	-0.88	0.6316	0.9144
**NOTCH BINDING**	17	-0.30	-0.88	0.6179	0.9144
**NEGATIVE REGULATION OF CELLULAR RESPONSE TO INSULIN STIMULUS**	29	-0.25	-0.87	0.6877	0.9144
**MUSCLE ADAPTATION**	28	-0.26	-0.89	0.6576	0.9145
**MALE SEX DIFFERENTIATION**	128	-0.19	-0.88	0.7559	0.9146
**NEGATIVE REGULATION OF ENDOTHELIAL CELL PROLIFERATION**	30	-0.26	-0.88	0.6551	0.9147
**REGULATION OF INFLAMMATORY RESPONSE**	257	-0.17	-0.88	0.9031	0.9149
**KERATAN SULFATE BIOSYNTHETIC PROCESS**	24	-0.26	-0.88	0.6372	0.9150
**REGULATION OF CELL SUBSTRATE ADHESION**	157	-0.18	-0.88	0.7760	0.9150
**REGULATION OF OSTEOBLAST DIFFERENTIATION**	103	-0.19	-0.88	0.7287	0.9151
**GLYCOSPHINGOLIPID BIOSYNTHETIC PROCESS**	21	-0.28	-0.88	0.6194	0.9152
**POSITIVE REGULATION OF IMMUNE RESPONSE**	447	0.17	0.81	0.9502	0.9159
**REGULATION OF BONE RESORPTION**	31	0.25	0.81	0.7650	0.9159
**INOSITOL LIPID MEDIATED SIGNALING**	114	-0.19	-0.87	0.7695	0.9161
**CARRECEPTOR ACTIVITY**	58	0.22	0.81	0.7892	0.9162
**POSITIVE CHEMOTAXIS**	31	-0.25	-0.87	0.6569	0.9163

**CELLULAR RESPONSE TO ACID CHEMICAL**	159	-0.18	-0.87	0.8073	0.9166
**CHLORIDE TRANSPORT**	88	-0.20	-0.87	0.7468	0.9167
**THYMOCYTE AGGREGATION**	42	-0.23	-0.87	0.7016	0.9167
**MAMMARY GLAND EPITHELIUM DEVELOPMENT**	51	0.23	0.81	0.7658	0.9168
**BONE DEVELOPMENT**	146	-0.18	-0.87	0.8095	0.9174
**REGULATION OF HEART RATE**	81	0.21	0.81	0.8192	0.9176
**REGULATION OF SYSTEM PROCESS**	477	0.17	0.81	0.9574	0.9177
**ANATOMICAL STRUCTURE HOMEOSTASIS**	232	0.18	0.81	0.8885	0.9177
**GTP DEPENDENT PROTEIN BINDING**	17	-0.29	-0.87	0.6465	0.9178
**GLUTATHIONE DERIVATIVE METABOLIC PROCESS**	16	0.29	0.81	0.6989	0.9178
**NEGATIVE REGULATION OF TUMOR NECROSIS FACTOR SUPERFAMILY CYTOKINE PRODUCTION**	40	-0.24	-0.87	0.6657	0.9179
**PHOSPHATASE BINDING**	138	0.19	0.81	0.8578	0.9181
**REGULATION OF NOREPINEPHRINE SECRETION**	17	-0.29	-0.87	0.6209	0.9184
**APICAL JUNCTION COMPLEX**	117	0.20	0.81	0.8534	0.9188
**DESMOSOME**	24	-0.27	-0.87	0.6908	0.9188
**POSTSYNAPSE**	348	0.17	0.80	0.9504	0.9189
**POSITIVE REGULATION OF ENDOTHELIAL CELL MIGRATION**	65	-0.21	-0.87	0.7423	0.9198
**REGULATION OF MEIOTIC CELL CYCLE**	38	0.24	0.80	0.7546	0.9200
**CELL SUBSTRATE JUNCTION**	365	0.17	0.80	0.9515	0.9202
**PLATELET DENSE GRANULE**	18	0.29	0.80	0.7326	0.9203
**PLASMA MEMBRANE PROTEIN COMPLEX**	455	0.17	0.80	0.9693	0.9204
**REGULATION OF SEQUESTERING OF CALCIUM ION**	93	-0.19	-0.87	0.7626	0.9205
**POSITIVE REGULATION OF TRANSCRIPTION FACTOR IMPORT INTO NUCLEUS**	45	-0.23	-0.87	0.7028	0.9211
**CIRCADIAN REGULATION OF GENE EXPRESSION**	52	-0.22	-0.87	0.7131	0.9212
**POSITIVE REGULATION OF PROTEIN IMPORT**	95	0.20	0.80	0.8356	0.9212
**TRANSFERASE ACTIVITY TRANSFERRING NITROGENOUS GROUPS**	22	0.27	0.80	0.7323	0.9214
**PHOSPHATIDYLINOSITOL 3 PHOSPHATE BIOSYNTHETIC PROCESS**	47	0.23	0.80	0.7925	0.9215
**PROTEIN AUTOPHOSPHORYLATION**	175	0.18	0.80	0.8919	0.9216

**REGULATION OF GLIOGENESIS**	83	0.21	0.80	0.8323	0.9216
**ENDODERM DEVELOPMENT**	67	-0.21	-0.87	0.7568	0.9218
**TISSUE MORPHOGENESIS**	496	-0.16	-0.87	0.9609	0.9225
**MICROVILLUS**	67	0.21	0.80	0.7925	0.9230
**REGULATION OF INTERLEUKIN 17 PRODUCTION**	19	-0.28	-0.87	0.6463	0.9233
**CARBOXYPEPTIDASE ACTIVITY**	38	0.24	0.80	0.7806	0.9235
**POSITIVE REGULATION OF MITOTIC NUCLEAR DIVISION**	44	-0.23	-0.87	0.7157	0.9235
**ERYTHROCYTE HOMEOSTASIS**	60	-0.21	-0.86	0.7808	0.9236
**AMINOGLYCAN METABOLIC PROCESS**	148	0.19	0.80	0.8942	0.9236
**RESPONSE TO ESTROGEN**	198	0.18	0.80	0.9044	0.9236
**SYNAPTIC VESICLE RECYCLING**	23	0.26	0.80	0.7240	0.9237
**NEUROTRANSMITTER SODIUM SYMPORTER ACTIVITY**	17	0.29	0.80	0.7177	0.9238
**POSITIVE REGULATION OF LIPASE ACTIVITY**	63	-0.21	-0.86	0.7213	0.9238
**NEGATIVE REGULATION OF PEPTIDYL TYROSINE PHOSPHORYLATION**	35	0.24	0.80	0.7708	0.9239
**MEIOTIC CHROMOSOME SEGREGATION**	52	-0.22	-0.86	0.7313	0.9240
**REGULATION OF TELOMERE CAPPING**	21	0.27	0.80	0.7333	0.9241
**SH3 DOMAIN BINDING**	106	-0.19	-0.86	0.7508	0.9242
**PLATELET DERIVED GROWTH FACTOR RECEPTOR SIGNALING PATHWAY**	31	0.25	0.80	0.7439	0.9245
**PEPTIDE SECRETION**	55	-0.22	-0.86	0.7003	0.9246
**POSITIVE REGULATION OF CYTOKINE SECRETION**	73	-0.20	-0.86	0.7761	0.9257
**REPRODUCTIVE SYSTEM DEVELOPMENT**	365	-0.16	-0.86	0.9379	0.9257
**MOTILE CILIUM**	86	0.20	0.80	0.8361	0.9263
**POSITIVE REGULATION OF PROTEIN KINASE B SIGNALING**	70	0.21	0.80	0.8293	0.9268
**CELLULAR RESPONSE TO OXYGEN LEVELS**	126	0.19	0.80	0.8754	0.9269
**SULFUR COMPOUND BINDING**	209	0.18	0.79	0.9099	0.9280
**PROSTAGLANDIN METABOLIC PROCESS**	23	-0.27	-0.86	0.6721	0.9282
**CARDIAC MUSCLE TISSUE DEVELOPMENT**	135	0.19	0.79	0.8826	0.9282
**CHRONIC INFLAMMATORY RESPONSE**	15	-0.30	-0.86	0.6531	0.9283
**ACTIVATION OF MAPK ACTIVITY**	124	0.19	0.79	0.8782	0.9285
**RAS PROTEIN SIGNAL TRANSDUCTION**	127	-0.18	-0.86	0.8384	0.9285
**REGULATION OF GASTRULATION**	34	0.24	0.79	0.7827	0.9287
**POSITIVE REGULATION OF PROTEIN SECRETION**	175	-0.17	-0.86	0.8715	0.9288

**ACTIN FILAMENT BINDING**	108	0.19	0.79	0.8874	0.9289
**BICELLULAR TIGHT JUNCTION ASSEMBLY**	28	-0.25	-0.86	0.6822	0.9292
**POSITIVE REGULATION OF OXIDOREDUCTASE ACTIVITY**	42	0.23	0.79	0.7745	0.9292
**POSITIVE REGULATION OF CATION CHANNEL ACTIVITY**	36	0.24	0.79	0.7855	0.9293
**TRANSMEMBRANE RECEPTOR PROTEIN PHOSPHATASE ACTIVITY**	17	-0.29	-0.86	0.6423	0.9294
**CARDIAC CHAMBER DEVELOPMENT**	136	0.19	0.79	0.8715	0.9295
**REGULATION OF INTERLEUKIN 6 PRODUCTION**	92	0.20	0.79	0.8423	0.9296
**POSITIVE REGULATION OF STRIATED MUSCLE CONTRACTION**	15	0.30	0.79	0.7274	0.9313
**NUCLEOSIDE TRANSPORT**	15	0.29	0.79	0.7635	0.9314
**REGULATION OF EXOCYTOSIS**	165	0.18	0.79	0.8984	0.9328
**ACTIN BASED CELL PROJECTION**	164	0.18	0.79	0.9008	0.9330
**MEMBRANE ASSEMBLY**	23	0.26	0.79	0.7453	0.9332
**NEGATIVE REGULATION OF CALCIUM ION TRANSMEMBRANE TRANSPORT**	27	0.25	0.79	0.8034	0.9332
**REGULATION OF MACROPHAGE ACTIVATION**	25	0.26	0.79	0.7483	0.9333
**COPPER ION TRANSPORT**	18	-0.29	-0.86	0.6522	0.9334
**REGULATION OF POTASSIUM ION TRANSPORT**	78	0.21	0.79	0.8453	0.9334
**BENZENE CONTAINING COMPOUND METABOLIC PROCESS**	22	0.27	0.79	0.7750	0.9335
**POTASSIUM ION IMPORT**	28	-0.26	-0.85	0.6502	0.9335
**REGULATION OF HISTONE METHYLATION**	50	0.22	0.79	0.8323	0.9336
**POSITIVE REGULATION OF JUN KINASE ACTIVITY**	57	0.22	0.79	0.8396	0.9336
**REGULATION OF SODIUM ION TRANSMEMBRANE TRANSPORTER ACTIVITY**	34	0.24	0.79	0.7885	0.9337
**RECEPTOR MEDIATED ENDOCYTOSIS**	186	0.18	0.79	0.9223	0.9338
**ACETYLGALACTOSAMINYLTRANSFERASE ACTIVITY**	30	0.25	0.79	0.7781	0.9338
**POSITIVE REGULATION OF PEPTIDE SECRETION**	82	-0.20	-0.85	0.7813	0.9340
**GLUTATHIONE DERIVATIVE BIOSYNTHETIC PROCESS**	16	0.29	0.79	0.7345	0.9341
**NEGATIVE REGULATION OF GLIAL CELL DIFFERENTIATION**	24	-0.26	-0.85	0.6870	0.9342

**PROSTANOID METABOLIC PROCESS**	23	-0.27	-0.86	0.6675	0.9344
**CENTRAL NERVOUS SYSTEM NEURON DEVELOPMENT**	64	-0.21	-0.85	0.7566	0.9345
**PRODUCTION OF MOLECULAR MEDIATOR OF IMMUNE RESPONSE**	48	0.22	0.79	0.8150	0.9348
**REGULATION OF EXTENT OF CELL GROWTH**	94	-0.20	-0.85	0.7876	0.9350
**ANCHORING JUNCTION**	448	-0.16	-0.85	0.9929	0.9352
**CENTROSOME LOCALIZATION**	16	-0.29	-0.85	0.6838	0.9352
**CARDIAC VENTRICLE DEVELOPMENT**	98	0.20	0.78	0.8746	0.9363
**REGULATION OF ANTIGEN RECEPTOR MEDIATED SIGNALING PATHWAY**	38	0.24	0.78	0.8192	0.9364
**POSITIVE REGULATION OF ERBB SIGNALING PATHWAY**	31	0.25	0.78	0.7892	0.9365
**CELL CELL JUNCTION**	352	0.17	0.78	0.9642	0.9365
**NEURONAL STEM CELL POPULATION MAINTENANCE**	19	0.28	0.78	0.7517	0.9365
**SUBSTRATE ADHESION DEPENDENT CELL SPREADING**	33	0.24	0.78	0.8036	0.9366
**NEGATIVE REGULATION OF TOLL LIKE RECEPTOR SIGNALING PATHWAY**	24	0.26	0.78	0.7709	0.9366
**NEUROTRANSMITTER TRANSPORTER ACTIVITY**	23	0.26	0.78	0.7655	0.9367
**BLASTODERM SEGMENTATION**	15	0.29	0.78	0.7436	0.9368
**SMOOTHENED SIGNALING PATHWAY**	65	-0.21	-0.85	0.7578	0.9376
**REGULATION OF ALCOHOL BIOSYNTHETIC PROCESS**	42	-0.23	-0.85	0.7416	0.9379
**O GLYCAN PROCESSING**	48	0.22	0.78	0.8175	0.9384
**POSITIVE REGULATION OF LIPID KINASE ACTIVITY**	28	0.25	0.78	0.7863	0.9390
**ANION CHANNEL ACTIVITY**	78	0.20	0.78	0.8567	0.9391
**PROTEIN HOMOTRIMERIZATION**	19	-0.27	-0.85	0.6611	0.9391
**RAS GUANYL NUCLEOTIDE EXCHANGE FACTOR ACTIVITY**	199	0.18	0.78	0.9387	0.9392
**INORGANIC ANION EXCHANGER ACTIVITY**	17	0.28	0.78	0.7620	0.9393
**REGULATION OF RAS PROTEIN SIGNAL TRANSDUCTION**	156	0.18	0.78	0.9243	0.9393
**KINASE ACTIVATOR ACTIVITY**	55	0.22	0.78	0.8311	0.9393
**SOMATIC STEM CELL POPULATION MAINTENANCE**	63	0.21	0.78	0.8447	0.9394
**ACTIN FILAMENT POLYMERIZATION**	19	0.28	0.78	0.7629	0.9394

**REGULATION OF STEROID HORMONE SECRETION**	18	0.28	0.78	0.7861	0.9394
**MYELOID CELL DIFFERENTIATION**	167	0.18	0.78	0.9271	0.9396
**LUNG EPITHELIUM DEVELOPMENT**	33	0.24	0.78	0.7997	0.9397
**REACTIVE OXYGEN SPECIES BIOSYNTHETIC PROCESS**	21	0.26	0.78	0.7560	0.9397
**KIDNEY EPITHELIUM DEVELOPMENT**	118	0.19	0.78	0.8946	0.9397
**DNA BIOSYNTHETIC PROCESS**	94	0.20	0.78	0.8748	0.9398
**NEGATIVE REGULATION OF NUCLEOSIDE METABOLIC PROCESS**	18	0.27	0.78	0.7739	0.9398
**REGULATION OF GLUCOSE IMPORT**	49	0.22	0.78	0.8270	0.9399
**CELL CELL RECOGNITION**	48	0.22	0.78	0.8281	0.9400
**NEGATIVE REGULATION OF SMALL GTPASE MEDIATED SIGNAL TRANSDUCTION**	34	0.24	0.78	0.7810	0.9401
**EPIDERMAL GROWTH FACTOR RECEPTOR SIGNALING PATHWAY**	53	0.22	0.78	0.8297	0.9401
**REGULATION OF VESICLE FUSION**	56	0.21	0.77	0.8632	0.9410
**NEGATIVE REGULATION OF ATP METABOLIC PROCESS**	18	0.27	0.77	0.7853	0.9410
**NEGATIVE REGULATION OF HISTONE MODIFICATION**	31	0.24	0.77	0.7939	0.9412
**TUBE MORPHOGENESIS**	302	0.17	0.77	0.9791	0.9417
**VASCULAR PROCESS IN CIRCULATORY SYSTEM**	152	-0.18	-0.85	0.8617	0.9418
**CATECHOLAMINE BINDING**	15	0.29	0.77	0.7606	0.9424
**MORPHOGENESIS OF AN ENDOTHELIUM**	15	-0.29	-0.85	0.6587	0.9425
**POSITIVE REGULATION OF HORMONE SECRETION**	107	0.19	0.77	0.8963	0.9426
**POSITIVE REGULATION OF NOTCH SIGNALING PATHWAY**	31	-0.24	-0.85	0.6885	0.9427
**EMBRYONIC DIGESTIVE TRACT MORPHOGENESIS**	16	-0.29	-0.85	0.6544	0.9428
**RESPONSE TO NICOTINE**	47	0.22	0.77	0.8500	0.9428
**BLOOD MICROPARTICLE**	89	0.20	0.77	0.8985	0.9428
**CELL CELL SIGNALING INVOLVED IN CARDIAC CONDUCTION**	21	-0.27	-0.84	0.6925	0.9428
**METALLOENDOPEPTIDASE ACTIVITY**	98	-0.19	-0.85	0.7860	0.9429
**ACTIN POLYMERIZATION OR DEPOLYMERIZATION**	33	-0.24	-0.85	0.7156	0.9429
**CYCLASE ACTIVITY**	21	0.26	0.77	0.7929	0.9430

**POSITIVE REGULATION OF NATURAL KILLER CELL MEDIATED IMMUNITY**	17	-0.28	-0.84	0.7280	0.9432
**REGULATION OF POTASSIUM ION TRANSMEMBRANE TRANSPORTER ACTIVITY**	39	0.23	0.77	0.7844	0.9433
**REGULATION OF HAIR CYCLE**	21	-0.27	-0.85	0.7067	0.9437
**CHEMOREPELLENT ACTIVITY**	27	-0.25	-0.84	0.7077	0.9438
**CILIUM**	373	-0.16	-0.84	0.9657	0.9439
**ACTIN FILAMENT ORGANIZATION**	152	-0.18	-0.84	0.8504	0.9440
**CELLULAR PROCESS INVOLVED IN REPRODUCTION IN MULTICELLULAR ORGANISM**	213	-0.17	-0.84	0.9033	0.9441
**PRODUCTION OF SMALL RNA INVOLVED IN GENE SILENCING BY RNA**	20	-0.27	-0.84	0.7026	0.9443
**DETECTION OF BIOTIC STIMULUS**	17	-0.28	-0.84	0.7197	0.9444
**REGULATION OF MYELOID LEUKOCYTE DIFFERENTIATION**	98	-0.19	-0.84	0.8404	0.9445
**REGULATION OF ADENYLATE CYCLASE ACTIVITY**	65	-0.20	-0.84	0.7867	0.9446
**POSITIVE REGULATION OF MUSCLE CONTRACTION**	44	-0.22	-0.84	0.7628	0.9446
**ASTROCYTE DIFFERENTIATION**	37	-0.23	-0.84	0.7245	0.9446
**POSITIVE REGULATION OF HISTONE METHYLATION**	29	-0.25	-0.84	0.7215	0.9447
**MEIOTIC CELL CYCLE PROCESS**	124	-0.18	-0.84	0.8596	0.9447
**MORPHOGENESIS OF AN EPITHELIUM**	370	-0.16	-0.84	0.9600	0.9448
**DSRNA FRAGMENTATION**	20	-0.27	-0.84	0.6767	0.9452
**NEGATIVE REGULATION OF ION TRANSPORT**	117	0.19	0.77	0.8983	0.9453
**POSITIVE REGULATION OF ADENYLATE CYCLASE ACTIVITY**	44	0.22	0.77	0.8228	0.9453
**DEOXYRIBOSE PHOSPHATE CATABOLIC PROCESS**	18	0.27	0.77	0.7837	0.9454
**LENS MORPHOGENESIS IN CAMERA TYPE EYE**	19	0.27	0.77	0.7792	0.9454
**BRANCHING INVOLVED IN SALIVARY GLAND MORPHOGENESIS**	16	0.28	0.77	0.7787	0.9457
**CALMODULIN DEPENDENT PROTEIN KINASE ACTIVITY**	26	0.25	0.77	0.7892	0.9457
**SENSORY PERCEPTION OF LIGHT STIMULUS**	192	-0.17	-0.84	0.9156	0.9458
**CILIARY PLASM**	53	0.21	0.77	0.8404	0.9464
**RESPONSE TO ALCOHOL**	330	-0.16	-0.84	0.9500	0.9465

**REGULATION OF REPRODUCTIVE PROCESS**	121	0.18	0.77	0.9231	0.9471
**BAF TYPE COMPLEX**	21	-0.27	-0.84	0.6989	0.9476
**STRIATED MUSCLE CONTRACTION**	94	0.19	0.76	0.8688	0.9480
**REGULATION OF POTASSIUM ION TRANSMEMBRANE TRANSPORT**	59	-0.21	-0.84	0.7644	0.9480
**POSITIVE REGULATION OF INTERLEUKIN 12 PRODUCTION**	31	0.24	0.76	0.8283	0.9480
**POSTSYNAPTIC MEMBRANE ORGANIZATION**	25	0.25	0.76	0.7987	0.9481
**RESPONSE TO VITAMIN**	93	-0.19	-0.84	0.8055	0.9484
**MODULATION OF SYNAPTIC TRANSMISSION**	283	0.17	0.76	0.9723	0.9485
**NEGATIVE REGULATION OF HISTONE METHYLATION**	16	-0.28	-0.83	0.6435	0.9491
**SECRETORY GRANULE**	316	-0.16	-0.83	0.9524	0.9493
**RETINA DEVELOPMENT IN CAMERA TYPE EYE**	121	0.18	0.76	0.9130	0.9495
**REGULATION OF RENAL SYSTEM PROCESS**	33	-0.23	-0.83	0.7532	0.9495
**TELOMERE CAPPING**	19	-0.27	-0.83	0.7070	0.9496
**PEPTIDASE INHIBITOR ACTIVITY**	134	-0.18	-0.83	0.9011	0.9499
**DIGESTIVE SYSTEM PROCESS**	57	0.21	0.76	0.8707	0.9499
**PHOSPHATIDYLSERINE ACYL CHAIN REMODELING**	16	0.28	0.76	0.7784	0.9507
**MEIOTIC CELL CYCLE**	152	0.18	0.76	0.9426	0.9508
**REGULATION OF STEM CELL DIFFERENTIATION**	104	0.19	0.76	0.9170	0.9508
**NEGATIVE REGULATION OF ACTIN FILAMENT BUNDLE ASSEMBLY**	18	0.27	0.76	0.8094	0.9512
**SENSORY PERCEPTION OF PAIN**	72	0.20	0.76	0.8649	0.9518
**EMBRYONIC HEART TUBE DEVELOPMENT**	69	0.20	0.76	0.8794	0.9521
**REGULATION OF JUN KINASE ACTIVITY**	72	0.20	0.76	0.8849	0.9523
**REGULATION OF EXECUTION PHASE OF APOPTOSIS**	20	0.26	0.76	0.8055	0.9526
**AXIS ELONGATION**	27	-0.24	-0.83	0.7203	0.9527
**EPHRIN RECEPTOR BINDING**	24	-0.25	-0.83	0.7147	0.9531
**PEPTIDE HORMONE BINDING**	35	-0.23	-0.83	0.7352	0.9534
**REGULATION OF CALCIUM ION DEPENDENT EXOCYTOSIS**	76	-0.20	-0.83	0.8233	0.9537
**REGULATION OF LAMELLIPODIUM ASSEMBLY**	23	0.25	0.76	0.8112	0.9537
**SALIVARY GLAND DEVELOPMENT**	31	0.24	0.76	0.8137	0.9538

**REGULATION OF RHO PROTEIN SIGNAL TRANSDUCTION**	92	-0.19	-0.83	0.8125	0.9539
**NEGATIVE REGULATION OF EPITHELIAL TO MESENCHYMAL TRANSITION**	22	-0.26	-0.83	0.7120	0.9541
**ENZYME ACTIVATOR ACTIVITY**	413	0.16	0.76	0.9905	0.9541
**CELLULAR RESPONSE TO MECHANICAL STIMULUS**	73	0.20	0.76	0.8795	0.9541
**REGULATION OF ACTIN FILAMENT DEPOLYMERIZATION**	37	-0.23	-0.83	0.7519	0.9543
**SMOOTH ENDOPLASMIC RETICULUM**	32	-0.24	-0.83	0.7476	0.9546
**CATECHOLAMINE METABOLIC PROCESS**	34	0.23	0.75	0.8574	0.9548
**REGULATION OF CELLULAR RESPONSE TO GROWTH FACTOR STIMULUS**	206	0.17	0.75	0.9482	0.9550
**ESTABLISHMENT OR MAINTENANCE OF APICAL BASAL CELL POLARITY**	31	0.24	0.75	0.8342	0.9551
**POSITIVE REGULATION OF REGULATED SECRETORY PATHWAY**	44	0.22	0.75	0.8616	0.9551
**CATECHOL CONTAINING COMPOUND METABOLIC PROCESS**	34	0.23	0.75	0.8459	0.9551
**RESPONSE TO GROWTH HORMONE**	24	-0.25	-0.83	0.6923	0.9553
**REGULATION OF OXIDOREDUCTASE ACTIVITY**	81	0.19	0.75	0.8918	0.9554
**CELL RECOGNITION**	110	0.18	0.75	0.9201	0.9554
**MUSCLE CONTRACTION**	220	0.17	0.75	0.9613	0.9556
**REGULATION OF COLLATERAL SPROUTING**	16	0.28	0.75	0.8179	0.9558
**NEGATIVE REGULATION OF CHROMATIN MODIFICATION**	40	0.22	0.75	0.8539	0.9558
**T CELL RECEPTOR COMPLEX**	17	-0.28	-0.83	0.6930	0.9559
**NOTCH SIGNALING PATHWAY**	103	0.19	0.75	0.9299	0.9560
**NEGATIVE REGULATION OF MUSCLE ORGAN DEVELOPMENT**	31	0.24	0.75	0.8341	0.9560
**ALDEHYDE DEHYDROGENASE NAD ACTIVITY**	18	0.27	0.75	0.8225	0.9562
**AMINO ACID BINDING**	95	0.19	0.75	0.9196	0.9563
**POSITIVE REGULATION OF MESENCHYMAL CELL PROLIFERATION**	23	0.25	0.75	0.8166	0.9564
**MODIFIED AMINO ACID BINDING**	57	0.20	0.75	0.8662	0.9565
**REGULATION OF BONE DEVELOPMENT**	15	-0.28	-0.82	0.6843	0.9567
**PHAGOCYTOSIS**	142	0.18	0.75	0.9451	0.9567
**POSITIVE REGULATION OF TISSUE REMODELING**	24	0.24	0.75	0.8429	0.9569

**POSITIVE REGULATION OF MAP KINASE ACTIVITY**	188	-0.17	-0.82	0.9109	0.9571
**ESTABLISHMENT OR MAINTENANCE OF BIPOLAR CELL POLARITY**	31	0.24	0.75	0.8163	0.9572
**CELL PROJECTION ASSEMBLY**	215	0.17	0.75	0.9688	0.9573
**CAMP MEDIATED SIGNALING**	36	0.23	0.75	0.8451	0.9573
**PROTEIN TYROSINE SERINE THREONINE PHOSPHATASE ACTIVITY**	43	0.22	0.75	0.8505	0.9575
**MYELOID DENDRITIC CELL DIFFERENTIATION**	18	-0.28	-0.82	0.7072	0.9576
**POSITIVE REGULATION OF EXTRINSIC APOPTOTIC SIGNALING PATHWAY VIA DEATH DOMAIN RECEPTORS**	15	0.28	0.74	0.8139	0.9576
**REGULATION OF LIPOPOLYSACCHARIDE MEDIATED SIGNALING PATHWAY**	16	0.27	0.74	0.7784	0.9578
**PLASMA MEMBRANE FUSION**	22	-0.26	-0.82	0.7543	0.9579
**CYCLIC NUCLEOTIDE BINDING**	35	0.23	0.74	0.8542	0.9579
**POST ANAL TAIL MORPHOGENESIS**	17	-0.27	-0.82	0.7116	0.9579
**DNA REPLICATION INITIATION**	21	0.25	0.75	0.8295	0.9580
**ARACHIDONIC ACID METABOLIC PROCESS**	31	0.23	0.75	0.8586	0.9581
**SECONDARY METABOLIC PROCESS**	33	0.23	0.75	0.8515	0.9581
**DNA DIRECTED DNA POLYMERASE ACTIVITY**	25	-0.25	-0.82	0.7401	0.9584
**REGULATION OF MONOOXYGENASE ACTIVITY**	55	-0.21	-0.82	0.7698	0.9585
**CELLULAR RESPONSE TO AMINO ACID STIMULUS**	47	-0.21	-0.82	0.7943	0.9586
**PEPTIDE HORMONE PROCESSING**	29	-0.24	-0.82	0.7375	0.9587
**POSITIVE REGULATION OF PROTEIN AUTOPHOSPHORYLATION**	20	-0.26	-0.82	0.7188	0.9590
**AMINOGLYCAN BIOSYNTHETIC PROCESS**	97	0.18	0.74	0.9099	0.9591
**BONE MINERALIZATION**	35	-0.23	-0.82	0.7354	0.9591
**BRANCHING INVOLVED IN MAMMARY GLAND DUCT MORPHOGENESIS**	20	0.25	0.74	0.8151	0.9593
**PATTERNING OF BLOOD VESSELS**	30	-0.24	-0.82	0.7364	0.9593
**BRANCHING MORPHOGENESIS OF AN EPITHELIAL TUBE**	124	0.18	0.74	0.9402	0.9595
**GLUTAMINE FAMILY AMINO ACID BIOSYNTHETIC PROCESS**	17	-0.28	-0.82	0.7342	0.9595
**ION CHANNEL BINDING**	104	0.18	0.74	0.9325	0.9597

**REGULATION OF INTRACELLULAR STEROID HORMONE RECEPTOR SIGNALING PATHWAY**	52	-0.21	-0.82	0.7920	0.9599
**LENS DEVELOPMENT IN CAMERA TYPE EYE**	61	0.20	0.74	0.8937	0.9605
**REGULATION OF FILOPODIUM ASSEMBLY**	33	-0.23	-0.82	0.7592	0.9605
**ENDODERMAL CELL DIFFERENTIATION**	38	0.22	0.74	0.8646	0.9606
**IMMUNOGLOBULIN PRODUCTION INVOLVED IN IMMUNOGLOBULIN MEDIATED IMMUNE RESPONSE**	15	0.28	0.74	0.8242	0.9607
**REGULATION OF ERAD PATHWAY**	24	0.25	0.74	0.8536	0.9607
**POSITIVE REGULATION OF NIK NF KAPPAB SIGNALING**	24	0.25	0.74	0.8484	0.9607
**SPECIFICATION OF SYMMETRY**	104	0.18	0.74	0.9343	0.9608
**APOPTOTIC NUCLEAR CHANGES**	22	0.25	0.74	0.8316	0.9609
**NEURON APOPTOTIC PROCESS**	27	0.24	0.74	0.8393	0.9611
**REGULATION OF RELEASE OF SEQUESTERED CALCIUM ION INTO CYTOSOL**	68	-0.20	-0.82	0.8192	0.9611
**GLUTATHIONE PEROXIDASE ACTIVITY**	16	0.27	0.74	0.8182	0.9611
**ADULT LOCOMOTORY BEHAVIOR**	72	-0.19	-0.81	0.8300	0.9612
**PERIKARYON**	98	0.18	0.74	0.9207	0.9613
**REGULATION OF LYASE ACTIVITY**	80	-0.19	-0.82	0.8722	0.9613
**REGULATION OF NEURONAL SYNAPTIC PLASTICITY**	44	0.21	0.74	0.8509	0.9614
**RESPONSE TO COPPER ION**	27	0.24	0.74	0.8281	0.9615
**CLATHRIN BINDING**	56	0.21	0.74	0.8833	0.9616
**MESENCHYME MORPHOGENESIS**	37	0.22	0.73	0.8744	0.9617
**NERVE DEVELOPMENT**	59	0.20	0.73	0.8936	0.9617
**FORMATION OF PRIMARY GERM LAYER**	102	0.18	0.73	0.9367	0.9618
**ERROR PRONE TRANSLESION SYNTHESIS**	15	-0.29	-0.81	0.7043	0.9618
**GLUTATHIONE METABOLIC PROCESS**	47	0.21	0.73	0.9029	0.9619
**CHANNEL REGULATOR ACTIVITY**	122	0.18	0.73	0.9508	0.9621
**RESPONSE TO FOOD**	17	-0.27	-0.81	0.7528	0.9621
**REGULATION OF NEUROTRANSMITTER TRANSPORT**	59	0.20	0.73	0.8901	0.9622
**CARBOHYDRATE BINDING**	212	0.17	0.74	0.9775	0.9622
**POTASSIUM ION BINDING**	15	0.27	0.73	0.8149	0.9624
**REGULATION OF EXTRINSIC APOPTOTIC SIGNALING PATHWAY VIA DEATH DOMAIN RECEPTORS**	50	0.21	0.73	0.9081	0.9627

**TUMOR NECROSIS FACTOR RECEPTOR BINDING**	27	0.23	0.73	0.8553	0.9629
**REGULATION OF PHOSPHOLIPID METABOLIC PROCESS**	55	0.20	0.73	0.8934	0.9630
**POSITIVE REGULATION OF ALCOHOL BIOSYNTHETIC PROCESS**	21	0.25	0.73	0.8287	0.9630
**POSITIVE REGULATION OF ENDOTHELIAL CELL DIFFERENTIATION**	15	-0.27	-0.81	0.7423	0.9630
**DENDRITIC SHAFT**	35	0.22	0.73	0.8593	0.9630
**REGULATION OF MEMBRANE DEPOLARIZATION**	39	0.22	0.73	0.8869	0.9630
**MICROTUBULE END**	21	0.25	0.73	0.8443	0.9631
**NEGATIVE CHEMOTAXIS**	39	0.22	0.73	0.8767	0.9631
**CALMODULIN BINDING**	162	-0.17	-0.81	0.9560	0.9631
**MODULATION OF TRANSCRIPTION IN OTHER ORGANISM INVOLVED IN SYMBIOTIC INTERACTION**	19	0.25	0.73	0.8108	0.9632
**PHOSPHATIDYLINOSITOL KINASE ACTIVITY**	47	0.21	0.73	0.8952	0.9633
**PHENOL CONTAINING COMPOUND BIOSYNTHETIC PROCESS**	31	-0.23	-0.81	0.7696	0.9634
**INTERCELLULAR BRIDGE**	35	-0.23	-0.81	0.7751	0.9639
**ATPASE ACTIVITY COUPLED TO TRANSMEMBRANE MOVEMENT OF IONS PHOSPHORYLATIVE MECHANISM**	33	0.22	0.73	0.8756	0.9641
**POSITIVE REGULATION OF ORGANIC ACID TRANSPORT**	28	-0.24	-0.81	0.7469	0.9653
**POSITIVE REGULATION OF AXON EXTENSION**	33	0.22	0.73	0.8787	0.9657
**RESPONSE TO TESTOSTERONE**	36	0.22	0.73	0.8663	0.9660
**GENITALIA DEVELOPMENT**	40	0.21	0.73	0.8989	0.9661
**HORMONE TRANSPORT**	73	0.19	0.72	0.9204	0.9669
**STEM CELL DIFFERENTIATION**	179	0.17	0.72	0.9808	0.9671
**PURINE NUCLEOBASE METABOLIC PROCESS**	18	-0.27	-0.81	0.7424	0.9672
**CILIARY TIP**	40	0.21	0.72	0.8847	0.9672
**SIN3 TYPE COMPLEX**	15	0.27	0.72	0.8198	0.9672
**SKELETAL MUSCLE TISSUE REGENERATION**	24	0.24	0.72	0.8503	0.9673
**NEUROPEPTIDE RECEPTOR BINDING**	25	0.24	0.72	0.8771	0.9674
**POSITIVE REGULATION OF EPITHELIAL CELL APOPTOTIC PROCESS**	21	0.24	0.72	0.8441	0.9674

**POSITIVE REGULATION OF RECEPTOR MEDIATED ENDOCYTOSIS**	41	0.21	0.72	0.8738	0.9674
**REGULATED EXOCYTOSIS**	202	0.16	0.72	0.9830	0.9675
**MUSCLE CELL PROLIFERATION**	18	0.26	0.72	0.8336	0.9675
**SPINAL CORD PATTERNING**	22	-0.25	-0.80	0.7472	0.9676
**BODY MORPHOGENESIS**	40	0.21	0.72	0.8869	0.9677
**MYELOID CELL HOMEOSTASIS**	72	0.19	0.72	0.9313	0.9677
**BONE CELL DEVELOPMENT**	20	0.25	0.72	0.8452	0.9678
**NEGATIVE REGULATION OF ANOIKIS**	15	-0.29	-0.81	0.7329	0.9678
**EYE PHOTORECEPTOR CELL DIFFERENTIATION**	38	0.21	0.72	0.8951	0.9680
**RESPONSE TO ELECTRICAL STIMULUS**	41	0.21	0.72	0.8925	0.9680
**CELLULAR RESPONSE TO GROWTH HORMONE STIMULUS**	16	-0.27	-0.80	0.7289	0.9682
**PEPTIDYL TYROSINE MODIFICATION**	170	0.17	0.72	0.9755	0.9682
**REGULATION OF PROTEIN TYROSINE KINASE ACTIVITY**	57	-0.20	-0.81	0.8329	0.9683
**MISMATCH REPAIR**	24	0.24	0.72	0.8526	0.9683
**INTRACELLULAR ESTROGEN RECEPTOR SIGNALING PATHWAY**	15	0.26	0.72	0.8351	0.9683
**HOMEOSTASIS OF NUMBER OF CELLS WITHIN A TISSUE**	26	0.24	0.72	0.8564	0.9684
**FC GAMMA RECEPTOR SIGNALING PATHWAY**	67	-0.19	-0.80	0.8665	0.9685
**RESPONSE TO MERCURY ION**	15	0.26	0.72	0.8300	0.9685
**NAD DEPENDENT PROTEIN DEACETYLASE ACTIVITY**	16	0.27	0.72	0.8502	0.9686
**TRACHEA DEVELOPMENT**	18	0.25	0.71	0.8420	0.9690
**ASYMMETRIC PROTEIN LOCALIZATION**	18	-0.26	-0.80	0.7529	0.9691
**REGULATION OF SODIUM ION TRANSMEMBRANE TRANSPORT**	41	0.21	0.71	0.9044	0.9694
**REGULATION OF PHOSPHATIDYLINOSITOL 3 KINASE SIGNALING**	129	-0.17	-0.80	0.9075	0.9695
**NEGATIVE REGULATION OF ORGAN GROWTH**	17	-0.27	-0.81	0.7240	0.9696
**POSITIVE REGULATION OF PROTEIN DEPOLYMERIZATION**	18	-0.27	-0.81	0.7153	0.9697
**POSITIVE REGULATION OF SMALL GTPASE MEDIATED SIGNAL TRANSDUCTION**	35	0.21	0.71	0.8951	0.9699
**ACYLGLYCEROL HOMEOSTASIS**	26	0.23	0.71	0.8556	0.9699

**1 PHOSPHATIDYLINOSITOL 3 KINASE ACTIVITY**	41	0.20	0.71	0.9100	0.9700
**NEGATIVE REGULATION OF DEPHOSPHORYLATION**	64	0.19	0.71	0.9146	0.9701
**LIPOPOLYSACCHARIDE MEDIATED SIGNALING PATHWAY**	30	0.22	0.71	0.8843	0.9701
**FERTILIZATION**	123	0.17	0.71	0.9635	0.9701
**PLACENTA BLOOD VESSEL DEVELOPMENT**	27	0.23	0.71	0.8577	0.9702
**PALLIUM DEVELOPMENT**	145	0.17	0.71	0.9847	0.9703
**TRIGLYCERIDE HOMEOSTASIS**	26	0.23	0.71	0.8711	0.9703
**REGULATION OF MESENCHYMAL CELL PROLIFERATION**	29	0.22	0.71	0.8915	0.9703
**REGULATION OF CALCIUM ION TRANSPORT INTO CYTOSOL**	84	0.18	0.71	0.9481	0.9704
**LABYRINTHINE LAYER DEVELOPMENT**	44	0.20	0.71	0.9038	0.9704
**NEGATIVE REGULATION OF TRANSMEMBRANE TRANSPORT**	80	0.18	0.71	0.9527	0.9704
**CARBOHYDRATE PHOSPHORYLATION**	20	0.25	0.71	0.8611	0.9705
**RNA POLYMERASE II TRANSCRIPTION COREPRESSOR ACTIVITY**	22	0.24	0.71	0.8659	0.9705
**NEGATIVE REGULATION OF LIPID BIOSYNTHETIC PROCESS**	38	-0.22	-0.80	0.8127	0.9711
**GMP METABOLIC PROCESS**	17	0.25	0.71	0.8776	0.9712
**SECRETORY VESICLE**	416	-0.15	-0.80	1.0000	0.9717
**ESTROUS CYCLE**	18	0.25	0.70	0.8657	0.9719
**REGULATION OF EXTRINSIC APOPTOTIC SIGNALING PATHWAY IN ABSENCE OF LIGAND**	42	-0.22	-0.80	0.8005	0.9720
**REGULATION OF INTERLEUKIN 1 SECRETION**	23	-0.25	-0.80	0.7794	0.9721
**DIGESTION**	112	-0.18	-0.80	0.9179	0.9722
**NEURON RECOGNITION**	32	-0.23	-0.80	0.7722	0.9725
**POSITIVE REGULATION OF PROTEIN ACETYLATION**	33	-0.23	-0.80	0.7934	0.9726
**FEAR RESPONSE**	26	-0.24	-0.80	0.7940	0.9727
**CARBOHYDRATE KINASE ACTIVITY**	18	-0.27	-0.80	0.7664	0.9729
**ADRENAL GLAND DEVELOPMENT**	21	-0.25	-0.80	0.7700	0.9729
**ESTABLISHMENT OR MAINTENANCE OF CELL POLARITY**	125	0.17	0.70	0.9813	0.9729
**CELLULAR MODIFIED AMINO ACID CATABOLIC PROCESS**	15	0.26	0.70	0.8463	0.9731

**POSITIVE REGULATION OF CYCLASE ACTIVITY**	56	0.19	0.70	0.9258	0.9731
**STEROID CATABOLIC PROCESS**	20	-0.26	-0.80	0.7683	0.9732
**ACTIN CYTOSKELETON**	390	0.15	0.70	0.9988	0.9732
**SCHWANN CELL DIFFERENTIATION**	29	0.22	0.70	0.9058	0.9734
**RHO GTPASE BINDING**	69	-0.19	-0.80	0.8452	0.9734
**REGULATION OF PHOSPHATIDYLINOSITOL 3 KINASE ACTIVITY**	35	0.21	0.70	0.9025	0.9735
**GLYCOPROTEIN COMPLEX**	19	0.25	0.70	0.8777	0.9735
**NEGATIVE REGULATION OF MULTI ORGANISM PROCESS**	124	0.17	0.70	0.9764	0.9735
**NEURON NEURON SYNAPTIC TRANSMISSION**	52	0.19	0.70	0.9320	0.9736
**POSITIVE REGULATION OF NUCLEAR DIVISION**	55	0.19	0.70	0.9315	0.9736
**CILIARY BASE**	20	0.24	0.70	0.8731	0.9737
**REGULATION OF ARF PROTEIN SIGNAL TRANSDUCTION**	15	0.26	0.70	0.8591	0.9737
**ACTIN BINDING**	342	-0.15	-0.79	0.9944	0.9737
**NEGATIVE REGULATION OF VIRAL ENTRY INTO HOST CELL**	16	0.25	0.69	0.8569	0.9737
**DEFENSE RESPONSE TO FUNGUS**	24	0.23	0.70	0.8950	0.9739
**PEPTIDASE REGULATOR ACTIVITY**	167	0.16	0.70	0.9890	0.9739
**APOPTOTIC CELL CLEARANCE**	23	0.23	0.70	0.8605	0.9740
**ALPHA ACTININ BINDING**	19	0.24	0.69	0.8752	0.9741
**CELL FATE COMMITMENT INVOLVED IN FORMATION OF PRIMARY GERM LAYER**	28	0.22	0.70	0.8854	0.9742
**ESTABLISHMENT OF TISSUE POLARITY**	15	0.26	0.69	0.8574	0.9748
**PROSTATE GLAND DEVELOPMENT**	39	-0.21	-0.79	0.8191	0.9748
**SECRETION BY CELL**	440	0.15	0.69	1.0000	0.9749
**STRIATED MUSCLE CELL DIFFERENTIATION**	162	0.16	0.69	0.9931	0.9749
**REGULATION OF T CELL DIFFERENTIATION IN THYMUS**	23	0.22	0.69	0.8841	0.9750
**OUTFLOW TRACT MORPHOGENESIS**	55	0.19	0.69	0.9303	0.9750
**VACUOLAR LUMEN**	95	-0.18	-0.79	0.9421	0.9750
**MODULATION OF EXCITATORY POSTSYNAPTIC POTENTIAL**	29	0.22	0.69	0.8767	0.9750
**CYTOKINE SECRETION**	30	0.22	0.69	0.9037	0.9751
**REGULATION OF PROTEIN MATURATION**	68	-0.19	-0.79	0.8716	0.9751
**ESTABLISHMENT OF CELL POLARITY**	79	0.18	0.69	0.9630	0.9751

**POLYOL BIOSYNTHETIC PROCESS**	23	0.23	0.69	0.8932	0.9751
**NEGATIVE REGULATION OF CELL JUNCTION ASSEMBLY**	18	0.24	0.69	0.8713	0.9751
**MUSCLE CELL DEVELOPMENT**	117	0.17	0.69	0.9822	0.9752
**CHANNEL INHIBITOR ACTIVITY**	33	0.21	0.69	0.9179	0.9752
**SOLUTE CATION ANTIPORTER ACTIVITY**	28	0.22	0.69	0.9089	0.9753
**SECRETORY GRANULE LUMEN**	73	0.18	0.69	0.9475	0.9754
**AUTONOMIC NERVOUS SYSTEM DEVELOPMENT**	37	-0.22	-0.79	0.8187	0.9754
**REGULATION OF HEART RATE BY CARDIAC CONDUCTION**	29	-0.23	-0.79	0.7819	0.9754
**SEX CHROMOSOME**	27	0.22	0.69	0.9067	0.9756
**SUBSTANTIA NIGRA DEVELOPMENT**	38	0.20	0.68	0.9408	0.9756
**MUCOPOLYSACCHARIDE METABOLIC PROCESS**	95	-0.18	-0.79	0.9323	0.9757
**REGULATION OF INTERLEUKIN 2 PRODUCTION**	44	0.20	0.69	0.9241	0.9757
**FATTY ACID DERIVATIVE METABOLIC PROCESS**	64	-0.19	-0.79	0.8712	0.9757
**ACTIN FILAMENT BUNDLE ORGANIZATION**	45	0.19	0.68	0.9285	0.9757
**SKIN EPIDERMIS DEVELOPMENT**	65	-0.19	-0.79	0.8787	0.9757
**REGULATION OF CALCIUM ION TRANSPORT**	191	-0.16	-0.79	0.9502	0.9757
**NEURAL PRECURSOR CELL PROLIFERATION**	66	-0.19	-0.79	0.8678	0.9757
**CARDIAC CONDUCTION**	77	-0.19	-0.79	0.8954	0.9758
**GLIAL CELL DIFFERENTIATION**	128	0.17	0.68	0.9849	0.9759
**ASPARTIC TYPE PEPTIDASE ACTIVITY**	17	-0.26	-0.79	0.7754	0.9760
**FILOPODIUM**	89	-0.18	-0.79	0.9103	0.9760
**POSITIVE REGULATION OF LYASE ACTIVITY**	56	0.19	0.68	0.9495	0.9761
**REGULATION OF STEM CELL POPULATION MAINTENANCE**	15	-0.28	-0.79	0.7466	0.9761
**PLATELET ACTIVATION**	132	-0.17	-0.79	0.9585	0.9762
**HYDROLASE ACTIVITY HYDROLYZING N GLYCOSYL COMPOUNDS**	18	-0.26	-0.79	0.7815	0.9764
**ICOSANOID METABOLIC PROCESS**	64	-0.19	-0.78	0.8778	0.9764
**NAD ADP RIBOSYLTRANSFERASE ACTIVITY**	19	-0.26	-0.79	0.7635	0.9766
**REGULATION OF CHROMATIN SILENCING**	20	-0.25	-0.78	0.7780	0.9767
**INTRACELLULAR LIGAND GATED ION CHANNEL ACTIVITY**	28	-0.23	-0.78	0.8325	0.9768

**INORGANIC CATION TRANSMEMBRANE TRANSPORTER ACTIVITY**	483	-0.14	-0.78	1.0000	0.9769
**PROSTATE GLAND MORPHOGENESIS**	22	-0.24	-0.78	0.7838	0.9769
**POSITIVE REGULATION OF TELOMERE CAPPING**	15	0.25	0.68	0.8571	0.9770
**MECHANORECEPTOR DIFFERENTIATION**	46	0.20	0.68	0.9387	0.9770
**NEGATIVE REGULATION OF EXOCYTOSIS**	24	0.22	0.68	0.8962	0.9771
**ACTIN FILAMENT BASED MOVEMENT**	87	-0.18	-0.78	0.8895	0.9771
**MYOBLAST FUSION**	19	-0.25	-0.78	0.7899	0.9772
**REGULATION OF RECEPTOR RECYCLING**	18	-0.26	-0.78	0.7973	0.9772
**PLACENTA DEVELOPMENT**	129	0.16	0.68	0.9903	0.9773
**MACROPHAGE ACTIVATION**	29	0.22	0.68	0.9215	0.9773
**INSULIN SECRETION**	37	0.20	0.68	0.9206	0.9774
**TELOMERE MAINTENANCE VIA RECOMBINATION**	30	-0.22	-0.78	0.8147	0.9775
**REGULATION OF PROTEIN TARGETING TO MEMBRANE**	23	-0.24	-0.78	0.7980	0.9776
**POSITIVE REGULATION OF MONOOXYGENASE ACTIVITY**	25	0.23	0.68	0.8770	0.9780
**SYNCYTIUM FORMATION**	23	0.22	0.67	0.8986	0.9780
**OLIGODENDROCYTE DIFFERENTIATION**	56	0.19	0.67	0.9624	0.9781
**ROUGH ENDOPLASMIC RETICULUM**	66	0.18	0.67	0.9617	0.9781
**GAP JUNCTION**	26	0.22	0.68	0.9171	0.9781
**CENTRIOLE ASSEMBLY**	16	0.24	0.66	0.8907	0.9783
**REGULATION OF THYMOCYTE AGGREGATION**	23	0.22	0.67	0.9085	0.9783
**GTP RHO BINDING**	15	0.25	0.67	0.8741	0.9783
**MRNA TRANSCRIPTION**	20	0.23	0.67	0.9034	0.9784
**REGULATION OF POSTTRANSCRIPTIONAL GENE SILENCING**	20	0.22	0.66	0.9099	0.9784
**CORTICAL CYTOSKELETON ORGANIZATION**	30	0.21	0.67	0.9103	0.9784
**MYELOID LEUKOCYTE MEDIATED IMMUNITY**	37	0.20	0.67	0.9252	0.9785
**PEPTIDASE ACTIVATOR ACTIVITY INVOLVED IN APOPTOTIC PROCESS**	17	0.24	0.67	0.8791	0.9785
**RESPONSE TO FUNGUS**	36	0.20	0.66	0.9210	0.9786
**CELLULAR RESPONSE TO CORTICOSTEROID STIMULUS**	52	0.19	0.67	0.9540	0.9787
**CELLULAR RESPONSE TO RETINOIC ACID**	61	0.18	0.67	0.9501	0.9788
**DEVELOPMENTAL INDUCTION**	27	0.21	0.67	0.9086	0.9790
**CELLULAR RESPONSE TO VITAMIN**	25	0.22	0.67	0.9134	0.9790

**NUCLEAR OUTER MEMBRANE**	21	0.23	0.67	0.9031	0.9791
**MORPHOGENESIS OF AN EPITHELIAL SHEET**	41	0.20	0.67	0.9421	0.9794
**B CELL ACTIVATION INVOLVED IN IMMUNE RESPONSE**	31	0.21	0.67	0.9351	0.9794
**NEGATIVE REGULATION OF CATION CHANNEL ACTIVITY**	32	0.20	0.67	0.9289	0.9796
**REGULATION OF TOLL LIKE RECEPTOR SIGNALING PATHWAY**	43	-0.21	-0.78	0.8401	0.9797
**REGULATION OF NUCLEAR TRANSCRIBED MRNA CATABOLIC PROCESS DEADENYLATION DEPENDENT DECAY**	15	0.24	0.67	0.9000	0.9798
**DNA CATABOLIC PROCESS ENDONUCLEOLYTIC**	17	0.24	0.66	0.8991	0.9803
**ESTABLISHMENT OF SPINDLE ORIENTATION**	24	0.22	0.66	0.9212	0.9805
**HEAD MORPHOGENESIS**	33	0.21	0.66	0.9173	0.9807
**ACROSOMAL MEMBRANE**	21	0.22	0.66	0.9063	0.9807
**REGULATION OF NEUROTRANSMITTER LEVELS**	171	0.15	0.66	0.9972	0.9807
**MITOTIC RECOMBINATION**	36	0.20	0.66	0.9301	0.9808
**CELLULAR RESPONSE TO DSRNA**	35	0.20	0.66	0.9370	0.9809
**PHOSPHATIDYLINOSITOL 3 KINASE BINDING**	27	0.21	0.66	0.9321	0.9809
**MESODERMAL CELL DIFFERENTIATION**	25	0.21	0.65	0.9155	0.9816
**CELLULAR RESPONSE TO OSMOTIC STRESS**	18	0.23	0.65	0.9078	0.9816
**POSITIVE REGULATION OF SIGNAL TRANSDUCTION BY P53 CLASS MEDIATOR**	15	0.24	0.65	0.8929	0.9818
**EXOCRINE SYSTEM DEVELOPMENT**	42	0.19	0.65	0.9551	0.9820
**NEURON PROJECTION MEMBRANE**	33	-0.22	-0.77	0.8199	0.9830
**CELLULAR RESPONSE TO ACETYLCHOLINE**	18	-0.26	-0.77	0.7588	0.9838
**NEGATIVE REGULATION OF VIRAL PROCESS**	69	-0.18	-0.77	0.9153	0.9840
**REGULATION OF INTRACELLULAR ESTROGEN RECEPTOR SIGNALING PATHWAY**	23	0.22	0.65	0.9247	0.9841
**POSITIVE REGULATION OF NUCLEAR TRANSCRIBED MRNA CATABOLIC PROCESS DEADENYLATION DEPENDENT DECAY**	15	0.24	0.64	0.9056	0.9844

**INNER EAR RECEPTOR CELL DEVELOPMENT**	32	-0.22	-0.77	0.8469	0.9845
**CYTOPLASMIC SIDE OF MEMBRANE**	145	-0.16	-0.77	0.9804	0.9845
**CRANIAL SKELETAL SYSTEM DEVELOPMENT**	53	0.18	0.64	0.9581	0.9846
**ADULT WALKING BEHAVIOR**	28	-0.22	-0.77	0.8262	0.9846
**RESPONSE TO ACETYLCHOLINE**	18	-0.26	-0.77	0.7945	0.9847
**MUSCLE MYOSIN COMPLEX**	17	-0.26	-0.77	0.7986	0.9847
**NUCLEOTIDE KINASE ACTIVITY**	21	0.22	0.64	0.9519	0.9847
**EXOCYST**	15	-0.27	-0.76	0.8160	0.9847
**NEGATIVE REGULATION OF INTRACELLULAR STEROID HORMONE RECEPTOR SIGNALING PATHWAY**	28	0.21	0.64	0.9426	0.9848
**SIGNAL TRANSDUCTION INVOLVED IN CELLULAR RESPONSE TO AMMONIUM ION**	18	-0.26	-0.76	0.8333	0.9848
**EMBRYONIC SKELETAL SYSTEM DEVELOPMENT**	112	-0.17	-0.77	0.9486	0.9849
**ACID SECRETION**	65	-0.19	-0.76	0.9218	0.9849
**GLIAL CELL MIGRATION**	33	-0.22	-0.76	0.8555	0.9850
**POSITIVE REGULATION OF OSTEOBLAST DIFFERENTIATION**	54	-0.19	-0.77	0.8740	0.9850
**LYMPH NODE DEVELOPMENT**	17	-0.26	-0.77	0.7973	0.9850
**REGULATION OF CELL SHAPE**	119	-0.17	-0.77	0.9431	0.9851
**REGULATION OF FIBROBLAST GROWTH FACTOR RECEPTOR SIGNALING PATHWAY**	25	-0.23	-0.76	0.8305	0.9852
**MATURE B CELL DIFFERENTIATION**	16	-0.27	-0.77	0.7653	0.9852
**G PROTEIN COUPLED ACETYLCHOLINE RECEPTOR SIGNALING PATHWAY**	18	-0.26	-0.76	0.8133	0.9853
**CELL SUBSTRATE ADHESION**	150	-0.16	-0.76	0.9665	0.9855
**POSTSYNAPTIC SIGNAL TRANSDUCTION**	18	-0.26	-0.77	0.7459	0.9857
**WATER TRANSPORT**	18	0.22	0.64	0.8973	0.9857
**COSTAMERE**	17	-0.26	-0.77	0.7920	0.9863
**FILAMENTOUS ACTIN**	20	0.22	0.64	0.9333	0.9865
**ACETYLCHOLINE RECEPTOR SIGNALING PATHWAY**	18	-0.26	-0.76	0.8182	0.9866
**DEFENSE RESPONSE TO GRAM NEGATIVE BACTERIUM**	31	-0.22	-0.77	0.8272	0.9866
**WALKING BEHAVIOR**	28	-0.22	-0.76	0.8377	0.9868
**BASE EXCISION REPAIR**	31	0.19	0.63	0.9583	0.9873
**RESPONSE TO SALT**	16	0.23	0.63	0.9235	0.9874

**REGULATION OF GENE SILENCING BY RNA**	20	0.22	0.63	0.9226	0.9876
**CATECHOLAMINE BIOSYNTHETIC PROCESS**	18	-0.25	-0.76	0.7916	0.9876
**VESICLE DOCKING INVOLVED IN EXOCYTOSIS**	33	0.19	0.63	0.9595	0.9876
**LATERAL PLASMA MEMBRANE**	45	0.18	0.63	0.9772	0.9877
**CRANIAL NERVE DEVELOPMENT**	38	0.19	0.63	0.9607	0.9877
**BIOMINERAL TISSUE DEVELOPMENT**	68	0.17	0.63	0.9741	0.9877
**STRUCTURAL CONSTITUENT OF EYE LENS**	19	0.22	0.63	0.9201	0.9877
**NEGATIVE REGULATION OF EMBRYONIC DEVELOPMENT**	25	0.21	0.63	0.9371	0.9879
**IMMUNOLOGICAL SYNAPSE**	31	0.20	0.63	0.9548	0.9879
**EPHRIN RECEPTOR ACTIVITY**	18	-0.25	-0.76	0.8242	0.9883
**CALCIUM DEPENDENT CELL CELL ADHESION VIA PLASMA MEMBRANE CELL ADHESION MOLECULES**	24	0.21	0.62	0.9508	0.9883
**MORPHOGENESIS OF A BRANCHING STRUCTURE**	158	0.15	0.62	1.0000	0.9883
**REGULATION OF MEIOTIC NUCLEAR DIVISION**	29	0.20	0.62	0.9479	0.9886
**SULFUR COMPOUND TRANSPORT**	27	0.20	0.62	0.9399	0.9886
**MOLTING CYCLE**	73	0.16	0.62	0.9791	0.9887
**METANEPHRIC EPITHELIUM DEVELOPMENT**	20	0.22	0.62	0.9277	0.9887
**ARGININE METABOLIC PROCESS**	15	0.24	0.62	0.9228	0.9888
**NEGATIVE REGULATION OF PROTEIN LOCALIZATION TO CELL PERIPHERY**	17	0.22	0.62	0.9203	0.9891
**NEGATIVE REGULATION OF CALCIUM ION TRANSPORT**	43	0.18	0.62	0.9759	0.9894
**CALCIUM ACTIVATED CATION CHANNEL ACTIVITY**	27	-0.23	-0.76	0.8474	0.9894
**CGMP BIOSYNTHETIC PROCESS**	15	-0.26	-0.75	0.8067	0.9894
**HAIR CYCLE**	73	0.16	0.62	0.9910	0.9894
**MESODERM DEVELOPMENT**	100	-0.17	-0.75	0.9439	0.9896
**EPIBOLY**	22	-0.24	-0.75	0.7986	0.9899
**VASOCONSTRICTION**	27	-0.23	-0.75	0.8409	0.9904
**CARDIAC SEPTUM MORPHOGENESIS**	49	-0.20	-0.75	0.8911	0.9906
**ACTOMYOSIN STRUCTURE ORGANIZATION**	71	0.16	0.61	0.9893	0.9908
**STRUCTURAL CONSTITUENT OF MUSCLE**	38	0.18	0.61	0.9662	0.9909
**REGULATION OF CALCIUM ION TRANSMEMBRANE TRANSPORT**	108	0.15	0.60	0.9958	0.9909

**SARCOPLASM**	62	0.16	0.60	0.9909	0.9910
**HISTONE KINASE ACTIVITY**	18	0.21	0.60	0.9371	0.9910
**PROTEIN TRIMERIZATION**	34	-0.21	-0.75	0.8551	0.9910
**PHOSPHATIDYLINOSITOL 3 4 5 TRISPHOSPHATE BINDING**	31	0.19	0.61	0.9537	0.9911
**RESPONSE TO RETINOIC ACID**	95	0.15	0.61	0.9928	0.9912
**MESODERM MORPHOGENESIS**	60	0.16	0.60	0.9955	0.9912
**REGULATION OF SYNAPTIC PLASTICITY**	134	0.15	0.61	0.9986	0.9912
**NEGATIVE REGULATION OF PROTEIN LOCALIZATION TO PLASMA MEMBRANE**	17	0.22	0.60	0.9503	0.9913
**HYALURONIC ACID BINDING**	19	0.21	0.60	0.9304	0.9913
**REGULATION OF B CELL DIFFERENTIATION**	20	0.20	0.60	0.9661	0.9916
**ACTIVATION OF GTPASE ACTIVITY**	70	0.16	0.59	0.9953	0.9916
**CYTOKINE METABOLIC PROCESS**	17	-0.25	-0.74	0.8103	0.9918
**LUNG MORPHOGENESIS**	43	-0.20	-0.73	0.9003	0.9918
**PROTEIN PHOSPHATASE 1 BINDING**	16	0.22	0.60	0.9696	0.9918
**PSEUDOPODIUM**	16	0.21	0.60	0.9556	0.9919
**REGULATION OF GENE SILENCING**	42	0.17	0.59	0.9811	0.9920
**RNA POLYMERASE II DISTAL ENHANCER SEQUENCE SPECIFIC DNA BINDING**	60	0.16	0.59	0.9938	0.9921
**NEGATIVE REGULATION OF REGULATED SECRETORY PATHWAY**	18	-0.24	-0.74	0.8349	0.9922
**POSITIVE REGULATION OF LYMPHOCYTE APOPTOTIC PROCESS**	16	-0.26	-0.73	0.8420	0.9923
**REGULATION OF CHEMOKINE PRODUCTION**	58	-0.18	-0.74	0.9182	0.9923
**ESTABLISHMENT OF MITOTIC SPINDLE ORIENTATION**	19	0.21	0.59	0.9564	0.9924
**MATING**	31	-0.21	-0.74	0.8575	0.9925
**CHONDROITIN SULFATE PROTEOGLYCAN METABOLIC PROCESS**	39	-0.20	-0.73	0.8985	0.9926
**SYMPATHETIC NERVOUS SYSTEM DEVELOPMENT**	17	0.21	0.59	0.9549	0.9926
**MATERNAL PLACENTA DEVELOPMENT**	28	-0.22	-0.73	0.8320	0.9927
**HEXOSE CATABOLIC PROCESS**	43	0.17	0.58	0.9754	0.9927
**CATECHOL CONTAINING COMPOUND BIOSYNTHETIC PROCESS**	18	-0.25	-0.74	0.8163	0.9928
**MIDDLE EAR MORPHOGENESIS**	20	-0.24	-0.73	0.8469	0.9928
**REGULATION OF LIPID KINASE ACTIVITY**	42	0.17	0.59	0.9837	0.9929
**REGULATION OF CHOLESTEROL EFFLUX**	17	-0.25	-0.75	0.8140	0.9929

**POSITIVE REGULATION OF EPIDERMAL CELL DIFFERENTIATION**	17	-0.25	-0.75	0.8299	0.9929
**NEURAL RETINA DEVELOPMENT**	47	-0.20	-0.74	0.9030	0.9929
**SYNAPSIS**	28	-0.21	-0.73	0.8689	0.9930
**NEGATIVE REGULATION OF ACTIN FILAMENT DEPOLYMERIZATION**	25	-0.23	-0.74	0.8627	0.9932
**POSITIVE REGULATION OF CHEMOKINE PRODUCTION**	42	-0.20	-0.74	0.8966	0.9932
**GLUCOSE 6 PHOSPHATE METABOLIC PROCESS**	19	-0.24	-0.74	0.8342	0.9933
**DETECTION OF STIMULUS**	194	-0.15	-0.73	0.9796	0.9934
**CELL MATRIX ADHESION**	111	-0.17	-0.75	0.9439	0.9934
**POSITIVE REGULATION OF PHOSPHOLIPID METABOLIC PROCESS**	37	-0.20	-0.74	0.8972	0.9935
**REGULATION OF INTERLEUKIN 13 PRODUCTION**	15	-0.26	-0.74	0.8122	0.9935
**FEMALE GENITALIA DEVELOPMENT**	15	-0.26	-0.74	0.8248	0.9935
**SPERMATID NUCLEUS DIFFERENTIATION**	15	-0.27	-0.75	0.7831	0.9936
**HEPARAN SULFATE PROTEOGLYCAN BINDING**	16	-0.26	-0.74	0.8288	0.9937
**MUSCLE CELL DIFFERENTIATION**	219	-0.15	-0.74	0.9919	0.9938
**REGULATION OF ERYTHROCYTE DIFFERENTIATION**	32	-0.21	-0.74	0.8915	0.9942
**POSITIVE REGULATION OF OSSIFICATION**	77	-0.18	-0.75	0.9466	0.9942
**HISTONE PHOSPHORYLATION**	24	-0.23	-0.73	0.8679	0.9944
**SPERM FLAGELLUM**	48	-0.19	-0.75	0.9195	0.9944
**REGULATION OF INTERFERON BETA PRODUCTION**	42	-0.19	-0.72	0.9229	0.9946
**NEURON PROJECTION REGENERATION**	29	0.18	0.57	0.9778	0.9947
**DENDRITE CYTOPLASM**	15	-0.25	-0.71	0.8401	0.9947
**FOCAL ADHESION ASSEMBLY**	21	0.20	0.57	0.9602	0.9947
**REGULATION OF ADHERENS JUNCTION ORGANIZATION**	45	-0.19	-0.71	0.9321	0.9947
**A BAND**	32	-0.20	-0.71	0.9131	0.9948
**SPECTRIN BINDING**	18	-0.24	-0.73	0.8689	0.9949
**RHYTHMIC BEHAVIOR**	16	0.20	0.56	0.9626	0.9949
**NEUROBLAST PROLIFERATION**	29	0.18	0.57	0.9748	0.9949
**EMBRYONIC EYE MORPHOGENESIS**	31	0.17	0.56	0.9802	0.9950
**NEGATIVE REGULATION OF GENE SILENCING**	19	0.20	0.57	0.9653	0.9950

**POSITIVE REGULATION OF INTERLEUKIN 8 PRODUCTION**	38	-0.19	-0.72	0.9280	0.9950
**EXOCYTIC VESICLE MEMBRANE**	53	-0.18	-0.72	0.9319	0.9951
**NEGATIVE REGULATION OF BIOMINERAL TISSUE DEVELOPMENT**	18	0.19	0.56	0.9579	0.9951
**SOMATIC STEM CELL DIVISION**	22	-0.23	-0.72	0.8935	0.9951
**CELL SUBSTRATE ADHERENS JUNCTION ASSEMBLY**	21	0.20	0.57	0.9688	0.9952
**RESPONSE TO ESTRADIOL**	135	-0.15	-0.72	0.9853	0.9952
**MUSCLE FIBER DEVELOPMENT**	40	0.17	0.57	0.9819	0.9952
**NEGATIVE REGULATION OF MYELOID LEUKOCYTE DIFFERENTIATION**	41	0.17	0.56	0.9836	0.9952
**DNA POLYMERASE ACTIVITY**	32	-0.21	-0.71	0.9142	0.9953
**HOMOLOGOUS CHROMOSOME SEGREGATION**	36	-0.20	-0.71	0.9323	0.9953
**NEGATIVE REGULATION OF TYPE I INTERFERON PRODUCTION**	34	-0.20	-0.71	0.9182	0.9954
**GLIOGENESIS**	163	-0.15	-0.71	0.9926	0.9954
**POSITIVE REGULATION OF SYNAPTIC TRANSMISSION GLUTAMATERGIC**	16	-0.25	-0.73	0.8529	0.9955
**AMINOGLYCAN CATABOLIC PROCESS**	60	-0.17	-0.72	0.9547	0.9955
**PEPTIDYL GLUTAMIC ACID MODIFICATION**	25	-0.22	-0.72	0.8412	0.9955
**ACUTE PHASE RESPONSE**	34	-0.21	-0.72	0.9028	0.9955
**NEUROTRANSMITTER TRANSPORT**	143	-0.15	-0.72	0.9829	0.9956
**POSITIVE REGULATION OF CYTOKINESIS**	30	-0.21	-0.72	0.9091	0.9956
**REGULATION OF MITOCHONDRIAL DEPOLARIZATION**	16	-0.23	-0.67	0.9062	0.9957
**STEM CELL PROLIFERATION**	58	-0.18	-0.72	0.9133	0.9957
**REGULATION OF NEUROTRANSMITTER SECRETION**	48	-0.18	-0.71	0.9413	0.9957
**GLUTAMATE RECEPTOR SIGNALING PATHWAY**	38	-0.19	-0.71	0.9319	0.9957
**LYMPH VESSEL DEVELOPMENT**	19	-0.22	-0.68	0.8998	0.9958
**REGULATION OF SMOOTH MUSCLE CELL DIFFERENTIATION**	18	-0.24	-0.71	0.8616	0.9958
**NEGATIVE REGULATION OF CYTOKINE BIOSYNTHETIC PROCESS**	25	-0.19	-0.61	0.9661	0.9959
**REGULATION OF TOLL LIKE RECEPTOR 4 SIGNALING PATHWAY**	15	-0.25	-0.71	0.8408	0.9959
**NUCLEOTIDE EXCISION REPAIR DNA GAP FILLING**	20	-0.23	-0.71	0.8732	0.9959
**GOLGI LUMEN**	78	-0.17	-0.73	0.9482	0.9960

**CELLULAR RESPONSE TO GLUCAGON STIMULUS**	33	-0.16	-0.55	0.9946	0.9960
**POSITIVE REGULATION OF NEUROLOGICAL SYSTEM PROCESS**	21	0.19	0.55	0.9863	0.9961
**EMBRYONIC CAMERA TYPE EYE DEVELOPMENT**	34	-0.19	-0.67	0.9293	0.9961
**ENHANCER BINDING**	86	-0.16	-0.72	0.9719	0.9962
**ENDODERM FORMATION**	48	-0.19	-0.72	0.9195	0.9962
**POSITIVE REGULATION OF FILOPODIUM ASSEMBLY**	22	-0.19	-0.61	0.9672	0.9962
**NEGATIVE REGULATION OF REPRODUCTIVE PROCESS**	51	-0.17	-0.67	0.9699	0.9962
**TRANSLESION SYNTHESIS**	30	-0.21	-0.72	0.9074	0.9962
**PARAXIAL MESODERM DEVELOPMENT**	15	-0.24	-0.68	0.8858	0.9963
**SECONDARY METABOLITE BIOSYNTHETIC PROCESS**	18	-0.24	-0.70	0.8698	0.9964
**ERK1 AND ERK2 CASCADE**	22	-0.18	-0.58	0.9730	0.9965
**CELLULAR RESPONSE TO FLUID SHEAR STRESS**	17	-0.21	-0.62	0.9236	0.9965
**ACTIVIN RECEPTOR SIGNALING PATHWAY**	22	-0.23	-0.73	0.8544	0.9965
**GLYCOPROTEIN BINDING**	91	-0.16	-0.67	0.9910	0.9966
**RESPONSE TO PAIN**	25	-0.17	-0.55	0.9924	0.9966
**CRANIAL NERVE MORPHOGENESIS**	20	-0.22	-0.68	0.8680	0.9966
**NEGATIVE REGULATION OF MEIOTIC CELL CYCLE**	18	-0.20	-0.59	0.9513	0.9967
**REGULATION OF EMBRYONIC DEVELOPMENT**	109	-0.16	-0.70	0.9794	0.9967
**MICROFILAMENT MOTOR ACTIVITY**	20	-0.17	-0.53	0.9739	0.9967
**NEGATIVE REGULATION OF STRESS FIBER ASSEMBLY**	15	-0.25	-0.70	0.8633	0.9967
**POSITIVE REGULATION OF PROTEIN COMPLEX DISASSEMBLY**	23	-0.19	-0.61	0.9551	0.9968
**POSITIVE REGULATION OF CALCIUM MEDIATED SIGNALING**	36	-0.17	-0.62	0.9718	0.9968
**RECIPROCAL DNA RECOMBINATION**	33	-0.18	-0.62	0.9658	0.9968
**M BAND**	19	-0.19	-0.59	0.9597	0.9968
**ORGAN FORMATION**	29	-0.21	-0.72	0.8750	0.9968
**AXONAL FASCICULATION**	19	-0.22	-0.68	0.9135	0.9969
**CARDIAC MUSCLE CELL ACTION POTENTIAL**	36	-0.16	-0.56	0.9950	0.9969
**ACTIN FILAMENT BUNDLE**	52	-0.14	-0.57	0.9971	0.9969
**GABA RECEPTOR ACTIVITY**	22	-0.22	-0.69	0.8884	0.9969

**PHOTORECEPTOR CELL DIFFERENTIATION**	46	0.16	0.55	0.9921	0.9969
**DERMATAN SULFATE PROTEOGLYCAN METABOLIC PROCESS**	16	-0.23	-0.67	0.9009	0.9970
**DETECTION OF LIGHT STIMULUS INVOLVED IN SENSORY PERCEPTION**	16	-0.24	-0.68	0.8635	0.9970
**FRIZZLED BINDING**	35	-0.16	-0.57	0.9900	0.9970
**GABA RECEPTOR COMPLEX**	18	-0.22	-0.67	0.9145	0.9970
**NEGATIVE REGULATION OF OSTEOCLAST DIFFERENTIATION**	22	-0.18	-0.58	0.9672	0.9970
**RESPONSE TO THYROID HORMONE**	17	-0.20	-0.58	0.9638	0.9971
**ANATOMICAL STRUCTURE ARRANGEMENT**	16	-0.20	-0.59	0.9509	0.9971
**MITOTIC SPINDLE**	46	-0.18	-0.68	0.9583	0.9971
**NEURAL NUCLEUS DEVELOPMENT**	57	-0.15	-0.59	0.9948	0.9971
**RECIPROCAL MEIOTIC RECOMBINATION**	33	-0.18	-0.62	0.9537	0.9971
**ANKYRIN BINDING**	16	-0.19	-0.55	0.9669	0.9972
**PHOTOTRANSDUCTION VISIBLE LIGHT**	20	-0.17	-0.53	0.9868	0.9972
**SPERM PART**	125	-0.15	-0.70	0.9966	0.9972
**HYPEROSMOTIC RESPONSE**	19	-0.19	-0.57	0.9714	0.9972
**POSITIVE REGULATION OF INTERFERON ALPHA PRODUCTION**	16	-0.20	-0.56	0.9652	0.9972
**LYSOSOMAL LUMEN**	75	-0.17	-0.69	0.9550	0.9973
**LIPASE INHIBITOR ACTIVITY**	16	-0.21	-0.61	0.9633	0.9973
**CELLULAR RESPONSE TO VIRUS**	20	0.19	0.54	0.9775	0.9973
**IONOTROPIC GLUTAMATE RECEPTOR SIGNALING PATHWAY**	22	-0.22	-0.70	0.8859	0.9974
**XY BODY**	15	-0.22	-0.62	0.9341	0.9974
**POSITIVE REGULATION OF LEUKOCYTE DEGRANULATION**	15	-0.20	-0.59	0.9615	0.9974
**POSITIVE REGULATION OF ACTIN NUCLEATION**	15	-0.20	-0.60	0.9529	0.9974
**ACTIN FILAMENT**	67	-0.13	-0.54	1.0000	0.9975
**ACTION POTENTIAL**	90	-0.16	-0.69	0.9783	0.9975
**CYTOSOLIC CALCIUM ION TRANSPORT**	46	-0.18	-0.70	0.9492	0.9975
**KERATAN SULFATE METABOLIC PROCESS**	29	-0.20	-0.70	0.9145	0.9975
**NEGATIVE REGULATION OF AXON GUIDANCE**	27	-0.20	-0.67	0.9430	0.9975
**NEGATIVE REGULATION OF CATION TRANSMEMBRANE TRANSPORT**	57	-0.15	-0.60	0.9915	0.9975
**DETECTION OF LIGHT STIMULUS INVOLVED IN VISUAL PERCEPTION**	16	-0.24	-0.68	0.9031	0.9975

**STEM CELL DIVISION**	29	-0.16	-0.55	0.9851	0.9975
**AXON REGENERATION**	20	-0.20	-0.61	0.9688	0.9976
**ECTODERM DEVELOPMENT**	20	-0.20	-0.59	0.9510	0.9976
**MYOSIN BINDING**	56	0.15	0.54	0.9969	0.9976
**MYOFIBRIL ASSEMBLY**	45	-0.18	-0.68	0.9658	0.9976
**REGULATION OF INTERFERON ALPHA PRODUCTION**	19	-0.17	-0.52	0.9810	0.9976
**REGULATION OF PROTEIN AUTOPHOSPHORYLATION**	32	-0.20	-0.69	0.9188	0.9978
**CHONDROITIN SULFATE BIOSYNTHETIC PROCESS**	24	-0.19	-0.62	0.9529	0.9978
**REGULATION OF LONG TERM NEURONAL SYNAPTIC PLASTICITY**	21	-0.22	-0.69	0.8769	0.9978
**REGULATION OF CARDIAC MUSCLE CELL MEMBRANE REPOLARIZATION**	20	-0.16	-0.51	0.9924	0.9978
**REGULATION OF LONG TERM SYNAPTIC POTENTIATION**	18	-0.18	-0.54	0.9861	0.9979
**POSITIVE REGULATION OF EPIDERMIS DEVELOPMENT**	28	-0.18	-0.61	0.9653	0.9979
**PHOSPHATIDYLINOSITOL 3 KINASE SIGNALING**	23	-0.19	-0.60	0.9584	0.9979
**FLUID TRANSPORT**	25	-0.18	-0.60	0.9758	0.9979
**OSTEOBLAST DEVELOPMENT**	17	-0.23	-0.69	0.9067	0.9979
**KINESIN BINDING**	26	-0.21	-0.68	0.9242	0.9980
**ACROSOMAL VESICLE**	84	-0.16	-0.70	0.9732	0.9980
**BRANCH ELONGATION OF AN EPITHELIUM**	17	-0.23	-0.69	0.8708	0.9981
**REGULATION OF NEURON PROJECTION REGENERATION**	18	-0.23	-0.68	0.8947	0.9981
**MYOTUBE DIFFERENTIATION**	50	-0.18	-0.69	0.9553	0.9982
**BASEMENT MEMBRANE**	84	0.14	0.53	0.9970	0.9982
**REGULATION OF KERATINOCYTE DIFFERENTIATION**	23	-0.19	-0.62	0.9455	0.9984
**POSITIVE REGULATION OF INTERLEUKIN 1 SECRETION**	16	0.19	0.53	0.9799	0.9984
**PROTEIN SERINE THREONINE TYROSINE KINASE ACTIVITY**	34	-0.20	-0.69	0.9177	0.9985
**NEGATIVE REGULATION OF STEM CELL PROLIFERATION**	15	-0.16	-0.45	0.9955	0.9987
**DEFINITIVE HEMOPOIESIS**	16	-0.23	-0.66	0.9071	0.9987
**MEMBRANE REPOLARIZATION**	15	-0.24	-0.69	0.8732	0.9987

**PROTEIN SERINE THREONINE KINASE ACTIVATOR ACTIVITY**	18	0.17	0.47	1.0000	0.9987
**TISSUE HOMEOSTASIS**	140	-0.15	-0.69	1.0000	0.9989
**SYNAPTONEMAL COMPLEX**	29	0.16	0.51	0.9983	0.9990
**HORMONE BINDING**	62	0.13	0.48	0.9984	0.9990
**REGULATION OF MEMBRANE REPOLARIZATION**	29	0.15	0.47	0.9951	0.9990
**POSITIVE REGULATION OF EXTRACELLULAR MATRIX ORGANIZATION**	16	-0.22	-0.62	0.9365	0.9990
**POSITIVE REGULATION OF INTERLEUKIN 4 PRODUCTION**	20	-0.15	-0.45	0.9974	0.9991
**ACTIN CYTOSKELETON REORGANIZATION**	51	0.13	0.49	0.9984	0.9991
**RESPONSE TO MAGNESIUM ION**	22	0.17	0.49	0.9886	0.9991
**ESTABLISHMENT OF MITOTIC SPINDLE LOCALIZATION**	22	0.16	0.48	0.9965	0.9991
**STRIATED MUSCLE ADAPTATION**	23	0.17	0.51	0.9898	0.9992
**CELL COMMUNICATION INVOLVED IN CARDIAC CONDUCTION**	35	-0.18	-0.66	0.9427	0.9992
**MUSCLE CELL MIGRATION**	18	-0.16	-0.48	0.9951	0.9992
**REGULATION OF MYELOID CELL APOPTOTIC PROCESS**	19	-0.22	-0.66	0.9115	0.9992
**GLUTATHIONE TRANSFERASE ACTIVITY**	27	0.16	0.50	0.9895	0.9992
**OXIDOREDUCTASE ACTIVITY ACTING ON PEROXIDE AS ACCEPTOR**	35	0.15	0.49	0.9984	0.9994
**MORPHOGENESIS OF A POLARIZED EPITHELIUM**	24	-0.21	-0.69	0.8875	0.9994
**SPINDLE LOCALIZATION**	35	0.15	0.50	0.9902	0.9995
**REGULATION OF NITRIC OXIDE SYNTHASE BIOSYNTHETIC PROCESS**	17	-0.22	-0.66	0.9113	0.9995
**CARDIAC MUSCLE CELL CONTRACTION**	29	0.14	0.45	0.9967	0.9995
**POSITIVE REGULATION OF EPITHELIAL TO MESENCHYMAL TRANSITION**	29	0.16	0.51	0.9850	0.9995
**MITOGEN ACTIVATED PROTEIN KINASE KINASE KINASE BINDING**	16	-0.22	-0.63	0.9397	0.9995
**RESPONSE TO GLUCAGON**	43	-0.18	-0.66	0.9676	0.9995
**NEGATIVE REGULATION OF PHOSPHOPROTEIN PHOSPHATASE ACTIVITY**	15	-0.16	-0.46	0.9957	0.9995
**P53 BINDING**	55	0.14	0.50	1.0000	0.9995
**GLUCOSE CATABOLIC PROCESS**	27	-0.19	-0.63	0.9589	0.9996

**REGULATION OF INTERLEUKIN 4 PRODUCTION**	25	-0.15	-0.48	0.9948	0.9996
**EXTRACELLULAR GLUTAMATE GATED ION CHANNEL ACTIVITY**	19	-0.20	-0.62	0.9578	0.9997
**EMBRYONIC CRANIAL SKELETON MORPHOGENESIS**	44	-0.17	-0.66	0.9423	0.9997
**CALCIUM ACTIVATED POTASSIUM CHANNEL ACTIVITY**	16	0.19	0.51	0.9892	0.9997
**NEGATIVE REGULATION OF EPITHELIAL CELL PROLIFERATION**	105	-0.15	-0.66	0.9965	0.9997
**MULTICELLULAR ORGANISM AGING**	24	0.14	0.43	0.9982	0.9998
**MELANOCYTE DIFFERENTIATION**	17	0.14	0.37	1.0000	0.9999
**POSITIVE REGULATION OF EXCITATORY POSTSYNAPTIC POTENTIAL**	21	-0.20	-0.63	0.9487	0.9999
**POSITIVE REGULATION OF STEM CELL DIFFERENTIATION**	44	-0.17	-0.62	0.9839	0.9999
**LIGAND GATED CALCIUM CHANNEL ACTIVITY**	16	0.14	0.39	1.0000	1.0000
**SULFUR COMPOUND TRANSMEMBRANE TRANSPORTER ACTIVITY**	23	-0.20	-0.66	0.9299	1.0000
**PROTEIN SECRETION**	103	-0.15	-0.65	0.9968	1.0000
**GAMMA AMINOBUTYRIC ACID SIGNALING PATHWAY**	23	-0.20	-0.65	0.9316	1.0000
**PROTEIN DEPOLYMERIZATION**	22	-0.20	-0.65	0.9515	1.0000
**MEMBRANE INVAGINATION**	24	-0.20	-0.64	0.9514	1.0000
**REGULATION OF ANDROGEN RECEPTOR SIGNALING PATHWAY**	20	-0.21	-0.64	0.9420	1.0000
**REGULATION OF INTERLEUKIN 8 PRODUCTION**	51	-0.16	-0.64	0.9751	1.0000
**HETEROTRIMERIC G PROTEIN COMPLEX**	29	-0.19	-0.64	0.9460	1.0000
**MULTICELLULAR ORGANISMAL RESPONSE TO STRESS**	61	-0.16	-0.64	0.9947	1.0000
**POSITIVE REGULATION OF COLLAGEN METABOLIC PROCESS**	19	-0.21	-0.64	0.9545	1.0000
**ENDOCHONDRAL BONE MORPHOGENESIS**	41	-0.17	-0.64	0.9614	1.0000
**REGULATION OF EXTRACELLULAR MATRIX ORGANIZATION**	27	-0.19	-0.64	0.9398	1.0000
**MYOSIN II COMPLEX**	23	-0.20	-0.64	0.9457	1.0000
**STEREOCILIUM BUNDLE**	35	-0.18	-0.63	0.9699	1.0000
**MEIOSIS I**	68	-0.15	-0.63	0.9917	1.0000
**ACTOMYOSIN**	58	-0.16	-0.63	0.9887	1.0000

**TONGUE DEVELOPMENT**	19	-0.21	-0.63	0.9467	1.0000
**POSITIVE REGULATION OF MULTICELLULAR ORGANISMAL METABOLIC PROCESS**	19	-0.21	-0.63	0.9426	1.0000
**REGULATION OF CELL JUNCTION ASSEMBLY**	63	-0.16	-0.63	0.9897	1.0000
